# Supplementary material for: Alteration of circadian machinery in monocytes underlies chronic kidney disease-associated cardiac inflammation and fibrosis
Source: Nat Commun. 2021 May 13;12:2783. doi: 10.1038/s41467-021-23050-x (PMC8119956; doi:10.1038/s41467-021-23050-x)
Supplement: Supplementary file 1 — Supplementary Information [file 41467_2021_23050_MOESM1_ESM.pdf]

Supplementary Information

Alteration of circadian machinery in monocytes underlies chronic kidney disease-associated cardiac inflammation and fibrosis

Yuya Yoshida[1, #], Naoya Matsunaga[1,2, #], Takaharu Nakao[1], Kengo Hamamura[1], Hideaki Kondo[3], Tomomi Ide[4], Hiroyuki Tsutsui[4], Akito Tsuruta[1], Masayuki Kurogi[1], Michio Nakaya[5], Hitoshi Kurose[5], Satoru Koyanagi[1,2], Shigehiro Ohdo[1, #, *]

1. Department of Pharmaceutics, Faculty of Pharmaceutical Sciences, Kyushu University, 3-1-1 Maidashi Higashi-ku, Fukuoka 812-8582, Japan
2. Department of Glocal Healthcare Science, Faculty of Pharmaceutical Sciences, Kyushu University, 3-1-1 Maidashi Higashi-ku, Fukuoka 812-8582, Japan
3. Center for Sleep Medicine, Saiseikai Nagasaki Hospital, Katafuchi, Nagasaki 850-0003, Japan.
4. Department of Cardiovascular Medicine, Faculty of Medical Sciences, Kyushu University, 3-1-1 Maidashi Higashi-ku, Fukuoka 812-8582, Japan
5. Department of Pharmacology and Toxicology, Facility of Pharmaceutical Sciences, Kyushu University, 3-1-1 Maidashi Higashi-ku, Fukuoka 812-8582, Japan

[#] These authors contributed equally to this work

* Correspondence should be addressed to S.O. (ohdo@phar.kyushu-u.ac.jp)

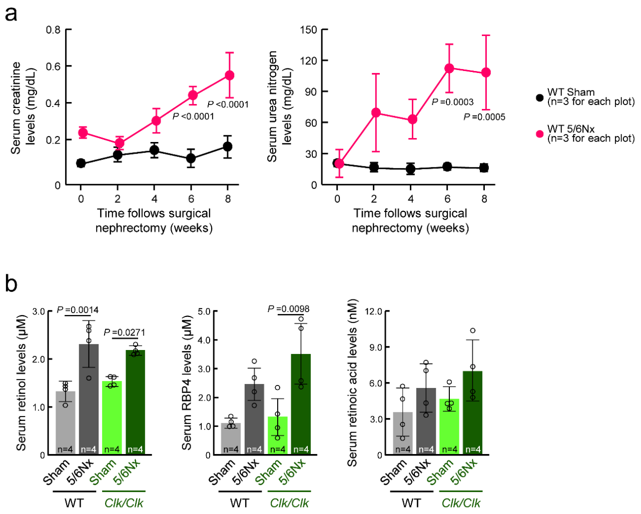

Supplementary Fig. 1 Development of renal dysfunction and alteration of retinol metabolism in wild-type (WT) and *Clk*/*Clk* mice with 5/6Nx.

(**a**) Time course of serum levels of urea nitrogen and creatinine in 5/6Nx WT mice. (**b**) Serum retinol, RBP4 and retinoic acid concentrations in wild-type (WT) and *Clk/Clk* mice with 5/6Nx. Blood samples were collected from wild-type (WT) and *Clk*/*Clk* mice with 5/6Nx 8 weeks after surgical nephrectomy. For all panels, graphs show the mean ± s.d of individual mice in independent experiments. Statistical significance was determined using two-way ANOVA with Tukey–Kramer post-hoc tests. Numbers and P-values are shown in each graph. Source data are provided as a Source Data file.

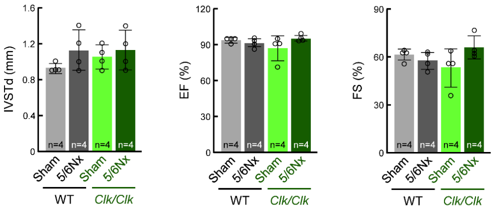

Supplementary Fig. 2 Echocardiography of wild-type and *Clk/Clk* mice with 5/6Nx

Echocardiography of wild-type (WT) or *Clk/Clk* mice with 5/6Nx. Interventricular septal thickness at end-diastole (IVSTd), ejection fraction (EF), and fractional shortening (FS) were compared between the groups. Graphs show the mean ± s.d of individual mice in independent experiments. Numbers and P-values are shown in each graph. Source data are provided as a Source Data file.

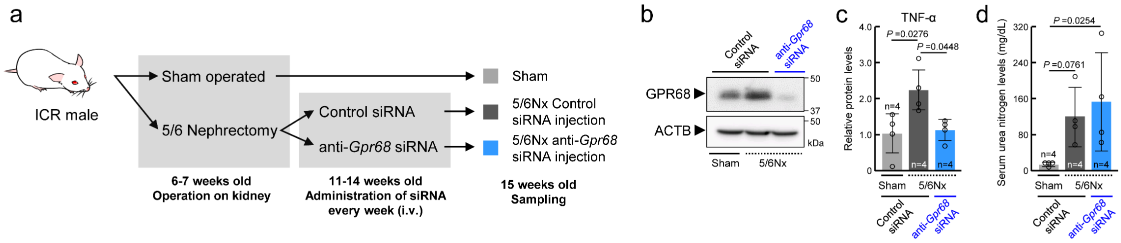

Supplementary Fig. 3 Downregulation of GPR68 in wild-type mice by injection of siRNA.

(**a**) Schematic of the experimental procedure for the downregulation of GPR68 in 5/6Nx mice. Control or anti-*Gpr68* siRNA (40 µg) encapsulated in HVJ-E was injected intravenously every week from 4 to 8 weeks after nephrectomy. (**b**) The expression levels of GPR68 in the ventricle of Sham and 5/6Nx wild-type mice after injection with control or anti-*Grp68* siRNA. Uncropped images are presented in Supplementary Fig. 22. (**c**) The expression levels of TNFα in the ventricle of Sham and 5/6Nx wild-type mice after injection with control or anti-*Grp68* siRNA. (**d**) Serum urea nitrogen levels in Sham and 5/6Nx mice after injection with control or anti-*Grp68* siRNA. For all panels, graphs show the mean ± s.d of individual mice in independent experiments. Statistical significance was determined using one-way ANOVA with Tukey–Kramer post-hoc tests. Numbers and P-values are shown in each graph. Source data are provided as a Source Data file.

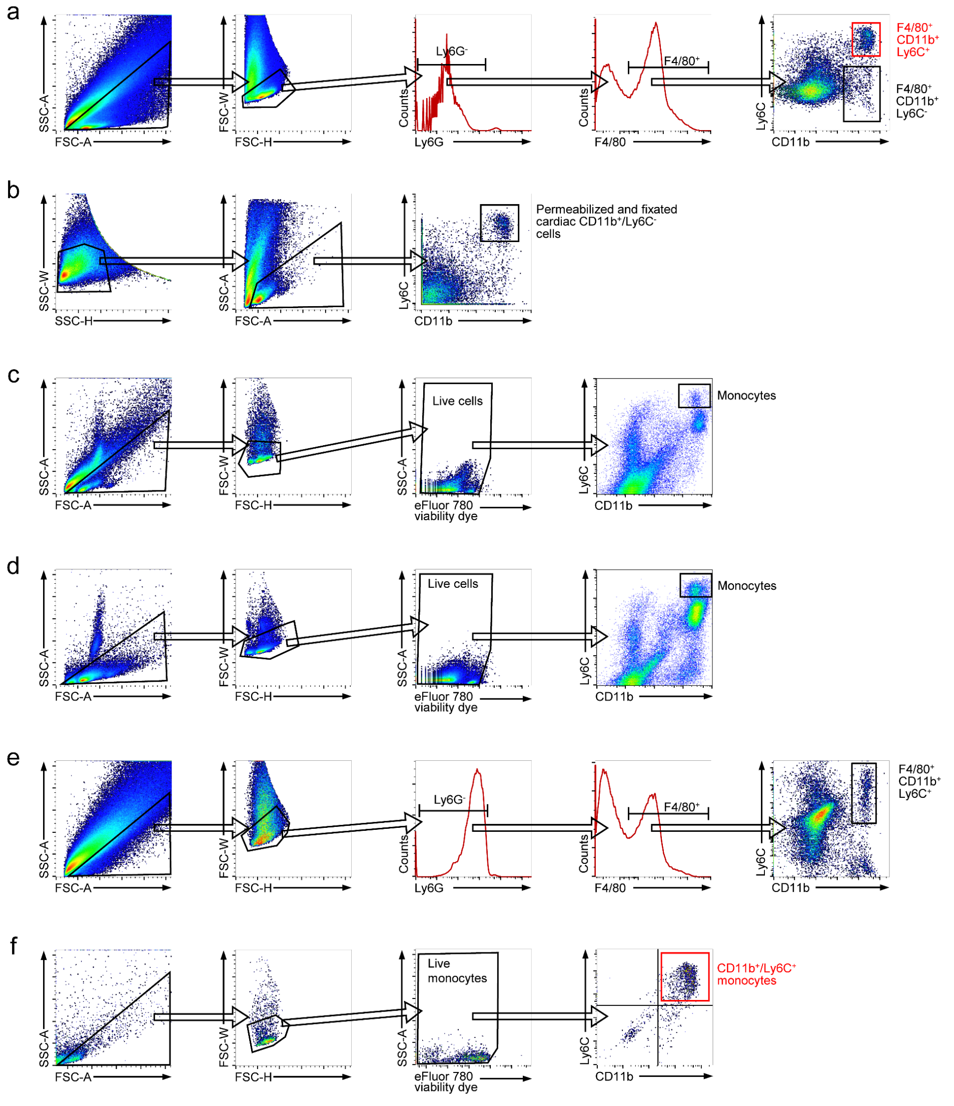

Supplementary Fig. 4 Gating strategies used for flow cytometry analysis

(**a**) Gating strategy for sorting F4/80$^+$/Ly6G$^-$/CD11b$^+$/Ly6C$^+$ cardiac cells and F4/80$^+$/Ly6G$^-$/CD11b$^+$/Ly6C$^-$ cardiac cells. The same strategy was applied to assess the number of monocytes-derived macrophages in the heart and GPR68 expression in cardiac monocytes presented on Fig. 2b, 3b, 3f, 4b, Supplementary Fig. 7b, and 12c. (**b**) Gating strategy for assessing GPR68, TNFα, and IL-6 expression in cardiac CD11b$^+$/Ly6C$^+$ cells presented on Fig. 2g. (**c**) Gating strategy for sorting CD11b$^+$/Ly6C$^+$ cells (monocytes) from circulating blood of mice. The same strategy was applied to assess GPR68 expression in circulating monocytes presented on Fig. 2h, 3a. (**d**) Gating strategy for sorting CD11b$^+$/Ly6C$^+$ cells (monocytes) from spleen and bone marrow of mice presented on Fig. 3a. (**e**) Gating strategy for sorting F4/80$^+$/Ly6G$^-$/CD11b$^+$/Ly6C$^+$ renal cells presented on Supplementary Fig. 19a. (**f**) Gating strategy to sort CD11b$^+$/Ly6C$^+$ cultured monocytes, presented on Supplementary Fig. 19d.

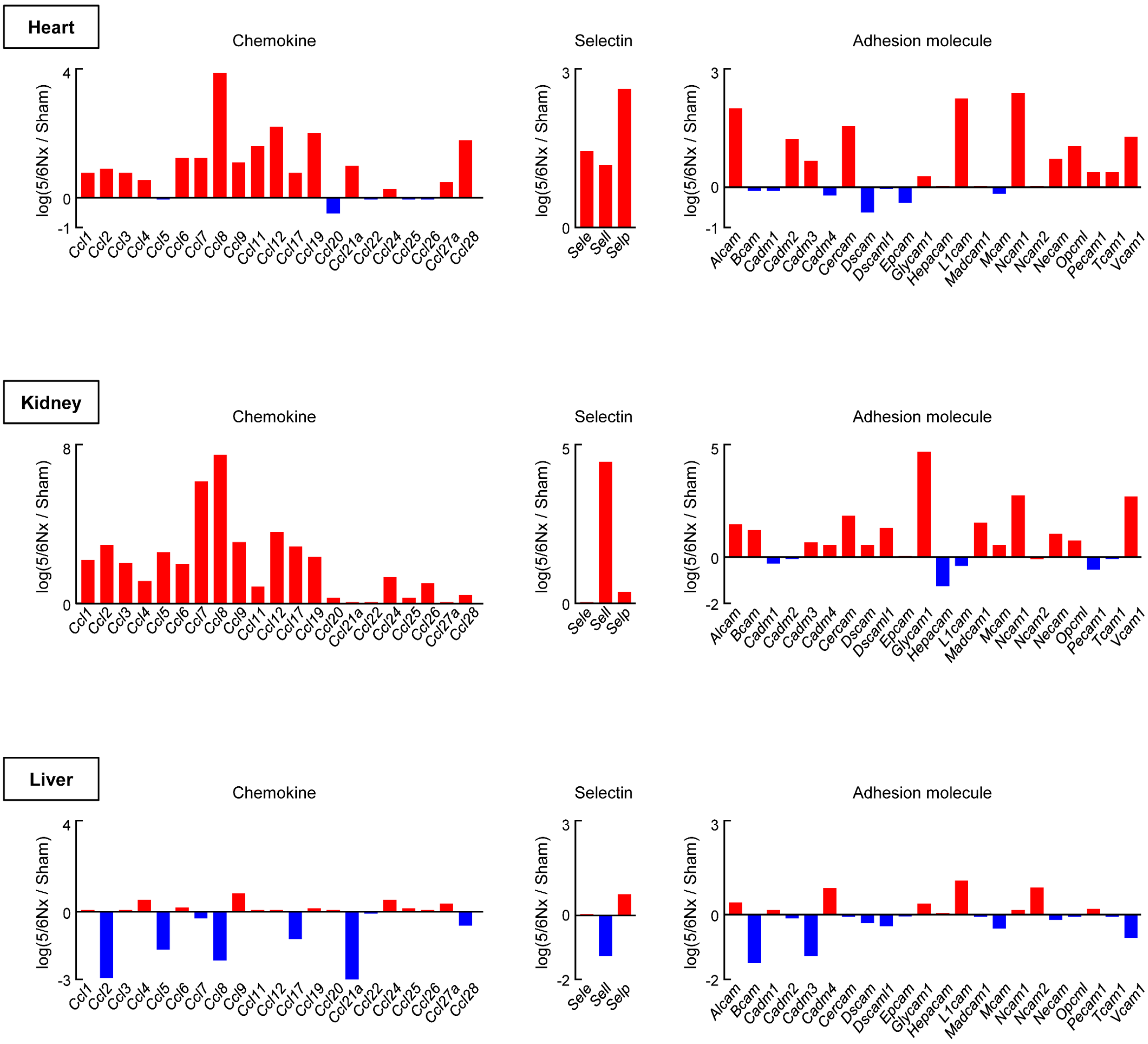

Supplementary Fig. 5 The expression profiles of mRNA for chemokine, selectin and adhesion molecules in Sham and 5/6Nx mice heart, kidney, and liver.
The expression levels of each factor were obtained by microarray analysis. Values in the panel are shown as the ratio of sham-operated group (Sham) to 5/6Nx ones (n=1 for each bar). Source data are available within the Gene Expression Omnibus at the National Center for Biotechnology Information (Cardiac ventricle: GSE150094, Kidney: GSE57799, Liver: GSE35135).

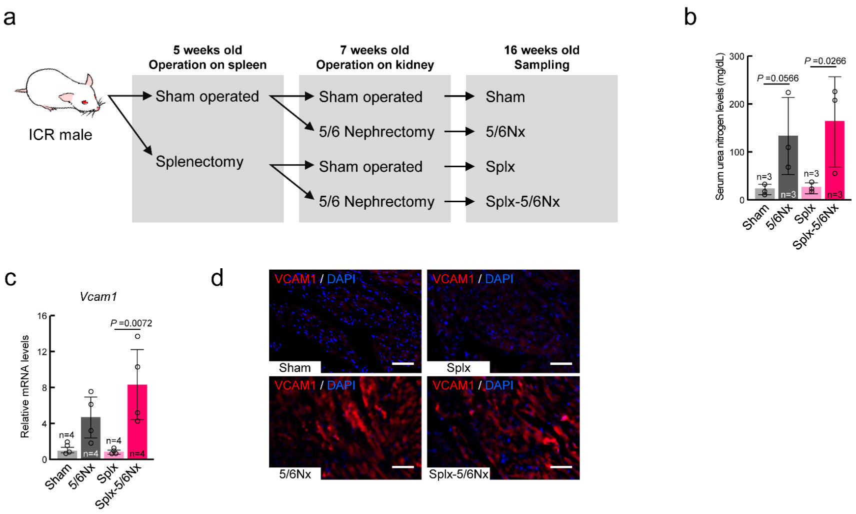

Supplementary Fig. 6 Preparation of splenectomized 5/6Nx mice.

(**a**) Schematic experimental procedure for preparation of splenectomized (Splx)-5/6Nx mice. (**b**) Serum concentrations of urea nitrogen in Sham, 5/6Nx, Splx, and Splx-5/6Nx mice. (**c**) The mRNA levels of *Vcam1* in the cardiac ventricle of Sham, 5/6Nx, Splx, and Splx-5/6Nx mice. The mean value of the sham-operated wild-type group was set as 1.0. (**d**) Immunofluorescence labeling of VCAM1 with DAPI in the ventricle slices prepared from Sham, 5/6Nx, Splx, and Splx-5/6Nx mice. The scale bar indicates 100 µm. For all panels, graphs show the mean ± s.d of individual mice in independent experiments. Statistical significance was determined using one-way ANOVA with Tukey–Kramer post-hoc tests. Numbers and P-values are shown in each graph. Source data are provided as a Source Data file.

.

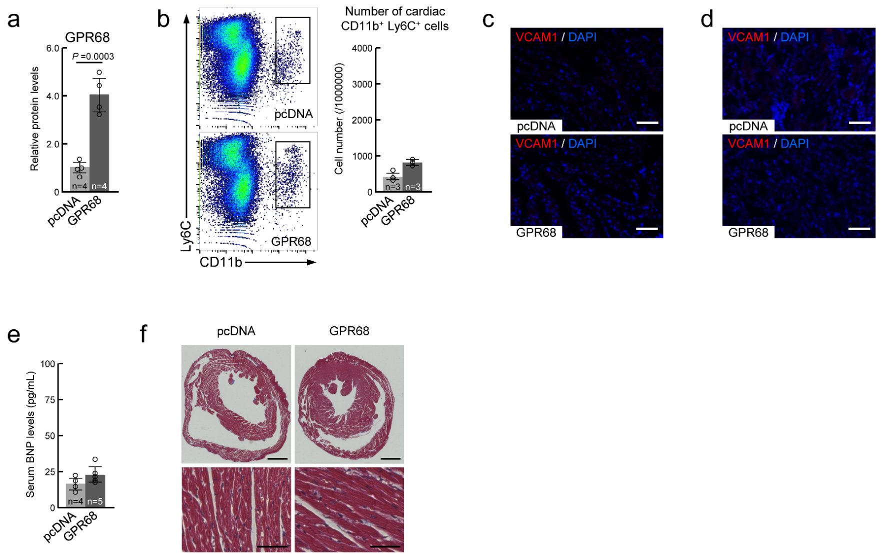

Supplementary Fig. 7 Influence of the transduction of GPR68-expressing plasmids on normal healthy mice.

(**a**) The protein levels of GPR68 in circulating monocytes prepared from normal healthy mice injected with pcDNA3.1 or GPR68-expressing plasmid. Each plasmid was encapsulated in HVJ-E and was injected intravenously every three days for two weeks. (**b**) The number of cardiac migrated CD11b$^+$/Ly6C$^+$ cells in mice injected with pcDNA3.1 or GPR68-expressing plasmid. (**c** and **d**) Immunofluorescence labeling of VCAM1 with DAPI in the ventricle (c) and kidney (d) slices prepared from mice injected with pcDNA3.1 or GPR68-expressing plasmid. The scale bar indicates 100 μm. (**e**) Serum BNP levels in mice injected with pcDNA3.1 or GPR68-expressing plasmid. (**f**) The Masson's trichrome staining performed using heart ventricle. Scale bars indicate 1 mm (upper panel) and 50 μm (lower panel). For all panels, graphs show the mean ± s.d of individual mice in independent experiments. Statistical significance was determined using two-tailed Student's-t tests. Numbers and P-values are shown in each graph. Source data are provided as a Source Data file.

.

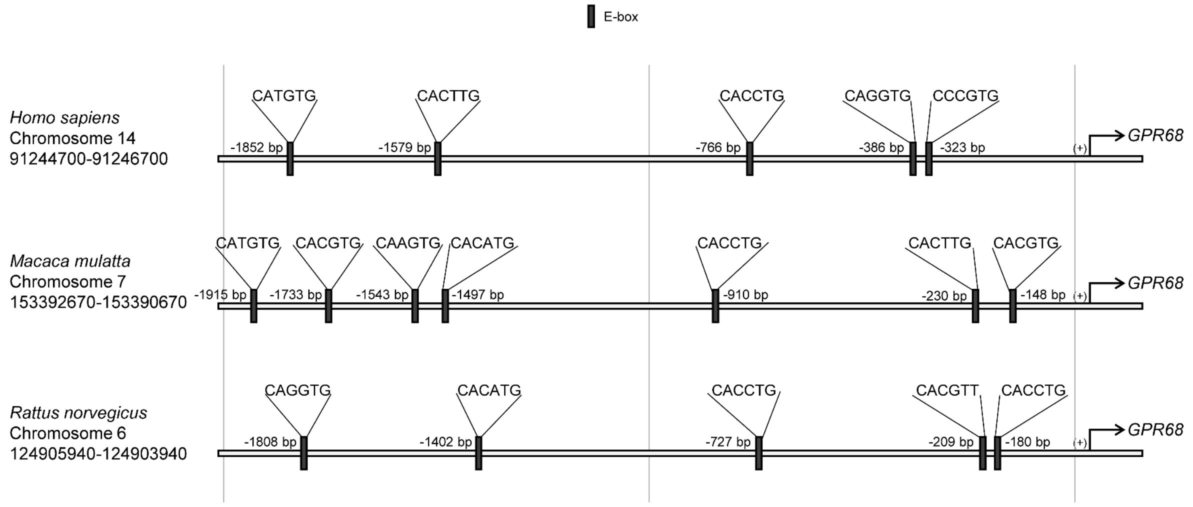

Supplementary Fig. 8 Schematic representation of the E-boxes within the upstream region of the *GPR68* gene of human, rhesus monkey, and rat.

The numbers indicate the distance from the transcription start site (+1). Black rectangles, E-box.

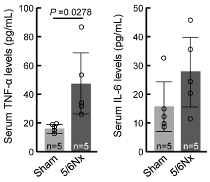

Supplementary Fig. 9 Serum concentrations of TNFα and IL-6 in Sham and 5/6Nx mice.
Blood samples were collected from Sham and 5/6Nx mice at 8 weeks after operation. Graphs show the mean ± s.d of individual mice in independent experiments. Statistical significance was determined using two-tailed Student's-t tests. Numbers and P-values are shown in each graph. Source data are provided as a Source Data file.

.

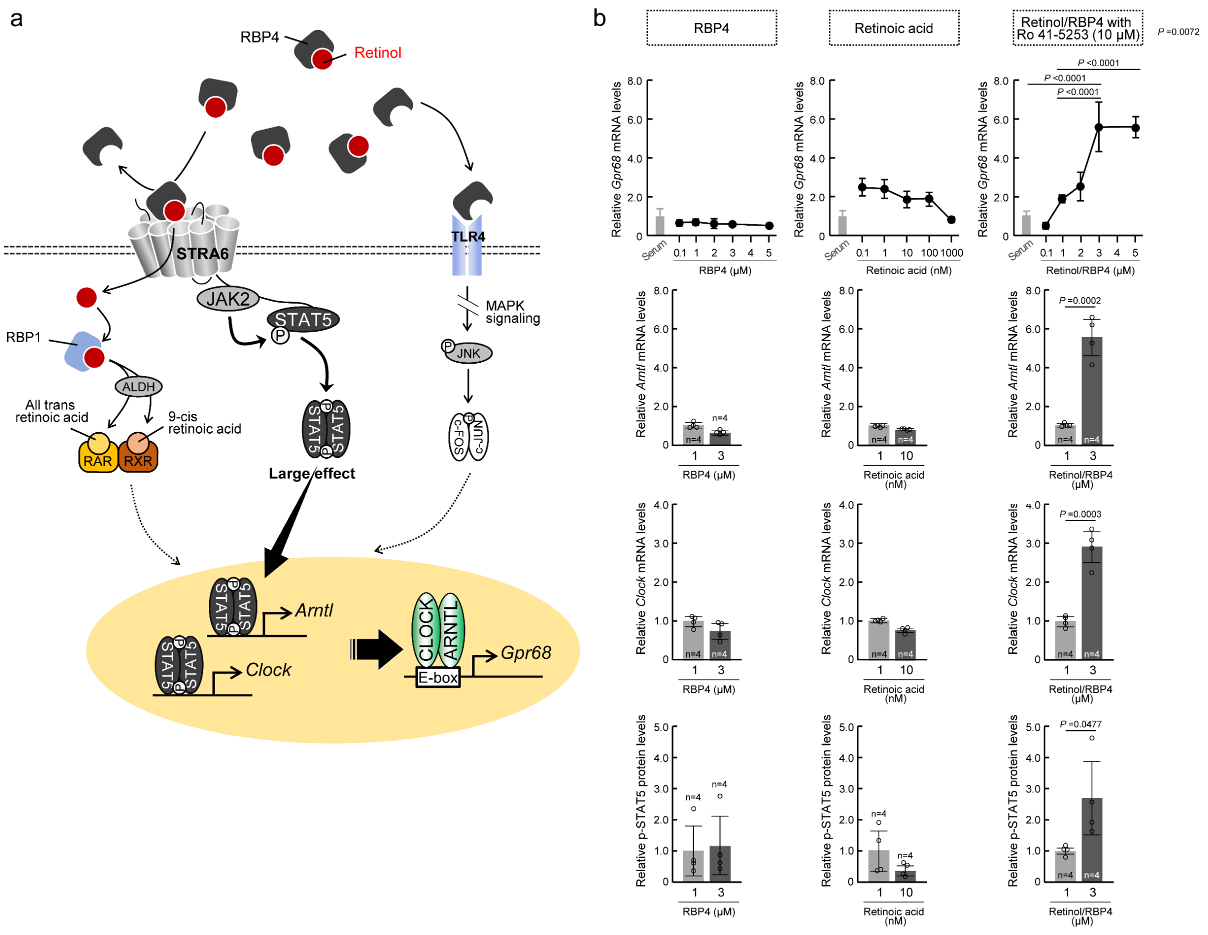

Supplementary Fig. 10 Influence of other retinoid-related signaling on GPR68 expression.
(**a**) Schematic diagram indicating the mechanism of retinol/RBP4-induced expression GPR68 in monocytes. (**b**) Influence of RBP4, retinoic acid, and RAR inhibitor Ro 41-5253 on the mRNA expression of *Gpr68, Clock*, *Arntl,* and phosphorylation of STAT5 in mouse primary cultured monocytes. Cells were treated with each compound for 24 h. Graphs show the mean ± s.d in independent experiments (n=4 for each group). Statistical significance was determined using one-way ANOVA with Dunnett’s post-hoc tests (upper polygonal line graph) or two-tailed Student’s-t tests (the other bar graphs). Numbers and P-values are shown in each graph. Source data are provided as a Source Data file.

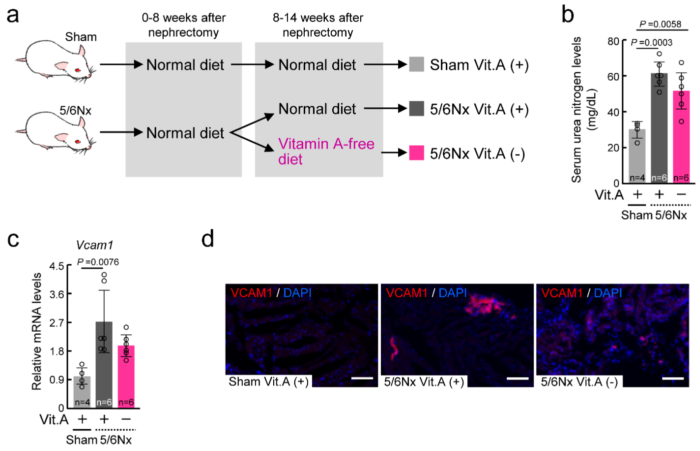

Supplementary Fig. 11 Influence of dietary deficiency of vitamin A on 5/6Nx mice.
(**a**) Schematic experimental procedure for feeding of vitamin A-free diet to Sham and 5/6Nx mice. (**b**) Serum concentrations of urea nitrogen in Sham and 5/6Nx mice fed with normal or vitamin A-free diet. (**c**) The mRNA levels of *Vcam1* in the cardiac ventricle of Sham and 5/6Nx mice fed with normal or Vitamin A-free diet. The mean value of the Sham mice fed with normal diet group was set as 1.0. (**d**) Immunofluorescence labeling of VCAM1 with DAPI in the ventricle slices prepared from Sham and 5/6Nx mice fed with normal or Vitamin A-free diet. The scale bar indicates 100 µm. For all panels, graphs show the mean ± s.d of individual mice in independent experiments. Statistical significance was determined using one-way ANOVA with Tukey–Kramer post-hoc tests. Numbers and P-values are shown in each graph. Source data are provided as a Source Data file.

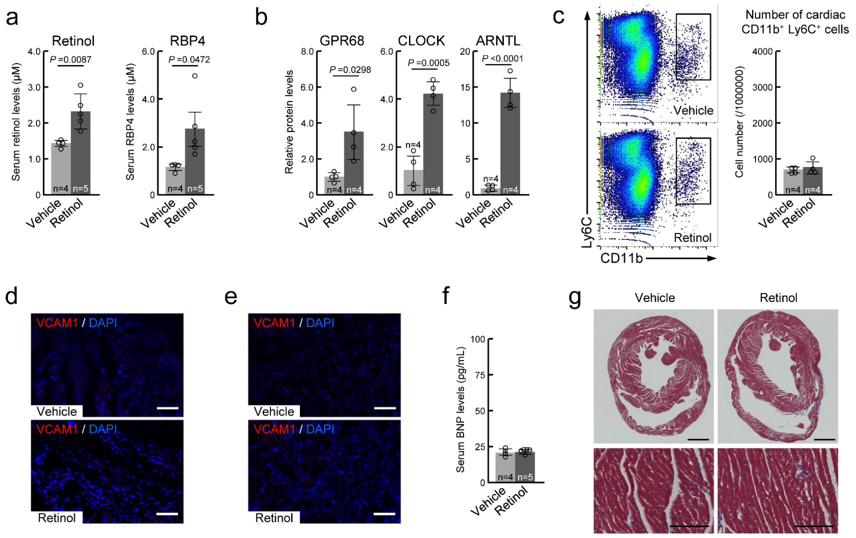

Supplementary Fig. 12 Influence of retinol administration on monocytic GPR68 expression and cardiac function of normal healthy mice.

(**a**) Serum retinol and RBP4 concentrations in normal mice administrated intraperitoneally with 100 μL of retinol (25mM) or vehicle (DMSO). Retinol was administrated every three days for two weeks. (**b**) The protein levels of GPR68, CLOCK and ARNTL in circulating monocytes prepared from retinol- or vehicle-administrated mice. (**c**) The number of cardiac migrated CD11b$^+$/Ly6C$^+$ cells in retinol- or vehicle-administrated mice. (**d** and **e**) Immunofluorescence labeling of VCAM1 with DAPI in the ventricle (c) and kidney (d) slices prepared from retinol- or vehicle-administrated mice. The scale bar indicates 100 μm. (**f**) Serum BNP levels in retinol- or vehicle-administrated mice. (**g**) The Masson's trichrome staining performed using heart ventricle prepared from retinol- or vehicle-administrated mice. Scale bars indicate 1 mm (upper panel) and 50 μm (lower panel). For all panels, graphs show the mean ± s.d of individual mice in independent experiments. Statistical significance was determined using two-tailed Student's-t tests. Numbers and P-values are shown in each graph. Source data are provided as a Source Data file.

Mouse *Clock* gene (Chromosome 5, 76304889 to 76304920)

```
TTCCTCGGGTGCCCGGGATCCCCAGGCGCGGC
||||||||| | ||||||||||| ||||||||
TTCTTCGGGCGTCCGGGATCCCCTGGCGCGGC
```

Human *CLOCK* gene (Chromosome 4, 55547023 to 55547053)

Mouse *Arntl* gene (Chromosome 7, 113206878 to 113206918)

```
GACTAGGACAAGCCAGGGGTTTCAGGAAGGTTCTGTCACTC
||||  | ||| |||||| ||| ||||||| |||| |||||
GACTTTGGCAAACCAGGGATTTTAGGAAGGGTCTGGCACTC
```

Human *ARNTL* gene (Chromosome 11, 13277142 to 13277184)

Supplementary Fig. 13 Sequence comparison of STAT5 binding sites within the upstream region of CLOCK and ARNTL genes between humans and mice. Mouse and human nucleotide sequences near the STAT5-binding sites upstream of CLOCK (upper) and ARNTL (lower) genes. Sequence written in red font indicate STAT5-binding sites.

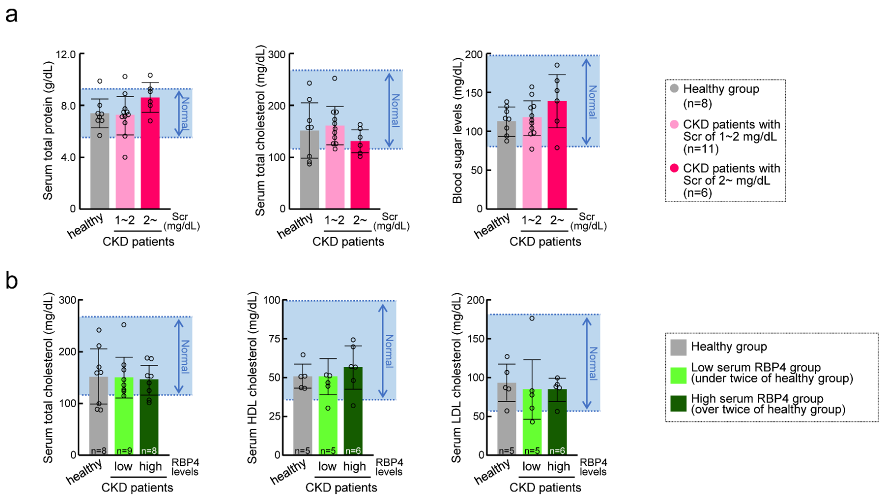

Supplementary Fig. 14 The levels of biochemical parameters in the serum collected from healthy subjects and CKD patients.

(**a**) Serum levels of total protein, total cholesterol and sugar in healthy subjects and CKD patients stratified by serum creatinine levels. (**b**) Serum levels of total-, HDL-, and LDL-cholesterol in healthy subjects and CKD patients stratified by serum RBP4 levels. For all panels, graphs show the mean ± s.d of individual human samples. Number are shown in each graphs. All values of measured parameters in blood were described in Supplementary table 8.

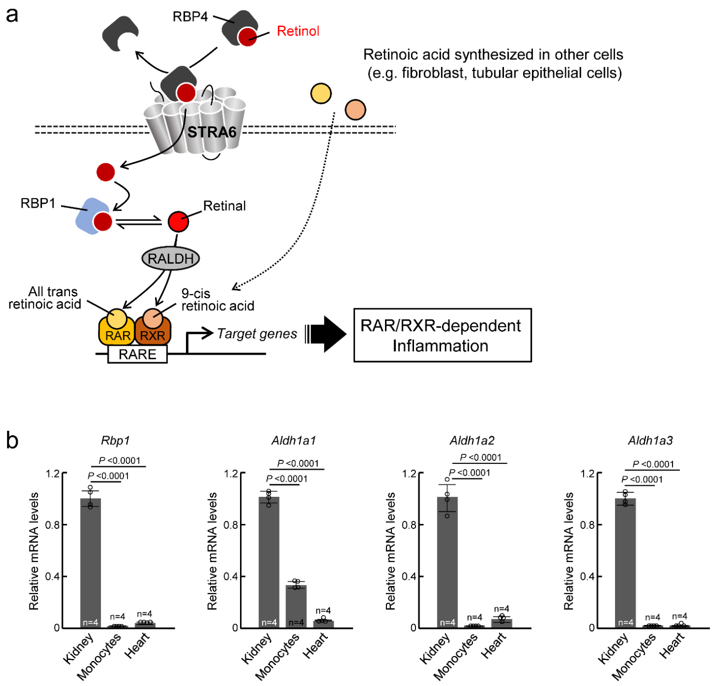

Supplementary Fig. 15 The mRNA expression profile of retinol metabolism-related gene in 5/6Nx mice.

(**a**) Schematic diagram indicating the mechanism of cellular uptake and metabolism of retinol to retinoic acid. (**b**) The mRNA levels of *Aldh1a1*, *1a2*, *1a3* and *Rbp1* in kidney, monocytes, and heart of 5/6Nx mice. Graphs show the mean ± s.d of individual mice in independent experiments. Statistical significance was determined using one-way ANOVA with Tukey–Kramer post-hoc tests. Numbers and P-values are shown in each graph. Source data are provided as a Source Data file.

a

Kidney

KEGG pathway	%	P-value
Staphylococcus aureus infection	1.9	6.7E-14
Complement and coagulation cascades	2.1	2.5E-11
Cytokine-cytokine receptor interaction	3.6	1.1E-09
Cell adhesion molecules (CAMs)	2.8	2.1E-09
Chemokine signaling pathway	3.0	7.4E-09

Liver

KEGG pathway	%	P-value
Retinol metabolism	2.9	2.9E-08
Chemical carcinogenesis	2.4	9.3E-08
Steroid hormone biosynthesis	2.1	1.9E-06
Arachidonic acid metabolism	2.1	2.5E-06
Linoleic acid metabolism	9.6	3.7E-05

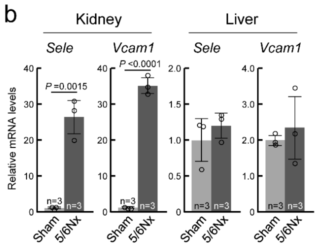

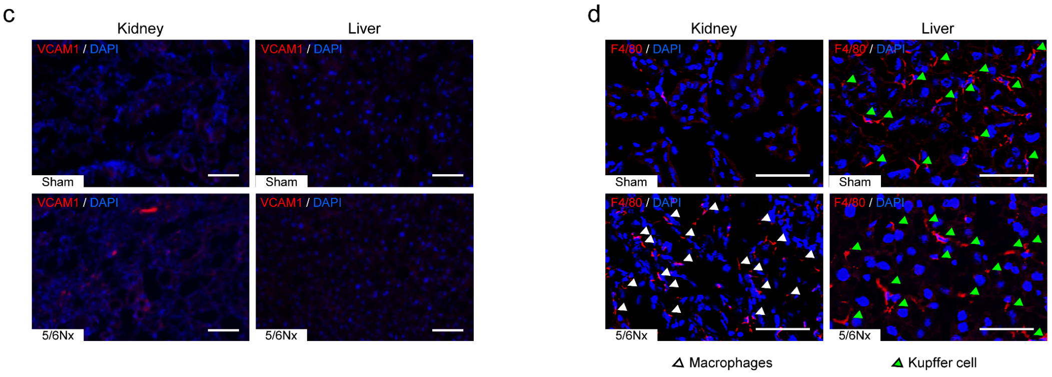

Supplementary Fig. 16 Influence of 5/6Nx on monocyte infiltration into the kidney and liver. (**a**) Functional analysis of the genes whose expression was changed in the kidney and liver of 5/6Nx mice using the KEGG database. (**b**) The mRNA levels of *Sele* and *Vcam1* in the kidney and liver of 5/6Nx mice. The mean value of each Sham group was set as 1.0. Graphs show the mean ± s.d of individual mice in independent experiments. Statistical significance was determined using two-tailed Student's-t tests. Numbers and P-values are shown in each graph. (**c**) Immuno-fluorescence labeling of VCAM1 (red) in the kidney and liver of Sham and 5/6Nx mice. The scale bar indicates 100 µm. (**d**) Immunofluorescence labeling of F4/80 (red) in the kidney and liver of Sham and 5/6Nx mice. The scale bar indicates 100 µm. Red fluorescence shown by white arrows indicate migrated monocyte in the kidney, whereas fluorescence shown by green arrows indicate resident Kupffer cells in the liver. Source data are provided as a Source Data file.

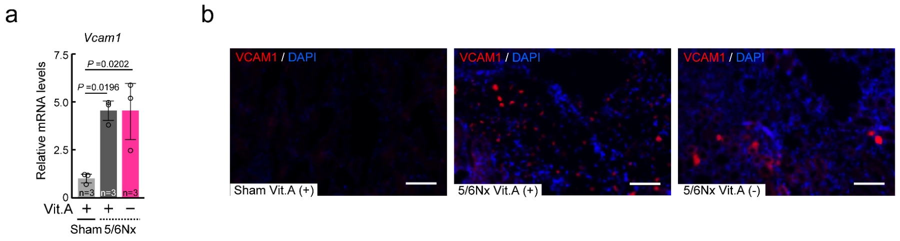

Supplementary Fig. 17 Influence of dietary deficiency of vitamin A on the expression of adhesion molecules in the kidney of 5/6Nx mice.

(**a**) The mRNA levels of *Vcam1* in the kidney of Sham and 5/6Nx mice fed with normal or vitamin A-free diet. The mean value of the Sham mice fed with normal diet group was set as 1.0. The graph shows the mean ± s.d of individual mice in independent experiments. Statistical significance was determined using one-way ANOVA with Tukey–Kramer post-hoc tests. Number and P-values are shown in graph. (**b**) Immunofluorescence labeling of VCAM1 with DAPI in the kidney slices prepared from Sham and 5/6Nx mice fed with normal or vitamin A-free diet. The scale bar indicates 100 μm. Source data are provided as a Source Data file.

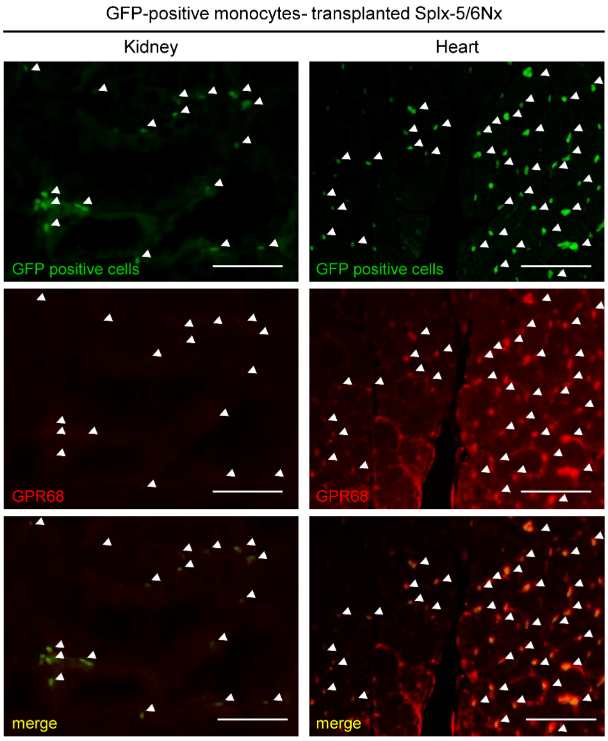

Supplementary Fig. 18 Infiltration of allogeneic transplanted-GFP reporter expressing monocytes in the kidney and heart of Splx- 5/6Nx mice.

Monocytes infected with lentivirus-expressing GFP reporter were intravenously injected into Splx-5/6Nx mice every 3 days for 2 weeks after the 5/6 nephrectomy operation. Immunofluorescence labeling of GFP (green) and GPR68 (red) was conducted using slices prepared from kidney and heart of Splx-5/6Nx mice at 2 weeks after initiating the injection of monocytes. White arrows indicate the position of GFP-positive monocytes. The scale bar indicates 50 μm.

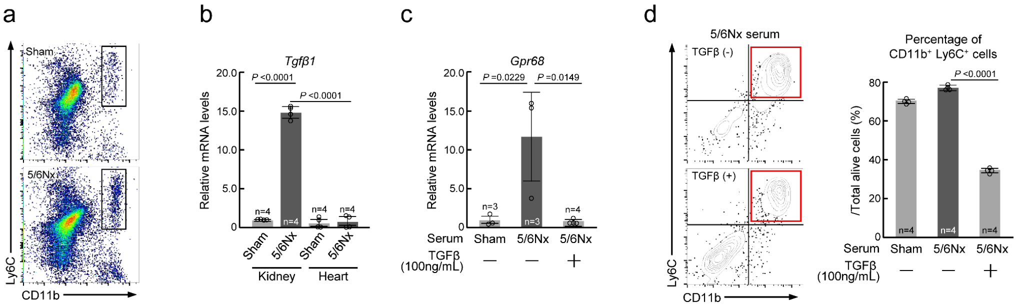

Supplementary Fig. 19 Influence of TGFβ on the expression of GPR68 in monocytes.
(**a**) The flowcytometry profile of CD11b^{+}/Ly6C^{+} cells in the kidney of Sham and 5/6Nx wild-type mice. (**b**) The mRNA levels of *Tgfβ1* in the cardiac ventricle and kidney of Sham and 5/6Nx mice. The mean value of Sham group was set as 1.0. Data are the mean ± s.d from individual mice. (**c**) The expression of *Gpr68* mRNA in primary cultured monocytes after incubation in the media containing 10% serum collected from Sham and 5/6Nx mice with the presence or absence of 100 ng/mL of TGFβ for 24 h. The mean value of Sham group was set as 1.0. (**d**) The percentage of CD11b^{+}/Ly6C^{+} cells in mouse primary cultured monocytes incubated in the media containing 10% serum collected from Sham and 5/6Nx mice with the presence or absence of 100 ng/mL of TGFβ for 24 h. For all panels, graphs show the mean ± s.d in independent experiments. Statistical significance was determined using two-way ANOVA (**b**), or one-way ANOVA (**c, d**) with Tukey–Kramer post-hoc tests. Numbers and P-values are shown in each graph. Source data are provided as a Source Data file.

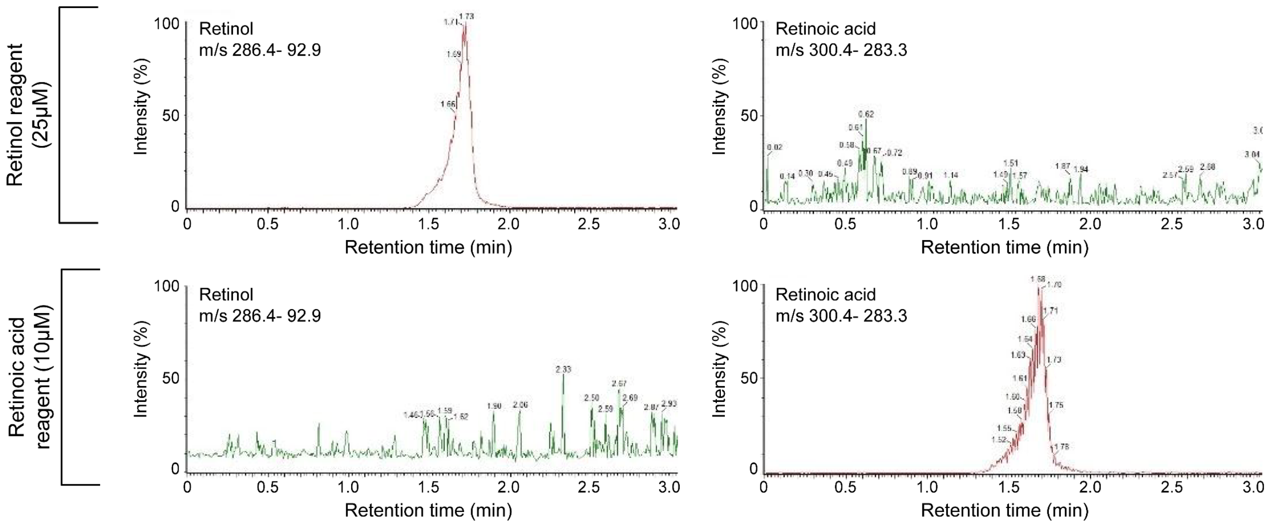

Supplementary Fig. 20 LC-MS/MS chromatograms from retinol and retinoic acid reagents used in the present study.

The contents of retinoic acid in retinol reagent were less than 0.7 %.

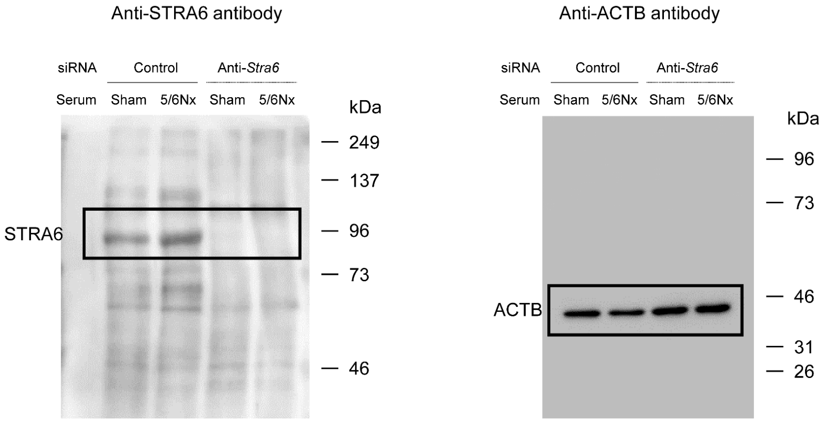

Supplementary Fig. 21 Uncropped images of Figure. 6k

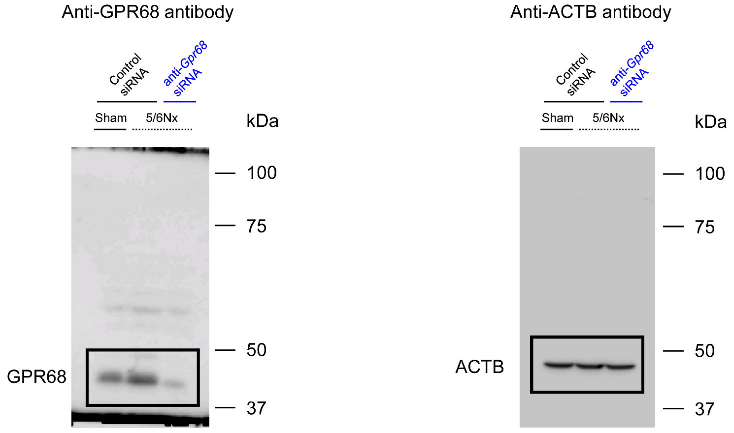

Supplementary Fig. 22 Uncropped images of Supplementary Fig. 3b

Supplementaly Table 1: Rhythmical expression genes by LombScargle analysis using CircaDB

Probeset_ID	Symbol	LombScargle P value	LombScargle Q value	LombScargle period (hr)	LombScargle phase (hr)
10518781	*Per3*	0.000365	0.630717	23.064	10.48
10487040	*Fbn1*	0.000417	0.630717	23.064	2.98
10417734	*Nr1d2*	0.000429	0.630717	23.064	10.1
10425601	*Tef*	0.000429	0.630717	23.064	13.33
10556463	*Arntl*	0.000488	0.630717	23.064	22.42
10553092	*Dbp*	0.000509	0.630717	23.064	10.07
10356601	*Per2*	0.000512	0.630717	23.064	11.94
10530733	*Clock*	0.000514	0.630717	23.064	22.85
10389786	*Hlf*	0.000518	0.630717	23.064	11.58
10558961	*Tspan4*	0.000524	0.630717	23.064	10.43
10365482	*Timp3*	0.000524	0.630717	23.064	14.31
10363773	*Rhobtb1*	0.000545	0.630717	23.064	13.45
10459496	*Ccbe1*	0.000569	0.630717	23.892	11.2
10540059	*Slc41a3*	0.000586	0.630717	23.892	0.94
10380887	*Tcap*	0.000605	0.630717	23.064	10.01
10390691	*Nr1d1*	0.000725	0.630717	23.064	6.8
10600502	*2810453I06Rik*	0.000787	0.630717	23.892	22.99
10359582	*Fmo2*	0.000788	0.630717	23.892	15.91
10487945	*Gpcpd1*	0.000813	0.630717	22.291	11.23
10528548	*Kcnh2*	0.000816	0.630717	24.783	10.62
10534168	*Auts2*	0.000832	0.630717	23.064	22.15
10598638	*Mid1ip1*	0.000833	0.630717	23.892	15.34
10345675	*Npas2*	0.000856	0.630717	23.064	23
10586722	*F830001A07Rik*	0.00086	0.630717	22.291	14.17
10604751	*Fgf13*	0.000863	0.630717	23.892	10.16
10483000	*Itgb6*	0.00088	0.630717	23.892	23.27
10440993	*Rcan1*	0.000899	0.630717	23.892	22.34
10418729	*Colq*	0.00093	0.630717	23.064	22.49
10349249	*Clasp1*	0.000943	0.630717	23.064	22.52
10584634	*Usp2*	0.000955	0.630717	23.064	10.85
10556266	*Wee1*	0.000971	0.630717	23.064	15.1
10371387	*Ckap4*	0.000986	0.630717	22.291	22.37
10492469	*Mlf1*	0.000994	0.630717	23.892	10.3
10530692	*Kdr*	0.000998	0.630717	23.064	23.44
10541532	*Foxj2*	0.00101	0.630717	23.064	22.12
10473384	*Slc43a3*	0.00103	0.630717	23.892	15.35
10411782	*Pik3r1*	0.00112	0.630717	23.892	15.81
10349773	*Klhdc8a*	0.00122	0.630717	22.291	22.8
10346298	*Coq10b*	0.00125	0.630717	22.291	10.26
10488382	*Cd93*	0.00127	0.630717	23.892	23
10398665	*Tnfaip2*	0.00131	0.630717	22.291	0.5
10426042	*Gramd4*	0.00135	0.630717	23.892	1.77
10527229	*2810453I06Rik*	0.00138	0.630717	23.892	23.07
10530806	*Ppat*	0.0014	0.630717	23.064	10.08

10356628	*Hdac4*	0.00151	0.630717	23.064	13.27
10584561	*9030425E11Rik*	0.00152	0.630717	23.892	1.85
10408543	*Mylk4*	0.00155	0.630717	23.892	10.68
10396476	*Rhoj*	0.0016	0.630717	24.783	23.99
10485117	*Creb3l1*	0.00164	0.630717	23.064	23.66
10415052	*Mmp14*	0.00167	0.630717	23.064	0.87
10565775	*Dgat2*	0.00167	0.630717	23.892	15.75
10363224	*Fabp7*	0.00171	0.630717	23.892	2.99
10381006	*Thra*	0.00189	0.630717	22.291	0.14
10351430	*Rxrg*	0.00193	0.630717	23.064	22.45
10587211	*Leo1*	0.00203	0.630717	23.064	22.79
10550332	*Slc1a5*	0.00205	0.630717	23.064	13.33
10388884	*Nlk*	0.00206	0.630717	24.783	23.9
10363173	*Gja1*	0.00207	0.630717	23.892	1.71
10457508	*Npc1*	0.00208	0.630717	23.064	10.56
10388938	*Wsb1*	0.0021	0.630717	23.064	23.67
10461251	*Lrrn4cl*	0.00211	0.630717	23.064	10.65
10395273	*Gdap10*	0.00218	0.630717	22.291	10.17
10549276	*Bhlhe41*	0.00221	0.630717	22.291	10.37
10578207	*Lonrf1*	0.00222	0.630717	23.892	15.38
10368748	*Amd1*	0.00223	0.630717	23.892	15.91
10368670	*Amd1*	0.00223	0.630717	24.783	15.87
10374035	*Xbp1*	0.00224	0.630717	23.064	22.61
10543233	*Ppp1r3a*	0.00225	0.630717	23.064	15.57
10592217	*Stt3a*	0.0023	0.630717	23.892	1.4
10434229	*Cldn5*	0.00234	0.630717	23.892	22.63
10353117	*Slco5a1*	0.00241	0.630717	23.892	15.83
10399696	*Rnf144a*	0.00243	0.630717	23.064	0.52
10485170	*Cry2*	0.00248	0.630717	23.064	13.82
10480035	*Pfkfb3*	0.00249	0.630717	22.291	10.06
10476033	*Stk35*	0.0025	0.630717	22.291	14.03
10485198	*Tspan18*	0.00255	0.630717	23.892	23.75
10553324	*Tmem86a*	0.00257	0.630717	23.892	6.11
10517312	*Tmem57*	0.0026	0.630717	23.892	15.87
10404376	*Agtr1a*	0.00265	0.630717	23.892	7.06
10512935	*Amd1*	0.00277	0.630717	24.783	15.89
10471555	*Angptl2*	0.00279	0.630717	23.064	10.75
10353878	*Ankrd23*	0.00284	0.630717	23.892	23.15
10473444	*Aplnr*	0.00284	0.630717	23.892	1.54
10459389	*Amd1*	0.00288	0.630717	24.783	15.91
10574023	*Mt2*	0.0029	0.630717	22.291	10.12
10359754	*Mpzl1*	0.00291	0.630717	23.064	1.52
10602385	*Pfkfb1*	0.00297	0.630717	23.892	9.54
10461629	*Ms4a4d*	0.00301	0.630717	22.291	10.87
10551469	*Dyrk1b*	0.00301	0.630717	22.291	10.29
10586718	*9530091C08Rik*	0.00305	0.630717	22.291	13.77
10538927	*Amd1*	0.00306	0.630717	23.892	15.8
10346015	*Col3a1*	0.00312	0.630717	22.291	3.19
10377439	*Per1*	0.00313	0.630717	23.064	9.92

10596575	*Manf*	0.00318	0.630717	22.291	22.3
10543785	*AB041803*	0.0032	0.630717	23.064	9.26
10362294	*Arhgap18*	0.00321	0.630717	23.892	0.03
10456622	*Mbd1*	0.00322	0.630717	23.064	14.66
10492330	*P2ry1*	0.00334	0.630717	24.783	17.15
10599927	*Aff2*	0.00335	0.630717	23.892	9.57
10529264	*Spon2*	0.00336	0.630717	22.291	23
10393364	*Cygb*	0.00341	0.630717	23.064	10.4
10605493	*Prrg1*	0.00342	0.630717	22.291	7.89
10357345	*Nckap5*	0.00346	0.630717	23.892	5.71
10538932	*Amd1*	0.00348	0.630717	23.892	15.83
10437516	*Ppl*	0.00349	0.630717	23.064	10.85
10576973	*Col4a1*	0.00349	0.630717	22.291	0.24
10549420	*Tmtc1*	0.00353	0.630717	23.892	16.07
10572786	*Ap1m1*	0.00353	0.630717	23.064	1.4
10523281	*Sep11*	0.00364	0.636174	23.892	22.93
10502805	*Ptgfr*	0.00368	0.636901	23.892	15.56
10387797	*Bcl6b*	0.00369	0.636901	23.064	0.42
10418842	*3425401B19Rik*	0.00375	0.636975	22.291	10.2
10471586	*Hspa5*	0.00376	0.636975	22.291	22.26
10418702	*Sh3bp5*	0.0039	0.640316	23.892	4.09
10469404	*Cacnb2*	0.00392	0.640316	23.064	8.06
10447951	*Thbs2*	0.00398	0.640316	22.291	10.53
10455259	*Arhgap26*	0.00405	0.640316	23.892	14.47
10606989	*Tsc22d3*	0.00406	0.640316	23.892	15.3
10531407	*Cxcl9*	0.00409	0.640316	22.291	6.49
10404874	*Mylip*	0.00409	0.640316	23.064	11.89
10498978	*Lrat*	0.00409	0.640316	23.064	10.38
10593225	*Zbtb16*	0.00412	0.640316	23.064	15.51
10416181	*Stc1*	0.00413	0.640316	23.892	10.11
10463997	*Pdcd4*	0.00416	0.640316	22.291	7.66
10352914	*A330023F24Rik*	0.00416	0.640316	23.892	9.72
10531869	*Mapk10*	0.0042	0.641816	22.291	12.21
10417946	*Sec24c*	0.00424	0.641816	23.064	8.88
10475335	*Pdia3*	0.00426	0.641816	23.892	0.33
10397476	*2310044G17Rik*	0.00431	0.64523	23.892	15.86
10562500	*Dpy19l3*	0.00436	0.64523	23.892	2.34
10381304	*Vps25*	0.0045	0.64523	22.291	10.11
10584712	*Hyou1*	0.00458	0.64523	22.291	22.06
10498379	*Igsf10*	0.00465	0.64523	23.892	2.85
10497817	*Anxa5*	0.00468	0.64523	25.742	3.79
10474064	*Trp53i11*	0.00469	0.64523	23.892	0.01
10538126	*Gimap4*	0.00469	0.64523	24.783	2.01
10476321	*Prnd*	0.0047	0.64523	23.064	4.45
10447510	*Amd1*	0.00472	0.64523	23.892	15.9
10554808	*Fzd4*	0.00475	0.64523	23.064	13.44
10400293	*6530401N04Rik*	0.00479	0.64523	23.892	8.39
10508490	*Srsf5*	0.0048	0.64523	23.892	5.88
10534343	*Eln*	0.00486	0.64523	23.892	22.93

10597900	*Zfp445*	0.00489	0.64523	22.291	14.53
10368888	*Foxo3*	0.00491	0.64523	23.892	16.02
10489266	*Chd6*	0.00491	0.64523	24.783	15.62
10502081	*Enpep*	0.00493	0.64523	23.892	2.95
10430997	*Pacsin2*	0.00496	0.64523	23.064	1.84
10405179	*S1pr3*	0.00497	0.64523	23.892	23.64
10348194	*Efhd1*	0.0051	0.64523	24.783	2.01
10538377	*Wipf3*	0.00512	0.64523	23.892	12.58
10417920	*Usp54*	0.00515	0.64523	22.291	10.85
10422396	*Stk24*	0.00516	0.64523	23.064	1.33
10375503	*Trim7*	0.00518	0.64523	23.064	9.82
10561104	*Axl*	0.00519	0.64523	23.892	15.7
10358091	*Nav1*	0.00529	0.654329	24.783	1.25
10373452	*Gm129*	0.00542	0.655677	23.064	9.9
10428857	*Mtss1*	0.00549	0.658444	24.783	3.28
10526363	*Por*	0.00553	0.659814	23.064	12.05
10542575	*Pde3a*	0.00556	0.661175	23.892	3.05
10469581	*Etl4*	0.00559	0.662527	23.892	23.05
10495685	*Arhgap29*	0.00561	0.662688	23.892	1.16
10509947	*Arhgef19*	0.00563	0.662849	23.064	21.9
10531633	*Hnrnpd*	0.00568	0.66348	23.892	8.73
10518726	*Slc25a33*	0.0057	0.66348	23.064	10.21
10399234	*Efr3b*	0.00577	0.668267	23.892	1.64
10444890	*Ier3*	0.00585	0.672497	23.892	15.46
10570068	*Col4a2*	0.00587	0.672497	23.064	2.25
10500272	*Gm129*	0.00591	0.672497	23.064	9.89
10505779	*Acer2*	0.00608	0.682849	23.892	11.73
10547100	*Plxnd1*	0.00624	0.682849	22.291	22.84
10491915	*Ccrn4l*	0.00626	0.682849	25.742	12.18
10348493	*Lrrfip1*	0.00626	0.682849	23.064	1.18
10440186	*Crybg3*	0.0063	0.682935	23.064	3.75
10381311	*Wnk4*	0.00638	0.683359	23.064	10.78
10607250	*Apex2*	0.00642	0.683442	23.064	7.73
10396926	*Srsf5*	0.00649	0.684821	23.892	5.86
10607116	*Ammecr1*	0.00656	0.686022	22.291	16.08
10357878	*Adora1*	0.00659	0.687138	25.742	9.93
10366814	*Cdk4*	0.00679	0.692812	23.064	8.72
10483817	*6030499A19Rik*	0.00687	0.692812	24.783	12.68
10399680	*Cys1*	0.00687	0.692812	23.892	14.15
10348580	*Klhl30*	0.00692	0.692812	23.064	22.14
10363696	*D630028G08Rik*	0.00697	0.692812	23.892	6.79
10489878	*Ptgis*	0.00697	0.692812	23.892	11
10462091	*Klf9*	0.00699	0.692812	23.064	10.52
10502655	*Cyr61*	0.00699	0.692812	24.783	23.92
10474642	*BC052040*	0.00699	0.692812	24.783	4.2
10364712	*Cirbp*	0.00705	0.692812	23.064	8.97
10584674	*Mcam*	0.00706	0.692812	23.064	1.17
10418921	*Sncg*	0.00715	0.692812	22.291	10.19
10344981	*Pi15*	0.00717	0.692812	23.892	2.06

10354085	*Rev1*	0.00719	0.692812	23.892	16.22
10357965	*Lgr6*	0.00725	0.696705	23.892	7.05
10555378	*Ucp3*	0.00742	0.702743	22.291	7.93
10580370	*Dnaja2*	0.00743	0.702743	22.291	11.8
10539907	*Tpra1*	0.00747	0.702743	23.892	0.8
10489377	*Serinc3*	0.00748	0.702743	23.064	15.63
10491753	*Intu*	0.00754	0.702743	22.291	10.37
10462442	*Il33*	0.00763	0.702851	23.064	15.4
10364361	*Icosl*	0.00774	0.703864	23.064	14.39
10501734	*Palmd*	0.00774	0.703864	24.783	22.52
10462039	*Trpm3*	0.00775	0.703864	23.892	4.9
10579799	*Tmem184c*	0.00778	0.703882	22.291	10.29
10503218	*Chd7*	0.00792	0.70855	22.291	22.01
10484201	*Ccdc141*	0.008	0.70855	23.064	10.18
10437639	*Emp2*	0.00804	0.70855	23.064	10.11
10360684	*Ephx1*	0.00806	0.70855	23.892	11.29
10405587	*Tgfbi*	0.00807	0.70855	24.783	16.01
10484197	*Ccdc141*	0.0081	0.70855	22.291	10.07
10416406	*Htr2a*	0.00813	0.70855	23.064	9.56
10547009	*Vgll4*	0.00832	0.709415	23.892	22.85
10540472	*Bhlhe40*	0.00856	0.721231	22.291	9.34
10428809	*Klhl38*	0.00873	0.724319	22.291	9.85
10410501	*Irx1*	0.00877	0.724319	23.892	15.6
10425016	*Commd5*	0.00878	0.724319	23.064	9.84
10395103	*Pxdn*	0.00881	0.725112	23.064	0.68
10413932	*E130203B14Rik*	0.00889	0.72992	22.291	23.32
10588495	*Dusp7*	0.00893	0.72992	23.892	12.14
10449452	*Fkbp5*	0.00907	0.736228	23.064	15.75
10352936	*Sox17*	0.00909	0.736228	22.291	11.21
10574404	*Setd6*	0.00929	0.745633	25.742	10.68
10394674	*Socs2*	0.00935	0.746198	23.064	10.64
10368886	*Foxo3*	0.00936	0.746198	23.064	16.15
10506031	*Nfia*	0.00957	0.750127	23.892	8.38
10461369	*Ahnak*	0.00964	0.750127	23.892	15.82
10466304	*Dtx4*	0.00966	0.750127	22.291	22.11
10554156	*Fam174b*	0.00967	0.750127	24.783	7.87
10368175	*Pde7b*	0.00969	0.750127	22.291	10.44
10533026	*Prkab1*	0.00986	0.750127	23.064	5.32
10460221	*Chka*	0.00986	0.750127	23.892	17.15
10397249	*Lin52*	0.00987	0.750127	23.064	10.84
10346668	*Fam117b*	0.00995	0.750127	23.064	10.48
10357103	*Cdh19*	0.01	0.750127	22.291	15.75
10586368	*Clpx*	0.01	0.750127	23.892	4.65
10583586	*Slc44a2*	0.0101	0.750872	23.892	11
10398211	*Hhipl1*	0.0102	0.750872	23.064	10.28
10601350	*Fgf16*	0.0102	0.750872	23.892	6.17
10520638	*0610007C21Rik*	0.0102	0.750872	23.064	9.76
10357363	*Nckap5*	0.0102	0.750872	25.742	3.99
10395466	*Dock4*	0.0103	0.756667	23.892	1.66

10396161	*Tmx1*	0.0105	0.760363	23.064	10.17
10582664	*2310022B05Rik*	0.0105	0.760363	23.064	3.32
10424779	*Cks2*	0.0105	0.760363	24.783	1.34
10518909	*Rnf207*	0.0105	0.760363	24.783	5.64
10417561	*Fam107a*	0.0106	0.763429	23.064	13.5
10384572	*1110067D22Rik*	0.0107	0.763429	22.291	12.46
10455961	*Iigp1*	0.0107	0.763429	23.064	9.78
10530100	*Arap2*	0.0108	0.763429	24.783	1.48
10497862	*Trpc3*	0.0108	0.763429	25.742	1.5
10538273	*BC022713*	0.0109	0.767446	22.291	5.33
10357676	*Cdk18*	0.011	0.771432	22.291	11.8
10500434	*Bcl9*	0.0112	0.771615	25.742	8.76
10544002	*Creb3l2*	0.0112	0.771615	23.064	23.24
10433057	*Calcoco1*	0.0112	0.771615	23.892	10.91
10399973	*Hdac9*	0.0113	0.771615	23.892	22.31
10493585	*Ube2q1*	0.0114	0.771615	23.064	9.44
10360589	*Ahctf1*	0.0116	0.771615	24.783	16.07
10401128	*Max*	0.0116	0.771615	22.291	14.37
10437687	*Litaf*	0.0117	0.771615	23.892	22.81
10423963	*Eny2*	0.0117	0.771615	23.064	9.44
10455148	*Pcdhga12*	0.0118	0.771615	23.892	2.63
10468746	*Hspa12a*	0.0118	0.771615	23.064	22.49
10509463	*Eif4g3*	0.0119	0.771615	23.064	0.03
10526943	*Gpr146*	0.0119	0.771615	23.064	9.91
10574471	*Cmtm3*	0.012	0.771615	23.064	3.23
10596383	*Col6a6*	0.0121	0.771615	25.742	8.41
10551169	*B3gnt8*	0.0123	0.771615	23.064	11.76
10584142	*Ets1*	0.0124	0.771615	23.064	22.32
10523772	*Lrrc8d*	0.0124	0.771615	23.064	2.15
10462237	*Smarca2*	0.0124	0.771615	23.064	16.26
10593499	*AI593442*	0.0124	0.771615	24.783	5.55
10359917	*Hsd17b7*	0.0125	0.771615	23.064	8.58
10512145	*Aqp7*	0.0125	0.771615	24.783	3.48
10544525	*Pdia4*	0.0126	0.772423	23.064	23.33
10372716	*Rap1b*	0.0126	0.772423	23.064	7.29
10500183	*Adamtsl4*	0.0131	0.790804	24.783	10.25
10381807	*Lin52*	0.0131	0.790804	23.064	11.25
10427310	*Hnrnpa1*	0.0131	0.790804	24.783	9.34
10408850	*Nedd9*	0.0133	0.79674	23.064	22.55
10441038	*Hlcs*	0.0134	0.79674	23.892	8.96
10416940	*Tpm3*	0.0134	0.79674	23.892	5.16
10594110	*Neo1*	0.0134	0.79674	22.291	3.55
10596222	*Ccrl1*	0.0135	0.797143	22.291	13.92
10350473	*B3galt2*	0.0135	0.797143	25.742	3.52
10472820	*Itga6*	0.0137	0.797143	24.783	3.18
10399208	*Tmem196*	0.0137	0.797143	24.783	5.8
10406614	*Mtx3*	0.0138	0.797143	25.742	0.24
10556598	*Xylt1*	0.0138	0.797143	23.892	6.02
10478415	*Wisp2*	0.0139	0.797143	23.892	10.31

10376950	*Pmp22*	0.014	0.800151	23.064	11.4
10387588	*Polr2a*	0.014	0.800151	23.064	7.44
10546631	*Frmd4b*	0.0142	0.800151	23.064	10.04
10382692	*2210020M01Rik*	0.0143	0.801973	24.783	13.85
10355278	*Erbb4*	0.0145	0.807841	23.892	10.44
10438603	*Igf2bp2*	0.0145	0.807841	23.064	22.28
10532085	*Tgfbr3*	0.0146	0.807841	23.892	15.03
10355050	*Raph1*	0.0146	0.807841	24.783	15.84
10546450	*Adamts9*	0.0147	0.807841	22.291	0.51
10565609	*Thrsp*	0.0148	0.808339	25.742	13.2
10545720	*Stambp*	0.0148	0.808339	23.064	9.21
10506488	*Ppap2b*	0.0149	0.810547	23.892	0.3
10471721	*Ptgs1*	0.0152	0.811488	23.064	14.65
10424909	*Hsf1*	0.0152	0.811488	23.064	21.61
10380087	*Mtmr4*	0.0154	0.814825	24.783	21.75
10577782	*Htra4*	0.0154	0.814825	23.064	13.2
10395163	*Lamb1*	0.0157	0.820925	22.291	21.95
10408450	*Sox4*	0.0158	0.823731	23.064	22.93
10478890	*Cebpb*	0.0159	0.825314	24.783	13.04
10371176	*Nfic*	0.016	0.825684	24.783	13.35
10356936	*Ppip5k2*	0.016	0.825684	23.892	14.24
10597279	*Ccrl2*	0.0161	0.827242	23.892	10.27
10518069	*Efhd2*	0.0164	0.832162	23.064	11.53
10498383	*Igsf10*	0.0164	0.832162	23.064	2.5
10424607	*Ptp4a3*	0.0164	0.832162	25.742	15.11
10358615	*Hmcn1*	0.0165	0.832162	22.291	21.98
10479221	*Gm14403*	0.0166	0.833658	23.064	15.98
10392056	*Cyb561*	0.0167	0.834578	25.742	11.03
10546834	*Rad18*	0.0168	0.834578	24.783	6.41
10597969	*Gm10052*	0.0169	0.834578	24.783	9.76
10454411	*Hnrnpa1*	0.0169	0.834578	24.783	9.51
10557843	*Fus*	0.0169	0.834578	23.892	10.09
10362245	*Epb4.1l2*	0.017	0.835175	23.064	16.02
10494122	*Rfx5*	0.017	0.835175	25.742	3.4
10555662	*Art1*	0.0171	0.835175	23.064	9.9
10590808	*Yap1*	0.0171	0.835175	23.064	21.96
10538842	*Gng12*	0.0172	0.835469	23.892	6.4
10565958	*P2ry6*	0.0172	0.835469	23.892	10.25
10501629	*Cdc14a*	0.0176	0.846801	22.291	10.09
10412826	*Gm10403*	0.0178	0.852208	23.064	9.99
10351905	*Spna1*	0.0179	0.852208	23.892	5.02
10534667	*Serpine1*	0.018	0.852208	22.291	10.02
10346943	*Creb1*	0.0182	0.854847	24.783	21.19
10392522	*Abca8a*	0.0183	0.857279	22.291	16.03
10546430	*Adamts9*	0.0183	0.857279	23.892	0.08
10427095	*Tenc1*	0.0184	0.859698	23.064	9.9
10575685	*Nudt7*	0.0188	0.864751	23.064	10.03
10380419	*Col1a1*	0.019	0.86722	22.291	5.78
10355343	*Abca12*	0.0191	0.868439	24.783	4.89

10436608	*Cxadr*	0.0193	0.869435	22.291	21.7
10500847	*Magi3*	0.0193	0.869435	22.291	3.79
10379262	*Nf1*	0.0194	0.869435	23.064	23.07
10433735	*Abcc1*	0.0194	0.869435	23.064	11.98
10558090	*Tacc2*	0.0197	0.869435	22.291	16.44
10468329	*Obfc1*	0.0197	0.869435	22.291	14.5
10352735	*Ints7*	0.0198	0.869435	23.892	23.52
10527732	*Fry*	0.0199	0.869435	23.892	15.77
10446763	*Lbh*	0.0199	0.869435	23.064	7.71
10399892	*Gpr22*	0.02	0.869435	25.742	12.33
10424370	*Trib1*	0.02	0.869435	24.783	10.43
10358583	*Hmcn1*	0.0201	0.869435	22.291	22.22
10494445	*Lix1l*	0.0201	0.869435	22.291	8.74
10416800	*Lmo7*	0.0201	0.869435	23.892	9.77
10493335	*5830417I10Rik*	0.0201	0.869435	23.064	0.45
10511580	*Pdp1*	0.0202	0.870583	23.892	9.08
10517328	*Tmem50a*	0.0203	0.871888	22.291	10.26
10390022	*Rsad1*	0.0205	0.871888	24.783	10.06
10491920	*Elf2*	0.0205	0.871888	24.783	10.93
10530441	*Corin*	0.0205	0.871888	23.892	16.18
10575363	*Zfp612*	0.0205	0.871888	23.064	10.16
10445826	*Mocs1*	0.0206	0.871969	22.291	10.7
10567043	*Rras2*	0.0208	0.875225	23.064	2.18
10555389	*Ucp2*	0.0208	0.875225	24.783	10.43
10400143	*Stxbp6*	0.021	0.878442	24.783	15.67
10363070	*Gp49a*	0.0213	0.881656	23.064	5.97
10365344	*Tcp11l2*	0.0216	0.8889	23.064	3.74
10587829	*Plod2*	0.0216	0.8889	22.291	10.34
10475544	*Sema6d*	0.0219	0.891955	23.892	0.78
10433639	*Mkl2*	0.0219	0.891955	22.291	15.08
10433472	*1810013L24Rik*	0.0221	0.897018	22.291	12
10346365	*Sgol2*	0.0223	0.898799	22.291	7.73
10546432	*Adamts9*	0.0223	0.898799	23.064	23.06
10399897	*Hbp1*	0.0224	0.898799	22.291	7.54
10447773	*Slc22a3*	0.0224	0.898799	24.783	3.6
10392221	*Pecam1*	0.0224	0.898799	25.742	21.92
10488655	*Bcl2l1*	0.0225	0.898799	23.064	13.95
10505982	*Fggy*	0.0226	0.898799	23.892	4.63
10546434	*Adamts9*	0.0226	0.898799	22.291	23.02
10596072	*Ppp2r3a*	0.0226	0.898799	23.064	11.01
10367641	*Mthfd1l*	0.0226	0.898799	24.783	9.07
10457686	*Dsc2*	0.0226	0.898799	23.064	3.86
10597743	*Cx3cr1*	0.0228	0.899752	25.742	2.5
10470248	*Gpsm1*	0.0228	0.899752	25.742	15.07
10463911	*Add3*	0.0231	0.904563	23.064	23.35
10457644	*Cdh2*	0.0231	0.904563	23.064	10.31
10594988	*Mapk6*	0.0231	0.904563	23.064	22.46
10347748	*Acsl3*	0.0234	0.910296	23.064	22.08
10354704	*Sf3b1*	0.0236	0.913081	22.291	11.35

10472277	*Mer17*	0.0236	0.913081	22.291	8.97
10358982	*Mr1*	0.0237	0.913858	23.064	11.72
10353899	*Sema4c*	0.0239	0.914735	23.064	22.14
10474229	*Cd59a*	0.024	0.916589	22.291	12.07
10393058	*H3f3b*	0.0241	0.917209	25.742	4.48
10487252	*Gabpb1*	0.0243	0.917209	23.892	2.79
10540273	*Ube2v2*	0.0243	0.917209	25.742	1.33
10366541	*Lrrc10*	0.0243	0.917209	23.892	11.61
10453918	*3110002H16Rik*	0.0243	0.917209	23.892	16.02
10486029	*Atpbd4*	0.0245	0.922514	23.892	5
10358658	*Hmcn1*	0.0246	0.922514	25.742	21.96
10467013	*Prkg1*	0.0248	0.923867	23.892	10.73
10581395	*Slc12a4*	0.0253	0.928973	24.783	15.7
10446376	*Man2a1*	0.0256	0.929759	23.892	15.42
10586781	*Myo1e*	0.0256	0.929759	23.064	1.53
10416099	*Adra1a*	0.0257	0.931487	23.892	10.22
10569972	*Lass4*	0.0257	0.931487	23.064	5.74
10501778	*Ptbp2*	0.0258	0.932085	25.742	3.28
10469066	*Ccdc3*	0.0258	0.932085	23.064	9.94
10352838	*Lamb3*	0.0259	0.932085	23.892	9.4
10400762	*Map4k5*	0.0261	0.935496	25.742	3.12
10506201	*Ror1*	0.0262	0.936805	24.783	22.23
10565962	*P2ry2*	0.0262	0.936805	23.892	11.09
10407042	*Dimt1*	0.0263	0.936805	25.742	5.25
10373325	*Gpr182*	0.0265	0.93848	23.892	10.34
10356520	*Col6a3*	0.0266	0.939215	22.291	2.45
10537298	*Chrm2*	0.0269	0.941394	24.783	10.2
10358539	*Hmcn1*	0.0272	0.94816	23.892	22.22
10368675	*Marcks*	0.0272	0.94816	24.783	0.83
10518341	*Ppp2r5a*	0.0274	0.950473	24.783	23.01
10511665	*Necab1*	0.0274	0.950473	25.742	4.44
10500808	*Olfml3*	0.0276	0.950677	22.291	15.36
10408329	*Gmnn*	0.0277	0.950677	23.892	10.28
10349404	*Mgat5*	0.028	0.952697	25.742	21.89
10502050	*Alpk1*	0.028	0.952697	22.291	9.76
10453512	*Kpna2*	0.0283	0.957407	22.291	10.74
10587085	*BC031353*	0.0284	0.958965	23.064	9.9
10423109	*Adamts12*	0.0289	0.963044	23.892	1
10394735	*Pdia6*	0.0296	0.971493	23.064	21.95
10379389	*Adap2*	0.0297	0.971493	22.291	10.49
10428018	*Ube2v2*	0.0297	0.971493	25.742	1.2
10455514	*Kcnn2*	0.0297	0.971493	23.892	7.66
10352777	*Slc30a1*	0.0298	0.972081	23.064	10.1
10518833	*Camta1*	0.0301	0.974093	23.892	8.34
10598178	*Disp1*	0.0302	0.974093	24.783	2.28
10427796	*Npr3*	0.0302	0.974093	23.892	16.16
10557009	*Eef2k*	0.0303	0.974093	24.783	14.98
10376998	*Pirt*	0.0304	0.974664	24.783	10.5
10365729	*Cdk17*	0.0307	0.976918	25.742	1.63

10525989	*Gpr133*	0.0307	0.976918	23.892	3.67
10455299	*Sh3rf2*	0.0308	0.976918	23.064	11.6
10445268	*Gpr116*	0.0308	0.976918	24.783	15.86
10540275	*Gxylt2*	0.0309	0.978027	23.892	10.12
10553080	*Car11*	0.0309	0.978027	23.892	12.74
10417912	*Usp54*	0.0311	0.979443	22.291	10.28
10540122	*Slc6a6*	0.0313	0.981607	22.291	15.11
10448202	*Tpm4*	0.0313	0.981607	24.783	2.9
10604735	*Rbmx*	0.0314	0.981607	24.783	9.02
10379524	*Ccl11*	0.0314	0.981607	24.783	9.57
10459183	*Slc26a2*	0.0315	0.981607	23.064	8.35
10544640	*Tra2a*	0.0317	0.984389	23.892	14.08
10537184	*Cald1*	0.0321	0.987325	23.064	1.68
10597758	*Csrnp1*	0.0324	0.991407	23.064	10.85
10484207	*Ccdc141*	0.0325	0.992402	23.892	10.08
10433782	*Efcab1*	0.0326	0.992402	25.742	3.38
10389300	*Dhrs11*	0.0326	0.992402	25.742	15.49
10595496	*Snap91*	0.0331	0.993168	23.064	8.38
10426891	*Mettl7a1*	0.0334	0.99712	23.892	16.09
10595094	*2310046A06Rik*	0.0334	0.99712	22.291	10.12
10382284	*Prkar1a*	0.0336	1	23.892	1.8
10551770	*Zfp30*	0.0337	1	23.892	16.2
10368630	*Rwdd1*	0.0337	1	24.783	11.06
10358621	*Hmcn1*	0.0337	1	23.892	22.74
10497503	*Kpna2*	0.0339	1	22.291	10.81
10359235	*Rasal2*	0.034	1	23.064	16.16
10602180	*Tmem164*	0.0341	1	23.064	9.47
10370999	*Lingo3*	0.0344	1	22.291	2.87
10414288	*Psmc6*	0.0344	1	23.064	10.07
10462313	*Slc1a1*	0.0344	1	24.783	3.59
10530666	*Lnx1*	0.0345	1	22.291	10.2
10578138	*Dctn6*	0.0345	1	24.783	10.7
10507594	*Slc2a1*	0.0347	1	23.892	10.64
10476834	*Xrn2*	0.0347	1	23.892	5.23
10485624	*Prrg4*	0.0348	1	22.291	9
10443131	*Itpr3*	0.0349	1	23.892	10.9
10580986	*Rbmxrt*	0.035	1	24.783	9.59
10484203	*Ccdc141*	0.0351	1	23.064	10.28
10412011	*Kif2a*	0.0352	1	25.742	2.07
10349733	*Nucks1*	0.0353	1	22.291	3.9
10400089	*Gm889*	0.0355	1	23.892	2.88
10531919	*Hsd17b11*	0.0358	1	22.291	10.07
10587880	*Pcolce2*	0.0359	1	24.783	16.07
10574350	*Mmp15*	0.0361	1	22.291	21.85
10513141	*Ptpn3*	0.0361	1	23.064	16.13
10583732	*Ldlr*	0.0362	1	23.064	22.42
10582322	*Snai3*	0.0365	1	23.892	10.42
10430770	*Tob2*	0.0365	1	23.892	10.19
10372069	*Socs2*	0.0366	1	23.892	11.49

10382425	*Gprc5c*	0.0367	1	25.742	3.86
10569927	*Map2k7*	0.0368	1	23.064	8.69
10531645	*Hnrpdl*	0.0369	1	23.892	10.18
10459510	*Mir694*	0.037	1	25.742	10.69
10510270	*Mthfr*	0.0373	1	22.291	21.59
10425695	*Srebf2*	0.0374	1	23.064	23.35
10584124	*Arhgap32*	0.038	1	22.291	7.46
10585703	*Rpp25*	0.0384	1	22.291	11.15
10506254	*Raver2*	0.0386	1	23.064	23.37
10350123	*Lmod1*	0.039	1	23.892	13.44
10443764	*Slc37a1*	0.0394	1	22.291	10.28
10368782	*Fig4*	0.0398	1	23.064	12.39
10570236	*Mcf2l*	0.0398	1	23.892	13.44
10440406	*Nrip1*	0.04	1	24.783	8.92
10428998	*Asap1*	0.0401	1	22.291	11.73
10354418	*Obfc2a*	0.0404	1	22.291	9.17
10564290	*Klf13*	0.0404	1	23.064	12.65
10358726	*Tsen15*	0.0405	1	25.742	3.06
10392284	*Kpna2*	0.0405	1	22.291	10.67
10569848	*Stxbp2*	0.0406	1	24.783	7.99
10455108	*Pcdhb16*	0.0407	1	23.064	3.07
10504902	*Murc*	0.0407	1	24.783	2.69
10593671	*Dmxl2*	0.0408	1	25.742	2.89
10414256	*Rbmxrt*	0.0409	1	23.892	10.15
10388185	*Smtnl2*	0.0409	1	23.892	15.63
10510162	*Ppp2r5a*	0.0409	1	23.064	22.65
10384349	*Polr2c*	0.041	1	24.783	11.11
10522335	*Atp10d*	0.0415	1	23.892	14.88
10350977	*4930523C07Rik*	0.0418	1	23.064	2.54
10440534	*Adamts5*	0.0418	1	25.742	3.54
10433433	*BC024814*	0.0419	1	23.064	9.45
10347460	*Ttll4*	0.042	1	25.742	21.57
10394731	*Rock2*	0.0421	1	23.892	10.27
10489660	*Elmo2*	0.0426	1	23.892	23.07
10404152	*Fam65b*	0.0429	1	25.742	3.62
10407420	*Net1*	0.0434	1	22.291	12.1
10362446	*Trdn*	0.0434	1	23.892	10.65
10491091	*Tnfsf10*	0.0442	1	23.892	9.98
10516605	*Hdac1*	0.0443	1	23.892	10.26
10356312	*Ncl*	0.0443	1	25.742	10.03
10447004	*Hdac1*	0.0444	1	23.064	10.16
10425257	*Polr2f*	0.0444	1	25.742	10.16
10500896	*H3f3b*	0.0444	1	25.742	3.04
10501608	*Vcam1*	0.0445	1	23.064	15.73
10554150	*Rgma*	0.0446	1	23.892	9.79
10604375	*Apln*	0.0448	1	22.291	2.56
10361156	*Rcor3*	0.0448	1	23.064	7.26
10415021	*Abhd4*	0.0449	1	23.064	10.21
10392735	*Cdc42ep4*	0.0454	1	23.892	11.27

10498168	*Exosc8*	0.0454	1	23.064	6.77
10552622	*2410002F23Rik*	0.0455	1	23.064	7.1
10487476	*1500011K16Rik*	0.0456	1	25.742	15.26
10536949	*Fam40b*	0.0456	1	23.064	11.52
10358432	*Zfp825*	0.0458	1	24.783	6.73
10349711	*Slc41a1*	0.0459	1	24.783	11.2
10461594	*Ms4a4c*	0.0459	1	23.064	6.1
10502748	*Lphn2*	0.046	1	22.291	3.84
10471953	*Acvr2a*	0.0461	1	25.742	21.46
10571162	*Eif4ebp1*	0.0465	1	23.892	13.99
10384797	*Ccdc85a*	0.0465	1	22.291	14.96
10439798	*Dzip3*	0.0469	1	22.291	8.04
10423049	*Prlr*	0.0471	1	22.291	7.75
10582152	*4632415K11Rik*	0.0471	1	24.783	10.49
10543017	*Pdk4*	0.0473	1	23.892	3.76
10595109	*Lrrc1*	0.0474	1	24.783	3.48
10402140	*Gpr68*	0.0478	1	25.742	13.83
10606016	*Il2rg*	0.0483	1	23.892	1.57
10422013	*Klf12*	0.049	1	23.064	15.52
10520521	*Cenpa*	0.049	1	24.783	4.7
10441497	*Tulp4*	0.0491	1	23.892	15.78
10435075	*Tfrc*	0.0495	1	22.291	21.71
10358537	*Hmcn1*	0.0497	1	23.064	22.15

Supplementaly Table 2: Genes bound CLOCK to -5000bp upstream of each genes by Chip-seq analysis

CLOCK:GSE117488	
**Symbol**	**MACS2 value**
Nr1d2	2666
Gpi1	2428
Ncl	2413
Klf16	2400
Nup50	2358
Lcorl	2316
Slc27a2	2265
Jade1	2253
Coq10b	2234
Gmfb	2182
Per3	2152
Adh5	2121
Hmgn2	2105
Ccno	2077
Mthfd1l	2070
Igsf8	2067
Nptn	2057
Gnpnat1	2022
Abcf2	2011
Fbxl22	2010
Aven	1988
Tulp4	1984
Sipa1l2	1983
Gys2	1983
Serpina16	1975
Rapgef1	1954
1810013L24Ri	1949
Avpi1	1929
Tef	1924
Mapk6	1887
Rint1	1863
Pus7	1863
Per2	1845
Smim17	1837
Cry1	1821
Bnip3	1821
Upp2	1806
Ciart	1798
Fbxo30	1789
Ankrd12	1777
Serf2	1774
Tspan4	1768
Polr2l	1768
Ntpcr	1765
Mapk8	1756
Tbcel	1755
Fibp	1754
Ccdc85b	1754
Ube2q1	1745
Taf1c	1738
Zfp526	1737
Dedd2	1737
Zfp292	1718

Bhlhe40	1712
Nfix	1702
Cldn34d	1694
Rnf145	1681
Sumo2	1669
Lamp1	1632
Rorc	1628
Car5a	1614
Agmat	1606
Sde2	1605
Ubald1	1597
Foxo3	1585
Junb	1582
Ilf3	1578
Tns2	1573
Atxn3	1572
Cnn3	1570
Ppp1r3c	1563
Nfe2	1561
Kdm6b	1560
Eef2k	1544
Ppia	1535
Phospho1	1531
Arl4a	1506
Dbp	1496
Supv3l1	1490
Ddx50	1489
Mex3d	1483
Nr1d1	1459
Fnip1	1458
Mknk2	1456
Bhlhe41	1455
Klf15	1453
Sertad2	1449
Bri3	1446
Hif1a	1443
Etnk2	1442
Slc35a5	1428
Atg3	1428
Gpr146	1426
Sertad1	1426
Gjb2	1408
St6gal1	1403
Inmt	1402
Car11	1401
Gstt2	1398
E030018B13Ri	1395
Zswim7	1385
Ttc19	1385
Ipo5	1384
Per1	1383
Eva1a	1377
Gga2	1377
Smim14	1375
Acsl1	1375
Sox12	1371
Phf23	1370
Dvl2	1370

Eif3a	1370
Rpp38	1368
Acbd7	1368
Herpud1	1362
Gnat1	1361
Ccne2	1356
Hlf	1351
Ccdc88c	1350
Pms2	1347
Aimp2	1347
Brox	1346
Aida	1346
Albfm1	1342
Map7	1340
Luc7l2	1335
Pabpc4	1334
Lrp4	1333
Slc41a1	1328
Cyp17a1	1315
Pcbp1	1313
Acy3	1311
Selenbp1	1305
Dlgap4	1304
Bcat2	1302
Nt5c2	1302
Gpatch3	1301
Osbpl2	1299
Mllt1	1298
Trim37	1297
Ski	1294
Casc3	1294
Lrrc63	1286
Lcp1	1286
Kmt2c	1284
Cdyl	1280
Cry2	1277
Ttc39b	1273
Fam76a	1269
Klf11	1267
Rbm4b	1265
Ptma	1252
Zfp91	1247
Lpxn	1247
Rtkn	1242
Arhgap42	1236
Ptp4a2	1233
Epha1	1231
Serpina6	1231
Cp	1231
Fam43a	1225
Hes1	1222
Golga5	1219
Pard6b	1215
Phf1	1211
Zbtb7b	1210
Napa	1207
Vps13a	1204
Repin1	1199

Gm5111	1199
Tha1	1199
Txndc16	1196
Gpr137c	1196
Zcwpw1	1196
Mepce	1196
Gas2	1193
Fancf	1193
Serpine1	1191
Cdk5r1	1182
Rnf43	1176
Ino80	1173
Gm3448	1171
Gm3417	1171
Tcte3	1171
Ermard	1171
Mphosph9	1166
2810006K23Ri	1166
Urad	1165
Susd6	1165
Pgm2l1	1161
Trpm7	1160
Atl3	1156
Ado	1155
L3hypdh	1152
Jkamp	1152
Cfh	1152
Mar8	1149
Sorbs3	1148
Slc20a1	1147
Adcy10	1146
Mlxipl	1142
Meig1	1138
Dclre1c	1138
Gm3435	1126
Utp11	1121
Mrpl48	1118
Cyp2a5	1117
Marveld1	1107
Nup155	1104
Mar9	1103
Cdk4	1103
Ggnbp2	1103
Unc93a	1103
Pipox	1099
Unc93a2	1098
Lrp1	1097
Trim11	1092
Nampt	1092
Eps15l1	1090
Pbld2	1090
Hnrnph3	1090
Plbd2	1089
Ppp1r3e	1085
Pim3	1085
Mfsd3	1082
Gpt	1082
Dgat2	1080

Qk	1080
Ngef	1078
Neu2	1078
Zbtb10	1077
Pigw	1074
Myo19	1074
2310011J03Ri	1071
Gnb1	1070
Prrg4	1069
Gbe1	1068
Tet3	1064
Lefty1	1063
Ecsit	1061
Clock	1060
Rbbp6	1059
Tnfsf13	1057
Senp3	1057
Rsad1	1056
Taf8	1054
Ccnd3	1054
Abcf1	1053
Tmem11	1053
Rps6ka4	1052
Usp2	1051
Ttc7	1049
Xrcc1	1049
Arrb2	1048
9030025P20Ri	1046
Pten	1044
Cln6	1043
Lekr1	1041
Pip4p1	1039
Atp2b1	1039
Pla2g6	1035
Rasl11b	1035
Ifrd2	1034
Slc26a2	1033
Ldlrad3	1033
Mcoln1	1030
Hdac11	1029
Cops7a	1028
Pdcd4	1027
Cep295	1022
Gpr84	1022
Por	1019
Nutf2	1019
Lsm12	1019
G6pc3	1019
Slc30a1	1016
Mgll	1016
Adora1	1016
Sesn1	1010
Cep57l1	1010
Pdxk	1009
Trappc10	1008
Prdx6	1004
Sirt1	999
Ttc21a	997

Gorasp1	997
Slc17a8	996
Ercc6	994
Usp36	993
Bahcc1	993
Ube2g1	992
Gpd1l	992
Dmxl2	991
Zzz3	987
Uba7	987
Lss	986
Atp6v1h	983
Tada2a	981
Lrp3	981
Mcfd2	979
Itsn1	979
Cryzl1	979
Sec24a	978
Bod1l	977
Anxa7	976
Pdcd11	975
Atp5md	975
Fbxo3	974
4931422A03Ri	974
Ccdc141	974
Nup210	974
Insig1	973
AW549877	971
Ces1e	971
Srd5a2	969
Dnajc16	968
Casp9	968
Eif5a2	966
Dusp12	966
Prkab1	966
Dis3l2	965
Vps26a	965
Brpf3	962
Rev1	958
Ppp1r15b	957
Prss8	956
Rnf13	954
Stk35	953
Rnaseh2b	952
Gpt2	947
Setd4	947
Cbr1	947
Odc1	946
Akr1d1	946
Rhebl1	946
Dusp7	941
Mknk1	939
Zfp36l2	937
Tbl1x	936
Cic	936
C1qbp	933
Fkbp4	933
Shmt1	932

Matr3	931
Ypel5	924
Dars	922
Ogfrl1	922
Igfbp4	921
Arsa	918
Hsdl1	917
Dnaaf1	917
Narf	915
Fam20b	915
Hacd1	914
Pnrc1	913
Cnnm4	913
Tmem185b	911
Atf3	910
Rbm38	909
Zfyve9	907
Rmnd1	906
Armt1	906
Xndc1	899
Rnf121	899
Bcl9l	899
Krr1	899
Susd1	898
Sox7	893
Tjp3	887
Pip5k1c	887
Ppm1a	884
Usp22	883
Ndufa6	882
Cyth1	878
Mfsd4b1	878
Fggy	878
Ddx39	877
Usp53	877
Reep6	876
Pcsk4	876
Kansl2	875
Ralb	873
2410002F23Ri	872
Znrf3	872
Clcn7	869
Slc6a6	868
Hbp1	868
Rrp9	866
Parp3	866
Echs1	864
Epop	864
Bax	864
Yif1b	861
Hmga1b	860
Hmga1	860
Trim8	859
Atp6v0c	858
Amdhd2	858
Ugt1a1	856
Reep3	855
Bop1	854

Spns2	852
Hnrnpa1	852
Cbx5	852
Cask	851
Tax1bp3	849
Emc6	849
Erich4	848
Dmac2	848
Adi1	846
Stag1	842
Arrdc1	840
Hnrnpf	833
Sgk2	833
Cblc	833
Phf7	828
Bap1	828
Slc7a2	828
Kdm2a	827
Ndufaf4	825
Syngap1	824
Cuta	824
Hdgf	824
Usp39	824
0610030E20Ri	824
Vdac1	823
Alpk1	823
Akap9	823
Fam50a	822
Fam92b	822
Edf1	821
Nup62	818
Il4i1b	818
Atf5	818
Bmp2k	817
Rab3gap2	816
Stbd1	816
Sephs2	815
Usp28	814
Xpnpep3	814
St13	814
Urb2	814
Taf5l	814
Rbp4	813
Tada1	812
Ccdc6	808
Aunip	807
Rybp	805
Hes7	805
Saa3	804
D10Wsu102e	803
Lonrf1	801
Hacd3	800
Gid4	799
Fam241a	798
Ppat	796
Paics	796
Snai3	795
Atf7	793

Pcx	792
Ankrd37	792
Srm	791
Slc38a4	791
Rpl14	789
Nfe2l2	788
Rrn3	785
Ywhag	785
Insig2	785
Zbtb14	785
Rcbtb1	784
Hexa	784
Proz	782
Jph1	781
Zbtb12	780
Ehmt2	780
Kng2	779
C4bp	776
Slc25a39	774
Smad2	774
Qdpr	772
Clrn2	772
Nr3c1	772
Hoga1	769
Wee1	768
Eif4g1	767
Lamtor4	767
Dnajb9	766
Pou2f1	765
Eif6	765
1700001J03Ri	764
Comt	764
Ctsf	764
Txnrd2	764
Wars2	763
P2ry2	762
Ntsr2	760
Slc6a9	758
Gpn3	758
Fam216a	758
Zmym6	757
Rassf6	757
Larp1	756
Rps9	756
Ccdc134	754
Tdrd3	754
Trim65	753
Jmy	752
Ttc32	750
Hjurp	749
A730008H23R	749
Lipe	749
Pdia3	748
Catsper2	748
Cpn1	748
Cecr2	747
Vkorc1	746
Prss53	746

Xpot	746
Cyp3a11	746
Lamc1	745
Snx8	744
Sel1l3	744
Cdca7	744
Ube2q2	743
Ifitm2	743
Ogdh	742
Prkaca	741
Syne3	741
Usp4	740
Hpn	739
Ubc	737
Phf20	736
Trib1	735
Kdm4c	735
Inhbe	734
Zkscan6	734
Arhgap12	734
Zc3h6	733
Mbnl1	733
Pnpla7	732
Mrpl41	732
Rasd2	731
Trip6	729
Tomm20	727
Tmem131l	727
Sbk1	726
Anks4b	724
Klf9	724
Prep	724
Rnf123	723
Mst1	723
Macrod1	722
Slc35a4	722
Apbb3	722
Tmc1	722
Lrba	722
Pfkl	721
Lman1	720
Ifrd1	719
Nr2f2	719
Dyrk1a	719
Chst12	717
Slc5a6	716
Atraid	716
Hcfc2	716
Tex14	714
Rad51c	714
Klf3	712
Camk2n1	712
Anks3	711
4930451G09Ri	711
Ran	711
Cnnm2	711
Memo1	711
Adipor2	710

Clmn	708
Txnl1	707
Mxd3	707
Crtc1	706
Pdk1	705
Lrrc8d	704
Efhd2	704
Gtf2f1	703
Ndufs6	703
Mrpl36	703
Met	703
Wdsub1	703
Dapk2	703
Rdh9	703
Polr3h	701
Tmem37	699
Gamt	697
Dazap1	697
Tmcc3	697
Zfp131	694
Cetn3	693
Siah2	692
Cyp2a4	691
Mfsd1	690
Crebrf	689
Gfod1	689
Map4k1	689
Eif3k	689
Smim10l1	688
Polr2h	687
Clcn2	687
Plcxd2	687
Dusp3	686
Cfap97d1	686
Rhobtb2	686
Prickle1	685
Dhrs7	684
Zeb1	681
Sik2	680
Elavl3	679
Snx33	679
Mnt	678
Bmp7	677
Rassf1	676
Scaf11	676
Rnf130	676
Enpp1	676
Irak1bp1	676
Rcc2	675
Utp15	673
Ankra2	673
Ubfd1	673
Ears2	673
Tor1aip2	671
Tor1aip1	671
Slc4a2	671
Clk4	670
BC049762	670

Ndrg1	667
Vars	666
D17H6S56E-5	666
Coq8a	666
Plag1	665
Chchd7	665
Zfp706	665
Ppm1l	664
Sdad1	664
Abca8b	663
Arrdc3	663
Map2k7	662
Tesmin	662
Slc25a37	662
Inpp5e	662
Usp49	662
Spag4	659
Nab2	657
Hsd11b1	657
Mthfr	656
Clcn6	656
Eci2	656
Cpeb1	656
Senp8	655
Myo9a	655
2310061I04Rik	655
Slc25a13	654
Pola2	654
Mcmdc2	653
Tmem175	652
Gak	652
Ptp4a1	652
Dbi	650
3110009E18Ri	650
Aph1c	649
Plcg1	649
Sh2b3	648
Zdhhc23	648
Paqr7	647
Thap4	646
Atg4b	646
Dph2	646
B4galt2	646
Atp6v0b	646
Acvr1b	646
Atmin	646
Rbm17	645
Pik3ap1	644
Fcgrt	643
Engase	641
Zswim6	641
Fam131c	641
Pfkfb3	640
1190007I07Rik	639
Pelo	638
Wdr4	637
Eid1	636
Srpk2	634

Fech	634
Esrra	633
Catsperz	633
Acsl5	633
Yy1	633
Parp1	632
Plekha8	632
Fkbp14	632
P4htm	632
Csrnp1	629
Leng9	629
Cobll1	629
Eif1ad	629
Banf1	629
Mesp2	628
Slc2a9	628
Frmd6	626
Zbtb25	626
Zbtb1	626
Kpna2	626
Knstrn	626
Hsd17b3	626
C4b	625
H2-DMa	624
Brd2	624
U2surp	624
Snx13	624
C1rl	622
Cfb	621
Tgif1	619
Atxn2	618
Aspg	617
Nrtn	617
Srebf1	614
Hyal1	613
Naa80	613
Zfp36	612
Aifm2	612
Slc38a9	612
Rdh1	612
Top1mt	611
Ubl4a	611
Slc10a3	611
Gm44504	611
A1cf	611
Txndc5	611
Tmem251	609
Moap1	609
4931406C07Ri	609
Fhit	608
Abcc2	607
Tbc1d10a	607
Stk40	607
Ldha	606
Lins1	605
Asb7	605
Hspa9	604
Nrep	604

Gtf3a	603
Fads2	603
Mrnip	603
Dctpp1	602
Adcy2	602
Hspe1	601
Hspd1	601
Ddx59	601
Pcyox1	600
Plin5	599
Ccdc9	599
Lrg1	599
E2f1	599
Srcap	598
Tcf3	598
Thrap3	597
Igfbp5	597
Egln2	595
Hook1	595
Rgl3	595
Zfhx2	593
Thtpa	593
Gpr68	593
Vgll4	593
Slc17a9	591
Nat10	590
Fetub	590
Etv5	589
Gna14	589
Foxred2	587
Hs6st1	587
Irs2	587
Ppara	587
Slc38a3	587
Ankrd9	586
Rnf19b	585
Rpia	585
Fubp1	584
Dnajb4	584
Il1rap	584
Ptpa	583
Crat	583
Hey1	583
Rps4x	583
Lactb2	582
Gatd3a	582
Arhgap35	582
Focad	582
Slc35g1	582
Gstm1	581
Ttc36	581
Actr8	580
Ctsd	579
Gm4969	579
Fbxo46	579
Stk17b	578
Stard4	577
Map2k6	577

Pqlc1	577
Nbeal2	577
Ccdc12	577
Ubtd1	577
Mms19	577
Txndc11	577
Mars2	576
Igfn1	576
Mpc1	575
Nmnat3	575
Sil1	575
Ttc25	575
Klb	575
Slc35f5	574
Pum1	574
Ndel1	573
Wdr38	573
C77080	573
Pla2g12a	573
Bmf	572
Angptl6	570
Entpd5	570
Ppfibp2	570
Fam91a1	569
Mat2b	568
Plekha3	568
Fkbp7	568
Nme6	566
Coa7	566
Bbc3	566
Hist3h2a	566
Agtpbp1	566
Fgd4	566
Azin1	565
Ankrd28	565
Borcs6	565
Msi2	564
Deaf1	563
Tmem80	563
Eef1aknmt	562
Ninj1	561
Hes6	561
Map3k9	560
Cfap20	559
Irf8	559
Fam102a	559
Ppl	558
Clpp	557
Pes1	556
Parp2	556
Gns	555
Slc25a51	555
Zfp141	555
Tbc1d1	554
0610040J01Rik	553
Atp7b	553
Alg11	553
Prdx2	553

Ssr1	552
Bsg	551
A430005L14Ri	551
Zfp36l1	551
Styx	549
Smarca2	546
Acaca	546
Osbpl1a	545
Cdk9	545
Rgs9bp	544
Ankrd27	544
Slmap	544
Sik1	543
Mindy2	543
Gnmt	542
Laptm4a	542
Top3a	542
Smcr8	542
Rab11fip2	541
Retreg1	541
Nme1	541
Serpina1b	540
Rmnd5a	540
Imp3	540
Myo1c	540
Ttc7b	540
Klf10	539
Inf2	539
Zfp750	539
Igsf23	538
Lonp1	537
Catsperd	537
Ggact	537
Aatf	537
Tmem43	536
Chchd4	536
Fadd	535
Mocs2	535
Erf	534
Pcgf5	534
Psen2	534
Cyp26a1	534
Zfp28	533
Cyp2c23	532
Nsd3	531
Letm2	531
Ywhaq	531
Cps1	531
P4hb	530
Arhgdia	530
Klhl21	530
Id1	529
Rbfox2	529
Ccdc33	529
Hebp1	528
Rabggta	528
Rps11	528
Rpl13a	528

Sos2	528
Gnai1	528
Masp1	527
1810055G02Ri	526
Cndp2	526
Pfn1	525
Eno3	525
Mapre2	525
Cc2d1b	525
Sbno1	525
Serpina3n	525
Cys1	525
Eef1a1	524
Ccdc38	524
Amdhd1	524
Esrp2	524
Syncrip	524
Sec31a	523
Cln5	523
Manf	523
Colgalt1	523
Rbm15b	523
Trp53bp2	523
Rubcn	523
Nmd3	522
Dock5	520
Atp8b1	518
Dyrk1b	518
Srd5a1	517
Nsun2	517
Cdv3	517
Sec14l4	517
Cyp2b10	517
Shox2	516
Frat2	516
Agbl5	516
Arap1	516
Gtdc1	515
Slc25a32	515
Dcaf13	515
Zfp281	515
Sema3b	515
Rsph9	515
Cited2	514
Erlin1	514
Eif4a1	513
Wbp2	513
Nsmf	513
Grsf1	513
Lmbr1l	512
Acaa2	512
Dpf2	512
Pld6	512
Slc25a33	512
Trip4	512
Pclaf	512
Xpo7	510
Btaf1	510

Lifr	510
Rrbp1	508
Slc37a1	508
Efna3	508
Mpp6	508
Asgr2	507
Uggt1	507
Reln	507
Riox2	506
Elk4	506
Abhd14b	505
Abhd14a	505
Usp42	505
Fnip2	504
Hsd17b2	504
Adcy9	504
Tsc1	503
Tecr	503
Gadd45g	503
Retreg2	502
Cnppd1	502
Rangap1	502
Cldn3	502
Hilpda	501
Hook2	501
Ass1	501
Smagp	501
Kras	501
Odf2	500
Stim1	500
Akap10	500
Vgf	500
Tmem266	500

Supplementary Table 3. Up-regulated genes in ventricle of 5/6Nx mice

Gene Symbol	Genbank accession	Description	5/6Nx / Sham	
			Z-score	Fold change
Mybphl	NM_026831	Mus musculus myosin binding protein H-like	17.804	2200.909753
Sln	NM_025540	Mus musculus sarcolipin	16.242	373.051075
Myl7	NM_022879	Mus musculus myosin, light polypeptide 7, regulatory	15.764	313.516979
Mlana	NM_029993	Mus musculus melan-A	13.028	279.484830
Hamp	NM_032541	Mus musculus hepcidin antimicrobial peptide	10.594	265.627511
Myl4	NM_010858	Mus musculus myosin, light polypeptide 4	15.155	251.114503
Adamts8	NM_013906	Mus musculus a disintegrin-like and metallopeptidase with thrombospondin type 1 motif, 8	12.369	210.173299
Dkk3	NM_015814	Mus musculus dickkopf homolog 3	15.637	164.101056
Tmem163	NM_028135	Mus musculus transmembrane protein 163	11.646	153.795002
Cilp2	NM_026818	Mus musculus cartilage intermediate layer protein 2	9.410	142.601871
Fgf12	NM_183064	Mus musculus fibroblast growth factor 12	11.469	142.460989
Mybph	NM_016749	Mus musculus myosin binding protein H	8.563	91.377821
Fbxw18	NM_001033794	Mus musculus F-box and WD-40 domain protein 18	8.269	78.278627
Sbk3	NM_001200041	Mus musculus SH3 domain binding kinase family, member 3	13.364	57.558427
Gsg1	NM_010352	Mus musculus germ cell-specific gene 1	7.562	53.993946
Fbxw20	NM_001008428	Mus musculus F-box and WD-40 domain protein 20	4.026	48.888804
DXBay18	NM_001025384	Mus musculus DNA segment, Chr X, Baylor 18	3.989	47.268102
Cfd	NM_013459	Mus musculus complement factor D	11.493	42.502772
Car3	NM_007606	Mus musculus carbonic anhydrase 3	8.599	41.229398
Adrb3	NM_013462	Mus musculus adrenergic receptor, beta 3	3.783	39.096938
Uts2b	NM_198166	Mus musculus urotensin 2B	6.911	38.346312
Gm5640	NM_001099302	Mus musculus predicted gene 5640	3.700	36.208410
Nxpe5	NM_001013773	Mus musculus neurexophilin and PC-esterase domain family, member 5	3.631	33.995133
Epyc	NM_007884	Mus musculus epiphycan	3.551	31.577004
Plin1	NM_175640	Mus musculus perilipin 1	3.504	30.243886
Ucp1	NM_009463	Mus musculus uncoupling protein 1	3.395	27.357530
Gm5936	NM_001081670	Mus musculus predicted gene 5936	3.369	26.708126
Cidec	NM_178373	Mus musculus cell death-inducing DFFA-like effector c	7.212	22.641907
Sds	NM_145565	Mus musculus serine dehydratase	5.780	21.165913
Alox8	NM_009661	Mus musculus arachidonate 8-lipoxygenase	3.074	20.355734
Adipoq	NM_009605	Mus musculus adiponectin, C1Q and collagen domain containing	5.643	19.703827
Car13	NM_024495	Mus musculus carbonic anhydrase 13	2.994	18.905191
Fras1	NM_175473	Mus musculus Fraser syndrome 1 homolog	5.538	18.644631
Ccr6	NM_009835	Mus musculus chemokine receptor 6	2.952	18.191914
Sphkap	NM_172430	Mus musculus SPHK1 interactor, AKAP domain containing	2.942	18.033549
Rab33a	NM_011228	Mus musculus RAB33A, member RAS oncogene family	5.457	17.862596
Npy1r	NM_010934	Mus musculus neuropeptide Y receptor Y1	6.625	17.563680
Cpa3	NM_007753	Mus musculus carboxypeptidase A3, mast cell	2.900	17.350335
Rxfp1	NM_212452	Mus musculus relaxin/insulin-like family peptide receptor 1	5.365	17.025630
Tescl	NM_001163810	Mus musculus tescalcin-like	5.349	16.875743
Pianp	NM_175696	Mus musculus PILR alpha associated neural protein	5.311	16.543489
Slurp1	NM_020519	Mus musculus secreted Ly6/Plaur domain containing 1	2.810	15.964036
Spp1	NM_009263	Mus musculus secreted phosphoprotein 1	5.213	15.715735
Adcy1	NM_009622	Mus musculus adenylate cyclase 1	5.212	15.706636
Iqcg	NM_178378	Mus musculus IQ motif containing G	8.419	15.599719
Retn	NM_001204959	Mus musculus resistin	5.196	15.577290
Ccl8	NM_021443	Mus musculus chemokine ligand 8	6.261	15.007080
Cyp2e1	NM_021282	Mus musculus cytochrome P450, family 2, subfamily e, polypeptide 1	2.742	14.994796
Chil1	NM_007695	Mus musculus chitinase-like 1	2.730	14.829665
Rspo3	NM_028351	Mus musculus R-spondin 3 homolog	2.712	14.590860
Rgs7	NM_011880	Mus musculus regulator of G protein signaling 7	2.705	14.491757
Hp	NM_017370	Mus musculus haptoglobin	8.138	14.232829
Mcpt4	NM_010779	Mus musculus mast cell protease 4	4.980	13.903886
Cxcl13	NM_018866	Mus musculus chemokine ligand 13	4.944	13.644178
Spib	NM_019866	Mus musculus Spi-B transcription factor	2.626	13.480262
Ptgfr	NM_008966	Mus musculus prostaglandin F receptor	5.995	13.378465
Nppa	NM_008725	Mus musculus natriuretic peptide type A	9.424	13.112126
1500015O10Rik	NM_024283	Mus musculus RIKEN cDNA 1500015O10 gene	5.936	13.042338
Ms4a8a	NM_022430	Mus musculus membrane-spanning 4-domains, subfamily A, member 8A	2.584	12.960823
Mcemp1	NM_026985	Mus musculus mast cell expressed membrane protein 1	2.551	12.581646
9030624J02Rik	NM_027815	Mus musculus RIKEN cDNA 9030624J02 gene	2.522	12.251745
Cybrd1	NM_028593	Mus musculus cytochrome b reductase 1	4.733	12.213636
Krt7	NM_033073	Mus musculus keratin 7	2.516	12.176127
Fbxw15	NM_199036	Mus musculus F-box and WD-40 domain protein 15	2.493	11.929226
Krt14	NM_016958	Mus musculus keratin 14	2.485	11.838091
Slc7a10	NM_017394	Mus musculus solute carrier family 7 , member 10	2.474	11.719561
Myl1	NM_021285	Mus musculus myosin, light polypeptide 1	6.731	11.703641
Scd4	NM_183216	Mus musculus stearoyl-coenzyme A desaturase 4	4.604	11.411532
Calcb	NM_054084	Mus musculus calcitonin-related polypeptide, beta	2.435	11.304662
Sept3	NM_011889	Mus musculus septin 3	2.434	11.293809
Dmkn	NM_001166173	Mus musculus dermokine	4.577	11.250826
Pon1	NM_011134	Mus musculus paraoxonase 1	2.403	10.972729
Ms4a1	NM_007641	Mus musculus membrane-spanning 4-domains, subfamily A, member 1	4.527	10.957038
Cldn22	NM_029383	Mus musculus claudin 22	2.397	10.917832
Fxyd3	NM_008557	Mus musculus FXYD domain-containing ion transport regulator 3	2.386	10.802360
Pla2g2e	NM_012044	Mus musculus phospholipase A2, group IIE	2.383	10.779485
Fbxl13	NM_001199632	Mus musculus F-box and leucine-rich repeat protein 13	2.379	10.731488
Galr1	NM_008082	Mus musculus galanin receptor 1	2.371	10.657225
Shox2	NM_001302359	Mus musculus short stature homeobox 2	4.468	10.624504

Tmem45b	NM_144936	Mus musculus transmembrane protein 45b	2.365	10.596692
Bmp10	NM_009756	Mus musculus bone morphogenetic protein 10	2.360	10.552099
Gfap	NM_001131020	Mus musculus glial fibrillary acidic protein	2.351	10.459384
Il6	NM_031168	Mus musculus interleukin 6	2.348	10.429716
Casq1	NM_009813	Mus musculus calsequestrin 1	5.401	10.348714
Zbtb32	NM_021397	Mus musculus zinc finger and BTB domain containing 32	2.315	10.125124
Sytl2	NM_001040087	Mus musculus synaptotagmin-like 2	4.372	10.098870
Acvr1c	NM_001111030	Mus musculus activin A receptor, type IC	2.300	9.987140
Kap	NM_010594	Mus musculus kidney androgen regulated protein	2.297	9.958590
Plac8	NM_139198	Mus musculus placenta-specific 8	4.307	9.763778
Nnat	NM_010923	Mus musculus neuronatin	2.274	9.744750
Alox15	NM_009660	Mus musculus arachidonate 15-lipoxygenase	2.272	9.730880
Stard10	NM_019990	Mus musculus START domain containing 10	6.959	9.691610
Pitx2	NM_001042502	Mus musculus paired-like homeodomain transcription factor 2	4.292	9.687792
Cpne5	NM_153166	Mus musculus copine V	5.241	9.660307
Spock2	NM_052994	Mus musculus sparc/osteonectin, cwcv and kazal-like domains proteoglycan 2	4.283	9.640327
Lppr5	NM_001305451	Mus musculus lipid phosphate phosphatase-related protein type 5	2.257	9.597680
Aqp5	NM_009701	Mus musculus aquaporin 5	2.247	9.510816
Cd2	NM_013486	Mus musculus CD2 antigen	4.236	9.406387
Rd3l	NM_001127685	Mus musculus retinal degeneration 3-like	2.231	9.365049
Cck	NM_001284508	Mus musculus cholecystokinin	2.222	9.290246
Spink2	NM_183284	Mus musculus serine peptidase inhibitor, Kazal type 2	4.205	9.254634
Lrtm2	NM_172492	Mus musculus leucine-rich repeats and transmembrane domains 2	2.205	9.145160
Slc22a7	NM_144856	Mus musculus solute carrier family 22 , member 7	2.186	8.988493
Nfasc	NM_182716	Mus musculus neurofascin	2.181	8.949476
Thsd7b	NM_172485	Mus musculus thrombospondin, type I, domain containing 7B	2.176	8.909817
Crb2	NM_001163566	Mus musculus crumbs family member 2	4.124	8.866952
Frem1	NM_177863	Mus musculus Fras1 related extracellular matrix protein 1	2.164	8.808723
Acp5	NM_001102405	Mus musculus acid phosphatase 5, tartrate resistant	6.661	8.795065
Vat1l	NM_173016	Mus musculus vesicle amine transport protein 1 homolog-like	2.161	8.781455
Igfbpl1	NM_018741	Mus musculus insulin-like growth factor binding protein-like 1	2.153	8.723366
Mmp27	NM_001030289	Mus musculus matrix metallopeptidase 27	2.146	8.664943
Tbc1d1	NM_019636	Mus musculus TBC1 domain family, member 1	2.142	8.629476
Trim67	NM_198632	Mus musculus tripartite motif-containing 67	2.142	8.628797
Nrg2	NM_001167891	Mus musculus neuregulin 2	2.138	8.600410
Fbxw27	NM_001303003	Mus musculus F-box and WD-40 domain protein 27	2.136	8.582641
Glb1l2	NM_153803	Mus musculus galactosidase, beta 1-like 2	2.127	8.517108
Prr15l	NM_146026	Mus musculus proline rich 15-like	4.039	8.478297
H2-Ea-ps	NM_010381	Mus musculus histocompatibility 2, class II antigen E alpha, pseudogene	2.118	8.440625
Pck1	NM_011044	Mus musculus phosphoenolpyruvate carboxykinase 1, cytosolic	4.023	8.410078
Lrrn4	NM_177303	Mus musculus leucine rich repeat neuronal 4	4.019	8.389311
Clic6	NM_172469	Mus musculus chloride intracellular channel 6	2.096	8.274080
Olfr1448	NM_146701	Mus musculus olfactory receptor 1448	2.066	8.046216
Cldn11	NM_008770	Mus musculus claudin 11	2.063	8.028616
Nlrp5	NM_011860	Mus musculus NLR family, pyrin domain containing 5	2.060	8.002339
C4b	NM_009780	Mus musculus complement component 4B	6.846	7.966100
Cyp2s1	NM_028775	Mus musculus cytochrome P450, family 2, subfamily s, polypeptide 1	4.792	7.954513
Folr1	NM_001252552	Mus musculus folate receptor 1	2.053	7.953435
Krt8	NM_031170	Mus musculus keratin 8	3.916	7.948365
Lrp2	NM_001081088	Mus musculus low density lipoprotein receptor-related protein 2	2.047	7.906941
Gm17455	NM_001164374	Mus musculus predicted gene, 17455	2.047	7.905920
Kcnma1	NM_001253378	Mus musculus potassium large conductance calcium-activated channel, subfamily M, alpha member 1	2.037	7.838682
Stmnd1	NM_001005422	Mus musculus stathmin domain containing 1	2.037	7.837127
Ptx4	NM_026747	Mus musculus pentraxin 4	3.878	7.791653
Prss12	NM_008939	Mus musculus protease, serine 12 neurotrypsin	3.877	7.789390
Upk3b	NM_175309	Mus musculus uroplakin 3B	3.863	7.731508
Rarres1	NM_001164763	Mus musculus retinoic acid receptor responder 1	2.022	7.730552
Ric3	NM_001038624	Mus musculus resistance to inhibitors of cholinesterase 3 homolog	2.019	7.704770
Prdm6	NM_001033281	Mus musculus PR domain containing 6	3.855	7.698261
Timp1	NM_001044384	Mus musculus tissue inhibitor of metalloproteinase 1	6.252	7.696111
Fbxw26	NM_198674	Mus musculus F-box and WD-40 domain protein 26	2.015	7.679623
Saa3	NM_011315	Mus musculus serum amyloid A 3	3.847	7.664463
Dnah7b	NM_001160386	Mus musculus dynein, axonemal, heavy chain 7B	2.011	7.654408
C530008M17Rik	NM_001163793	Mus musculus RIKEN cDNA C530008M17 gene	2.005	7.611007
Slc22a1	NM_009202	Mus musculus solute carrier family 22 , member 1	6.205	7.579713
Clca3a1	NM_009899	Mus musculus chloride channel calcium activated 3A1	3.820	7.556159
Cacna1d	NM_001302637	Mus musculus calcium channel, voltage-dependent, L type, alpha 1D subunit	1.997	7.554915
4930435E12Rik	NM_029042	Mus musculus RIKEN cDNA 4930435E12 gene	1.979	7.430491
Steap2	NM_001103157	Mus musculus six transmembrane epithelial antigen of prostate 2	1.977	7.418105
Enkur	NM_027728	Mus musculus enkurin, TRPC channel interacting protein	3.784	7.416797
Pdzrn4	NM_001164593	Mus musculus PDZ domain containing RING finger 4	3.781	7.406522
Bdh2	NM_001172055	Mus musculus 3-hydroxybutyrate dehydrogenase, type 2	3.768	7.353643
Rab3b	NM_023537	Mus musculus RAB3B, member RAS oncogene family	4.608	7.347373
Cldn23	NM_027998	Mus musculus claudin 23	1.962	7.312197
Lect1	NM_010701	Mus musculus leukocyte cell derived chemotaxin 1	1.957	7.280877
Rps6ka6	NM_025949	Mus musculus ribosomal protein S6 kinase polypeptide 6	1.953	7.255479
Sall1	NM_021390	Mus musculus sal-like 1	1.951	7.243194
Cdh3	NM_001037809	Mus musculus cadherin 3	1.949	7.227851
Rab27a	NM_023635	Mus musculus RAB27A, member RAS oncogene family	1.943	7.189346
Btbd9	NM_172618	Mus musculus BTB domain containing 9	4.555	7.180257
Baiap2l1	NM_025833	Mus musculus BAI1-associated protein 2-like 1	4.526	7.090289
Cpz	NM_153107	Mus musculus carboxypeptidase Z	3.694	7.074235
Sgip1	NM_001285852	Mus musculus SH3-domain GRB2-like interacting protein 1	1.924	7.062723
Wfdc2	NM_026323	Mus musculus WAP four-disulfide core domain 2	3.687	7.048546
Tnfrsf11b	NM_008764	Mus musculus tumor necrosis factor receptor superfamily, member 11b	1.918	7.025526
Dio2	NM_010050	Mus musculus deiodinase, iodothyronine, type II	4.502	7.017787

Bst1	NM_009763	Mus musculus bone marrow stromal cell antigen 1	4.497	7.003383
Fst	NM_008046	Mus musculus follistatin	1.915	7.003318
Plet1	NM_029639	Mus musculus placenta expressed transcript 1	3.673	6.994657
Ap1s3	NM_183027	Mus musculus adaptor-related protein complex AP-1, sigma 3	1.912	6.987768
Btla	NM_001037719	Mus musculus B and T lymphocyte associated	1.907	6.953088
Nfe2l3	NM_010903	Mus musculus nuclear factor, erythroid derived 2, like 3	1.907	6.952037
Bpifb4	NM_001034875	Mus musculus BPI fold containing family B, member 4	1.904	6.936524
Cfi	NM_007686	Mus musculus complement component factor i	3.649	6.907815
Cntfr	NM_001136056	Mus musculus ciliary neurotrophic factor receptor	3.640	6.875001
Podn	NM_001285956	Mus musculus podocan	5.882	6.822630
Flrt3	NM_001172160	Mus musculus fibronectin leucine rich transmembrane protein 3	1.885	6.812725
Tas2r102	NM_199153	Mus musculus taste receptor, type 2, member 102	1.877	6.763770
Cd209d	NM_130904	Mus musculus CD209d antigen	3.606	6.752619
Syn2	NM_013681	Mus musculus synapsin II	4.413	6.752379
Upk1b	NM_178924	Mus musculus uroplakin 1B	3.588	6.690457
Fbln7	NM_024237	Mus musculus fibulin 7	3.583	6.674111
Comp	NM_016685	Mus musculus cartilage oligomeric matrix protein	5.808	6.659224
C130026I21Rik	NM_175219	Mus musculus RIKEN cDNA C130026I21 gene	1.855	6.627505
Sbk2	NM_001146329	Mus musculus SH3-binding domain kinase family, member 2	3.566	6.612491
Ly6d	NM_010742	Mus musculus lymphocyte antigen 6 complex, locus D	3.564	6.606336
Gng2	NM_010315	Mus musculus guanine nucleotide binding protein , gamma 2	4.361	6.604113
Serpina3i	NM_001199940	Mus musculus serine peptidase inhibitor, clade A, member 3I	1.851	6.602167
Speer4a	NM_029376	Mus musculus spermatogenesis associated glutamate -rich protein 4a	1.846	6.570372
Mmp16	NM_019724	Mus musculus matrix metallopeptidase 16	1.841	6.542821
Wwc1	NM_170779	Mus musculus WW, C2 and coiled-coil domain containing 1	1.839	6.527944
Col8a2	NM_199473	Mus musculus collagen, type VIII, alpha 2	1.836	6.515668
Slc38a8	NM_001009950	Mus musculus solute carrier family 38, member 8	3.531	6.492127
Camk1d	NM_177343	Mus musculus calcium/calmodulin-dependent protein kinase ID	4.316	6.475243
Gabra3	NM_008067	Mus musculus gamma-aminobutyric acid A receptor, subunit alpha 3	1.821	6.424524
Lgals2	NM_025622	Mus musculus lectin, galactose-binding, soluble 2	1.817	6.398539
Foxp2	NM_053242	Mus musculus forkhead box P2	3.501	6.392049
Glyat	NM_145935	Mus musculus glycine-N-acyltransferase	1.815	6.388253
Sostdc1	NM_025312	Mus musculus sclerostin domain containing 1	1.815	6.385745
Fhad1	NM_177868	Mus musculus forkhead-associated phosphopeptide binding domain 1	1.813	6.375062
Tnfrsf13c	NM_028075	Mus musculus tumor necrosis factor receptor superfamily, member 13c	1.813	6.373724
Plekha7	NM_001305186	Mus musculus pleckstrin homology domain containing, family A member 7	3.495	6.372640
Pou2af1	NM_011136	Mus musculus POU domain, class 2, associating factor 1	3.483	6.332385
Msln	NM_018857	Mus musculus mesothelin	5.647	6.319525
Nkain4	NM_021426	Mus musculus Na+/K+ transporting ATPase interacting 4	4.259	6.318520
Adamts19	NM_175506	Mus musculus a disintegrin-like and metallopeptidase with thrombospondin type 1 motif, 19	3.475	6.306133
Wisp1	NM_018865	Mus musculus WNT1 inducible signaling pathway protein 1	4.238	6.260680
Slc39a8	NM_001135150	Mus musculus solute carrier family 39 , member 8	1.790	6.243944
Slco1c1	NM_021471	Mus musculus solute carrier organic anion transporter family, member 1c1	1.788	6.228516
Selp	NM_011347	Mus musculus selectin, platelet	1.784	6.209157
Dmrtb1	NM_019872	Mus musculus DMRT-like family B with proline-rich C-terminal, 1	1.783	6.203992
Ifi27l2a	NM_029803	Mus musculus interferon, alpha-inducible protein 27 like 2A	6.022	6.203722
Serpinb2	NM_011111	Mus musculus serine peptidase inhibitor, clade B, member 2	1.782	6.198082
Olfr1294	NM_146885	Mus musculus olfactory receptor 1294	1.771	6.132709
Srpx	NM_016911	Mus musculus sushi-repeat-containing protein	3.410	6.093202
Otop1	NM_172709	Mus musculus otopetrin 1	1.756	6.052588
Acsm1	NM_054094	Mus musculus acyl-CoA synthetase medium-chain family member 1	1.755	6.042181
Retnla	NM_020509	Mus musculus resistin like alpha	5.500	6.022604
Hapln1	NM_013500	Mus musculus hyaluronan and proteoglycan link protein 1	1.743	5.978456
Heatr9	NM_001045543	Mus musculus HEAT repeat containing 9	1.742	5.974105
Cxcr5	NM_007551	Mus musculus chemokine receptor 5	1.740	5.962449
Mdfi	NM_001109973	Mus musculus MyoD family inhibitor	5.439	5.904177
Stxbp5l	NM_172440	Mus musculus syntaxin binding protein 5-like	1.727	5.888549
Lgi2	NM_144945	Mus musculus leucine-rich repeat LGI family, member 2	3.342	5.878164
Palm3	NM_028877	Mus musculus paralemmin 3	1.723	5.870181
Arhgef4	NM_183019	Mus musculus Rho guanine nucleotide exchange factor 4	1.723	5.869837
Lrat	NM_023624	Mus musculus lecithin-retinol acyltransferase	1.723	5.868940
Krt19	NM_008471	Mus musculus keratin 19	4.084	5.857684
Anxa8	NM_013473	Mus musculus annexin A8	4.080	5.849250
Cst9	NM_009979	Mus musculus cystatin 9	1.719	5.845399
Capn6	NM_007603	Mus musculus calpain 6	1.717	5.838726
Cma1	NM_010780	Mus musculus chymase 1, mast cell	3.328	5.835479
Rbfox3	NM_001039167	Mus musculus RNA binding protein, fox-1 homolog 3	1.711	5.807148
Fgfbp1	NM_001271616	Mus musculus fibroblast growth factor binding protein 1	3.317	5.801631
Col12a1	NM_001290308	Mus musculus collagen, type XII, alpha 1	1.709	5.795272
Gal3st2	NM_199366	Mus musculus galactose-3-O-sulfotransferase 2	3.312	5.786837
Cacna1i	NM_001044308	Mus musculus calcium channel, voltage-dependent, alpha 1I subunit	1.703	5.763946
Barx2	NM_013800	Mus musculus BarH-like homeobox 2	1.701	5.753922
Dner	NM_152915	Mus musculus delta/notch-like EGF-related receptor	1.698	5.733140
Sult1e1	NM_023135	Mus musculus sulfotransferase family 1E, member 1	3.285	5.704615
Gjb3	NM_001160012	Mus musculus gap junction protein, beta 3	1.690	5.694357
Cited1	NM_007709	Mus musculus Cbp/p300-interacting transactivator with Glu/Asp-rich carboxy-terminal domain 1	4.003	5.655766
Pik3r5	NM_177320	Mus musculus phosphoinositide-3-kinase, regulatory subunit 5, p101	1.664	5.558674
Col4a6	NM_053185	Mus musculus collagen, type IV, alpha 6	3.959	5.550432
Cacna2d2	NM_001174047	Mus musculus calcium channel, voltage-dependent, alpha 2/delta subunit 2	5.245	5.543303
Ephb2	NM_001290753	Mus musculus Eph receptor B2	1.659	5.533384
Nkx6-2	NM_183248	Mus musculus NK6 homeobox 2	1.658	5.529025
Frmpd1	NM_001081172	Mus musculus FERM and PDZ domain containing 1	3.222	5.520437
Prrx2	NM_009116	Mus musculus paired related homeobox 2	3.213	5.492560
Mmp3	NM_010809	Mus musculus matrix metallopeptidase 3	1.650	5.489070
Slco2a1	NM_033314	Mus musculus solute carrier organic anion transporter family, member 2a1	3.927	5.473078
Cd19	NM_009844	Mus musculus CD19 antigen	3.199	5.453793

Gja5	NM_008121	Mus musculus gap junction protein, alpha 5	5.185	5.436045
Crip3	NM_053250	Mus musculus cysteine-rich protein 3	3.906	5.423445
Insc	NM_173767	Mus musculus inscuteable homolog	1.637	5.420427
Sp140	NM_001013817	Mus musculus Sp140 nuclear body protein	3.185	5.413965
Gpm6a	NM_153581	Mus musculus glycoprotein m6a	3.875	5.353019
Smad9	NM_019483	Mus musculus SMAD family member 9	1.619	5.334592
Tnfrsf18	NM_009400	Mus musculus tumor necrosis factor receptor superfamily, member 18	1.617	5.323664
Abi3bp	NM_178790	Mus musculus ABI gene family, member 3 binding protein	1.615	5.314726
Lypd1	NM_145100	Mus musculus Ly6/Plaur domain containing 1	1.612	5.297153
Cand1	NM_027994	Mus musculus cullin associated and neddylation disassociated 1	1.611	5.295564
Zfp579	NM_026741	Mus musculus zinc finger protein 579	5.497	5.290588
Hamp2	NM_183257	Mus musculus hepcidin antimicrobial peptide 2	3.141	5.288788
Slc7a7	NM_011405	Mus musculus solute carrier family 7 , member 7	5.092	5.272778
Itgax	NM_021334	Mus musculus integrin alpha X	1.606	5.272294
Glt8d2	NM_029102	Mus musculus glycosyltransferase 8 domain containing 2	1.605	5.262897
Cfb	NM_008198	Mus musculus complement factor B	3.825	5.237269
Zbtb8b	NM_153541	Mus musculus zinc finger and BTB domain containing 8b	1.597	5.226579
Cldn3	NM_009902	Mus musculus claudin 3	1.597	5.224557
Bcan	NM_007529	Mus musculus brevican	1.596	5.223196
D430019H16Rik	NM_001252508	Mus musculus RIKEN cDNA D430019H16 gene	3.117	5.222619
Slc24a4	NM_172152	Mus musculus solute carrier family 24 , member 4	1.590	5.190804
Stat4	NM_011487	Mus musculus signal transducer and activator of transcription 4	1.587	5.177581
Frmd3	NM_172869	Mus musculus FERM domain containing 3	1.585	5.170872
Gm136	NM_001033255	Mus musculus predicted gene 136	1.583	5.160721
Smoc1	NM_001146217	Mus musculus SPARC related modular calcium binding 1	3.790	5.159063
Rab38	NM_028238	Mus musculus RAB38, member RAS oncogene family	1.583	5.158302
Cd79b	NM_008339	Mus musculus CD79B antigen	3.789	5.157957
Krt42	NM_212483	Mus musculus keratin 42	3.088	5.144085
Htr2b	NM_008311	Mus musculus 5-hydroxytryptamine receptor 2B	1.580	5.143683
Traf3ip3	NM_153137	Mus musculus TRAF3 interacting protein 3	1.576	5.125453
Fmod	NM_021355	Mus musculus fibromodulin	3.071	5.100164
Slc1a3	NM_148938	Mus musculus solute carrier family 1 , member 3	3.071	5.098555
Ildr2	NM_001164528	Mus musculus immunoglobulin-like domain containing receptor 2	3.071	5.098307
H60b	NM_001177775	Mus musculus histocompatibility 60b	1.562	5.062827
Dixdc1	NM_178118	Mus musculus DIX domain containing 1	1.562	5.059919
Depdc1b	NM_178683	Mus musculus DEP domain containing 1B	1.561	5.057856
Met	NM_008591	Mus musculus met proto-oncogene	1.561	5.056915
Slc23a3	NM_194333	Mus musculus solute carrier family 23 , member 3	1.561	5.054333
Clca3a2	NM_030601	Mus musculus chloride channel calcium activated 3A2	1.557	5.039287
Cgn	NM_001037711	Mus musculus cingulin	3.048	5.038360
Hcn1	NM_010408	Mus musculus hyperpolarization-activated, cyclic nucleotide-gated K+ 1	1.551	5.010826
1700001C02Rik	NM_029285	Mus musculus RIKEN cDNA 1700001C02 gene	1.551	5.007724
Clvs1	NM_028940	Mus musculus clavesin 1	3.028	4.983681
Adgrg6	NM_001002268	Mus musculus adhesion G protein-coupled receptor G6	1.544	4.976
Mbp	NM_001025245	Mus musculus myelin basic protein	3.702	4.967
Rbm46	NM_001277170	Mus musculus RNA binding motif protein 46	1.541	4.965
Tmem74	NM_175502	Mus musculus transmembrane protein 74	1.536	4.939
Fpr1	NM_013521	Mus musculus formyl peptide receptor 1	1.524	4.886
Xpnpep2	NM_001289729	Mus musculus X-prolyl aminopeptidase 2, membrane-bound	3.662	4.882
Sort1	NM_001271599	Mus musculus sortilin 1	5.221	4.866
Vill	NM_001164567	Mus musculus villin-like	1.517	4.857
Coch	NM_007728	Mus musculus coagulation factor C homolog	1.517	4.856
Gm10375	NM_001098269	Mus musculus predicted gene 10375	1.512	4.835
Nmnat2	NM_175460	Mus musculus nicotinamide nucleotide adenylyltransferase 2	1.512	4.832
Klra4	NM_010649	Mus musculus killer cell lectin-like receptor, subfamily A, member 4	1.511	4.828
Arxes1	NM_029541	Mus musculus adipocyte-related X-chromosome expressed sequence 1	1.510	4.824
Ear12	NM_001012766	Mus musculus eosinophil-associated, ribonuclease A family, member 12	1.508	4.815
Card11	NM_175362	Mus musculus caspase recruitment domain family, member 11	1.507	4.809
Igsf23	NM_027308	Mus musculus immunoglobulin superfamily, member 23	2.956	4.799
L1cam	NM_008478	Mus musculus L1 cell adhesion molecule	1.504	4.798
Bzrap1	NM_172449	Mus musculus benzodiazepine receptor associated protein 1	1.502	4.789
Lta	NM_010735	Mus musculus lymphotoxin A	1.501	4.786
C2	NM_013484	Mus musculus complement component 2	3.616	4.785
Ninl	NM_207204	Mus musculus ninein-like	1.497	4.766
Plagl1	NM_009538	Mus musculus pleiomorphic adenoma gene-like 1	4.780	4.762
Bpifa6	NM_001080811	Mus musculus BPI fold containing family A, member 6	1.496	4.762
Rspo1	NM_138683	Mus musculus R-spondin homolog	4.777	4.758
Gipr	NM_001080815	Mus musculus gastric inhibitory polypeptide receptor	1.494	4.753
Cd163	NM_053094	Mus musculus CD163 antigen	1.493	4.749
Camk1g	NM_144817	Mus musculus calcium/calmodulin-dependent protein kinase I gamma	3.595	4.742
Peg3	NM_008817	Mus musculus paternally expressed 3	2.931	4.738
Ahsg	NM_013465	Mus musculus alpha-2-HS-glycoprotein	4.759	4.731
Slpi	NM_011414	Mus musculus secretory leukocyte peptidase inhibitor	2.927	4.727
Hal	NM_010401	Mus musculus histidine ammonia lyase	3.587	4.725
Pabpc4l	NM_001101479	Mus musculus poly binding protein, cytoplasmic 4-like	1.487	4.722
Ctgf	NM_010217	Mus musculus connective tissue growth factor	5.117	4.714
H2-Ob	NM_010389	Mus musculus histocompatibility 2, O region beta locus	1.484	4.710
Krt18	NM_010664	Mus musculus keratin 18	3.575	4.702
Stk26	NM_133729	Mus musculus serine/threonine kinase 26	1.482	4.699
Arxes2	NM_029823	Mus musculus adipocyte-related X-chromosome expressed sequence 2	2.914	4.695
Olfm1	NM_019498	Mus musculus olfactomedin 1	4.730	4.685
Agap2	NM_001033263	Mus musculus ArfGAP with GTPase domain, ankyrin repeat and PH domain 2	1.478	4.685
Optc	NM_054076	Mus musculus opticin	1.473	4.661
Asic1	NM_001289791	Mus musculus acid-sensing ion channel 1	1.471	4.656
Hist1h4m	NM_001195421	Mus musculus histone cluster 1, H4m	1.463	4.621
Ascl2	NM_008554	Mus musculus achaete-scute complex homolog 2	1.462	4.617

Lbp	NM_008489	Mus musculus lipopolysaccharide binding protein	3.529	4.610
1700026L06Rik	NM_027283	Mus musculus RIKEN cDNA 1700026L06 gene	1.455	4.585
Gcnt1	NM_173442	Mus musculus glucosaminyl transferase 1, core 2	3.512	4.575
Gjb5	NM_010291	Mus musculus gap junction protein, beta 5	1.452	4.573
Zgrf1	NM_197997	Mus musculus zinc finger, GRF-type containing 1	1.448	4.557
Ept1	NM_027652	Mus musculus ethanolaminephosphotransferase 1	1.448	4.556
Gm4841	NM_001034859	Mus musculus predicted gene 4841	1.447	4.553
Hpca	NM_001130419	Mus musculus hippocalcin	1.447	4.552
Ncam1	NM_010875	Mus musculus neural cell adhesion molecule 1	2.852	4.545
Tmem119	NM_146162	Mus musculus transmembrane protein 119	3.493	4.538
Ptn	NM_008973	Mus musculus pleiotrophin	2.849	4.537
Stap1	NM_019992	Mus musculus signal transducing adaptor family member 1	1.442	4.534
Slmo1	NM_144867	Mus musculus slowmo homolog 1	1.442	4.532
Ceacam16	NM_001033419	Mus musculus carcinoembryonic antigen-related cell adhesion molecule 16	1.441	4.529
Alppl2	NM_007433	Mus musculus alkaline phosphatase, placental-like 2	1.441	4.527
Ccl12	NM_011331	Mus musculus chemokine ligand 12	3.477	4.507
Add2	NM_001271859	Mus musculus adducin 2	1.436	4.505
Olfr1225	NM_146891	Mus musculus olfactory receptor 1225	1.427	4.468
Ptafr	NM_001081211	Mus musculus platelet-activating factor receptor	2.817	4.462
Cd24a	NM_009846	Mus musculus CD24a antigen	3.454	4.462
Pear1	NM_001289600	Mus musculus platelet endothelial aggregation receptor 1	1.417	4.428
Mfap2	NM_008546	Mus musculus microfibrillar-associated protein 2	3.434	4.424
Lrrc75b	NM_198860	Mus musculus leucine rich repeat containing 75B	2.800	4.423
Sfrp5	NM_018780	Mus musculus secreted frizzled-related sequence protein 5	3.430	4.415
Hmga2	NM_010441	Mus musculus high mobility group AT-hook 2	1.412	4.409
H2-T9	NM_010399	Mus musculus histocompatibility 2, T region locus 9	2.793	4.406
Col6a5	NM_001167923	Mus musculus collagen, type VI, alpha 5	2.778	4.372
Cd209b	NM_026972	Mus musculus CD209b antigen	2.778	4.372
Myl9	NM_172118	Mus musculus myosin, light polypeptide 9, regulatory	4.024	4.370
C4a	NM_011413	Mus musculus complement component 4A	2.777	4.368
Sez6l	NM_001253916	Mus musculus seizure related 6 homolog like	1.399	4.358
Gxylt2	NM_198612	Mus musculus glucoside xylosyltransferase 2	2.758	4.327
St8sia5	NM_153124	Mus musculus ST8 alpha-N-acetyl-neuraminide alpha-2,8-sialyltransferase 5	3.368	4.299
Fn1	NM_010233	Mus musculus fibronectin 1	4.807	4.291
Cnn1	NM_009922	Mus musculus calponin 1	4.452	4.280
B3gnt3	NM_028189	Mus musculus UDP-GlcNAc:betaGal beta-1,3-N-acetylglucosaminyltransferase 3	1.377	4.267
Pamr1	NM_173749	Mus musculus peptidase domain containing associated with muscle regeneration 1	3.340	4.248
Sema3d	NM_028882	Mus musculus sema domain, immunoglobulin domain , short basic domain, secreted, 3D	3.339	4.246
Atp6v0a4	NM_080467	Mus musculus ATPase, H+ transporting, lysosomal V0 subunit A4	2.711	4.221
Tmem158	NM_001002267	Mus musculus transmembrane protein 158	4.405	4.215
Slc2a13	NM_001033633	Mus musculus solute carrier family 2 , member 13	2.709	4.215
Ang4	NM_177544	Mus musculus angiogenin, ribonuclease A family, member 4	1.363	4.214
Flrt1	NM_201411	Mus musculus fibronectin leucine rich transmembrane protein 1	2.707	4.211
Smim1	NM_001163721	Mus musculus small integral membrane protein 1	3.320	4.211
Naip6	NM_010871	Mus musculus NLR family, apoptosis inhibitory protein 6	1.358	4.196
Tceal5	NM_177919	Mus musculus transcription elongation factor A -like 5	2.695	4.185
Uncx	NM_013702	Mus musculus UNC homeobox	1.355	4.182
Hvcn1	NM_001042489	Mus musculus hydrogen voltage-gated channel 1	2.690	4.173
Slit3	NM_011412	Mus musculus slit homolog 3	3.296	4.168
Gm5141	NM_001256065	Mus musculus predicted gene 5141	1.348	4.154
Plac9a	NM_207229	Mus musculus placenta specific 9a	4.691	4.142
Plvap	NM_032398	Mus musculus plasmalemma vesicle associated protein	4.331	4.114
Marco	NM_010766	Mus musculus macrophage receptor with collagenous structure	2.659	4.106
Ptgs2	NM_011198	Mus musculus prostaglandin-endoperoxide synthase 2	1.334	4.104
Cilp	NM_173385	Mus musculus cartilage intermediate layer protein, nucleotide pyrophosphohydrolase	3.258	4.100
Mcoln2	NM_026656	Mus musculus mucolipin 2	1.332	4.094
Rab6b	NM_173781	Mus musculus RAB6B, member RAS oncogene family	2.649	4.084
Piezo2	NM_001039485	Mus musculus piezo-type mechanosensitive ion channel component 2	2.646	4.077
Lsr	NM_017405	Mus musculus lipolysis stimulated lipoprotein receptor	3.237	4.062
Csf2rb	NM_007780	Mus musculus colony stimulating factor 2 receptor, beta, low-affinity	2.635	4.055
Wnt2	NM_023653	Mus musculus wingless-type MMTV integration site family, member 2	2.631	4.046
6330403K07Rik	NM_134022	Mus musculus RIKEN cDNA 6330403K07 gene	2.625	4.033
Sfrp4	NM_016687	Mus musculus secreted frizzled-related protein 4	1.315	4.030
Nov	NM_010930	Mus musculus nephroblastoma overexpressed gene	3.216	4.026
Mpzl2	NM_007962	Mus musculus myelin protein zero-like 2	3.212	4.019
Pmaip1	NM_021451	Mus musculus phorbol-12-myristate-13-acetate-induced protein 1	1.308	4.005
Mal2	NM_178920	Mus musculus mal, T cell differentiation protein 2	1.308	4.005
Cd209f	NM_026956	Mus musculus CD209f antigen	4.245	4.001
Ckmt1	NM_009897	Mus musculus creatine kinase, mitochondrial 1, ubiquitous	2.606	3.993
Lrrcc1	NM_028915	Mus musculus leucine rich repeat and coiled-coil domain containing 1	3.195	3.990
Snhg11	NM_175692	Mus musculus small nucleolar RNA host gene 11	2.603	3.987
Itgb4	NM_001005608	Mus musculus integrin beta 4	3.193	3.986
Gpr162	NM_013533	Mus musculus G protein-coupled receptor 162	2.602	3.985
Alcam	NM_009655	Mus musculus activated leukocyte cell adhesion molecule	1.300	3.977
Pla2g2f	NM_012045	Mus musculus phospholipase A2, group IIF	1.296	3.961
C3	NM_009778	Mus musculus complement component 3	3.745	3.948
Aebp1	NM_001291857	Mus musculus AE binding protein 1	4.526	3.940
Bnc1	NM_007562	Mus musculus basonuclin 1	1.284	3.919
Sh3bp2	NM_011893	Mus musculus SH3-domain binding protein 2	1.280	3.904
Dmrtc1a	NM_027591	Mus musculus DMRT-like family C1a	1.279	3.899
Ngef	NM_001111314	Mus musculus neuronal guanine nucleotide exchange factor	2.559	3.896
Pilrb1	NM_133209	Mus musculus paired immunoglobin-like type 2 receptor beta 1	3.131	3.880
Ephx4	NM_001001804	Mus musculus epoxide hydrolase 4	2.549	3.877
Ccl19	NM_011888	Mus musculus chemokine ligand 19	3.125	3.871
Lgals7	NM_008496	Mus musculus lectin, galactose binding, soluble 7	1.270	3.867
Col3a1	NM_009930	Mus musculus collagen, type III, alpha 1	3.683	3.859

Pappa2	NM_001085376	Mus musculus pappalysin 2	1.267	3.856
Kif1a	NM_001294149	Mus musculus kinesin family member 1A	2.537	3.850
Ptpn22	NM_008979	Mus musculus protein tyrosine phosphatase, non-receptor type 22	1.264	3.848
Cthrc1	NM_026778	Mus musculus collagen triple helix repeat containing 1	1.261	3.837
Ramp1	NM_016894	Mus musculus receptor activity modifying protein 1	3.104	3.835
Tceal6	NM_025355	Mus musculus transcription elongation factor A -like 6	2.529	3.835
Bnc2	NM_172870	Mus musculus basonuclin 2	1.259	3.829
Dock3	NM_153413	Mus musculus dedicator of cyto-kinesis 3	1.257	3.822
Ptx3	NM_008987	Mus musculus pentraxin related gene	1.252	3.804
Il18	NM_008360	Mus musculus interleukin 18	2.510	3.796
Jchain	NM_152839	Mus musculus immunoglobulin joining chain	2.509	3.796
Sytl1	NM_031393	Mus musculus synaptotagmin-like 1	2.508	3.792
Ppp1r1b	NM_144828	Mus musculus protein phosphatase 1, regulatory subunit 1B	2.505	3.787
Inmt	NM_009349	Mus musculus indolethylamine N-methyltransferase	4.388	3.778
Zfp941	NM_001001180	Mus musculus zinc finger protein 941	1.241	3.766
B430306N03Rik	NM_177083	Mus musculus RIKEN cDNA B430306N03 gene	2.492	3.762
Foxf1	NM_010426	Mus musculus forkhead box F1	1.239	3.759
Gpx3	NM_008161	Mus musculus glutathione peroxidase 3	3.608	3.755
Pthlh	NM_008970	Mus musculus parathyroid hormone-like peptide	2.485	3.748
Ltbp2	NM_013589	Mus musculus latent transforming growth factor beta binding protein 2	4.029	3.729
Aldh1b1	NM_028270	Mus musculus aldehyde dehydrogenase 1 family, member B1	4.020	3.718
D19Bwg1357e	NM_177474	Mus musculus DNA segment, Chr 19, Brigham & Women's Genetics 1357 expressed	4.331	3.714
Stk32a	NM_178749	Mus musculus serine/threonine kinase 32A	1.225	3.712
Cntf	NM_170786	Mus musculus ciliary neurotrophic factor	3.026	3.708
Egr2	NM_010118	Mus musculus early growth response 2	3.026	3.708
Amica1	NM_001005421	Mus musculus adhesion molecule, interacts with CXADR antigen 1	2.464	3.706
Gap43	NM_008083	Mus musculus growth associated protein 43	1.218	3.686
Dclk1	NM_001111053	Mus musculus doublecortin-like kinase 1	3.007	3.677
Ptms	NM_026988	Mus musculus parathymosin	3.980	3.669
Tceal3	NM_001029978	Mus musculus transcription elongation factor A -like 3	2.445	3.669
Gm11837	NM_001243100	Mus musculus predicted gene 11837	2.994	3.658
Pdgfc	NM_019971	Mus musculus platelet-derived growth factor, C polypeptide	2.989	3.650
Vcan	NM_001081249	Mus musculus versican	2.982	3.638
Clec10a	NM_010796	Mus musculus C-type lectin domain family 10, member A	3.950	3.634
Megf6	NM_001162977	Mus musculus multiple EGF-like-domains 6	2.979	3.634
Prg4	NM_021400	Mus musculus proteoglycan 4	2.974	3.627
Tmeff2	NM_019790	Mus musculus transmembrane protein with EGF-like and two follistatin-like domains 2	1.198	3.620
Slc52a3	NM_027172	Mus musculus solute carrier protein family 52, member 3	2.412	3.607
Cpxm1	NM_019696	Mus musculus carboxypeptidase X 1	3.925	3.604
Scg5	NM_009162	Mus musculus secretogranin V	1.187	3.584
Lrrn2	NM_010732	Mus musculus leucine rich repeat protein 2, neuronal	2.947	3.584
Pdk3	NM_145630	Mus musculus pyruvate dehydrogenase kinase, isoenzyme 3	2.389	3.564
Wisp2	NM_016873	Mus musculus WNT1 inducible signaling pathway protein 2	3.878	3.549
Mgst2	NM_174995	Mus musculus microsomal glutathione S-transferase 2	1.174	3.542
Igfbp5	NM_010518	Mus musculus insulin-like growth factor binding protein 5	4.172	3.539
Cyct	NM_009989	Mus musculus cytochrome c, testis	1.169	3.523
Npr3	NM_008728	Mus musculus natriuretic peptide receptor 3	2.367	3.523
Rpap2	NM_001163462	Mus musculus RNA polymerase II associated protein 2	2.364	3.516
Apol9b	NM_001168660	Mus musculus apolipoprotein L 9b	2.359	3.508
Sh3rf3	NM_172788	Mus musculus SH3 domain containing ring finger 3	1.162	3.503
Gstt1	NM_008185	Mus musculus glutathione S-transferase, theta 1	3.822	3.485
Kiss1r	NM_053244	Mus musculus KISS1 receptor	1.156	3.484
S100a4	NM_011311	Mus musculus S100 calcium binding protein A4	4.104	3.466
Shd	NM_009168	Mus musculus src homology 2 domain-containing transforming protein D	2.336	3.465
Cdkn2a	NM_009877	Mus musculus cyclin-dependent kinase inhibitor 2A	1.148	3.458
Dock2	NM_033374	Mus musculus dedicator of cyto-kinesis 2	1.148	3.457
Col14a1	NM_181277	Mus musculus collagen, type XIV, alpha 1	2.331	3.456
Ear2	NM_007895	Mus musculus eosinophil-associated, ribonuclease A family, member 2	2.328	3.451
Axin2	NM_015732	Mus musculus axin2	1.144	3.444
Hoxb7	NM_010460	Mus musculus homeobox B7	1.144	3.443
Tgfbrap1	NM_001013025	Mus musculus transforming growth factor, beta receptor associated protein 1	1.143	3.441
Gpr158	NM_001004761	Mus musculus G protein-coupled receptor 158	2.320	3.437
Col2a1	NM_031163	Mus musculus collagen, type II, alpha 1	2.843	3.426
Lppr3	NM_181681	Mus musculus lipid phosphate phosphatase-related protein type 3	2.314	3.426
Chrdl1	NM_001114385	Mus musculus chordin-like 1	1.136	3.418
Arhgap36	NM_001081123	Mus musculus Rho GTPase activating protein 36	1.135	3.418
Olfr1039	NM_001011784	Mus musculus olfactory receptor 1039	1.134	3.414
Negr1	NM_001039094	Mus musculus neuronal growth regulator 1	1.132	3.406
Pdlim3	NM_016798	Mus musculus PDZ and LIM domain 3	3.752	3.406
LOC547349	NM_001025208	Mus musculus MHC class I family member	2.822	3.395
Slc17a9	NM_183161	Mus musculus solute carrier family 17, member 9	2.295	3.392
Ltb	NM_008518	Mus musculus lymphotoxin B	2.814	3.384
Fam167b	NM_182783	Mus musculus family with sequence similarity 167, member B	2.289	3.381
Syngr4	NM_001291064	Mus musculus synaptogyrin 4	1.123	3.379
Igfbp4	NM_010517	Mus musculus insulin-like growth factor binding protein 4	2.809	3.377
Bend6	NM_177235	Mus musculus BEN domain containing 6	2.281	3.367
Gli2	NM_001081125	Mus musculus GLI-Kruppel family member GLI2	1.119	3.366
F10	NM_007972	Mus musculus coagulation factor X	1.115	3.354
Prss23	NM_029614	Mus musculus protease, serine 23	3.703	3.352
Amhr2	NM_144547	Mus musculus anti-Mullerian hormone type 2 receptor	2.268	3.344
Slc24a2	NM_172426	Mus musculus solute carrier family 24 , member 2	1.109	3.336
Pde3b	NM_011055	Mus musculus phosphodiesterase 3B, cGMP-inhibited	2.777	3.330
Pam	NM_013626	Mus musculus peptidylglycine alpha-amidating monooxygenase	3.971	3.329
Ptprf	NM_011213	Mus musculus protein tyrosine phosphatase, receptor type, F	2.775	3.327
Grip1	NM_028736	Mus musculus glutamate receptor interacting protein 1	1.106	3.325
Fcho1	NM_028715	Mus musculus FCH domain only 1	1.105	3.324

Ifitm6	NM_001033632	Mus musculus interferon induced transmembrane protein 6	2.256	3.323
Grin2c	NM_010350	Mus musculus glutamate receptor, ionotropic, NMDA2C	2.772	3.322
Adamtsl1	NM_029967	Mus musculus ADAMTS-like 1	2.254	3.320
Galnt6	NM_001161767	Mus musculus UDP-N-acetyl-alpha-D-galactosamine:polypeptide N-acetylgalactosaminyltransferase 6	2.252	3.316
Slc2a6	NM_172659	Mus musculus solute carrier family 2 , member 6	1.099	3.306
Ptprc	NM_011210	Mus musculus protein tyrosine phosphatase, receptor type, C	1.098	3.301
Cd207	NM_144943	Mus musculus CD207 antigen	2.240	3.295
Clic3	NM_027085	Mus musculus chloride intracellular channel 3	2.239	3.292
Svep1	NM_022814	Mus musculus sushi, von Willebrand factor type A, EGF and pentraxin domain containing 1	3.639	3.283
Pak3	NM_001195049	Mus musculus p21 protein -activated kinase 3	1.088	3.271
Foxc1	NM_008592	Mus musculus forkhead box C1	2.734	3.268
Ttc9	NM_001033149	Mus musculus tetratricopeptide repeat domain 9	2.224	3.267
Olfml1	NM_172907	Mus musculus olfactomedin-like 1	2.732	3.267
Crtac1	NM_145123	Mus musculus cartilage acidic protein 1	1.086	3.266
Gng8	NM_010320	Mus musculus guanine nucleotide binding protein , gamma 8	2.221	3.262
Wscd2	NM_177292	Mus musculus WSC domain containing 2	2.217	3.254
2610524H06Rik	NM_181075	Mus musculus RIKEN cDNA 2610524H06 gene	2.210	3.244
Gnao1	NM_010308	Mus musculus guanine nucleotide binding protein, alpha O	3.600	3.242
Csf2rb2	NM_007781	Mus musculus colony stimulating factor 2 receptor, beta 2, low-affinity	2.208	3.239
Gpr45	NM_053107	Mus musculus G protein-coupled receptor 45	2.206	3.236
Scn2b	NM_001014761	Mus musculus sodium channel, voltage-gated, type II, beta	1.074	3.229
Ccdc146	NM_029195	Mus musculus coiled-coil domain containing 146	2.202	3.229
Postn	NM_015784	Mus musculus periostin, osteoblast specific factor	3.574	3.215
Vpreb3	NM_009514	Mus musculus pre-B lymphocyte gene 3	1.066	3.205
Vmn1r48	NM_053218	Mus musculus vomeronasal 1 receptor 48	1.065	3.201
Serpina3n	NM_009252	Mus musculus serine peptidase inhibitor, clade A, member 3N	2.685	3.200
Dnah6	NM_001164669	Mus musculus dynein, axonemal, heavy chain 6	1.063	3.197
Ust	NM_177387	Mus musculus uronyl-2-sulfotransferase	1.063	3.196
Chrna6	NM_021369	Mus musculus cholinergic receptor, nicotinic, alpha polypeptide 6	1.063	3.196
Vsig4	NM_177789	Mus musculus V-set and immunoglobulin domain containing 4	2.181	3.195
H2-DMb1	NM_010387	Mus musculus histocompatibility 2, class II, locus Mb1	2.680	3.194
Dync1i1	NM_010063	Mus musculus dynein cytoplasmic 1 intermediate chain 1	1.061	3.192
Slitrk6	NM_175499	Mus musculus SLIT and NTRK-like family, member 6	2.177	3.188
Irf6	NM_016851	Mus musculus interferon regulatory factor 6	2.176	3.186
Procr	NM_011171	Mus musculus protein C receptor, endothelial	2.673	3.183
Wnt9b	NM_011719	Mus musculus wingless-type MMTV integration site family, member 9B	2.173	3.181
Twist2	NM_007855	Mus musculus twist basic helix-loop-helix transcription factor 2	2.172	3.179
Macrod2	NM_001013802	Mus musculus MACRO domain containing 2	1.057	3.178
Ncald	NM_134094	Mus musculus neurocalcin delta	1.056	3.175
Col5a2	NM_007737	Mus musculus collagen, type V, alpha 2	2.663	3.170
Socs3	NM_007707	Mus musculus suppressor of cytokine signaling 3	3.531	3.169
Dlgap1	NM_177639	Mus musculus discs, large homolog-associated protein 1	2.162	3.163
Kcnn3	NM_080466	Mus musculus potassium intermediate/small conductance calcium-activated channel, subfamily N, member 3	1.044	3.142
Fam171b	NM_175514	Mus musculus family with sequence similarity 171, member B	2.147	3.138
Fjx1	NM_010218	Mus musculus four jointed box 1	1.041	3.132
Nat8l	NM_001001985	Mus musculus N-acetyltransferase 8-like	2.142	3.129
Psrc1	NM_019976	Mus musculus proline/serine-rich coiled-coil 1	1.039	3.127
Mmp9	NM_013599	Mus musculus matrix metallopeptidase 9	2.133	3.115
Fndc1	NM_001081416	Mus musculus fibronectin type III domain containing 1	2.128	3.107
Itgb7	NM_013566	Mus musculus integrin beta 7	2.127	3.104
Capn9	NM_023709	Mus musculus calpain 9	2.124	3.100
Iglon5	NM_001164518	Mus musculus IgLON family member 5	2.119	3.092
Fbln1	NM_010180	Mus musculus fibulin 1	3.726	3.091
Hsbp1l1	NM_001136181	Mus musculus heat shock factor binding protein 1-like 1	2.102	3.065
Susd5	NM_001101510	Mus musculus sushi domain containing 5	2.095	3.053
Thbs1	NM_011580	Mus musculus thrombospondin 1	2.575	3.052
Ccl11	NM_011330	Mus musculus chemokine ligand 11	2.570	3.046
Sprn	NM_183147	Mus musculus shadow of prion protein	2.087	3.041
Angpt1	NM_009640	Mus musculus angiopoietin 1	3.667	3.036
Ccdc74a	NM_001166164	Mus musculus coiled-coil domain containing 74A	1.006	3.034
Fgf7	NM_008008	Mus musculus fibroblast growth factor 7	2.083	3.033
Bmper	NM_028472	Mus musculus BMP-binding endothelial regulator	3.387	3.024
Slc16a4	NM_146136	Mus musculus solute carrier family 16 , member 4	1.002	3.024
Cr2	NM_007758	Mus musculus complement receptor 2	1.002	3.023
Spon1	NM_145584	Mus musculus spondin 1, extracellular matrix protein	2.074	3.020
Rab19	NM_011226	Mus musculus RAB19, member RAS oncogene family	2.067	3.009
Pcdh20	NM_178685	Mus musculus protocadherin 20	2.063	3.002
Greb1	NM_015764	Mus musculus gene regulated by estrogen in breast cancer protein	2.062	3.000
Ifit3	NM_010501	Mus musculus interferon-induced protein with tetratricopeptide repeats 3	2.533	2.996
Fkbp10	NM_010221	Mus musculus FK506 binding protein 10	3.616	2.989
Rab9b	NM_176971	Mus musculus RAB9B, member RAS oncogene family	2.054	2.988
Cldn15	NM_021719	Mus musculus claudin 15	2.525	2.987
Tex9	NM_009359	Mus musculus testis expressed gene 9	2.053	2.986
Esr1	NM_001302532	Mus musculus estrogen receptor 1	0.987	2.980
Efhd2	NM_025994	Mus musculus EF hand domain containing 2	3.342	2.980
Adgrg2	NM_178712	Mus musculus adhesion G protein-coupled receptor G2	2.045	2.974
Fam84a	NM_029007	Mus musculus family with sequence similarity 84, member A	0.983	2.969
Mfap4	NM_029568	Mus musculus microfibrillar-associated protein 4	2.508	2.965
Vsig8	NM_177723	Mus musculus V-set and immunoglobulin domain containing 8	0.980	2.962
Tpbg	NM_011627	Mus musculus trophoblast glycoprotein	0.980	2.961
Lilrb4	NM_001291894	Mus musculus leukocyte immunoglobulin-like receptor, subfamily B, member 4	3.320	2.959
Phgdh	NM_016966	Mus musculus 3-phosphoglycerate dehydrogenase	2.498	2.952
Zfp185	NM_009549	Mus musculus zinc finger protein 185	2.028	2.948
Diras2	NM_001024474	Mus musculus DIRAS family, GTP-binding RAS-like 2	2.027	2.946
Glrb	NM_010298	Mus musculus glycine receptor, beta subunit	0.974	2.945
Tspan5	NM_019571	Mus musculus tetraspanin 5	2.484	2.935

Cwc22	NM_030560	Mus musculus CWC22 spliceosome-associated protein homolog	2.484	2.934
Brsk1	NM_001003920	Mus musculus BR serine/threonine kinase 1	2.018	2.932
Tmprss6	NM_027902	Mus musculus transmembrane serine protease 6	0.967	2.926
Pcyox1l	NM_172832	Mus musculus prenylcysteine oxidase 1 like	0.967	2.925
Kcnj3	NM_008426	Mus musculus potassium inwardly-rectifying channel, subfamily J, member 3	3.280	2.920
Col4a3	NM_007734	Mus musculus collagen, type IV, alpha 3	2.008	2.916
Tchh	NM_001163098	Mus musculus trichohyalin	2.467	2.912
Zfp365	NM_178679	Mus musculus zinc finger protein 365	2.003	2.908
Itih5	NM_172471	Mus musculus inter-alpha inhibitor H5	2.457	2.900
Plcl1	NM_001114663	Mus musculus phospholipase C-like 1	2.456	2.899
Rbpjl	NM_009036	Mus musculus recombination signal binding protein for immunoglobulin kappa J region-like	0.953	2.889
Cdon	NM_021339	Mus musculus cell adhesion molecule-related/down-regulated by oncogenes	3.236	2.879
Dtnb	NM_007886	Mus musculus dystrobrevin, beta	1.979	2.873
Nr2f2	NM_009697	Mus musculus nuclear receptor subfamily 2, group F, member 2	3.228	2.872
Arhgef40	NM_198249	Mus musculus Rho guanine nucleotide exchange factor 40	0.940	2.856
Scml2	NM_001290651	Mus musculus sex comb on midleg-like 2	1.966	2.852
Il18r1	NM_008365	Mus musculus interleukin 18 receptor 1	1.959	2.843
Kalrn	NM_001164268	Mus musculus kalirin, RhoGEF kinase	0.934	2.840
D17H6S56E-5	NM_033075	Mus musculus DNA segment, Chr 17, human D6S56E 5	1.956	2.838
Pcdhb18	NM_053143	Mus musculus protocadherin beta 18	1.956	2.838
H60a	NM_010400	Mus musculus histocompatibility 60a	0.933	2.837
Pla2g4a	NM_008869	Mus musculus phospholipase A2, group IVA	2.406	2.837
Megf10	NM_001001979	Mus musculus multiple EGF-like-domains 10	1.950	2.829
Ndn	NM_010882	Mus musculus necdin	3.432	2.827
Slc2a10	NM_130451	Mus musculus solute carrier family 2 , member 10	1.946	2.823
Scara3	NM_172604	Mus musculus scavenger receptor class A, member 3	1.943	2.819
Eno4	NM_178689	Mus musculus enolase 4	0.925	2.815
Dtx1	NM_008052	Mus musculus deltex 1 homolog	1.938	2.812
Sox9	NM_011448	Mus musculus SRY -box 9	2.384	2.810
Sorcs2	NM_030889	Mus musculus sortilin-related VPS10 domain containing receptor 2	2.381	2.806
C1ra	NM_023143	Mus musculus complement component 1, r subcomponent A	3.153	2.803
Heph	NM_001159628	Mus musculus hephaestin	1.932	2.802
Sfrp2	NM_009144	Mus musculus secreted frizzled-related protein 2	2.378	2.802
Vwa5a	NM_172767	Mus musculus von Willebrand factor A domain containing 5A	0.919	2.799
Smpd3	NM_021491	Mus musculus sphingomyelin phosphodiesterase 3, neutral	1.928	2.796
Ntrk3	NM_182809	Mus musculus neurotrophic tyrosine kinase, receptor, type 3	0.917	2.795
Mgp	NM_008597	Mus musculus matrix Gla protein	2.797	2.795
Tro	NM_001002272	Mus musculus trophinin	1.921	2.786
Samsn1	NM_023380	Mus musculus SAM domain, SH3 domain and nuclear localization signals, 1	0.914	2.786
Pcgf6	NM_027654	Mus musculus polycomb group ring finger 6	0.912	2.783
Iqgap3	NM_001033484	Mus musculus IQ motif containing GTPase activating protein 3	0.911	2.781
Cd84	NM_001252472	Mus musculus CD84 antigen	0.910	2.778
Nr1h4	NM_001163504	Mus musculus nuclear receptor subfamily 1, group H, member 4	0.910	2.777
Aspg	NM_001081169	Mus musculus asparaginase homolog	1.911	2.771
Hoxd4	NM_010469	Mus musculus homeobox D4	1.909	2.768
Best1	NM_011913	Mus musculus bestrophin 1	0.906	2.766
Gpc3	NM_016697	Mus musculus glypican 3	2.336	2.752
Col6a3	NM_001243008	Mus musculus collagen, type VI, alpha 3	1.897	2.752
Actn3	NM_013456	Mus musculus actinin alpha 3	0.897	2.744
Begain	NM_001163175	Mus musculus brain-enriched guanylate kinase-associated	1.891	2.743
Ptgs1	NM_008969	Mus musculus prostaglandin-endoperoxide synthase 1	3.087	2.742
Dpysl5	NM_023047	Mus musculus dihydropyrimidinase-like 5	1.890	2.741
Drp2	NM_010078	Mus musculus dystrophin related protein 2	0.896	2.740
Wnt4	NM_009523	Mus musculus wingless-type MMTV integration site family, member 4	1.889	2.739
Fam49a	NM_029758	Mus musculus family with sequence similarity 49, member A	2.321	2.735
Kcna4	NM_021275	Mus musculus potassium voltage-gated channel, shaker-related subfamily, member 4	0.893	2.734
Ccdc80	NM_026439	Mus musculus coiled-coil domain containing 80	2.734	2.732
Pcdhb7	NM_053132	Mus musculus protocadherin beta 7	1.883	2.732
Rab15	NM_134050	Mus musculus RAB15, member RAS oncogene family	2.317	2.730
Shisa4	NM_175259	Mus musculus shisa family member 4	1.878	2.724
Ptpn7	NM_177081	Mus musculus protein tyrosine phosphatase, non-receptor type 7	0.887	2.720
Tspan8	NM_146010	Mus musculus tetraspanin 8	1.873	2.717
Nkd1	NM_027280	Mus musculus naked cuticle 1 homolog	1.872	2.716
C1s2	NM_173864	Mus musculus complement component 1, s subcomponent 2	3.057	2.716
Sct	NM_011328	Mus musculus secretin	1.871	2.714
Izumo4	NM_027829	Mus musculus IZUMO family member 4	1.868	2.709
Cyp4f18	NM_024444	Mus musculus cytochrome P450, family 4, subfamily f, polypeptide 18	1.866	2.707
Lgals3	NM_001145953	Mus musculus lectin, galactose binding, soluble 3	3.047	2.707
Ankrd42	NM_028665	Mus musculus ankyrin repeat domain 42	0.882	2.707
L3hypdh	NM_026038	Mus musculus L-3-hydroxyproline dehydratase	2.295	2.705
Fcgr1	NM_010186	Mus musculus Fc receptor, IgG, high affinity I	1.863	2.703
Gm5595	NM_001008427	Mus musculus predicted gene 5595	2.702	2.700
Soat1	NM_009230	Mus musculus sterol O-acyltransferase 1	0.879	2.699
Lck	NM_010693	Mus musculus lymphocyte protein tyrosine kinase	1.858	2.696
Col1a1	NM_007742	Mus musculus collagen, type I, alpha 1	3.274	2.695
Slc1a5	NM_009201	Mus musculus solute carrier family 1 , member 5	3.033	2.695
Rbp4	NM_011255	Mus musculus retinol binding protein 4, plasma	3.031	2.693
Cd248	NM_054042	Mus musculus CD248 antigen, endosialin	3.030	2.692
Wasf1	NM_031877	Mus musculus WAS protein family, member 1	1.852	2.688
Athl1	NM_145387	Mus musculus ATH1, acid trehalase-like 1	2.280	2.686179
Lgi4	NM_144556	Mus musculus leucine-rich repeat LGI family, member 4	3.020	2.683561
Tagln	NM_011526	Mus musculus transgelin	3.256	2.680263
Crabp1	NM_001284507	Mus musculus cellular retinoic acid binding protein I	0.871	2.679669
Glra1	NM_001290821	Mus musculus glycine receptor, alpha 1 subunit	0.871	2.679236
Treml4	NM_172623	Mus musculus triggering receptor expressed on myeloid cells-like 4	0.871	2.678474
Mrgprx2	NM_001034868	Mus musculus MAS-related GPR, member X2	0.871	2.677948

Krtap11-1	NM_001113406	Mus musculus keratin associated protein 11-1	0.870	2.675851
Tslp	NM_021367	Mus musculus thymic stromal lymphopoietin	0.869	2.674254
Ppm1n	NM_177691	Mus musculus protein phosphatase, Mg2+/Mn2+ dependent, 1N	0.868	2.672163
Ch25h	NM_009890	Mus musculus cholesterol 25-hydroxylase	1.841	2.671834
Kcnmb4	NM_021452	Mus musculus potassium large conductance calcium-activated channel, subfamily M, beta member 4	0.867	2.668968
Pnpla3	NM_054088	Mus musculus patatin-like phospholipase domain containing 3	1.838	2.666889
Fgl2	NM_008013	Mus musculus fibrinogen-like protein 2	2.996	2.662408
Star	NM_011485	Mus musculus steroidogenic acute regulatory protein	1.834	2.662339
Atp2b2	NM_009723	Mus musculus ATPase, Ca++ transporting, plasma membrane 2	2.256	2.658969
Mtap7d3	NM_177293	Mus musculus MAP7 domain containing 3	1.830	2.656320
Plekha4	NM_148927	Mus musculus pleckstrin homology domain containing, family A member 4	2.254	2.656082
Hoxb4	NM_010459	Mus musculus homeobox B4	1.826	2.650677
Ubxn10	NM_001285928	Mus musculus UBX domain protein 10	1.826	2.650432
Slc38a5	NM_172479	Mus musculus solute carrier family 38, member 5	2.977	2.645871
Lox	NM_010728	Mus musculus lysyl oxidase	2.244	2.644775
Cobl	NM_172496	Mus musculus cordon-bleu WH2 repeat	1.813	2.632849
Rgs6	NM_015812	Mus musculus regulator of G-protein signaling 6	1.813	2.632177
Ces2g	NM_197999	Mus musculus carboxylesterase 2G	1.813	2.632122
Mtmr6	NM_144843	Mus musculus myotubularin related protein 6	0.852	2.631400
Pak1	NM_011035	Mus musculus p21 protein -activated kinase 1	0.851	2.630309
Trf	NM_133977	Mus musculus transferrin	3.192	2.628775
Gucy1a3	NM_021896	Mus musculus guanylate cyclase 1, soluble, alpha 3	2.956	2.627806
Sirpb1b	NM_001173460	Mus musculus signal-regulatory protein beta 1B	1.809	2.626665
Kdm4a	NM_172382	Mus musculus lysine -specific demethylase 4A	1.805	2.622098
Dcaf12l2	NM_175539	Mus musculus DDB1 and CUL4 associated factor 12-like 2	3.183	2.621030
Ctsk	NM_007802	Mus musculus cathepsin K	2.222	2.620008
Calml4	NM_138304	Mus musculus calmodulin-like 4	1.801	2.616276
Flrt2	NM_201518	Mus musculus fibronectin leucine rich transmembrane protein 2	2.218	2.615761
Zfp772	NM_145577	Mus musculus zinc finger protein 772	0.845	2.614887
Wdr86	NM_001081441	Mus musculus WD repeat domain 86	0.843	2.609661
Gm10354	NM_001281514	Mus musculus predicted gene 10354	0.841	2.605748
B3galt1	NM_020283	Mus musculus UDP-Gal:betaGlcNAc beta 1,3-galactosyltransferase, polypeptide 1	0.840	2.604461
Tpsb2	NM_010781	Mus musculus tryptase beta 2	1.792	2.604376
Bend4	NM_001164806	Mus musculus BEN domain containing 4	2.207	2.603387
Qpct	NM_027455	Mus musculus glutaminyl-peptide cyclotransferase	2.206	2.602290
Ankrd1	NM_013468	Mus musculus ankyrin repeat domain 1	3.512	2.599186
Vpreb2	NM_016983	Mus musculus pre-B lymphocyte gene 2	0.837	2.597503
Cd5	NM_007650	Mus musculus CD5 antigen	0.837	2.596410
Fstl1	NM_008047	Mus musculus follistatin-like 1	3.147	2.592680
Ccdc136	NM_145574	Mus musculus coiled-coil domain containing 136	1.779	2.585999
Ackr1	NM_010045	Mus musculus atypical chemokine receptor 1	0.832	2.585284
Klra22	NM_053152	Mus musculus killer cell lectin-like receptor subfamily A, member 22	0.830	2.579163
Cytl1	NM_001081106	Mus musculus cytokine-like 1	2.897	2.578027
Ptprz1	NM_001081306	Mus musculus protein tyrosine phosphatase, receptor type Z, polypeptide 1	1.772	2.577138
Slc9a2	NM_001033289	Mus musculus solute carrier family 9 , member 2	1.769	2.572129
Tmem52b	NM_001081186	Mus musculus transmembrane protein 52B	1.768	2.571649
Cysltr1	NM_021476	Mus musculus cysteinyl leukotriene receptor 1	2.177	2.570169
Grin3a	NM_001276355	Mus musculus glutamate receptor ionotropic, NMDA3A	0.826	2.569099
Tmem254a	NM_026679	Mus musculus transmembrane protein 254a	1.766	2.568818
Slc27a3	NM_011988	Mus musculus solute carrier family 27 , member 3	2.175	2.567842
Aldh1a2	NM_009022	Mus musculus aldehyde dehydrogenase family 1, subfamily A2	2.172	2.564440
Igfbp6	NM_008344	Mus musculus insulin-like growth factor binding protein 6	3.108	2.562627
Cd300lh	NM_199201	Mus musculus CD300 antigen like family member H	0.822	2.561038
Clip4	NM_030179	Mus musculus CAP-GLY domain containing linker protein family, member 4	3.099	2.555458
Olfr63	NM_146937	Mus musculus olfactory receptor 63	0.819	2.553876
Tgfb3	NM_009368	Mus musculus transforming growth factor, beta 3	3.092	2.550172
Vasn	NM_139307	Mus musculus vasorin	2.863	2.549526
Nmrk2	NM_027120	Mus musculus nicotinamide riboside kinase 2	3.091	2.549066
Tram1l1	NM_146140	Mus musculus translocation associated membrane protein 1-like 1	1.748	2.543964
Eln	NM_007925	Mus musculus elastin	2.154	2.543814
Inhba	NM_008380	Mus musculus inhibin beta-A	2.147	2.536709
Zfp605	NM_001163996	Mus musculus zinc finger protein 605	1.742	2.536409
Chm	NM_018818	Mus musculus choroidermia	0.810	2.532370
Sertad4	NM_198247	Mus musculus SERTA domain containing 4	1.737	2.528902
Shisa2	NM_145463	Mus musculus shisa family member 2	1.732	2.522531
Csprs	NM_033616	Mus musculus component of Sp100-rs	2.133	2.521721
Prkag2	NM_145401	Mus musculus protein kinase, AMP-activated, gamma 2 non-catalytic subunit	3.054	2.520946
Mfap5	NM_015776	Mus musculus microfibrillar associated protein 5	3.051	2.518376
Cgref1	NM_026770	Mus musculus cell growth regulator with EF hand domain 1	2.129	2.517412
Pla2g5	NM_001122954	Mus musculus phospholipase A2, group V	2.819	2.513263
Tmem87a	NM_001110497	Mus musculus transmembrane protein 87A	0.801	2.511323
Ano7	NM_001271884	Mus musculus anoctamin 7	1.720	2.507528
Fgf2	NM_008006	Mus musculus fibroblast growth factor 2	0.799	2.506396
C1s1	NM_144938	Mus musculus complement component 1, s subcomponent 1	2.808	2.503781
Kcnh1	NM_010600	Mus musculus potassium voltage-gated channel, subfamily H , member 1	0.796	2.501236
Calr4	NM_001285898	Mus musculus calreticulin 4	0.795	2.497734
B4galnt1	NM_008080	Mus musculus beta-1,4-N-acetyl-galactosaminyl transferase 1	0.795	2.497044
Chd5	NM_001081376	Mus musculus chromodomain helicase DNA binding protein 5	1.712	2.496638
Gdf10	NM_145741	Mus musculus growth differentiation factor 10	2.105	2.490515
Thbs3	NM_013691	Mus musculus thrombospondin 3	2.788	2.488064
Clec2g	NM_027562	Mus musculus C-type lectin domain family 2, member g	0.790	2.485651
Cubn	NM_001081084	Mus musculus cubilin	1.700	2.480420
Col1a2	NM_007743	Mus musculus collagen, type I, alpha 2	2.467	2.479182
Sema3c	NM_013657	Mus musculus sema domain, immunoglobulin domain , short basic domain, secreted, 3C	2.777	2.478983
Pirt	NM_178656	Mus musculus phosphoinositide-interacting regulator of transient receptor potential channels	1.698	2.477921
Mdk	NM_010784	Mus musculus midkine	2.089	2.473640

Myh7	NM_080728	Mus musculus myosin, heavy polypeptide 7, cardiac muscle, beta	2.461	2.473602
Bank1	NM_001033350	Mus musculus B cell scaffold protein with ankyrin repeats 1	1.692	2.470870
Siglec1	NM_011426	Mus musculus sialic acid binding Ig-like lectin 1, sialoadhesin	1.691	2.469399
Elavl2	NM_207685	Mus musculus ELAV -like 2	0.782	2.467261
Unc13b	NM_001081413	Mus musculus unc-13 homolog B	2.762	2.467049
Sema3a	NM_009152	Mus musculus sema domain, immunoglobulin domain , short basic domain, secreted, 3A	1.688	2.465792
Srcin1	NM_018873	Mus musculus SRC kinase signaling inhibitor 1	0.780	2.464557
Fcmr	NM_026976	Mus musculus Fas apoptotic inhibitory molecule 3	1.682	2.458067
Tmem254c	NM_001270498	Mus musculus transmembrane protein 254c	2.073	2.457103
Adam8	NM_007403	Mus musculus a disintegrin and metallopeptidase domain 8	1.679	2.453338
Osmr	NM_011019	Mus musculus oncostatin M receptor	1.679	2.453050
Dhh	NM_007857	Mus musculus desert hedgehog	2.069	2.452853
Tpm2	NM_009416	Mus musculus tropomyosin 2, beta	2.069	2.452781
Cdo1	NM_033037	Mus musculus cysteine dioxygenase 1, cytosolic	1.674	2.446884
Slc5a3	NM_017391	Mus musculus solute carrier family 5 , member 3	1.674	2.446790
Igf1	NM_010512	Mus musculus insulin-like growth factor 1	2.736	2.445963
Mrvi1	NM_194464	Mus musculus MRV integration site 1	2.061	2.444113
Cyp1b1	NM_009994	Mus musculus cytochrome P450, family 1, subfamily b, polypeptide 1	2.060	2.442982
Pdpn	NM_010329	Mus musculus podoplanin	2.730	2.441438
Abcg4	NM_138955	Mus musculus ATP-binding cassette, sub-family G , member 4	1.668	2.439320
Bmx	NM_009759	Mus musculus BMX non-receptor tyrosine kinase	0.769	2.437922
Gimap3	NM_031247	Mus musculus GTPase, IMAP family member 3	1.665	2.435795
Lum	NM_008524	Mus musculus lumican	2.417	2.434572
Nat8	NM_023455	Mus musculus N-acetyltransferase 8	0.765	2.430466
Nfxl1	NM_133921	Mus musculus nuclear transcription factor, X-box binding-like 1	1.661	2.430097
Tbx5	NM_011537	Mus musculus T-box 5	2.714	2.428497
Cfh	NM_009888	Mus musculus complement component factor h	2.405	2.423717
Cstad	NM_030137	Mus musculus CSA-conditional, T cell activation-dependent protein	2.039	2.421231
Med23	NM_027347	Mus musculus mediator complex subunit 23	0.761	2.420658
Csrp2	NM_007792	Mus musculus cysteine and glycine-rich protein 2	2.918	2.418614
Bfsp1	NM_001291061	Mus musculus beaded filament structural protein 1, in lens-CP94	0.759	2.417517
Npl	NM_028749	Mus musculus N-acetylneuraminate pyruvate lyase	1.650	2.417054
Zfp600	NM_001177546	Mus musculus zinc finger protein 600	1.650	2.416179
Enpp2	NM_001136077	Mus musculus ectonucleotide pyrophosphatase/phosphodiesterase 2	2.697	2.414860
Spon2	NM_133903	Mus musculus spondin 2, extracellular matrix protein	2.695	2.413444
Maged2	NM_030700	Mus musculus melanoma antigen, family D, 2	2.910	2.412864
Col18a1	NM_009929	Mus musculus collagen, type XVIII, alpha 1	2.690	2.409259
Dzip1l	NM_028258	Mus musculus DAZ interacting protein 1-like	1.644	2.408270
Bex4	NM_212457	Mus musculus brain expressed gene 4	1.643	2.407606
Ucma	NM_026754	Mus musculus upper zone of growth plate and cartilage matrix associated	0.753	2.402735
Ccr2	NM_009915	Mus musculus chemokine receptor 2	1.636	2.399327
Gprc5b	NM_022420	Mus musculus G protein-coupled receptor, family C, group 5, member B	2.676	2.398371
Mmgt2	NM_175002	Mus musculus membrane magnesium transporter 2	2.017	2.398102
Rad51b	NM_009014	Mus musculus RAD51 homolog B	1.635	2.398084
Il17re	NM_145826	Mus musculus interleukin 17 receptor E	1.627	2.387517
Rhoh	NM_001081105	Mus musculus ras homolog gene family, member H	1.626	2.386036
Col16a1	NM_028266	Mus musculus collagen, type XVI, alpha 1	2.658	2.384731
Ccl6	NM_009139	Mus musculus chemokine ligand 6	2.657	2.383952
Nyap1	NM_175521	Mus musculus neuronal tyrosine-phosphorylated phosphoinositide 3-kinase adaptor 1	1.623	2.382977
Zfp503	NM_145459	Mus musculus zinc finger protein 503	1.623	2.382927
Dfna5	NM_018769	Mus musculus deafness, autosomal dominant 5	1.622	2.380796
Psat1	NM_001205339	Mus musculus phosphoserine aminotransferase 1	2.000	2.380794
Prkcb	NM_008855	Mus musculus protein kinase C, beta	1.998	2.377892
Kif5c	NM_008449	Mus musculus kinesin family member 5C	1.618	2.376387
Tlr5	NM_016928	Mus musculus toll-like receptor 5	1.617	2.375470
Mapk10	NM_009158	Mus musculus mitogen-activated protein kinase 10	2.858	2.375355
Tppp3	NM_026481	Mus musculus tubulin polymerization-promoting protein family member 3	2.857	2.374598
Ccdc163	NM_026714	Mus musculus coiled-coil domain containing 163	1.993	2.373491
Ripply2	NM_001037907	Mus musculus ripply2 homolog	1.614	2.371631
Ppl	NM_008909	Mus musculus periplakin	1.991	2.371071
Camkmt	NM_028576	Mus musculus calmodulin-lysine N-methyltransferase	0.738	2.370893
Serp2	NM_001160326	Mus musculus stress-associated endoplasmic reticulum protein family member 2	1.613	2.370254
Jazf1	NM_001168277	Mus musculus JAZF zinc finger 1	0.738	2.369928
Dpt	NM_019759	Mus musculus dermatopontin	2.633	2.365100
Tspan11	NM_026743	Mus musculus tetraspanin 11	1.609	2.364530
Col23a1	NM_153393	Mus musculus collagen, type XXIII, alpha 1	1.608	2.364322
Dpep2	NM_176913	Mus musculus dipeptidase 2	1.608	2.364165
Cadm2	NM_178721	Mus musculus cell adhesion molecule 2	0.734	2.362398
Il20rb	NM_001033543	Mus musculus interleukin 20 receptor beta	1.607	2.362113
Apol9a	NM_173786	Mus musculus apolipoprotein L 9a	1.602	2.356289
Il7r	NM_008372	Mus musculus interleukin 7 receptor	0.731	2.355256
Sult5a1	NM_020564	Mus musculus sulfotransferase family 5A, member 1	1.601	2.354809
Cd209g	NM_027343	Mus musculus CD209g antigen	1.975	2.354374
Ikzf1	NM_001025597	Mus musculus IKAROS family zinc finger 1	0.730	2.352840
Evpl	NM_025276	Mus musculus envoplakin	1.598	2.350741
Spta1	NM_011465	Mus musculus spectrin alpha, erythrocytic 1	0.729	2.350536
Cd209a	NM_133238	Mus musculus CD209a antigen	0.728	2.347685
Cd7	NM_009854	Mus musculus CD7 antigen	0.726	2.344316
Cd52	NM_013706	Mus musculus CD52 antigen	1.961	2.340189
Slc35f2	NM_028060	Mus musculus solute carrier family 35, member F2	0.724	2.338898
Ccl7	NM_013654	Mus musculus chemokine ligand 7	1.954	2.333123
Ptgfrn	NM_011197	Mus musculus prostaglandin F2 receptor negative regulator	2.586	2.329448
Acta2	NM_007392	Mus musculus actin, alpha 2, smooth muscle, aorta	1.950	2.329344
Cspg5	NM_001166273	Mus musculus chondroitin sulfate proteoglycan 5	0.718	2.327779
Snx29	NM_028964	Mus musculus sorting nexin 29	0.717	2.325912
Traf1	NM_009421	Mus musculus TNF receptor-associated factor 1	1.576	2.324289

Cish	NM_009895	Mus musculus cytokine inducible SH2-containing protein	2.781	2.320411
Timp2	NM_011594	Mus musculus tissue inhibitor of metalloproteinase 2	2.283	2.318115
Tmem2	NM_031997	Mus musculus transmembrane protein 2	1.936	2.315607
Gareml	NM_001167879	Mus musculus GRB2 associated, regulator of MAPK1-like	0.713	2.315600
Loxl3	NM_013586	Mus musculus lysyl oxidase-like 3	1.569	2.315414
AI661453	NM_145489	Mus musculus expressed sequence AI661453	1.933	2.312587
Clcn5	NM_001243762	Mus musculus chloride channel 5	0.711	2.312464
Aif1	NM_019467	Mus musculus allograft inflammatory factor 1	2.561	2.310327
Pacrg	NM_027032	Mus musculus PARK2 co-regulated	0.710	2.310228
A630001G21Rik	NM_177055	Mus musculus RIKEN cDNA A630001G21 gene	0.710	2.309779
Aplp1	NM_007467	Mus musculus amyloid beta precursor-like protein 1	1.929	2.308435
Sell	NM_001164059	Mus musculus selectin, lymphocyte	1.562	2.307790
Cd300a	NM_170758	Mus musculus CD300A antigen	0.709	2.307266
Dzip1	NM_025943	Mus musculus DAZ interacting protein 1	0.708	2.306595
Zmynd15	NM_001029929	Mus musculus zinc finger, MYND-type containing 15	0.708	2.304872
Pla2g2c	NM_008868	Mus musculus phospholipase A2, group IIC	1.558	2.302980
Kcnt2	NM_001081027	Mus musculus potassium channel, subfamily T, member 2	1.557	2.301262
Sqle	NM_009270	Mus musculus squalene epoxidase	1.556	2.300442
Sypl2	NM_008596	Mus musculus synaptophysin-like 2	0.703	2.295703
Nova1	NM_021361	Mus musculus neuro-oncological ventral antigen 1	1.916	2.295371
2200002D01Rik	NM_028179	Mus musculus RIKEN cDNA 2200002D01 gene	1.915	2.294818
Ache	NM_001290010	Mus musculus acetylcholinesterase	0.703	2.294605
Fam60a	NM_019643	Mus musculus family with sequence similarity 60, member A	0.702	2.293635
Dpy19l3	NM_178704	Mus musculus dpy-19-like 3	0.701	2.291903
Slc13a3	NM_054055	Mus musculus solute carrier family 13 , member 3	0.701	2.290014
Shroom2	NM_172441	Mus musculus shroom family member 2	0.699	2.286888
Ptgis	NM_008968	Mus musculus prostaglandin I2 synthase	1.907	2.286880
Gprasp2	NM_001163015	Mus musculus G protein-coupled receptor associated sorting protein 2	1.904	2.283617
Olfr482	NM_146733	Mus musculus olfactory receptor 482	0.696	2.281299
Adgrb2	NM_001290714	Mus musculus adhesion G protein-coupled receptor B2	1.901	2.280750
Apcdd1	NM_133237	Mus musculus adenomatosis polyposis coli down-regulated 1	1.539	2.279031
Atrnl1	NM_181415	Mus musculus attractin like 1	1.538	2.277962
Slc12a8	NM_134251	Mus musculus solute carrier family 12 , member 8	1.535	2.275282
Gm715	NM_001271548	Mus musculus predicted gene 715	1.535	2.275250
Erc2	NM_177814	Mus musculus ELKS/RAB6-interacting/CAST family member 2	0.693	2.274559
Rimkla	NM_177572	Mus musculus ribosomal modification protein rimK-like family member A	1.894	2.273647
Gpr182	NM_007412	Mus musculus G protein-coupled receptor 182	2.509	2.271765
Srgap3	NM_080448	Mus musculus SLIT-ROBO Rho GTPase activating protein 3	1.532	2.271419
Garnl3	NM_178888	Mus musculus GTPase activating RANGAP domain-like 3	1.890	2.269967
Slc1a7	NM_146255	Mus musculus solute carrier family 1 , member 7	1.530	2.268729
Scml4	NM_172938	Mus musculus sex comb on midleg-like 4	1.888	2.268418
Neil3	NM_146208	Mus musculus nei like 3	0.690	2.268064
Enpp6	NM_177304	Mus musculus ectonucleotide pyrophosphatase/phosphodiesterase 6	1.527	2.265075
Tfpi	NM_011576	Mus musculus tissue factor pathway inhibitor	2.499	2.264447
Il21r	NM_021887	Mus musculus interleukin 21 receptor	1.523	2.260081
Dlg4	NM_007864	Mus musculus discs, large homolog 4	1.521	2.257863
Synpo	NM_177340	Mus musculus synaptopodin	1.877	2.257467
Itih4	NM_018746	Mus musculus inter alpha-trypsin inhibitor, heavy chain 4	1.875	2.255341
Kmo	NM_133809	Mus musculus kynurenine 3-monooxygenase	1.517	2.252883
Fbxo27	NM_001163702	Mus musculus F-box protein 27	1.516	2.251831
Aqp4	NM_009700	Mus musculus aquaporin 4	1.515	2.251298
Gpr183	NM_183031	Mus musculus G protein-coupled receptor 183	1.515	2.251214
Scara5	NM_001168318	Mus musculus scavenger receptor class A, member 5	1.514	2.249580
Espn	NM_207687	Mus musculus espin	1.514	2.249419
Ralgps2	NM_001159965	Mus musculus Ral GEF with PH domain and SH3 binding motif 2	2.476	2.247483
H2-Eb1	NM_010382	Mus musculus histocompatibility 2, class II antigen E beta	2.673	2.245175
Fam167a	NM_177628	Mus musculus family with sequence similarity 167, member A	1.509	2.244476
Matn2	NM_016762	Mus musculus matrilin 2	2.472	2.244449
Pla2g4e	NM_177845	Mus musculus phospholipase A2, group IVE	0.679	2.244346
Degs2	NM_027299	Mus musculus degenerative spermatocyte homolog 2 , lipid desaturase	1.508	2.242371
Casp3	NM_001284409	Mus musculus caspase 3	0.677	2.241739
Rnasel	NM_011882	Mus musculus ribonuclease L	1.861	2.241289
Matk	NM_010768	Mus musculus megakaryocyte-associated tyrosine kinase	1.860	2.240599
Elovl6	NM_130450	Mus musculus ELOVL family member 6, elongation of long chain fatty acids	1.859	2.239650
Moxd1	NM_021509	Mus musculus monooxygenase, DBH-like 1	1.502	2.235449
Il33	NM_001164724	Mus musculus interleukin 33	0.674	2.234553
Tnni1	NM_021467	Mus musculus troponin I, skeletal, slow 1	1.849	2.229819
Sulf1	NM_172294	Mus musculus sulfatase 1	2.451	2.229138
Hrh1	NM_001252643	Mus musculus histamine receptor H1	1.494	2.226413
Ccr7	NM_007719	Mus musculus chemokine receptor 7	0.670	2.226034
Slc43a1	NM_001081349	Mus musculus solute carrier family 43, member 1	1.493	2.225039
Fcna	NM_007995	Mus musculus ficolin A	1.843	2.224328
Ugt1a6b	NM_201410	Mus musculus UDP glucuronosyltransferase 1 family, polypeptide A6B	1.843	2.223908
Tmod2	NM_001038710	Mus musculus tropomodulin 2	0.669	2.223445
Zmym3	NM_019831	Mus musculus zinc finger, MYM-type 3	0.668	2.222127
Adam33	NM_033615	Mus musculus a disintegrin and metallopeptidase domain 33	0.667	2.221211
Crybb2	NM_007773	Mus musculus crystallin, beta B2	0.667	2.221044
Vit	NM_028813	Mus musculus vitrin	1.840	2.220994
Fzd2	NM_020510	Mus musculus frizzled homolog 2	1.836	2.217894
Pi16	NM_023734	Mus musculus peptidase inhibitor 16	2.160	2.217157
Sowahc	NM_172939	Mus musculus sosondowah ankyrin repeat domain family member C	1.483	2.213753
Robo1	NM_019413	Mus musculus roundabout homolog 1	1.483	2.213098
Antxr1	NM_054041	Mus musculus anthrax toxin receptor 1	2.427	2.211908
Creb3l2	NM_178661	Mus musculus cAMP responsive element binding protein 3-like 2	1.829	2.210822
Cp	NM_007752	Mus musculus ceruloplasmin	2.620	2.209913
Serpina3g	NM_009251	Mus musculus serine peptidase inhibitor, clade A, member 3G	1.826	2.208150

Adgre1	NM_010130	Mus musculus adhesion G protein-coupled receptor E1	2.421	2.207336
Crlf1	NM_018827	Mus musculus cytokine receptor-like factor 1	1.824	2.205934
Cers6	NM_172856	Mus musculus ceramide synthase 6	0.659	2.203912
Shc4	NM_199022	Mus musculus SHC family, member 4	2.610	2.203256
Aldh1a3	NM_053080	Mus musculus aldehyde dehydrogenase family 1, subfamily A3	1.474	2.203002
Kcnab1	NM_010597	Mus musculus potassium voltage-gated channel, shaker-related subfamily, beta member 1	1.473	2.202239
Msr1	NM_031195	Mus musculus macrophage scavenger receptor 1	0.658	2.201878
Klhl13	NM_026167	Mus musculus kelch-like 13	2.413	2.201729
Kcne4	NM_021342	Mus musculus potassium voltage-gated channel, Isk-related subfamily, gene 4	0.657	2.199376
Gm13298	NM_001085530	Mus musculus predicted gene 13298	0.656	2.198518
Celf5	NM_176954	Mus musculus CUGBP, Elav-like family member 5	0.656	2.197884
Gmfg	NM_022024	Mus musculus glia maturation factor, gamma	2.407	2.197248
Itgbl1	NM_145467	Mus musculus integrin, beta-like 1	2.406	2.196422
Ptges	NM_022415	Mus musculus prostaglandin E synthase	1.468	2.196069
Rbms3	NM_001172123	Mus musculus RNA binding motif, single stranded interacting protein	2.404	2.195178
Smarca1	NM_053123	Mus musculus SWI/SNF related, matrix associated, actin dependent regulator of chromatin, subfamily a, member 1	1.464	2.190917
Islr	NM_012043	Mus musculus immunoglobulin superfamily containing leucine-rich repeat	2.397	2.190171
Ism1	NM_001276489	Mus musculus isthmin 1 homolog	1.462	2.188796
Cbln1	NM_019626	Mus musculus cerebellin 1 precursor protein	1.461	2.187860
Emb	NM_010330	Mus musculus embigin	1.460	2.186747
Fcrls	NM_030707	Mus musculus Fc receptor-like S, scavenger receptor	1.803	2.186459
Lrrn3	NM_010733	Mus musculus leucine rich repeat protein 3, neuronal	0.650	2.186108
Zfp677	NM_172486	Mus musculus zinc finger protein 677	1.458	2.184440
Cep112	NM_029606	Mus musculus centrosomal protein 112	1.458	2.184120
Clec4n	NM_020001	Mus musculus C-type lectin domain family 4, member n	1.799	2.182660
Pzp	NM_007376	Mus musculus pregnancy zone protein	0.648	2.182051
Ror2	NM_013846	Mus musculus receptor tyrosine kinase-like orphan receptor 2	1.455	2.180613
Myh9	NM_022410	Mus musculus myosin, heavy polypeptide 9, non-muscle	1.454	2.180309
Amigo2	NM_178114	Mus musculus adhesion molecule with Ig like domain 2	1.453	2.179345
Zc3hav1l	NM_172467	Mus musculus zinc finger CCCH-type, antiviral 1-like	1.453	2.179
Fabp7	NM_021272	Mus musculus fatty acid binding protein 7, brain	1.794	2.178
Pdgfrl	NM_026840	Mus musculus platelet-derived growth factor receptor-like	2.379	2.177
Rtn4rl1	NM_177708	Mus musculus reticulon 4 receptor-like 1	1.792	2.176
Opcml	NM_177906	Mus musculus opioid binding protein/cell adhesion molecule-like	0.645	2.175
Itch	NM_001243712	Mus musculus itchy, E3 ubiquitin protein ligase	0.645	2.175
Igsf10	NM_001162884	Mus musculus immunoglobulin superfamily, member 10	1.449	2.174
Fam114a1	NM_026667	Mus musculus family with sequence similarity 114, member A1	2.374	2.174
Gadd45g	NM_011817	Mus musculus growth arrest and DNA-damage-inducible 45 gamma	2.565	2.173
Uchl1	NM_011670	Mus musculus ubiquitin carboxy-terminal hydrolase L1	1.787	2.171
Efemp1	NM_146015	Mus musculus epidermal growth factor-containing fibulin-like extracellular matrix protein 1	2.560	2.170
Prr15	NM_030024	Mus musculus proline rich 15	1.781	2.166
BC022687	NM_145450	Mus musculus cDNA sequence BC022687	1.780	2.164
Btnl2	NM_079835	Mus musculus butyrophilin-like 2	1.440	2.164
Anpep	NM_008486	Mus musculus alanyl aminopeptidase	2.358	2.162
Pilra	NM_153510	Mus musculus paired immunoglobin-like type 2 receptor alpha	1.438	2.161
E2f1	NM_007891	Mus musculus E2F transcription factor 1	1.437	2.161
Pole2	NM_011133	Mus musculus polymerase , epsilon 2	0.637	2.159
Maoa	NM_173740	Mus musculus monoamine oxidase A	1.431	2.154
Cldn10	NM_021386	Mus musculus claudin 10	0.634	2.154
Tgfbi	NM_009369	Mus musculus transforming growth factor, beta induced	2.535	2.154
Cfp	NM_008823	Mus musculus complement factor properdin	1.768	2.153
D630045J12Rik	NM_194061	Mus musculus RIKEN cDNA D630045J12 gene	0.633	2.153
Foxo6	NM_194060	Mus musculus forkhead box O6	2.343	2.152
Hebp2	NM_019487	Mus musculus heme binding protein 2	1.426	2.148
Grhl1	NM_145890	Mus musculus grainyhead-like 1	0.631	2.148
Ntm	NM_172290	Mus musculus neurotrimin	0.631	2.147
H2-T22	NM_010397	Mus musculus histocompatibility 2, T region locus 22	1.760	2.146
Bace2	NM_019517	Mus musculus beta-site APP-cleaving enzyme 2	1.757	2.143
Maml3	NM_001004176	Mus musculus mastermind like 3	1.420	2.141
Nbl1	NM_008675	Mus musculus neuroblastoma, suppression of tumorigenicity 1	2.514	2.140
Tgfb2	NM_009367	Mus musculus transforming growth factor, beta 2	1.753	2.140
Opn4	NM_013887	Mus musculus opsin 4	1.418	2.140
Sbsn	NM_172205	Mus musculus suprabasin	1.417	2.138
Myof	NM_001302140	Mus musculus myoferlin	1.751	2.137
Gbp11	NM_001039647	Mus musculus guanylate binding protein 11	1.415	2.136
Phlda3	NM_013750	Mus musculus pleckstrin homology-like domain, family A, member 3	2.320	2.136
F13a1	NM_028784	Mus musculus coagulation factor XIII, A1 subunit	2.506	2.135
E330021D16Rik	NM_001201390	Mus musculus RIKEN cDNA E330021D16 gene	1.413	2.134
Clu	NM_013492	Mus musculus clusterin	2.790	2.134
Pigz	NM_172822	Mus musculus phosphatidylinositol glycan anchor biosynthesis, class Z	1.746	2.133
Dsn1	NM_025853	Mus musculus DSN1, MIND kinetochore complex component, homolog	1.411	2.132
Dpp4	NM_010074	Mus musculus dipeptidylpeptidase 4	1.410	2.130
Zfhx4	NM_030708	Mus musculus zinc finger homeodomain 4	0.622	2.130
Dok1	NM_010070	Mus musculus docking protein 1	1.409	2.129
Frzb	NM_011356	Mus musculus frizzled-related protein	1.739	2.127
Adgra3	NM_054044	Mus musculus adhesion G protein-coupled receptor A3	1.739	2.127
Rufy3	NM_001289776	Mus musculus RUN and FYVE domain containing 3	1.406	2.126
Camp	NM_009921	Mus musculus cathelicidin antimicrobial peptide	0.620	2.125
Phldb3	NM_001102613	Mus musculus pleckstrin homology-like domain, family B, member 3	1.405	2.124
Hspa1l	NM_013558	Mus musculus heat shock protein 1-like	1.404	2.123
Tspan32	NM_020286	Mus musculus tetraspanin 32	1.403	2.123
Magi1	NM_001029850	Mus musculus membrane associated guanylate kinase, WW and PDZ domain containing 1	0.618	2.122
Myo1g	NM_178440	Mus musculus myosin IG	1.734	2.122
Nme7	NM_138314	Mus musculus NME/NM23 family member 7	1.400	2.119
Socs1	NM_009896	Mus musculus suppressor of cytokine signaling 1	1.731	2.119
Aldh1a7	NM_011921	Mus musculus aldehyde dehydrogenase family 1, subfamily A7	0.615	2.117

Rfc5	NM_028128	Mus musculus replication factor C 5	1.726	2.115
Scrn1	NM_027268	Mus musculus secernin 1	2.283	2.110
Apobr	NM_138310	Mus musculus apolipoprotein B receptor	1.391	2.109
Gm10228	NM_001270487	Mus musculus predicted gene 10228	0.610	2.107
Aldh3b1	NM_026316	Mus musculus aldehyde dehydrogenase 3 family, member B1	1.718	2.107
Brca1	NM_009764	Mus musculus breast cancer 1	0.609	2.106
Has1	NM_008215	Mus musculus hyaluronan synthase1	0.609	2.105
Nalcn	NM_177393	Mus musculus sodium leak channel, non-selective	0.609	2.105
Thumpd1	NM_145585	Mus musculus THUMP domain containing 1	2.275	2.105
Rs1	NM_011302	Mus musculus retinoschisis 1	2.458	2.104
Socs2	NM_007706	Mus musculus suppressor of cytokine signaling 2	1.714	2.104
Mst1r	NM_009074	Mus musculus macrophage stimulating 1 receptor	1.713	2.103
Aldoc	NM_009657	Mus musculus aldolase C, fructose-bisphosphate	1.385	2.102
Tekt2	NM_011902	Mus musculus tektin 2	1.385	2.102
Rhou	NM_133955	Mus musculus ras homolog gene family, member U	1.712	2.102
Klhl4	NM_172781	Mus musculus kelch-like 4	1.384	2.101
Lyve1	NM_053247	Mus musculus lymphatic vessel endothelial hyaluronan receptor 1	2.269	2.101
Rbm3	NM_001166409	Mus musculus RNA binding motif protein 3	2.451	2.099
Nefm	NM_008691	Mus musculus neurofilament, medium polypeptide	1.382	2.099
Zfp808	NM_001039239	Mus musculus zinc finger protein 80	0.606	2.099
BC026585	NM_001033284	Mus musculus cDNA sequence BC026585	1.382	2.099
Hoxd8	NM_008276	Mus musculus homeobox D8	1.382	2.099
Epha1	NM_023580	Mus musculus Eph receptor A1	0.606	2.098
Gpnmb	NM_053110	Mus musculus glycoprotein nmb	0.605	2.098
Acsl6	NM_001033599	Mus musculus acyl-CoA synthetase long-chain family member 6	1.704	2.095
Tceal8	NM_025703	Mus musculus transcription elongation factor A -like 8	1.377	2.094
C77370	NM_001077354	Mus musculus expressed sequence C77370	0.603	2.094
Cd300lf	NM_001169153	Mus musculus CD300 antigen like family member F	2.433	2.088
Adamts15	NM_001024139	Mus musculus a disintegrin-like and metallopeptidase with thrombospondin type 1 motif, 15	2.250	2.087
Pcolce	NM_008788	Mus musculus procollagen C-endopeptidase enhancer protein	1.993	2.087
Lef1	NM_010703	Mus musculus lymphoid enhancer binding factor 1	0.599	2.086
Gpx2	NM_030677	Mus musculus glutathione peroxidase 2	0.599	2.085
Zfp455	NM_001048204	Mus musculus zinc finger protein 455	0.598	2.084
Dhrs7	NM_025522	Mus musculus dehydrogenase/reductase member 7	1.986	2.081
4930503L19Rik	NM_172967	Mus musculus RIKEN cDNA 4930503L19 gene	0.596	2.080
Prkar2b	NM_011158	Mus musculus protein kinase, cAMP dependent regulatory, type II beta	1.688	2.080
Clec2i	NM_020257	Mus musculus C-type lectin domain family 2, member i	0.596	2.079
Akr1c13	NM_013778	Mus musculus aldo-keto reductase family 1, member C13	1.685	2.077
Lsp1	NM_019391	Mus musculus lymphocyte specific 1	1.684	2.076
Cgnl1	NM_001304362	Mus musculus cingulin-like 1	2.410	2.073
Fam69a	NM_026062	Mus musculus family with sequence similarity 69, member A	1.356	2.071
Timp3	NM_011595	Mus musculus tissue inhibitor of metalloproteinase 3	2.404	2.069
Armc3	NM_001081083	Mus musculus armadillo repeat containing 3	0.590	2.069
Egfr	NM_207655	Mus musculus epidermal growth factor receptor	1.675	2.069
Nkd2	NM_028186	Mus musculus naked cuticle 2 homolog	1.353	2.067
Tbrg1	NM_025289	Mus musculus transforming growth factor beta regulated gene 1	2.398	2.066
Rasgef1a	NM_027526	Mus musculus RasGEF domain family, member 1A	0.588	2.065
Syn1	NM_013680	Mus musculus synapsin I	1.350	2.064
Lrrc4c	NM_178725	Mus musculus leucine rich repeat containing 4C	0.588	2.064
Nacad	NM_001081652	Mus musculus NAC alpha domain containing	1.349	2.063
Trim35	NM_029979	Mus musculus tripartite motif-containing 35	0.587	2.063
Tmem100	NM_026433	Mus musculus transmembrane protein 100	1.345	2.059
Bivm	NM_144558	Mus musculus basic, immunoglobulin-like variable motif containing	0.585	2.059
Fgf5	NM_010203	Mus musculus fibroblast growth factor 5	0.584	2.057
Efemp2	NM_021474	Mus musculus epidermal growth factor-containing fibulin-like extracellular matrix protein 2	2.204	2.056
Trps1	NM_032000	Mus musculus trichorhinophalangeal syndrome I	1.655	2.050
Chp2	NM_027363	Mus musculus calcineurin-like EF hand protein 2	1.337	2.050
Vim	NM_011701	Mus musculus vimentin	1.943	2.048
Flywch2	NM_029798	Mus musculus FLYWCH family member 2	1.652	2.048
Il17ra	NM_008359	Mus musculus interleukin 17 receptor A	0.579	2.048
Lppr4	NM_177664	Mus musculus lipid phosphate phosphatase-related protein type 4	0.578	2.046
Timd4	NM_178759	Mus musculus T cell immunoglobulin and mucin domain containing 4	1.333	2.045
S100b	NM_009115	Mus musculus S100 protein, beta polypeptide, neural	1.649	2.045
Nrk	NM_013724	Mus musculus Nik related kinase	0.578	2.045
Trim59	NM_025863	Mus musculus tripartite motif-containing 59	1.330	2.043
Rac2	NM_009008	Mus musculus RAS-related C3 botulinum substrate 2	1.644	2.041
Cmah	NM_007717	Mus musculus cytidine monophospho-N-acetylneuraminic acid hydroxylase	0.575	2.040
Slc22a17	NM_021551	Mus musculus solute carrier family 22 , member 17	1.642	2.040
Plekhb1	NM_013746	Mus musculus pleckstrin homology domain containing, family B member 1	1.642	2.039
Nr2f1	NM_010151	Mus musculus nuclear receptor subfamily 2, group F, member 1	2.178	2.039
Apbb1ip	NM_019456	Mus musculus amyloid beta precursor protein-binding, family B, member 1 interacting protein	1.641	2.038
Tifa	NM_145133	Mus musculus TRAF-interacting protein with forkhead-associated domain	1.325	2.037
Cacnb3	NM_007581	Mus musculus calcium channel, voltage-dependent, beta 3 subunit	1.322	2.034
Dok2	NM_010071	Mus musculus docking protein 2	2.169	2.033
Ddx41	NM_134059	Mus musculus DEAD box polypeptide 41	0.572	2.033
Crip1	NM_007763	Mus musculus cysteine-rich protein 1	1.923	2.033
Laptm5	NM_010686	Mus musculus lysosomal-associated protein transmembrane 5	1.635	2.033
Cyth4	NM_028195	Mus musculus cytohesin 4	2.167	2.032
Susd3	NM_025491	Mus musculus sushi domain containing 3	1.320	2.032
Ddit4l	NM_030143	Mus musculus DNA-damage-inducible transcript 4-like	1.633	2.031
Pnp2	NM_001123371	Mus musculus purine-nucleoside phosphorylase 2	1.319	2.031
AI467606	NM_178901	Mus musculus expressed sequence AI467606	1.319	2.030
Map1b	NM_008634	Mus musculus microtubule-associated protein 1B	1.630	2.029
Cacna1g	NM_009783	Mus musculus calcium channel, voltage-dependent, T type, alpha 1G subunit	1.629	2.027
Nnmt	NM_010924	Mus musculus nicotinamide N-methyltransferase	1.315	2.027
Aif1l	NM_145144	Mus musculus allograft inflammatory factor 1-like	1.627	2.026

Scg3	NM_009130	Mus musculus secretogranin III	0.568	2.026
Maf	NM_001025577	Mus musculus avian musculoaponeurotic fibrosarcoma AS42 oncogene homolog	2.158	2.026
Fam109b	NM_177391	Mus musculus family with sequence similarity 109, member B	1.314	2.026
Lrp1	NM_008512	Mus musculus low density lipoprotein receptor-related protein 1	2.328	2.023
Nxnl2	NM_029173	Mus musculus nucleoredoxin-like 2	0.565	2.021
Mrc2	NM_008626	Mus musculus mannose receptor, C type 2	1.621	2.021
Ccl9	NM_011338	Mus musculus chemokine ligand 9	2.149	2.020
Cenpk	NM_021790	Mus musculus centromere protein K	0.564	2.020
4930564C03Rik	NM_029257	Mus musculus RIKEN cDNA 4930564C03 gene	0.564	2.019
Prl2c1	NM_001045532	Mus musculus Prolactin family 2, subfamily c, member 1	1.619	2.019
Dusp6	NM_026268	Mus musculus dual specificity phosphatase 6	2.144	2.017
Gria3	NM_016886	Mus musculus glutamate receptor, ionotropic, AMPA3	0.562	2.017
Plekhg3	NM_153804	Mus musculus pleckstrin homology domain containing, family G member 3	1.305	2.016
Il1rap	NM_008364	Mus musculus interleukin 1 receptor accessory protein	1.615	2.016
Padi3	NM_011060	Mus musculus peptidyl arginine deiminase, type III	0.561	2.013
Ddc	NM_001190448	Mus musculus dopa decarboxylase	1.612	2.013
S100a6	NM_011313	Mus musculus S100 calcium binding protein A6	1.894	2.013
Nap1l5	NM_021432	Mus musculus nucleosome assembly protein 1-like 5	1.301	2.012
Fam46b	NM_175307	Mus musculus family with sequence similarity 46, member B	1.611	2.012
Thsd4	NM_001040426	Mus musculus thrombospondin, type I, domain containing 4	1.608	2.010
Id4	NM_031166	Mus musculus inhibitor of DNA binding 4	0.559	2.009
Scn3b	NM_178227	Mus musculus sodium channel, voltage-gated, type III, beta	1.608	2.009
Slc25a17	NM_011399	Mus musculus solute carrier family 25 , member 17	2.132	2.009
Klc3	NM_146182	Mus musculus kinesin light chain 3	1.297	2.008
Krt85	NM_016879	Mus musculus keratin 85	1.605	2.007
Panx1	NM_019482	Mus musculus pannexin 1	1.604	2.006
Basp1	NM_027395	Mus musculus brain abundant, membrane attached signal protein 1	1.604	2.006
Nupr1	NM_019738	Mus musculus nuclear protein transcription regulator 1	2.127	2.005
Rab7b	NM_145509	Mus musculus RIKEN cDNA 5430435G22 gene	2.126	2.005
Rph3al	NM_001291159	Mus musculus rabphilin 3A-like	0.555	2.004
Lrrc75a	NM_198861	Mus musculus leucine rich repeat containing 75A	2.124	2.003
Gm1987	NM_001193667	Mus musculus predicted gene 1987	1.881	2.003
Mdga1	NM_001081160	Mus musculus MAM domain containing glycosylphosphatidylinositol anchor 1	1.596	1.999
4930427A07Rik	NM_134041	Mus musculus RIKEN cDNA 4930427A07 gene	1.288	1.998
Trim2	NM_001271727	Mus musculus tripartite motif-containing 2	2.112	1.996
Zfp869	NM_001039967	Mus musculus zinc finger protein 869	1.285	1.995
Fcgr2b	NM_001077189	Mus musculus Fc receptor, IgG, low affinity IIb	1.589	1.993
Mrc1	NM_008625	Mus musculus mannose receptor, C type 1	2.108	1.993
Ptpru	NM_001083119	Mus musculus protein tyrosine phosphatase, receptor type, U	1.588	1.992
Panx2	NM_001002005	Mus musculus pannexin 2	0.548	1.990
Slc29a4	NM_146257	Mus musculus solute carrier family 29 , member 4	0.547	1.989
Lcp1	NM_008879	Mus musculus lymphocyte cytosolic protein 1	1.279	1.988
Srsf9	NM_025573	Mus musculus serine/arginine-rich splicing factor 9	2.270	1.987
Lrrc25	NM_153074	Mus musculus leucine rich repeat containing 25	1.276	1.985
Lhfpl2	NM_172589	Mus musculus lipoma HMGIC fusion partner-like 2	1.274	1.983
Mecom	NM_021442	Mus musculus MDS1 and EVI1 complex locus	0.544	1.983
Nbea	NM_030595	Mus musculus neurobeachin	0.544	1.982
Vcam1	NM_011693	Mus musculus vascular cell adhesion molecule 1	1.271	1.981
Kcna1	NM_010595	Mus musculus potassium voltage-gated channel, shaker-related subfamily, member 1	1.571	1.978
Adm	NM_009627	Mus musculus adrenomedullin	1.571	1.978
Cercam	NM_207298	Mus musculus cerebral endothelial cell adhesion molecule	0.541	1.977
Scin	NM_009132	Mus musculus scinderin	1.267	1.976
Cd5l	NM_009690	Mus musculus CD5 antigen-like	1.567	1.975
Ifi202b	NM_008327	Mus musculus interferon activated gene 202B	1.567	1.974
Bst2	NM_198095	Mus musculus bone marrow stromal cell antigen 2	2.078	1.974
Dock10	NM_001285927	Mus musculus dedicator of cytokinesis 10	1.265	1.974
Ccl21a	NM_011124	Mus musculus chemokine ligand 21A	2.245	1.972
Lzts3	NM_197945	Mus musculus leucine zipper, putative tumor suppressor family member 3	2.245	1.972
Lad1	NM_133664	Mus musculus ladinin	2.072	1.970
B3galnt1	NM_020026	Mus musculus UDP-GalNAc:betaGlcNAc beta 1,3-galactosaminyltransferase, polypeptide 1	1.562	1.970
2010300C02Rik	NM_028096	Mus musculus RIKEN cDNA 2010300C02 gene	1.260	1.969
C1ql3	NM_153155	Mus musculus C1q-like 3	0.536	1.969
Rabgap1l	NM_013862	Mus musculus RAB GTPase activating protein 1-like	2.068	1.967
Tnni2	NM_009405	Mus musculus troponin I, skeletal, fast 2	1.557	1.966
Cotl1	NM_028071	Mus musculus coactosin-like 1	2.234	1.965
Ager	NM_001271424	Mus musculus advanced glycosylation end product-specific receptor	0.534	1.965
Cchcr1	NM_146248	Mus musculus coiled-coil alpha-helical rod protein 1	1.256	1.965
Dsel	NM_001081316	Mus musculus dermatan sulfate epimerase-like	1.554	1.963
Tuba1b	NM_011654	Mus musculus tubulin, alpha 1B	2.483	1.961
Greb1l	NM_001083628	Mus musculus growth regulation by estrogen in breast cancer-like	1.252	1.960
Angptl4	NM_020581	Mus musculus angiopoietin-like 4	2.055	1.959
Has2	NM_008216	Mus musculus hyaluronan synthase 2	1.250	1.959
Btg1	NM_007569	Mus musculus B cell translocation gene 1, anti-proliferative	0.530	1.958
Rpl3	NM_013762	Mus musculus ribosomal protein L3	1.818	1.957
Scn2a1	NM_001099298	Mus musculus sodium channel, voltage-gated, type II, alpha 1	0.530	1.956
Loxl1	NM_010729	Mus musculus lysyl oxidase-like 1	2.219	1.956
Figf	NM_010216	Mus musculus c-fos induced growth factor	1.546	1.956
Eef1a1	NM_010106	Mus musculus eukaryotic translation elongation factor 1 alpha 1	1.815	1.956
Gpm6b	NM_001177956	Mus musculus glycoprotein m6b	2.210	1.951
Pou4f1	NM_011143	Mus musculus POU domain, class 4	2.210	1.951
Batf	NM_016767	Mus musculus basic leucine zipper transcription factor, ATF-like	1.239	1.947
Fgf14	NM_010201	Mus musculus fibroblast growth factor 14	0.524	1.946
Nid2	NM_008695	Mus musculus nidogen 2	1.534	1.946
Kazn	NM_001109685	Mus musculus kazrin, periplakin interacting protein	1.531	1.944
Pms1	NM_153556	Mus musculus postmeiotic segregation increased 1	1.236	1.944
Sfrp1	NM_013834	Mus musculus secreted frizzled-related protein 1	1.236	1.944

Golm1	NM_027307	Mus musculus golgi membrane protein 1	2.030	1.943
Plxna3	NM_008883	Mus musculus plexin A3	1.232	1.940
Ucp2	NM_011671	Mus musculus uncoupling protein 2	1.526	1.939
Rbp1	NM_011254	Mus musculus retinol binding protein 1, cellular	2.189	1.939
Stxbp6	NM_144552	Mus musculus syntaxin binding protein 6	1.525	1.938
Rab3il1	NM_144538	Mus musculus RAB3A interacting protein -like 1	2.021	1.938
Polm	NM_017401	Mus musculus polymerase , mu	1.229	1.937
Clec2l	NM_001101507	Mus musculus C-type lectin domain family 2, member L	1.229	1.936
Gm7609	NM_001081746	Mus musculus predicted pseudogene 7609	0.518	1.936
Dbn1	NM_001177371	Mus musculus drebrin 1	1.521	1.935
BC030499	NM_001287206	Mus musculus cDNA sequence BC030499	0.517	1.934
Rgs16	NM_011267	Mus musculus regulator of G-protein signaling 16	1.519	1.934
Tmem35	NM_026239	Mus musculus transmembrane protein 35	1.518	1.933
Nsg1	NM_010942	Mus musculus neuron specific gene family member 1	2.014	1.933
Rassf2	NM_175445	Mus musculus Ras association domain family member 2	1.518	1.933
Trnp1	NM_001081156	Mus musculus TMF1-regulated nuclear protein 1	2.013	1.932
Col8a1	NM_007739	Mus musculus collagen, type VIII, alpha 1	2.178	1.932
Pxdc1	NM_025831	Mus musculus PX domain containing 1	2.012	1.932
Osr1	NM_011859	Mus musculus odd-skipped related 1	1.515	1.930
Synpo2l	NM_175132	Mus musculus synaptopodin 2-like	1.514	1.929
Sash3	NM_028773	Mus musculus SAM and SH3 domain containing 3	1.513	1.929
Gas6	NM_019521	Mus musculus growth arrest specific 6	1.775	1.927
Itih2	NM_010582	Mus musculus inter-alpha trypsin inhibitor, heavy chain 2	0.512	1.925
Fgfr1	NM_010206	Mus musculus fibroblast growth factor receptor 1	2.166	1.925
Loxl4	NM_001164311	Mus musculus lysyl oxidase-like 4	0.512	1.925
Rabgap1	NM_001033960	Mus musculus RAB GTPase activating protein 1	2.000	1.924
Gm5592	NM_001033782	Mus musculus predicted gene 5592	1.215	1.923
Tlr6	NM_011604	Mus musculus toll-like receptor 6	1.215	1.923
Vav1	NM_011691	Mus musculus vav 1 oncogene	1.506	1.923
Tnk2	NM_016788	Mus musculus tyrosine kinase, non-receptor, 2	2.161	1.923
Tmem45a	NM_019631	Mus musculus transmembrane protein 45a	1.505	1.922
Gldc	NM_138595	Mus musculus glycine decarboxylase	1.213	1.921
Klhdc8b	NM_030075	Mus musculus kelch domain containing 8B	1.213	1.921
Serpinf1	NM_011340	Mus musculus serine peptidase inhibitor, clade F, member 1	1.502	1.920
Fibin	NM_026271	Mus musculus fin bud initiation factor homolog	1.992	1.919
Apod	NM_007470	Mus musculus apolipoprotein D	2.154	1.918
Efhd1	NM_028889	Mus musculus EF hand domain containing 1	1.498	1.916
Cpt1c	NM_153679	Mus musculus carnitine palmitoyltransferase 1c	1.497	1.915
Hoxa3	NM_010452	Mus musculus homeobox A3	1.207	1.915
Tns3	NM_001083587	Mus musculus tensin 3	1.207	1.915
Serpine2	NM_009255	Mus musculus serine peptidase inhibitor, clade E, member 2	2.147	1.914
Gstm6	NM_008184	Mus musculus glutathione S-transferase, mu 6	1.496	1.914
Fam198b	NM_133187	Mus musculus family with sequence similarity 198, member B	2.145	1.913
Anxa9	NM_001085383	Mus musculus annexin A9	1.203	1.911
St6galnac1	NM_011371	Mus musculus ST6 -N-acetylgalactosaminide alpha-2,6-sialyltransferase 1	1.977	1.910
Apoe	NM_009696	Mus musculus apolipoprotein E	2.385	1.910
Bok	NM_016778	Mus musculus BCL2-related ovarian killer	1.488	1.908
Mgat1	NM_010794	Mus musculus mannoside acetylglucosaminyltransferase 1	0.502	1.908
Plxnc1	NM_018797	Mus musculus plexin C1	1.486	1.906
Fbn1	NM_007993	Mus musculus fibrillin 1	2.132	1.906
Cacna2d3	NM_009785	Mus musculus calcium channel, voltage-dependent, alpha2/delta subunit 3	1.196	1.903
Nfatc1	NM_198429	Mus musculus nuclear factor of activated T cells, cytoplasmic, calcineurin dependent 1	1.965	1.903
Omd	NM_012050	Mus musculus osteomodulin	1.481	1.902
Sdc1	NM_011519	Mus musculus syndecan 1	1.193	1.901
Chit1	NM_027979	Mus musculus chitinase 1	1.192	1.900
Bmp6	NM_007556	Mus musculus bone morphogenetic protein 6	1.477	1.899
Vps13b	NM_177151	Mus musculus vacuolar protein sorting 13B	0.497	1.898
Stt3a	NM_008408	Mus musculus STT3, subunit of the oligosaccharyltransferase complex, homolog A	0.496	1.898
Smo	NM_176996	Mus musculus smoothened homolog	1.956	1.897
Ssx2ip	NM_001253768	Mus musculus synovial sarcoma, X breakpoint 2 interacting protein	0.496	1.897
Rarres2	NM_027852	Mus musculus retinoic acid receptor responder 2	2.117	1.897
Adam11	NM_001110778	Mus musculus a disintegrin and metallopeptidase domain 11	1.474	1.897
Arhgef25	NM_028027	Mus musculus Rho guanine nucleotide exchange factor 25	2.116	1.896
Cd200	NM_010818	Mus musculus CD200 antigen	1.955	1.896
Abcc8	NM_011510	Mus musculus ATP-binding cassette, sub-family C , member 8	2.112	1.894
Elovl7	NM_029001	Mus musculus ELOVL family member 7, elongation of long chain fatty acids	1.183	1.891
Hmha1	NM_001142701	Mus musculus histocompatibility HA-1	1.944	1.889
Cabp5	NM_013877	Mus musculus calcium binding protein 5	2.103	1.889
Nrros	NM_146069	Mus musculus negative regulator of reactive oxygen species	1.942	1.888
Kcnn4	NM_001163510	Mus musculus potassium intermediate/small conductance calcium-activated channel, subfamily N, member 4	1.180	1.888
Ly86	NM_010745	Mus musculus lymphocyte antigen 86	1.940	1.887
Dennd1c	NM_153551	Mus musculus DENN/MADD domain containing 1C	1.177	1.884
Tenm4	NM_011858	Mus musculus teneurin transmembrane protein 4	1.176	1.884
Dab2	NM_001037905	Mus musculus disabled 2, mitogen-responsive phosphoprotein	1.176	1.884
Sema6c	NM_001272024	Mus musculus sema domain, transmembrane domain , and cytoplasmic domain, 6C	1.458	1.883
Foxc2	NM_013519	Mus musculus forkhead box C2	1.174	1.882
Plcxd2	NM_001134480	Mus musculus phosphatidylinositol-specific phospholipase C, X domain containing 2	0.487	1.881
Snap91	NM_013669	Mus musculus synaptosomal-associated protein 91	1.455	1.881
Adcy7	NM_001037724	Mus musculus adenylate cyclase 7	1.930	1.881
Cxcl12	NM_021704	Mus musculus chemokine ligand 12	1.927	1.879
Golgb1	NM_030035	Mus musculus golgi autoantigen, golgin subfamily b, macrogolgin 1	0.486	1.879
Tenm3	NM_011857	Mus musculus teneurin transmembrane protein 3	1.452	1.879
Col15a1	NM_009928	Mus musculus collagen, type XV, alpha 1	1.170	1.878
Ccl2	NM_011333	Mus musculus chemokine ligand 2	1.923	1.877
Slc15a2	NM_021301	Mus musculus solute carrier family 15 , member 2	1.165	1.873
Gpr34	NM_011823	Mus musculus G protein-coupled receptor 34	1.165	1.873

Bgn	NM_007542	Mus musculus biglycan	1.445	1.873
Ssc5d	NM_173008	Mus musculus scavenger receptor cysteine rich family, 5 domains	1.164	1.872
Itpripl2	NM_001033380	Mus musculus inositol 1,4,5-triphosphate receptor interacting protein-like 2	2.072	1.871
Galnt12	NM_172693	Mus musculus UDP-N-acetyl-alpha-D-galactosamine:polypeptide N-acetylgalactosaminyltransferase 12	1.163	1.871
Mmrn1	NM_027613	Mus musculus multimerin 1	1.440	1.869
Ngfr	NM_033217	Mus musculus nerve growth factor receptor	1.161	1.869
Wfikkn2	NM_181819	Mus musculus WAP, follistatin/kazal, immunoglobulin, kunitz and netrin domain containing 2	1.440	1.868
C1qc	NM_007574	Mus musculus complement component 1, q subcomponent, C chain	2.067	1.868
Sost	NM_024449	Mus musculus sclerostin	0.479	1.867
Wdfy3	NM_172882	Mus musculus WD repeat and FYVE domain containing 3	0.478	1.867
Ucp3	NM_009464	Mus musculus uncoupling protein 3	1.436	1.866
Rbbp9	NM_015754	Mus musculus retinoblastoma binding protein 9	1.436	1.866
Tmem110	NM_028839	Mus musculus transmembrane protein 110	1.157	1.865
Lonrf3	NM_028894	Mus musculus LON peptidase N-terminal domain and ring finger 3	0.478	1.865
Enc1	NM_007930	Mus musculus ectodermal-neural cortex 1	1.904	1.865
Plxna4	NM_175750	Mus musculus plexin A4	1.904	1.865
Mfsd6	NM_178081	Mus musculus major facilitator superfamily domain containing 6	1.156	1.864
Sema4a	NM_013658	Mus musculus sema domain, immunoglobulin domain , transmembrane domain and short cytoplasmic domain, 4A	1.901	1.863
Tmem106a	NM_144830	Mus musculus transmembrane protein 106A	0.476	1.862
Pcsk5	NM_001163144	Mus musculus proprotein convertase subtilisin/kexin type 5	1.153	1.861
Kdelr3	NM_134090	Mus musculus KDEL endoplasmic reticulum protein retention receptor 3	1.430	1.861
Dnah8	NM_013811	Mus musculus dynein, axonemal, heavy chain 8	1.429	1.860
Smc4	NM_133786	Mus musculus structural maintenance of chromosomes 4	1.429	1.860
Thsd7a	NM_001164805	Mus musculus thrombospondin, type I, domain containing 7A	1.428	1.859
Rgs12	NM_173402	Mus musculus regulator of G-protein signaling 12	2.048	1.858
Cog1	NM_013581	Mus musculus component of oligomeric golgi complex 1	1.426	1.857
Tlr1	NM_030682	Mus musculus toll-like receptor 1	0.471	1.855
Tnxb	NM_031176	Mus musculus tenascin XB	2.043	1.855
Ddx60	NM_001293783	Mus musculus DEAD box polypeptide 60	0.471	1.854
Adgrd1	NM_001081342	Mus musculus adhesion G protein-coupled receptor D1	1.146	1.854
Arpc1b	NM_023142	Mus musculus actin related protein 2/3 complex, subunit 1B	1.668	1.854
Slc38a1	NM_134086	Mus musculus solute carrier family 38, member 1	1.884	1.853
Gprc5c	NM_147217	Mus musculus G protein-coupled receptor, family C, group 5, member C	1.143	1.851
Myo5a	NM_010864	Mus musculus myosin VA	1.418	1.851
2210408I21Rik	NM_001145676	Mus musculus RIKEN cDNA 2210408I21 gene	0.469	1.851
Ptk2b	NM_001162365	Mus musculus PTK2 protein tyrosine kinase 2 beta	1.879	1.850
Txndc5	NM_145367	Mus musculus thioredoxin domain containing 5	1.879	1.849
Cfc1	NM_007685	Mus musculus cripto, FRL-1, cryptic family 1	0.468	1.849
Capg	NM_001042534	Mus musculus capping protein , gelsolin-like	2.030	1.848
Zfp36l2	NM_001001806	Mus musculus zinc finger protein 36, C3H type-like 2	2.027	1.846
Hid1	NM_175454	Mus musculus HID1 domain containing	1.408	1.843
Casc4	NM_199038	Mus musculus cancer susceptibility candidate 4	1.406	1.841
Tbxas1	NM_011539	Mus musculus thromboxane A synthase 1, platelet	1.405	1.840
Fto	NM_011936	Mus musculus fat mass and obesity associated	0.463	1.839
Zfp248	NM_028335	Mus musculus zinc finger protein 248	0.462	1.839
Gm20736	NM_001037748	Mus musculus predicted gene, 20736	1.129	1.838
Apoc1	NM_007469	Mus musculus apolipoprotein C-I	1.858	1.837
Etv1	NM_007960	Mus musculus ets variant 1	1.857	1.837
3110035E14Rik	NM_178399	Mus musculus RIKEN cDNA 3110035E14 gene	1.127	1.836
Rab32	NM_026405	Mus musculus RAB32, member RAS oncogene family	1.398	1.835
Kcnj5	NM_010605	Mus musculus potassium inwardly-rectifying channel, subfamily J, member 5	2.007	1.835
Tbc1d8b	NM_001081499	Mus musculus TBC1 domain family, member 8B	1.397	1.834
Wfdc17	NM_001081957	Mus musculus WAP four-disulfide core domain 17	1.853	1.834
Ywhaq	NM_011739	Mus musculus tyrosine 3-monooxygenase/tryptophan 5-monooxygenase activation protein, theta polypeptide	1.852	1.833
Art4	NM_026639	Mus musculus ADP-ribosyltransferase 4	1.851	1.833
Atp9b	NM_015805	Mus musculus ATPase, class II, type 9B	1.123	1.832
Anxa1	NM_010730	Mus musculus annexin A1	1.849	1.832
Runx1	NM_001111023	Mus musculus runt related transcription factor 1	1.122	1.831
Casp4	NM_007609	Mus musculus caspase 4, apoptosis-related cysteine peptidase	1.845	1.829
Glo1	NM_025374	Mus musculus glyoxalase 1	1.997	1.829
Bicc1	NM_031397	Mus musculus bicaudal C homolog 1	1.995	1.828
Col5a1	NM_015734	Mus musculus collagen, type V, alpha 1	1.993	1.827
Adamts2	NM_175643	Mus musculus a disintegrin-like and metallopeptidase with thrombospondin type 1 motif, 2	0.455	1.827
Gm4788	NM_001029977	Mus musculus predicted gene 4788	1.838	1.825
Tlcd2	NM_027249	Mus musculus TLC domain containing 2	1.837	1.824449
Slc9a9	NM_177909	Mus musculus solute carrier family 9 , member 9	1.381	1.822068
Evi2a	NM_001033711	Mus musculus ecotropic viral integration site 2a	1.381	1.822028
Irf5	NM_012057	Mus musculus interferon regulatory factor 5	1.112	1.821648
Cdc42	NM_001243769	Mus musculus cell division cycle 42	1.377	1.818226
Arhgap4	NM_138630	Mus musculus Rho GTPase activating protein 4	1.108	1.817605
Slc25a1	NM_153150	Mus musculus solute carrier family 25 , member 1	1.375	1.817072
Slc29a3	NM_023596	Mus musculus solute carrier family 29 , member 3	1.106	1.816001
Bmp2	NM_007553	Mus musculus bone morphogenetic protein 2	1.106	1.815868
Clstn3	NM_153508	Mus musculus calsyntenin 3	1.106	1.815556
Dock1	NM_001033420	Mus musculus dedicator of cytokinesis 1	1.105	1.814550
Meox1	NM_010791	Mus musculus mesenchyme homeobox 1	1.820	1.814447
Akr1c12	NM_013777	Mus musculus aldo-keto reductase family 1, member C12	1.105	1.814314
Zfp354c	NM_013922	Mus musculus zinc finger protein 354C	1.371	1.814222
Igfbp3	NM_008343	Mus musculus insulin-like growth factor binding protein 3	1.970	1.813974
Nppb	NM_008726	Mus musculus natriuretic peptide type B	1.606	1.812355
Zpbp	NM_015785	Mus musculus zona pellucida binding protein	0.446	1.811295
Tcaf2	NM_146174	Mus musculus TRPM8 channel-associated factor 2	1.367	1.810711
Ush1c	NM_001163733	Mus musculus Usher syndrome 1C	1.365	1.809437
Uggt2	NM_001081252	Mus musculus UDP-glucose glycoprotein glucosyltransferase 2	0.445	1.809213
Tmed3	NM_025360	Mus musculus transmembrane emp24 domain containing 3	1.600	1.808082
Pdlim2	NM_145978	Mus musculus PDZ and LIM domain 2	1.809	1.807987

Adgrg3	NM_173036	Mus musculus adhesion G protein-coupled receptor G3	1.363	1.807971
Adam23	NM_011780	Mus musculus a disintegrin and metallopeptidase domain 23	0.444	1.807818
Cebpd	NM_007679	Mus musculus CCAAT/enhancer binding protein , delta	1.956	1.806504
C1rb	NM_001113356	Mus musculus complement component 1, r subcomponent B	1.096	1.806177
Col4a4	NM_007735	Mus musculus collagen, type IV, alpha 4	1.095	1.805398
Mbtd1	NM_134012	Mus musculus mbt domain containing 1	0.442	1.804853
Epha4	NM_007936	Mus musculus Eph receptor A4	1.802	1.804182
Ptpdc1	NM_207232	Mus musculus protein tyrosine phosphatase domain containing 1	0.441	1.803956
Cmtm5	NM_026066	Mus musculus CKLF-like MARVEL transmembrane domain containing 5	1.094	1.803868
Gm13304	NM_001193666	Mus musculus predicted gene 13304	1.592	1.802897
Ctss	NM_021281	Mus musculus cathepsin S	1.948	1.801992
Rcn3	NM_026555	Mus musculus reticulocalbin 3, EF-hand calcium binding domain	1.355	1.801500
Wls	NM_026582	Mus musculus wntless homolog	1.797	1.800770
Nek6	NM_001159631	Mus musculus NIMA -related expressed kinase 6	1.090	1.800651
Emp1	NM_001288628	Mus musculus epithelial membrane protein 1	1.942	1.798911
Zfp521	NM_145492	Mus musculus zinc finger protein 521	1.791	1.797140
Acer2	NM_001290543	Mus musculus alkaline ceramidase 2	0.437	1.797058
Klra1	NM_016659	Mus musculus killer cell lectin-like receptor, subfamily A, member 1	1.790	1.796751
Mmp23	NM_011985	Mus musculus matrix metallopeptidase 23	1.349	1.796364
Lyz1	NM_013590	Mus musculus lysozyme 1	1.935	1.794988
Pcdh18	NM_130448	Mus musculus protocadherin 18	1.347	1.794787
Fxyd5	NM_008761	Mus musculus FXYD domain-containing ion transport regulator 5	1.785	1.793959
Igfbp7	NM_008048	Mus musculus insulin-like growth factor binding protein 7	2.156	1.793266
Reck	NM_016678	Mus musculus reversion-inducing-cysteine-rich protein with kazal motifs	1.783	1.792786
Mrgprg	NM_203492	Mus musculus MAS-related GPR, member G	1.344	1.792742
Tspan18	NM_183180	Mus musculus tetraspanin 18	1.929	1.791675
3830403N18Rik	NM_027510	Mus musculus RIKEN cDNA 3830403N18 gene	1.081	1.791639
Lrmp	NM_008511	Mus musculus lymphoid-restricted membrane protein	1.080	1.791483
Hck	NM_001172117	Mus musculus hemopoietic cell kinase	1.340	1.790041
Serping1	NM_009776	Mus musculus serine peptidase inhibitor, clade G, member 1	1.572	1.789927
Gng13	NM_022422	Mus musculus guanine nucleotide binding protein , gamma 13	1.079	1.789704
Adam19	NM_001291891	Mus musculus a disintegrin and metallopeptidase domain 19	1.778	1.789641
Elmo3	NM_172760	Mus musculus engulfment and cell motility 3	1.337	1.787447
Igsf1	NM_177591	Mus musculus immunoglobulin superfamily, member 1	1.919	1.786227
Xkr8	NM_201368	Mus musculus X Kell blood group precursor related family member 8 homolog	1.074	1.785723
Zfp873	NM_001024626	Mus musculus zinc finger protein 873	0.430	1.784779
Rhobtb1	NM_001252638	Mus musculus Rho-related BTB domain containing 1	0.430	1.784516
Cygb	NM_030206	Mus musculus cytoglobin	1.563	1.783678
Rpp25	NM_133982	Mus musculus ribonuclease P/MRP 25 subunit	1.071	1.782321
Pqlc3	NM_172574	Mus musculus PQ loop repeat containing	1.330	1.781701
Notch2	NM_010928	Mus musculus notch 2	1.324	1.777649
Chl1	NM_007697	Mus musculus cell adhesion molecule with homology to L1CAM	0.425	1.777584
Wdr60	NM_146039	Mus musculus WD repeat domain 60	1.065	1.777340
Arl5c	NM_207231	Mus musculus ADP-ribosylation factor-like 5C	1.324	1.777184
Trim16	NM_053169	Mus musculus tripartite motif-containing 16	1.065	1.777148
Clec11a	NM_009131	Mus musculus C-type lectin domain family 11, member a	1.065	1.776916
Parvg	NM_001162500	Mus musculus parvin, gamma	0.425	1.776158
Evc2	NM_145920	Mus musculus Ellis van Creveld syndrome 2	1.754	1.776051
Lamc3	NM_011836	Mus musculus laminin gamma 3	1.064	1.775795
Egf	NM_010113	Mus musculus epidermal growth factor	1.321	1.775280
Themis2	NM_001033308	Mus musculus thymocyte selection associated family member 2	1.062	1.774333
Pdk4	NM_013743	Mus musculus pyruvate dehydrogenase kinase, isoenzyme 4	1.895	1.773463
Btk	NM_013482	Mus musculus Bruton agammaglobulinemia tyrosine kinase	1.060	1.771867
Pmp22	NM_001302260	Mus musculus peripheral myelin protein 22	1.892	1.771836
Vwf	NM_011708	Mus musculus Von Willebrand factor homolog	1.892	1.771569
Ada	NM_007398	Mus musculus adenosine deaminase	1.314	1.770028
Sparc	NM_009242	Mus musculus secreted acidic cysteine rich glycoprotein	1.541	1.769480
Gpsm2	NM_029522	Mus musculus G-protein signalling modulator 2	1.313	1.769296
Cnpy4	NM_178612	Mus musculus canopy 4 homolog	1.313	1.768868
Pla2g2d	NM_011109	Mus musculus phospholipase A2, group IID	1.312	1.767949
Itgav	NM_008402	Mus musculus integrin alpha V	1.740	1.767820
Psd3	NM_030263	Mus musculus pleckstrin and Sec7 domain containing 3	1.311	1.767699
Hook3	NM_207659	Mus musculus hook homolog 3	0.419	1.766664
Mmp19	NM_021412	Mus musculus matrix metallopeptidase 19	1.310	1.766366
Crlf3	NM_018776	Mus musculus cytokine receptor-like factor 3	1.735	1.765010
Sema3f	NM_011349	Mus musculus sema domain, immunoglobulin domain , short basic domain, secreted, 3F	1.051	1.764340
Ccdc149	NM_001256059	Mus musculus coiled-coil domain containing 149	1.307	1.764133
P2rx7	NM_011027	Mus musculus purinergic receptor P2X, ligand-gated ion channel, 7	1.305	1.762989
Fbp1	NM_019395	Mus musculus fructose bisphosphatase 1	1.046	1.758888
Glce	NM_033320	Mus musculus glucuronyl C5-epimerase	0.413	1.757913
Creb3	NM_013497	Mus musculus cAMP responsive element binding protein 3	1.722	1.757521
Tmem41b	NM_153525	Mus musculus transmembrane protein 41B	1.722	1.757409
Emilin1	NM_133918	Mus musculus elastin microfibril interfacer 1	1.043	1.756847
Lyz2	NM_017372	Mus musculus lysozyme 2	1.721	1.756847
Gria1	NM_001113325	Mus musculus glutamate receptor, ionotropic, AMPA1	1.043	1.756738
AI593442	NM_001286641	Mus musculus expressed sequence AI593442	1.043	1.756509
Ntn3	NM_010947	Mus musculus netrin 3	1.042	1.756000
Ermard	NM_001039552	Mus musculus ER membrane associated RNA degradation	0.412	1.755587
Sntb2	NM_009229	Mus musculus syntrophin, basic 2	1.295	1.755144
Mipol1	NM_001164370	Mus musculus mirror-image polydactyly gene 1 homolog	0.411	1.753384
Prss55	NM_001081063	Mus musculus protease, serine 55	1.039	1.752961
Pcolce2	NM_029620	Mus musculus procollagen C-endopeptidase enhancer 2	1.714	1.752696
Itm2c	NM_022417	Mus musculus integral membrane protein 2C	1.513	1.751508
Adamts4	NM_172845	Mus musculus a disintegrin-like and metallopeptidase with thrombospondin type 1 motif, 4	0.409	1.751413
Net1	NM_019671	Mus musculus neuroepithelial cell transforming gene 1	1.711	1.751119
Gpr68	NM_175493	Mus musculus G protein-coupled receptor 68	1.036	1.749814

Cd72	NM_001110320	Mus musculus CD72 antigen	1.034	1.748347
Akr1b8	NM_008012	Mus musculus aldo-keto reductase family 1, member B8	1.847	1.747783
Aurkc	NM_001080966	Mus musculus aurora kinase C	1.033	1.747659
Magee1	NM_053201	Mus musculus melanoma antigen, family E, 1	1.705	1.747535
Ccr5	NM_009917	Mus musculus chemokine receptor 5	1.032	1.746527
Cma2	NM_001024714	Mus musculus chymase 2, mast cell	1.283	1.746355
Iah1	NM_026347	Mus musculus isoamyl acetate-hydrolyzing esterase 1 homolog	1.283	1.745890
Bmp1	NM_009755	Mus musculus bone morphogenetic protein 1	1.843	1.745707
Dapp1	NM_011932	Mus musculus dual adaptor for phosphotyrosine and 3-phosphoinositides 1	1.281	1.745021
Ldlrad4	NM_172631	Mus musculus low density lipoprotein receptor class A domain containing 4	1.281	1.744473
Tmsb10	NM_001190327	Mus musculus thymosin, beta 10	1.841	1.744368
Pnmt	NM_008890	Mus musculus phenylethanolamine-N-methyltransferase	1.030	1.744157
Col6a4	NM_026763	Mus musculus collagen, type VI, alpha 4	0.405	1.744157
Aatk	NM_007377	Mus musculus apoptosis-associated tyrosine kinase	1.280	1.743855
Armcx1	NM_030066	Mus musculus armadillo repeat containing, X-linked 1	1.280	1.743816
Oxsm	NM_027695	Mus musculus 3-oxoacyl-ACP synthase, mitochondrial	1.839	1.743569
Nudt11	NM_021431	Mus musculus nudix -type motif 11	1.279	1.743052
Pde1a	NM_016744	Mus musculus phosphodiesterase 1A, calmodulin-dependent	1.278	1.742712
Armcx2	NM_026139	Mus musculus armadillo repeat containing, X-linked 2	1.696	1.742632
Kcne1l	NM_021487	Mus musculus potassium voltage-gated channel, Isk-related family, member 1-like, pseudogene	0.404	1.742516
Rab34	NM_033475	Mus musculus RAB34, member RAS oncogene family	1.694	1.741513
Tex29	NM_029326	Mus musculus testis expressed 29	0.403	1.741156
Tgif1	NM_001164075	Mus musculus TGFB-induced factor homeobox 1	1.275	1.740209
Ptger4	NM_001136079	Mus musculus prostaglandin E receptor 4	1.691	1.739892
Wnt6	NM_009526	Mus musculus wingless-type MMTV integration site family, member 6	0.402	1.739833
Pdzd4	NM_001029868	Mus musculus PDZ domain containing 4	1.024	1.739413
Crlf2	NM_016715	Mus musculus cytokine receptor-like factor 2	1.831	1.738999
Nckap5	NM_172484	Mus musculus NCK-associated protein 5	1.023	1.738389
Plk4	NM_011495	Mus musculus polo-like kinase 4	0.401	1.737679
Sh3pxd2b	NM_177364	Mus musculus SH3 and PX domains 2B	1.022	1.737380
2610002J02Rik	NM_001190445	Mus musculus RIKEN cDNA 2610002J02 gene	1.827	1.737361
Entpd2	NM_009849	Mus musculus ectonucleoside triphosphate diphosphohydrolase 2	1.827	1.737269
Zfp773	NM_029584	Mus musculus zinc finger protein 773	0.400	1.736707
Vmn1r25	NM_053238	Mus musculus vomeronasal 1 receptor 25	0.400	1.736393
Egr1	NM_007913	Mus musculus early growth response 1	1.685	1.736254
Psd	NM_028627	Mus musculus pleckstrin and Sec7 domain containing	1.021	1.736150
D930015E06Rik	NM_172681	Mus musculus RIKEN cDNA D930015E06 gene	0.399	1.735300
Slit2	NM_178804	Mus musculus slit homolog 2	1.267	1.734091
Coro1a	NM_001301374	Mus musculus coronin, actin binding protein 1A	1.681	1.733888
Gstt3	NM_133994	Mus musculus glutathione S-transferase, theta 3	1.267	1.733787
Gfra2	NM_008115	Mus musculus glial cell line derived neurotrophic factor family receptor alpha 2	1.266	1.733473
Clec4a3	NM_153197	Mus musculus C-type lectin domain family 4, member a3	1.018	1.733214
Cstf3	NM_177253	Mus musculus cleavage stimulation factor, 3' pre-RNA, subunit 3	0.398	1.732907
Wdr11	NM_172255	Mus musculus WD repeat domain 11	0.397	1.732384
Afap1l2	NM_146102	Mus musculus actin filament associated protein 1-like 2	1.264	1.732145
Crispld1	NM_031402	Mus musculus cysteine-rich secretory protein LCCL domain containing 1	1.016	1.731859
Ptpro	NM_011216	Mus musculus protein tyrosine phosphatase, receptor type, O	1.264	1.731706
Tnfrsf23	NM_024290	Mus musculus tumor necrosis factor receptor superfamily, member 23	0.397	1.731426
Cdk14	NM_011074	Mus musculus cyclin-dependent kinase 14	1.263	1.731013
Fzd1	NM_021457	Mus musculus frizzled homolog 1	1.263	1.730811
Serpinb1a	NM_025429	Mus musculus serine peptidase inhibitor, clade B, member 1a	1.674	1.730228
Tfcp2l1	NM_023755	Mus musculus transcription factor CP2-like 1	0.396	1.730017
Sgce	NM_011360	Mus musculus sarcoglycan, epsilon	1.813	1.729846
Phip	NM_001081216	Mus musculus pleckstrin homology domain interacting protein	0.395	1.728770
Glis2	NM_031184	Mus musculus GLIS family zinc finger 2	1.668	1.726929
Trim12c	NM_001146007	Mus musculus tripartite motif-containing 12C	1.257	1.726789
Tbx2	NM_009324	Mus musculus T-box 2	1.256	1.726199
Myc	NM_010849	Mus musculus myelocytomatosis oncogene	0.393	1.725891
Fam21	NM_026585	Mus musculus family with sequence similarity 21	1.009	1.725851
Rad54b	NM_001039556	Mus musculus RAD54 homolog B	0.393	1.725740
Kcna6	NM_013568	Mus musculus potassium voltage-gated channel, shaker-related, subfamily, member 6	1.255	1.725218
Sept6	NM_001177324	Mus musculus septin 6	1.007	1.723838
Fam65b	NM_001080381	Mus musculus family with sequence similarity 65, member B	1.006	1.722323
Iqsec1	NM_001134383	Mus musculus IQ motif and Sec7 domain 1	0.391	1.722141
Hdac1	NM_008228	Mus musculus histone deacetylase 1	1.251	1.722090
Ppp1r42	NM_145692	Mus musculus protein phosphatase 1, regulatory subunit 42	0.391	1.721853
Nrtn	NM_008738	Mus musculus neurturin	1.250	1.721687
Rasgef1b	NM_145839	Mus musculus RasGEF domain family, member 1B	1.658	1.721409
Tnfaip6	NM_009398	Mus musculus tumor necrosis factor alpha induced protein 6	1.004	1.720995
Cd44	NM_009851	Mus musculus CD44 antigen	1.249	1.720919
Clec4d	NM_010819	Mus musculus C-type lectin domain family 4, member d	1.004	1.720839
Ssr1	NM_025965	Mus musculus signal sequence receptor, alpha	1.003	1.720073
Hk3	NM_001033245	Mus musculus hexokinase 3	1.248	1.720045
Spsb2	NM_001306050	Mus musculus splA/ryanodine receptor domain and SOCS box containing 2	1.655	1.719732
Fhl4	NM_010214	Mus musculus four and a half LIM domains 4	0.389	1.719411
Prnp	NM_001278256	Mus musculus prion protein	1.654	1.719125
Stab1	NM_138672	Mus musculus stabilin 1	1.792	1.719023
Cdh11	NM_009866	Mus musculus cadherin 11	1.246	1.718331
Lrrc49	NM_145616	Mus musculus leucine rich repeat containing 49	1.001	1.718174
Id2	NM_010496	Mus musculus inhibitor of DNA binding 2	1.653	1.718159
Mzb1	NM_027222	Mus musculus marginal zone B and B1 cell-specific protein 1	1.001	1.718131
Map6	NM_001043355	Mus musculus microtubule-associated protein 6	1.651	1.717433
Mmp2	NM_008610	Mus musculus matrix metallopeptidase 2	1.651	1.717008
Lefty1	NM_010094	Mus musculus left right determination factor 1	1.000	1.716961
Pdlim5	NM_001190852	Mus musculus PDZ and LIM domain 5	1.243	1.716457
Snx2	NM_026386	Mus musculus sorting nexin 2	1.649	1.715871

Brdt	NM_054054	Mus musculus bromodomain, testis-specific	0.998	1.715524
Tchhl1	NM_027762	Mus musculus trichohyalin-like 1	1.241	1.714784
Ncf4	NM_008677	Mus musculus neutrophil cytosolic factor 4	1.646	1.714638
Slc16a2	NM_009197	Mus musculus solute carrier family 16 , member 2	1.646	1.714342
Mcm10	NM_027290	Mus musculus minichromosome maintenance deficient 10	0.386	1.714279
Rgs14	NM_016758	Mus musculus regulator of G-protein signaling 14	0.996	1.713858
Wdfy2	NM_175546	Mus musculus WD repeat and FYVE domain containing 2	0.386	1.713722
Numbl	NM_010950	Mus musculus numb-like	1.240	1.713718
Daf2	NM_007827	Mus musculus decay accelerating factor 2	0.995	1.713095
Pcbd1	NM_025273	Mus musculus pterin 4 alpha carbinolamine dehydratase/dimerization cofactor of hepatocyte nuclear factor 1 alpha 1	1.239	1.713005
Defb25	NM_001039122	Mus musculus defensin beta 25	0.995	1.712713
Tmem176b	NM_023056	Mus musculus transmembrane protein 176B	1.236	1.711228
Slc41a2	NM_177388	Mus musculus solute carrier family 41, member 2	0.993	1.711101
Entpd1	NM_001304721	Mus musculus ectonucleoside triphosphate diphosphohydrolase 1	0.993	1.711047
Camkk1	NM_018883	Mus musculus calcium/calmodulin-dependent protein kinase kinase 1, alpha	1.236	1.710860
Aldh1l2	NM_153543	Mus musculus aldehyde dehydrogenase 1 family, member L2	0.992	1.709779
Nfia	NM_001122953	Mus musculus nuclear factor I/A	0.383	1.709316
Pyroxd2	NM_029011	Mus musculus pyridine nucleotide-disulphide oxidoreductase domain 2	1.233	1.708592
B3gnt9	NM_178879	Mus musculus UDP-GlcNAc:betaGal beta-1,3-N-acetylglucosaminyltransferase 9	1.233	1.708562
Rassf7	NM_025886	Mus musculus Ras association domain family member 7	0.990	1.708276
Cd55	NM_010016	Mus musculus CD55 antigen	1.232	1.708057
Folr2	NM_008035	Mus musculus folate receptor 2	1.634	1.707884
Myo1d	NM_177390	Mus musculus myosin ID	1.771	1.707796
Fads2	NM_019699	Mus musculus fatty acid desaturase 2	0.989	1.707751
Cfhr2	NM_001025575	Mus musculus complement factor H-related 2	1.634	1.707469
Mob1a	NM_145571	Mus musculus MOB kinase activator 1A	0.382	1.707311
Timm10b	NM_019502	Mus musculus translocase of inner mitochondrial membrane 10B	1.633	1.707058
Lair1	NM_178611	Mus musculus leukocyte-associated Ig-like receptor 1	0.987	1.705406
Mxra7	NM_026280	Mus musculus matrix-remodelling associated 7	1.228	1.705168
Arhgap8	NM_001205334	Mus musculus Rho GTPase activating protein 8	0.986	1.705130
Itga10	NM_001302471	Mus musculus integrin, alpha 10	0.986	1.705107
Erdr1	NM_133362	Mus musculus erythroid differentiation regulator 1	1.437	1.704178
Bcl2l15	NM_001142959	Mus musculus BCLl2-like 15	0.379	1.703982
Kbtbd11	NM_029116	Mus musculus kelch repeat and BTB domain containing 11	1.225	1.703206
Ppap2c	NM_015817	Mus musculus phosphatidic acid phosphatase type 2C	1.626	1.703048
Fkbp7	NM_010222	Mus musculus FK506 binding protein 7	1.761	1.702803
Loxl2	NM_033325	Mus musculus lysyl oxidase-like 2	1.761	1.702718
Wfdc18	NM_007969	Mus musculus WAP four-disulfide core domain 18	0.984	1.702657
Sept8	NM_001252332	Mus musculus septin 8	0.984	1.702536
Klf10	NM_013692	Mus musculus Kruppel-like factor 10	1.624	1.702384
1700019D03Rik	NM_144953	Mus musculus RIKEN cDNA 1700019D03 gene	0.983	1.701943
Cebpa	NM_007678	Mus musculus CCAAT/enhancer binding protein , alpha	1.222	1.701041
Irak3	NM_028679	Mus musculus interleukin-1 receptor-associated kinase 3	0.377	1.700682
Pikfyve	NM_011086	Mus musculus phosphoinositide kinase, FYVE finger containing	0.981	1.700391
Mafb	NM_010658	Mus musculus v-maf musculoaponeurotic fibrosarcoma oncogene family, protein B	1.221	1.700321
Pth1r	NM_011199	Mus musculus parathyroid hormone 1 receptor	1.221	1.700253
Slc16a3	NM_030696	Mus musculus solute carrier family 16 , member 3	1.220	1.699578
P3h3	NM_013534	Mus musculus prolyl 3-hydroxylase 3	1.619	1.699306
Tspyl3	NM_198617	Mus musculus TSPY-like 3	1.220	1.699268
Fam26e	NM_178908	Mus musculus family with sequence similarity 26, member E	0.980	1.699166
Tgfb1i1	NM_001289550	Mus musculus transforming growth factor beta 1 induced transcript 1	0.980	1.699102
Mtr	NM_001081128	Mus musculus 5-methyltetrahydrofolate-homocysteine methyltransferase	0.376	1.698796
Gpc2	NM_172412	Mus musculus glypican 2	0.978	1.697792
Mysm1	NM_177239	Mus musculus myb-like, SWIRM and MPN domains 1	0.375	1.697562
Hist2h2bb	NM_175666	Mus musculus histone cluster 2, H2bb	1.217	1.697369
Zfp937	NM_001142411	Mus musculus zinc finger protein 937	1.954	1.697050
C920025E04Rik	NM_001271005	Mus musculus RIKEN cDNA C920025E04 gene	1.750	1.697011
Slc24a5	NM_175034	Mus musculus solute carrier family 24, member 5	0.375	1.696910
Ncf2	NM_010877	Mus musculus neutrophil cytosolic factor 2	0.975	1.695294
Pafah1b3	NM_008776	Mus musculus platelet-activating factor acetylhydrolase, isoform 1b, subunit 3	1.214	1.695158
Gas1	NM_008086	Mus musculus growth arrest specific 1	1.611	1.694970
Hist1h1d	NM_145713	Mus musculus histone cluster 1, H1d	0.374	1.694955
Espl1	NM_001014976	Mus musculus extra spindle pole bodies 1	0.975	1.694856
Gm8909	NM_001081032	Mus musculus predicted gene 8909	1.611	1.694808
1810041L15Rik	NM_001163145	Mus musculus RIKEN cDNA 1810041L15 gene	1.214	1.694714
Gcgr	NM_008101	Mus musculus glucagon receptor	1.214	1.694607
Fbxl16	NM_001164225	Mus musculus F-box and leucine-rich repeat protein 16	1.213	1.694370
Cd163l1	NM_172909	Mus musculus CD163 molecule-like 1	0.974	1.693923
2810006K23Rik	NM_028310	Mus musculus RIKEN cDNA 2810006K23 gene	0.373	1.693125
Xpo7	NM_023045	Mus musculus exportin 7	0.372	1.692733
Card14	NM_130886	Mus musculus caspase recruitment domain family, member 14	0.972	1.692630
Fignl1	NM_001163359	Mus musculus fidgetin-like 1	0.972	1.692464
Prkcd	NM_011103	Mus musculus protein kinase C, delta	0.972	1.692377
Prrg1	NM_001289683	Mus musculus proline rich Gla 1	0.972	1.692362
Anxa5	NM_009673	Mus musculus annexin A5	1.740	1.691664
Adgrb1	NM_174991	Mus musculus adhesion G protein-coupled receptor B1	0.371	1.690973
Pla2g7	NM_013737	Mus musculus phospholipase A2, group VII	1.207	1.690116
Zak	NM_023057	Mus musculus sterile alpha motif and leucine zipper containing kinase AZK	0.969	1.689702
Nrcam	NM_176930	Mus musculus neuronal cell adhesion molecule	0.969	1.689124
Sfi1	NM_030207	Mus musculus Sfi1 homolog, spindle assembly associated	0.968	1.688935
Cd302	NM_001290660	Mus musculus CD302 antigen	1.598	1.687599
Erbb2	NM_001003817	Mus musculus v-erb-b2 erythroblastic leukemia viral oncogene homolog 2, neuro/glioblastoma derived oncogene homo	1.597	1.687533
Plaur	NM_011113	Mus musculus plasminogen activator, urokinase receptor	1.204	1.687286
Mical1	NM_138315	Mus musculus microtubule associated monooxygenase, calponin and LIM domain containing 1	1.595	1.686237
Pglyrp1	NM_009402	Mus musculus peptidoglycan recognition protein 1	0.368	1.686143
4930563E22Rik	NM_001163728	Mus musculus RIKEN cDNA 4930563E22 gene	0.964	1.685156

Prkg2	NM_008926	Mus musculus protein kinase, cGMP-dependent, type II	0.367	1.685023
Fam179a	NM_177087	Mus musculus family with sequence similarity 179, member A	0.963	1.684122
Tm6sf1	NM_145375	Mus musculus transmembrane 6 superfamily member 1	0.962	1.683729
Dram1	NM_027878	Mus musculus DNA-damage regulated autophagy modulator 1	0.961	1.682823
Polr2l	NM_025593	Mus musculus polymerase II polypeptide L	1.588	1.682082
Arhgap6	NM_009707	Mus musculus Rho GTPase activating protein 6	1.196	1.681574
Cplx2	NM_009946	Mus musculus complexin 2	1.720	1.681497
Wdr92	NM_178909	Mus musculus WD repeat domain 92	1.401	1.681477
Csf1r	NM_001037859	Mus musculus colony stimulating factor 1 receptor	1.400	1.681252
Slc9a3r1	NM_012030	Mus musculus solute carrier family 9 , member 3 regulator 1	1.195	1.681025
Irf8	NM_008320	Mus musculus interferon regulatory factor 8	1.195	1.680747
Abca8a	NM_153145	Mus musculus ATP-binding cassette, sub-family A , member 8a	1.193	1.679560
Vat1	NM_012037	Mus musculus vesicle amine transport protein 1 homolog	1.193	1.679451
Gatm	NM_025961	Mus musculus glycine amidinotransferase	0.957	1.679295
Il6ra	NM_010559	Mus musculus interleukin 6 receptor, alpha	0.362	1.677
Fam198a	NM_177743	Mus musculus family with sequence similarity 198, member A	0.362	1.677
Vgll3	NM_028572	Mus musculus vestigial like 3	1.190	1.677
Wbp5	NM_011712	Mus musculus WW domain binding protein 5	1.578	1.677
Efs	NM_010112	Mus musculus embryonal Fyn-associated substrate	1.187	1.675
Gm2030	NM_001100445	Mus musculus predicted gene 2030	0.951	1.674
Cenpt	NM_177150	Mus musculus centromere protein T	0.951	1.674
Atf7	NM_146065	Mus musculus activating transcription factor 7	1.185	1.673
Trim47	NM_001205081	Mus musculus tripartite motif-containing 47	1.704	1.673
Ica1	NM_010492	Mus musculus islet cell autoantigen 1	1.569	1.672
Agpat4	NM_026644	Mus musculus 1-acylglycerol-3-phosphate O-acyltransferase 4	1.568	1.671
Cntln	NM_177385	Mus musculus centlein, centrosomal protein	1.181	1.671
Slc25a45	NM_134154	Mus musculus solute carrier family 25, member 45	1.181	1.671
Gnai1	NM_010305	Mus musculus guanine nucleotide binding protein , alpha inhibiting 1	1.180	1.670
Cnksr2	NM_177751	Mus musculus connector enhancer of kinase suppressor of Ras 2	0.357	1.670
Mybpc2	NM_146189	Mus musculus myosin binding protein C, fast-type	1.563	1.669
AU018091	NM_001004153	Mus musculus expressed sequence AU018091	0.945	1.669
C1qtnf2	NM_026979	Mus musculus C1q and tumor necrosis factor related protein 2	1.562	1.668
P3h4	NM_176830	Mus musculus prolyl 3-hydroxylase family member 4	1.561	1.668
Selplg	NM_009151	Mus musculus selectin, platelet ligand	1.176	1.667
Zscan18	NM_001017955	Mus musculus zinc finger and SCAN domain containing 18	0.944	1.667
Pard6g	NM_053117	Mus musculus par-6 family cell polarity regulator gamma	1.560	1.667
Csrp1	NM_007791	Mus musculus cysteine and glycine-rich protein 1	1.374	1.666
Aspa	NM_023113	Mus musculus aspartoacylase	1.173	1.665
Trim68	NM_198012	Mus musculus tripartite motif-containing 68	1.557	1.665
Trim46	NM_183037	Mus musculus tripartite motif-containing 46	0.942	1.665
Kctd8	NM_175519	Mus musculus potassium channel tetramerisation domain containing 8	0.354	1.665
Lcp2	NM_010696	Mus musculus lymphocyte cytosolic protein 2	0.941	1.665
Cntn2	NM_177129	Mus musculus contactin 2	0.940	1.664
Fam129a	NM_022018	Mus musculus family with sequence similarity 129, member A	1.555	1.664
Fgf10	NM_008002	Mus musculus fibroblast growth factor 10	0.940	1.664
Kcnip3	NM_019789	Mus musculus Kv channel interacting protein 3, calsenilin	0.353	1.664
Ahnak	NM_001039959	Mus musculus AHNAK nucleoprotein	1.684	1.663
Tbx20	NM_194263	Mus musculus T-box 20	1.551	1.662
Nab2	NM_008668	Mus musculus Ngfi-A binding protein 2	1.550	1.661
Hs3st1	NM_010474	Mus musculus heparan sulfate 3-O-sulfotransferase 1	0.937	1.661
Fmnl2	NM_172409	Mus musculus formin-like 2	0.352	1.661
Fam149a	NM_153535	Mus musculus family with sequence similarity 149, member A	0.937	1.661
Sphk1	NM_025367	Mus musculus sphingosine kinase 1	1.167	1.661
Rnase4	NM_021472	Mus musculus ribonuclease, RNase A family 4	1.678	1.660
P2ry6	NM_183168	Mus musculus pyrimidinergic receptor P2Y, G-protein coupled, 6	1.546	1.659
Sfxn1	NM_027324	Mus musculus sideroflexin 1	1.543	1.658
Cnga3	NM_001282010	Mus musculus cyclic nucleotide gated channel alpha 3	0.932	1.657
Col6a2	NM_146007	Mus musculus collagen, type VI, alpha 2	1.541	1.657
Clmp	NM_133733	Mus musculus CXADR-like membrane protein	0.932	1.657
Lypd6b	NM_027990	Mus musculus LY6/PLAUR domain containing 6B	1.160	1.656
Prmt2	NM_001077638	Mus musculus protein arginine N-methyltransferase 2	1.669	1.656
Zc2hc1a	NM_173181	Mus musculus zinc finger, C2HC-type containing 1A	1.160	1.656
Ctdspl2	NM_212450	Mus musculus CTD small phosphatase like 2	0.931	1.656
Vmn1r65	NM_030738	Mus musculus vomeronasal 1 receptor 65	1.160	1.656
Ociad2	NM_026950	Mus musculus OCIA domain containing 2	1.159	1.655
Rhod	NM_007485	Mus musculus ras homolog gene family, member D	1.159	1.655
Zkscan2	NM_001081329	Mus musculus zinc finger with KRAB and SCAN domains 2	0.348	1.655
Pvrl1	NM_021424	Mus musculus poliovirus receptor-related 1	1.158	1.654
Nxpe3	NM_001134457	Mus musculus neurexophilin and PC-esterase domain family, member 3	1.158	1.654
Clic1	NM_033444	Mus musculus chloride intracellular channel 1	1.665	1.654
Snx10	NM_028035	Mus musculus sorting nexin 10	1.157	1.654
Pcdhgb1	NM_033574	Mus musculus protocadherin gamma subfamily B, 1	0.347	1.654
Ppfibp1	NM_026221	Mus musculus PTPRF interacting protein, binding protein 1	1.156	1.653
Dpysl3	NM_009468	Mus musculus dihydropyrimidinase-like 3	1.534	1.653
Fkbp1b	NM_016863	Mus musculus FK506 binding protein 1b	1.156	1.653
C5ar1	NM_001173550	Mus musculus complement component 5a receptor 1	1.156	1.653
Bcas1	NM_029815	Mus musculus breast carcinoma amplified sequence 1	0.926	1.652
Lca5	NM_027448	Mus musculus Leber congenital amaurosis 5	0.925	1.651
Cys1	NM_138686	Mus musculus cystin 1	1.152	1.650
Nfkbid	NM_172142	Mus musculus nuclear factor of kappa light polypeptide gene enhancer in B cells inhibitor, delta	0.924	1.650
Stxbp2	NM_011503	Mus musculus syntaxin binding protein 2	1.151	1.649
Trem2	NM_001272078	Mus musculus triggering receptor expressed on myeloid cells 2	1.526	1.649
Hmcn1	NM_001024720	Mus musculus hemicentin 1	0.922	1.648
Epha7	NM_010141	Mus musculus Eph receptor A7	0.921	1.648
Flna	NM_001290421	Mus musculus filamin, alpha	1.652	1.647
App	NM_001198823	Mus musculus amyloid beta precursor protein	1.344	1.647

Mnd1	NM_029797	Mus musculus meiotic nuclear divisions 1 homolog	1.147	1.647
Eno2	NM_013509	Mus musculus enolase 2, gamma neuronal	1.147	1.647
Gm7120	NM_001039244	Mus musculus predicted gene 7120	1.147	1.646
H2-T23	NM_010398	Mus musculus histocompatibility 2, T region locus 23	1.649	1.646
Zfp462	NM_172867	Mus musculus zinc finger protein 462	1.520	1.646
Ntsr2	NM_008747	Mus musculus neurotensin receptor 2	1.145	1.645
Pcdhb20	NM_053145	Mus musculus protocadherin beta 20	1.144	1.644
Rab43	NM_001039394	Mus musculus RAB43, member RAS oncogene family	0.340	1.643
Cxxc4	NM_001004367	Mus musculus CXXC finger 4	0.340	1.643
Tcl1b2	NM_013775	Mus musculus T cell leukemia/lymphoma 1B, 2	0.340	1.643
Hps1	NM_019424	Mus musculus Hermansky-Pudlak syndrome 1 homolog	1.142	1.643
Npdc1	NM_008721	Mus musculus neural proliferation, differentiation and control 1	1.641	1.642
Iqgap1	NM_016721	Mus musculus IQ motif containing GTPase activating protein 1	1.513	1.641
Mmp14	NM_008608	Mus musculus matrix metallopeptidase 14	0.914	1.641
Corin	NM_016869	Mus musculus corin	1.638	1.641
Krt80	NM_028770	Mus musculus keratin 80	0.913	1.640
Ptprj	NM_008982	Mus musculus protein tyrosine phosphatase, receptor type, J	1.138	1.640
Maged1	NM_019791	Mus musculus melanoma antigen, family D, 1	1.637	1.640
Ryr3	NM_177652	Mus musculus ryanodine receptor 3	1.137	1.640
Dpysl2	NM_009955	Mus musculus dihydropyrimidinase-like 2	1.636	1.639
B4galt2	NM_017377	Mus musculus UDP-Gal:betaGlcNAc beta 1,4- galactosyltransferase, polypeptide 2	1.137	1.639
Stox1	NM_001033260	Mus musculus storkhead box 1	0.337	1.639
Crtap	NM_019922	Mus musculus cartilage associated protein	1.634	1.638
Scn7a	NM_009135	Mus musculus sodium channel, voltage-gated, type VII, alpha	0.910	1.638
Atp8b1	NM_001001488	Mus musculus ATPase, class I, type 8B, member 1	0.910	1.638
Wnk4	NM_175638	Mus musculus WNK lysine deficient protein kinase 4	1.135	1.638
Farp1	NM_134082	Mus musculus FERM, RhoGEF and pleckstrin domain protein 1	0.909	1.637
Lpp	NM_178665	Mus musculus LIM domain containing preferred translocation partner in lipoma	0.336	1.637
Ltn1	NM_001081068	Mus musculus listerin E3 ubiquitin protein ligase 1	0.908	1.636
Bcl3	NM_033601	Mus musculus B cell leukemia/lymphoma 3	1.502	1.636
Sox4	NM_009238	Mus musculus SRY -box 4	1.501	1.635
Shcbp1	NM_011369	Mus musculus Shc SH2-domain binding protein 1	0.335	1.635
Pygb	NM_153781	Mus musculus brain glycogen phosphorylase	1.500	1.635
Sh3bgrl	NM_019989	Mus musculus SH3-binding domain glutamic acid-rich protein like	1.626	1.634
Vars2	NM_175137	Mus musculus valyl-tRNA synthetase 2, mitochondrial	1.129	1.634
Stk25	NM_021537	Mus musculus serine/threonine kinase 25	1.129	1.634
Tigd3	NM_198634	Mus musculus tigger transposable element derived 3	0.334	1.634
Zfp934	NM_175252	Mus musculus zinc finger protein 934	0.905	1.634
Trip13	NM_027182	Mus musculus thyroid hormone receptor interactor 13	0.904	1.633
Adam5	NM_007401	Mus musculus a disintegrin and metallopeptidase domain 5	0.333	1.633
Slc16a12	NM_172838	Mus musculus solute carrier family 16 , member 12	0.902	1.631
Sfxn3	NM_053197	Mus musculus sideroflexin 3	1.493	1.631
Ms4a6d	NM_026835	Mus musculus membrane-spanning 4-domains, subfamily A, member 6D	0.901	1.630
Prr3	NM_145487	Mus musculus proline-rich polypeptide 3	1.124	1.630
Anxa2	NM_007585	Mus musculus annexin A2	1.314	1.629
Cadm3	NM_053199	Mus musculus cell adhesion molecule 3	0.899	1.629
Rhof	NM_175092	Mus musculus ras homolog gene family, member f	0.899	1.628
Lrrc59	NM_133807	Mus musculus leucine rich repeat containing 59	0.899	1.628
Tet1	NM_027384	Mus musculus tet methylcytosine dioxygenase 1	0.899	1.628
AA414768	NM_001272033	Mus musculus expressed sequence AA414768	0.898	1.628
Fzd7	NM_008057	Mus musculus frizzled homolog 7	1.119	1.627
Cyp46a1	NM_010010	Mus musculus cytochrome P450, family 46, subfamily a, polypeptide 1	0.329	1.627
Rnf149	NM_001033135	Mus musculus ring finger protein 149	1.119	1.627
Gem	NM_010276	Mus musculus GTP binding protein	1.117	1.625
Ifit1	NM_008331	Mus musculus interferon-induced protein with tetratricopeptide repeats 1	0.895	1.625
Pi15	NM_053191	Mus musculus peptidase inhibitor 15	0.328	1.625
Atp10a	NM_009728	Mus musculus ATPase, class V, type 10A	1.117	1.625
Tmem181a	NM_001033178	Mus musculus transmembrane protein 181A	0.328	1.625
S100a10	NM_009112	Mus musculus S100 calcium binding protein A10	1.606	1.625
Fbxw17	NM_175401	Mus musculus F-box and WD-40 domain protein 17	1.481	1.624
Ngfrap1	NM_009750	Mus musculus nerve growth factor receptor associated protein 1	1.116	1.624
Slc22a18	NM_001042760	Mus musculus solute carrier family 22 , member 18	0.894	1.624
Myh11	NM_013607	Mus musculus myosin, heavy polypeptide 11, smooth muscle	1.115	1.624
Tmem189	NM_145538	Mus musculus transmembrane protein 189	0.327	1.624
Rbm47	NM_178446	Mus musculus RNA binding motif protein 47	0.893	1.623
Jak2	NM_008413	Mus musculus Janus kinase 2	0.893	1.623
Avil	NM_009635	Mus musculus advillin	0.892	1.623
Emp3	NM_010129	Mus musculus epithelial membrane protein 3	1.476	1.622
Vkorc1	NM_178600	Mus musculus vitamin K epoxide reductase complex, subunit 1	1.601	1.622
Itpr3	NM_080553	Mus musculus inositol 1,4,5-triphosphate receptor 3	1.475	1.621
Slc2a5	NM_019741	Mus musculus solute carrier family 2 , member 5	0.890	1.621
Dynlt1c	NM_001166630	Mus musculus dynein light chain Tctex-type 1C	1.599	1.621
Pcdhb14	NM_053139	Mus musculus protocadherin beta 14	0.889	1.620
Eya2	NM_010165	Mus musculus eyes absent 2 homolog	1.109	1.620
Adamts10	NM_172619	Mus musculus a disintegrin-like and metallopeptidase with thrombospondin type 1 motif, 10	0.888	1.619
Nid1	NM_010917	Mus musculus nidogen 1	1.595	1.619
Mxra8	NM_024263	Mus musculus matrix-remodelling associated 8	1.108	1.619
Fam13c	NM_024244	Mus musculus family with sequence similarity 13, member C	0.887	1.619
Tmem121	NM_153776	Mus musculus transmembrane protein 121	0.887	1.619
Mtfr1	NM_026182	Mus musculus mitochondrial fission regulator 1	1.594	1.618
Mpp1	NM_008621	Mus musculus membrane protein, palmitoylated	1.468	1.618
Ltc4s	NM_008521	Mus musculus leukotriene C4 synthase	1.467	1.617
Igsf6	NM_030691	Mus musculus immunoglobulin superfamily, member 6	1.105	1.617
Mamdc2	NM_174857	Mus musculus MAM domain containing 2	0.885	1.617
Csf1	NM_001113530	Mus musculus colony stimulating factor 1	1.104	1.616
Arap1	NM_001040111	Mus musculus ArfGAP with RhoGAP domain, ankyrin repeat and PH domain 1	1.588	1.616

Trim11	NM_001290988	Mus musculus tripartite motif-containing 11	1.588	1.616
Pcdhac2	NM_001003672	Mus musculus protocadherin alpha subfamily C, 2	0.322	1.615
Ciita	NM_001302619	Mus musculus class II transactivator	1.103	1.615
Golim4	NM_001291069	Mus musculus golgi integral membrane protein 4	1.463	1.615
Tmem194b	NM_001142647	Mus musculus transmembrane protein 194B	0.882	1.614
Adamtsl2	NM_029981	Mus musculus ADAMTS-like 2	1.098	1.612
Epb4.1l3	NM_013813	Mus musculus erythrocyte protein band 4.1-like 3	0.877	1.610
Tyms	NM_021288	Mus musculus thymidylate synthase	1.095	1.610
Ptgr1	NM_025968	Mus musculus prostaglandin reductase 1	1.094	1.610
Dnajc10	NM_024181	Mus musculus DnaJ homolog, subfamily C, member 10	1.452	1.609
Ovgp1	NM_007696	Mus musculus oviductal glycoprotein 1	0.876	1.609
Spn	NM_009259	Mus musculus sialophorin	0.876	1.609
Disc1	NM_174853	Mus musculus disrupted in schizophrenia 1	0.317	1.609
Apobec1	NM_031159	Mus musculus apolipoprotein B mRNA editing enzyme, catalytic polypeptide 1	1.093	1.608
Snx7	NM_029655	Mus musculus sorting nexin 7	1.092	1.608
Ptpn13	NM_011204	Mus musculus protein tyrosine phosphatase, non-receptor type 13	0.874	1.607
Irs3	NM_010571	Mus musculus insulin receptor substrate 3	0.873	1.607
Tnnt3	NM_001163664	Mus musculus troponin T3, skeletal, fast	0.873	1.606
Dpy19l1	NM_172920	Mus musculus dpy-19-like 1	0.315	1.606
Marveld1	NM_183195	Mus musculus MARVEL domain containing 1	1.089	1.606
Fndc4	NM_022424	Mus musculus fibronectin type III domain containing 4	1.445	1.606
D3Ertd751e	NM_028667	Mus musculus DNA segment, Chr 3, ERATO Doi 751, expressed	1.445	1.606
Tiam1	NM_009384	Mus musculus T cell lymphoma invasion and metastasis 1	0.872	1.605
Ppic	NM_008908	Mus musculus peptidylprolyl isomerase C	1.567	1.605
Hmgcs2	NM_008256	Mus musculus 3-hydroxy-3-methylglutaryl-Coenzyme A synthase 2	1.444	1.605
Tagln2	NM_178598	Mus musculus transgelin 2	1.272	1.604
Mcm3	NM_008563	Mus musculus minichromosome maintenance deficient 3	0.870	1.604
Sod3	NM_011435	Mus musculus superoxide dismutase 3, extracellular	1.562	1.603
Aff3	NM_001290814	Mus musculus AF4/FMR2 family, member 3	1.084	1.603
Fyb	NM_011815	Mus musculus FYN binding protein	1.084	1.602
Hltf	NM_009210	Mus musculus helicase-like transcription factor	1.438	1.602
Ano6	NM_001253813	Mus musculus anoctamin 6	0.867	1.602
Klk1b5	NM_008456	Mus musculus kallikrein 1-related peptidase b5	0.312	1.601
Prkcdbp	NM_028444	Mus musculus protein kinase C, delta binding protein	1.558	1.601
Phkg1	NM_011079	Mus musculus phosphorylase kinase gamma 1	1.435	1.601
Kirrel	NM_001170985	Mus musculus kin of IRRE like	1.081	1.600
Edem2	NM_145537	Mus musculus ER degradation enhancer, mannosidase alpha-like 2	0.311	1.600
Trmt44	NM_030208	Mus musculus tRNA methyltransferase 44	0.311	1.599
Arhgap11a	NM_181416	Mus musculus Rho GTPase activating protein 11A	0.311	1.599
Galnt16	NM_001081421	Mus musculus UDP-N-acetyl-alpha-D-galactosamine:polypeptide N-acetylgalactosaminyltransferase 16	1.431	1.599
S100a11	NM_016740	Mus musculus S100 calcium binding protein A11	1.431	1.599
Enpp1	NM_008813	Mus musculus ectonucleotide pyrophosphatase/phosphodiesterase 1	0.863	1.598
Ctso	NM_177662	Mus musculus cathepsin O	0.863	1.598
Fstl3	NM_031380	Mus musculus follistatin-like 3	1.078	1.598
Cdc14b	NM_172587	Mus musculus CDC14 cell division cycle 14B	1.551	1.598
Uxs1	NM_026430	Mus musculus UDP-glucuronate decarboxylase 1	0.309	1.597
Map4k1	NM_008279	Mus musculus mitogen-activated protein kinase kinase kinase kinase 1	0.862	1.597
Scpep1	NM_029023	Mus musculus serine carboxypeptidase 1	1.549	1.597
Igf2	NM_010514	Mus musculus insulin-like growth factor 2	1.076	1.597
Pirb	NM_011095	Mus musculus paired Ig-like receptor B	1.427	1.596
Mertk	NM_008587	Mus musculus c-mer proto-oncogene tyrosine kinase	0.861	1.596
Gm13154	NM_001014397	Mus musculus predicted gene 13154	1.075	1.596
Cdt1	NM_026014	Mus musculus chromatin licensing and DNA replication factor 1	1.073	1.595
Dse	NM_172508	Mus musculus dermatan sulfate epimerase	0.859	1.595
Ntn4	NM_021320	Mus musculus netrin 4	0.858	1.594
Fasn	NM_007988	Mus musculus fatty acid synthase	1.544	1.594
Il27ra	NM_016671	Mus musculus interleukin 27 receptor, alpha	1.072	1.594
Ncf1	NM_001286037	Mus musculus neutrophil cytosolic factor 1	0.857	1.593
Trp53bp1	NM_013735	Mus musculus transformation related protein 53 binding protein 1	0.306	1.593
Actn1	NM_134156	Mus musculus actinin, alpha 1	1.420	1.593
Kif7	NM_001291222	Mus musculus kinesin family member 7	0.306	1.592
Chgb	NM_007694	Mus musculus chromogranin B	0.305	1.592
Ecm1	NM_007899	Mus musculus extracellular matrix protein 1	0.854	1.591
Atp11b	NM_029570	Mus musculus ATPase, class VI, type 11B	0.304	1.590
Vmn2r3	NM_001104614	Mus musculus vomeronasal 2, receptor 3	0.304	1.590
P3h1	NM_019782	Mus musculus prolyl 3-hydroxylase 1	0.853	1.590
Pros1	NM_011173	Mus musculus protein S	1.535	1.590
Cdc25b	NM_023117	Mus musculus cell division cycle 25B	0.853	1.589
Carns1	NM_134148	Mus musculus carnosine synthase 1	1.413	1.589
Oas1c	NM_033541	Mus musculus 2'-5' oligoadenylate synthetase 1C	0.852	1.589
Sec61a1	NM_016906	Mus musculus Sec61 alpha 1 subunit	1.529	1.587
Ankrd54	NM_144849	Mus musculus ankyrin repeat domain 54	0.850	1.587
Wtip	NM_207212	Mus musculus WT1-interacting protein	1.062	1.587
Cxcr6	NM_030712	Mus musculus chemokine receptor 6	0.850	1.587
Pdgfra	NM_011058	Mus musculus platelet derived growth factor receptor, alpha polypeptide	1.408	1.587
Reps2	NM_178256	Mus musculus RALBP1 associated Eps domain containing protein 2	1.060	1.586
Shb	NM_001033306	Mus musculus src homology 2 domain-containing transforming protein B	0.849	1.586
Tmco6	NM_028036	Mus musculus transmembrane and coiled-coil domains 6	0.848	1.586
Ms4a6c	NM_028595	Mus musculus membrane-spanning 4-domains, subfamily A, member 6C	0.848	1.586
Arhgap9	NM_001285785	Mus musculus Rho GTPase activating protein 9	1.405	1.585
Reep4	NM_180588	Mus musculus receptor accessory protein 4	0.847	1.585
Ptpn18	NM_011206	Mus musculus protein tyrosine phosphatase, non-receptor type 18	1.524	1.585
Mall	NM_145532	Mus musculus mal, T cell differentiation protein-like	1.058	1.584
Tshz2	NM_080455	Mus musculus teashirt zinc finger family member 2	0.300	1.584
Ppfia4	NM_001144855	Mus musculus protein tyrosine phosphatase, receptor type, f polypeptide , interacting protein , alpha 4	0.845	1.583
Tmem97	NM_133706	Mus musculus transmembrane protein 97	1.056	1.583

Sgsm2	NM_197943	Mus musculus small G protein signaling modulator 2	1.401	1.583
Frmd4b	NM_145148	Mus musculus FERM domain containing 4B	1.056	1.583
Arpc5	NM_026369	Mus musculus actin related protein 2/3 complex, subunit 5	1.521	1.583
Pcdh17	NM_001013753	Mus musculus protocadherin 17	1.055	1.582
Pard3	NM_033620	Mus musculus par-3 family cell polarity regulator	1.055	1.582
Timeless	NM_011589	Mus musculus timeless circadian clock 1	0.299	1.582
Dapk2	NM_010019	Mus musculus death-associated protein kinase 2	1.398	1.581
Stmn3	NM_009133	Mus musculus stathmin-like 3	0.298	1.581
Cd86	NM_019388	Mus musculus CD86 antigen	0.842	1.581
Cd80	NM_009855	Mus musculus CD80 antigen	1.052	1.580
Shc2	NM_001024539	Mus musculus SHC transforming protein 2	0.841	1.580
Slc16a7	NM_011391	Mus musculus solute carrier family 16 , member 7	0.840	1.579
Exoc3l4	NM_028807	Mus musculus exocyst complex component 3-like 4	1.393	1.579
Eva1c	NM_027627	Mus musculus eva-1 homolog C	1.049	1.578
Hspa12a	NM_175199	Mus musculus heat shock protein 12A	1.391	1.578
Tm4sf1	NM_008536	Mus musculus transmembrane 4 superfamily member 1	1.225	1.577
Gadd45b	NM_008655	Mus musculus growth arrest and DNA-damage-inducible 45 beta	1.048	1.577
Mff	NM_029409	Mus musculus mitochondrial fission factor	1.224	1.577
Ncoa7	NM_172495	Mus musculus nuclear receptor coactivator 7	0.295	1.576
Vsig10	NM_001033311	Mus musculus V-set and immunoglobulin domain containing 10	1.045	1.576
Heg1	NM_175256	Mus musculus HEG homolog 1	1.045	1.576
Cachd1	NM_198037	Mus musculus cache domain containing 1	0.835	1.575
Sept5	NM_213614	Mus musculus septin 5	1.044	1.575
Bcat1	NM_007532	Mus musculus branched chain aminotransferase 1, cytosolic	0.293	1.574
Lmo2	NM_008505	Mus musculus LIM domain only 2	1.040	1.572
Cd34	NM_133654	Mus musculus CD34 antigen	1.215	1.572
Hmox1	NM_010442	Mus musculus heme oxygenase 1	1.380	1.572
B3gnt8	NM_146184	Mus musculus UDP-GlcNAc:betaGal beta-1,3-N-acetylglucosaminyltransferase 8	0.831	1.572
Cib2	NM_019686	Mus musculus calcium and integrin binding family member 2	1.379	1.571
Akt1	NM_009652	Mus musculus thymoma viral proto-oncogene 1	1.038	1.571
Paqr6	NM_198410	Mus musculus progestin and adipoQ receptor family member VI	0.291	1.571
Arhgef19	NM_172520	Mus musculus Rho guanine nucleotide exchange factor 19	1.038	1.570
Fat1	NM_001081286	Mus musculus FAT tumor suppressor homolog 1	1.377	1.570
Lpar1	NM_010336	Mus musculus lysophosphatidic acid receptor 1	1.037	1.570
Dennd2d	NM_028110	Mus musculus DENN/MADD domain containing 2D	0.290	1.570
Dst	NM_010081	Mus musculus dystonin	0.829	1.569
Tbx15	NM_009323	Mus musculus T-box 15	0.290	1.569
Samd14	NM_146025	Mus musculus sterile alpha motif domain containing 14	0.828	1.569
Zfp52	NM_144515	Mus musculus zinc finger protein 52	0.828	1.569
Mgl2	NM_145137	Mus musculus macrophage galactose N-acetyl-galactosamine specific lectin 2	0.828	1.569
Rnf26	NM_153762	Mus musculus ring finger protein 26	0.828	1.569
Fap	NM_007986	Mus musculus fibroblast activation protein	1.373	1.568
Arl4a	NM_001039515	Mus musculus ADP-ribosylation factor-like 4A	1.033	1.567
Mageh1	NM_023788	Mus musculus melanoma antigen, family H, 1	1.033	1.567
Dgkg	NM_138650	Mus musculus diacylglycerol kinase, gamma	0.288	1.567
Arhgap30	NM_001005508	Mus musculus Rho GTPase activating protein 30	0.826	1.567
C2cd3	NM_001017985	Mus musculus C2 calcium-dependent domain containing 3	0.826	1.567
Tmem98	NM_029537	Mus musculus transmembrane protein 98	1.370	1.567
Glis3	NM_175459	Mus musculus GLIS family zinc finger 3	0.288	1.566
Copg1	NM_201244	Mus musculus coatomer protein complex, subunit gamma 1	0.824	1.566
Renbp	NM_023132	Mus musculus renin binding protein	0.824	1.566
Dclre1c	NM_146114	Mus musculus DNA cross-link repair 1C, PSO2 homolog	0.824	1.566
Nucb2	NM_001130479	Mus musculus nucleobindin 2	1.030	1.565
Rgs19	NM_026446	Mus musculus regulator of G-protein signaling 19	1.030	1.565
Nradd	NM_026012	Mus musculus neurotrophin receptor associated death domain	0.824	1.565
Tg	NM_009375	Mus musculus thyroglobulin	0.823	1.565
Arhgap24	NM_146161	Mus musculus Rho GTPase activating protein 24	1.366	1.565
Gm2016	NM_001122662	Mus musculus predicted gene 2016	0.823	1.565
Pcdh9	NM_001271799	Mus musculus protocadherin 9	0.823	1.565
Prr5l	NM_001083810	Mus musculus proline rich 5 like	0.287	1.564
Ptma	NM_008972	Mus musculus prothymosin alpha	1.481	1.564
Ampd2	NM_028779	Mus musculus adenosine monophosphate deaminase 2	1.363	1.563
Ndnf	NM_172399	Mus musculus neuron-derived neurotrophic factor	0.821	1.563
Ctsz	NM_022325	Mus musculus cathepsin Z	1.477	1.562
Plekha6	NM_182930	Mus musculus pleckstrin homology domain containing, family A member 6	1.361	1.562
Sdk1	NM_177879	Mus musculus sidekick homolog 1	0.820	1.562
Ms4a6b	NM_027209	Mus musculus membrane-spanning 4-domains, subfamily A, member 6B	0.820	1.562
Ptrf	NM_008986	Mus musculus polymerase I and transcript release factor	1.198	1.562
Prcp	NM_028243	Mus musculus prolylcarboxypeptidase	1.360	1.562
Selm	NM_053267	Mus musculus selenoprotein M	1.476	1.562
Zfp30	NM_013705	Mus musculus zinc finger protein 30	1.359	1.561
Stxbp1	NM_001113569	Mus musculus syntaxin binding protein 1	1.357	1.560
Fbn2	NM_010181	Mus musculus fibrillin 2	0.817	1.560
Sec16b	NM_033354	Mus musculus SEC16 homolog B	1.022	1.560
Sh3bp4	NM_133816	Mus musculus SH3-domain binding protein 4	1.022	1.560
Trim30a	NM_009099	Mus musculus tripartite motif-containing 30A	1.022	1.560
Ece1	NM_199307	Mus musculus endothelin converting enzyme 1	1.471	1.559
Tiam2	NM_011878	Mus musculus T cell lymphoma invasion and metastasis 2	0.816	1.559
Zfp251	NM_001007568	Mus musculus zinc finger protein 251	0.283	1.559
Chpf2	NM_133913	Mus musculus chondroitin polymerizing factor 2	1.020	1.558
Ifih1	NM_027835	Mus musculus interferon induced with helicase C domain 1	1.020	1.558
Slfn2	NM_011408	Mus musculus schlafen 2	1.020	1.558
Vrk2	NM_001252447	Mus musculus vaccinia related kinase 2	1.019	1.558
Ifi204	NM_008329	Mus musculus interferon activated gene 204	1.467	1.558
Ms4a7	NM_001276398	Mus musculus membrane-spanning 4-domains, subfamily A, member 7	1.351	1.557
Dpp7	NM_031843	Mus musculus dipeptidylpeptidase 7	1.349	1.556

Zdhhc24	NM_027476	Mus musculus zinc finger, DHHC domain containing 24	0.813	1.556
Armcx4	NM_001202500	Mus musculus armadillo repeat containing, X-linked 4	1.016	1.556
C1qtnf5	NM_001040632	Mus musculus C1q and tumor necrosis factor related protein 5	1.015	1.555
Susd4	NM_144796	Mus musculus sushi domain containing 4	0.809	1.553
Slamf9	NM_029612	Mus musculus SLAM family member 9	1.343	1.553
Fgf6	NM_010204	Mus musculus fibroblast growth factor 6	1.010	1.552
Rcn1	NM_009037	Mus musculus reticulocalbin 1	1.455	1.552
Mcm6	NM_008567	Mus musculus minichromosome maintenance deficient 6	1.340	1.552
Rnf152	NM_178779	Mus musculus ring finger protein 152	0.277	1.551
Hcst	NM_011827	Mus musculus hematopoietic cell signal transducer	0.806	1.551
Ccnd2	NM_009829	Mus musculus cyclin D2	1.337	1.550
Mrps6	NM_080456	Mus musculus mitochondrial ribosomal protein S6	1.451	1.550
2210407C18Rik	NM_144544	Mus musculus RIKEN cDNA 2210407C18 gene	1.336	1.550
Haus8	NM_001163042	Mus musculus 4HAUS augmin-like complex, subunit 8	1.335	1.549
Rab8b	NM_173413	Mus musculus RAB8B, member RAS oncogene family	0.804	1.549
Ubtd2	NM_173784	Mus musculus ubiquitin domain containing 2	1.006	1.549
Zbp1	NM_001139519	Mus musculus Z-DNA binding protein 1	0.803	1.549
Sh3gl3	NM_017400	Mus musculus SH3-domain GRB2-like 3	0.803	1.548
Laptm4b	NM_033521	Mus musculus lysosomal-associated protein transmembrane 4B	1.174	1.548
Tspan6	NM_019656	Mus musculus tetraspanin 6	1.334	1.548
Fam228b	NM_175431	Mus musculus family with sequence similarity 228, member B	0.802	1.547
Nxn	NM_008750	Mus musculus nucleoredoxin	1.445	1.547
Gm5431	NM_001024230	Mus musculus predicted gene 5431	0.274	1.547
Nt5c	NM_015807	Mus musculus 5',3'-nucleotidase, cytosolic	1.002	1.547
Rhox8	NM_001004193	Mus musculus reproductive homeobox 8	1.612	1.545
Fam122b	NM_001166583	Mus musculus family with sequence similarity 122, member B	0.273	1.545
Pcdhga9	NM_033592	Mus musculus protocadherin gamma subfamily A, 9	1.441	1.545
Wif1	NM_011915	Mus musculus Wnt inhibitory factor 1	1.327	1.545
Mtmr12	NM_172958	Mus musculus myotubularin related protein 12	0.273	1.545
Srd5a3	NM_020611	Mus musculus steroid 5 alpha-reductase 3	1.000	1.545
Irf9	NM_001159417	Mus musculus interferon regulatory factor 9	1.326	1.545
Tpm3	NM_001253738	Mus musculus tropomyosin 3, gamma	1.167	1.544
Zfp618	NM_028326	Mus musculus zinc finger protein 618	0.797	1.543
Itga8	NM_001001309	Mus musculus integrin alpha 8	0.996	1.543
Oasl2	NM_011854	Mus musculus 2'-5' oligoadenylate synthetase-like 2	0.996	1.543
Tmem43	NM_028766	Mus musculus transmembrane protein 43	1.322	1.543
Megf8	NM_001160400	Mus musculus multiple EGF-like-domains 8	1.322	1.542
Myef2	NM_001162417	Mus musculus myelin basic protein expression factor 2, repressor	0.795	1.542
Irgm2	NM_019440	Mus musculus immunity-related GTPase family M member 2	1.320	1.542
Ddx25	NM_013932	Mus musculus DEAD box polypeptide 25	1.434	1.542
Susd2	NM_027890	Mus musculus sushi domain containing 2	0.270	1.541
Axl	NM_009465	Mus musculus AXL receptor tyrosine kinase	1.160	1.541
Nanp	NM_026086	Mus musculus N-acetylneuraminic acid phosphatase	0.793	1.541
B020031M17Rik	NM_001033769	Mus musculus RIKEN cDNA B020031M17 gene	1.160	1.541
Tubb2b	NM_023716	Mus musculus tubulin, beta 2B class IIB	0.993	1.540
Aim2	NM_001013779	Mus musculus absent in melanoma 2	0.793	1.540
Hoxa7	NM_010455	Mus musculus homeobox A7	0.792	1.540
Sept1	NM_017461	Mus musculus septin 1	0.792	1.540
Csf2ra	NM_009970	Mus musculus colony stimulating factor 2 receptor, alpha, low-affinity	1.316	1.540
Slitrk4	NM_178740	Mus musculus SLIT and NTRK-like family, member 4	0.792	1.539427
Nr5a2	NM_030676	Mus musculus nuclear receptor subfamily 5, group A, member 2	0.792	1.539226
Dagla	NM_198114	Mus musculus diacylglycerol lipase, alpha	0.991	1.539039
Kazald1	NM_178929	Mus musculus Kazal-type serine peptidase inhibitor domain 1	0.990	1.538693
Gm16404	NM_001220497	Mus musculus predicted gene 16404	0.791	1.538582
Rara	NM_001177302	Mus musculus retinoic acid receptor, alpha	0.990	1.538391
Zrsr2	NM_178794	Mus musculus zinc finger , RNA binding motif and serine/arginine rich 2	0.790	1.538143
Cyp4f13	NM_130882	Mus musculus cytochrome P450, family 4, subfamily f, polypeptide 13	0.790	1.538087
Armcx3	NM_027870	Mus musculus armadillo repeat containing, X-linked 3	0.989	1.537846
Rbm14	NM_019869	Mus musculus RNA binding motif protein 14	0.268	1.537836
Cep41	NM_031998	Mus musculus centrosomal protein 41	0.267	1.537038
Dennd2a	NM_172477	Mus musculus DENN/MADD domain containing 2A	1.310	1.536601
H2-Aa	NM_010378	Mus musculus histocompatibility 2, class II antigen A, alpha	0.986	1.536159
Acrbp	NM_016845	Mus musculus proacrosin binding protein	0.788	1.535882
Naaa	NM_025972	Mus musculus N-acylethanolamine acid amidase	1.308	1.535475
Sec14l2	NM_144520	Mus musculus SEC14-like 2	0.266	1.535365
Acaca	NM_133360	Mus musculus acetyl-Coenzyme A carboxylase alpha	1.307	1.535091
Mpl	NM_001122949	Mus musculus myeloproliferative leukemia virus oncogene	0.786	1.535050
Prr14l	NM_194340	Mus musculus proline rich 14-like	0.984	1.534798
Cttnbp2nl	NM_030249	Mus musculus CTTNBP2 N-terminal like	0.984	1.534555
Osbpl5	NM_024289	Mus musculus oxysterol binding protein-like 5	1.418	1.534265
Serinc5	NM_172588	Mus musculus serine incorporator 5	1.305	1.534148
Sfxn2	NM_053196	Mus musculus sideroflexin 2	0.983	1.534090
Tmem151a	NM_001001885	Mus musculus transmembrane protein 151A	0.785	1.533492
Man2a1	NM_008549	Mus musculus mannosidase 2, alpha 1	1.304	1.533380
Igsf3	NM_207205	Mus musculus immunoglobulin superfamily, member 3	0.982	1.533117
Col6a1	NM_009933	Mus musculus collagen, type VI, alpha 1	1.415	1.532947
Tnfsf13b	NM_033622	Mus musculus tumor necrosis factor superfamily, member 13b	0.783	1.532021
Pvrl2	NM_008990	Mus musculus poliovirus receptor-related 2	0.979	1.531218
Ptpn6	NM_001077705	Mus musculus protein tyrosine phosphatase, non-receptor type 6	0.979	1.531037
Ahi1	NM_026203	Mus musculus Abelson helper integration site 1	0.978	1.530596
Rin2	NM_028724	Mus musculus Ras and Rab interactor 2	1.298	1.530480
Gcm1	NM_008103	Mus musculus glial cells missing homolog 1	1.576	1.530359
Htr7	NM_008315	Mus musculus 5-hydroxytryptamine receptor 7	0.781	1.530241
Phactr1	NM_001005748	Mus musculus phosphatase and actin regulator 1	0.977	1.530216
H2-M3	NM_013819	Mus musculus histocompatibility 2, M region locus 3	0.977	1.529930
Irf2bp2	NM_001164598	Mus musculus interferon regulatory factor 2 binding protein 2	1.407	1.529390

Clip2	NM_009990	Mus musculus CAP-GLY domain containing linker protein 2	0.779	1.529252
2410089E03Rik	NM_001162906	Mus musculus RIKEN cDNA 2410089E03 gene	0.779	1.529219
Plxdc2	NM_026162	Mus musculus plexin domain containing 2	1.406	1.528775
H2-K1	NM_001001892	Mus musculus histocompatibility 2, K1, K region	1.138	1.528047
Slc26a11	NM_178743	Mus musculus solute carrier family 26, member 11	0.974	1.527937
Stbd1	NM_175096	Mus musculus starch binding domain 1	0.261	1.527330
Arid5a	NM_001290726	Mus musculus AT rich interactive domain 5A	1.291	1.527172
Pign	NM_013784	Mus musculus phosphatidylinositol glycan anchor biosynthesis, class N	0.776	1.526843
Tgfbr2	NM_009371	Mus musculus transforming growth factor, beta receptor II	1.402	1.526825
Syk	NM_011518	Mus musculus spleen tyrosine kinase	1.290	1.526677
Ugdh	NM_009466	Mus musculus UDP-glucose dehydrogenase	1.290	1.526488
Cks1b	NM_016904	Mus musculus CDC28 protein kinase 1b	1.289	1.526187
Ppap2b	NM_080555	Mus musculus phosphatidic acid phosphatase type 2B	1.400	1.526081
Slc7a4	NM_144852	Mus musculus solute carrier family 7 , member 4	0.971	1.525672
Rell1	NM_145923	Mus musculus RELT-like 1	1.288	1.525557
Cxcl1	NM_008176	Mus musculus chemokine ligand 1	0.775	1.525555
Kctd11	NM_153143	Mus musculus potassium channel tetramerisation domain containing 11	1.287	1.525274
Mr1	NM_008209	Mus musculus major histocompatibility complex, class I-related	0.774	1.524654
Adam32	NM_153397	Mus musculus a disintegrin and metallopeptidase domain 32	0.258	1.524201
Ogg1	NM_010957	Mus musculus 8-oxoguanine DNA-glycosylase 1	0.968	1.523994
Crebzf	NM_145151	Mus musculus CREB/ATF bZIP transcription factor	1.285	1.523925
Dock7	NM_026082	Mus musculus dedicator of cytokinesis 7	0.258	1.523013
Olfr699	NM_001011862	Mus musculus olfactory receptor 699	0.770	1.522196
Map1s	NM_173013	Mus musculus microtubule-associated protein 1S	0.965	1.521994
Gsn	NM_146120	Mus musculus gelsolin	1.556	1.521886
Nsmf	NM_001039386	Mus musculus NMDA receptor synaptonuclear signaling and neuronal migration factor	1.390	1.521319
Yy2	NM_001098723	Mus musculus Yy2 transcription factor	0.769	1.521129
Fam171a1	NM_001081161	Mus musculus family with sequence similarity 171, member A1	0.769	1.520831
AI429214	NM_001039220	Mus musculus expressed sequence AI429214	0.256	1.520620
Cd82	NM_001271432	Mus musculus CD82 antigen	1.388	1.520468
Foxp1	NM_053202	Mus musculus forkhead box P1	0.963	1.520410
Slc39a11	NM_027216	Mus musculus solute carrier family 39 , member 11	0.963	1.520382
Lima1	NM_023063	Mus musculus LIM domain and actin binding 1	1.277	1.520255
Clcn6	NM_011929	Mus musculus chloride channel 6	0.768	1.520203
Notch1	NM_008714	Mus musculus notch 1	1.387	1.520086
Ptgir	NM_008967	Mus musculus prostaglandin I receptor	0.962	1.519742
Slc38a4	NM_027052	Mus musculus solute carrier family 38, member 4	0.767	1.519683
Ctsh	NM_007801	Mus musculus cathepsin H	0.961	1.519661
Synm	NM_201639	Mus musculus synemin, intermediate filament protein	1.275	1.519131
Ubash3b	NM_176860	Mus musculus ubiquitin associated and SH3 domain containing, B	0.960	1.518839
Zeb2	NM_015753	Mus musculus zinc finger E-box binding homeobox 2	0.766	1.518665
Samd5	NM_177271	Mus musculus sterile alpha motif domain containing 5	0.960	1.518489
Fads1	NM_146094	Mus musculus fatty acid desaturase 1	0.959	1.518361
Fgfr4	NM_008011	Mus musculus fibroblast growth factor receptor 4	0.766	1.518256
Tlr2	NM_011905	Mus musculus toll-like receptor 2	0.959	1.517961
Pcdhb16	NM_053141	Mus musculus protocadherin beta 16	0.765	1.517858
Pgbd5	NM_171824	Mus musculus piggyBac transposable element derived 5	0.959	1.517803
Hist1h2ao	NM_001177544	Mus musculus histone cluster 1, H2ao	1.382	1.517549
Carhsp1	NM_025821	Mus musculus calcium regulated heat stable protein 1	1.382	1.517502
Stmn4	NM_019675	Mus musculus stathmin-like 4	0.958	1.517268
Tnfrsf19	NM_013869	Mus musculus tumor necrosis factor receptor superfamily, member 19	0.957	1.516859
Limd2	NM_172397	Mus musculus LIM domain containing 2	1.270	1.516789
Capn5	NM_007602	Mus musculus calpain 5	0.957	1.516643
Bcl2	NM_009741	Mus musculus B cell leukemia/lymphoma 2	1.269	1.516344
Tox3	NM_172913	Mus musculus TOX high mobility group box family member 3	0.956	1.516301
Cfap45	NM_027972	Mus musculus cilia and flagella associated protein 45	0.763	1.516277
Chn2	NM_023543	Mus musculus chimerin 2	0.763	1.516133
Taok3	NM_001199685	Mus musculus TAO kinase 3	0.762	1.515675
Glb1	NM_009752	Mus musculus galactosidase, beta 1	1.267	1.515253
Aoah	NM_012054	Mus musculus acyloxyacyl hydrolase	0.761	1.514743
Ctps2	NM_018737	Mus musculus cytidine 5'-triphosphate synthase 2	0.760	1.514083
Frrs1	NM_009146	Mus musculus ferric-chelate reductase 1	0.953	1.514073
Rabgef1	NM_019983	Mus musculus RAB guanine nucleotide exchange factor 1	0.251	1.513968
Olfml2b	NM_177068	Mus musculus olfactomedin-like 2B	1.374	1.513861
Rnf41	NM_001164237	Mus musculus ring finger protein 41	0.760	1.513846
Zfp9	NM_011763	Mus musculus zinc finger protein 9	0.251	1.513760
Foxp4	NM_001110824	Mus musculus forkhead box P4	1.264	1.513704
Lhfp	NM_175386	Mus musculus lipoma HMGIC fusion partner	1.373	1.513522
Pip4k2a	NM_008845	Mus musculus phosphatidylinositol-5-phosphate 4-kinase, type II, alpha	0.251	1.513444
Cd68	NM_001291058	Mus musculus CD68 antigen	0.952	1.513424
Rab39	NM_175562	Mus musculus RAB39, member RAS oncogene family	0.251	1.513394
Psip1	NM_133948	Mus musculus PC4 and SFRS1 interacting protein 1	0.951	1.512689
Casp1	NM_009807	Mus musculus caspase 1	0.951	1.512688
Gale	NM_178389	Mus musculus galactose-4-epimerase, UDP	0.950	1.512217
Myh10	NM_175260	Mus musculus myosin, heavy polypeptide 10, non-muscle	1.261	1.512153
Scn1b	NM_011322	Mus musculus sodium channel, voltage-gated, type I, beta	0.949	1.511601
Bcr	NM_001081412	Mus musculus breakpoint cluster region	1.260	1.511598
Nr1i3	NM_009803	Mus musculus nuclear receptor subfamily 1, group I, member 3	0.757	1.511590
Uap1l1	NM_001033293	Mus musculus UDP-N-acteylglucosamine pyrophosphorylase 1-like 1	0.949	1.511416
Was	NM_009515	Mus musculus Wiskott-Aldrich syndrome homolog	0.949	1.511172
Atat1	NM_028476	Mus musculus alpha tubulin acetyltransferase 1	0.249	1.510564
Epb4.1l2	NM_001199265	Mus musculus erythrocyte protein band 4.1-like 2	1.365	1.510069
Chsy1	NM_001081163	Mus musculus chondroitin sulfate synthase 1	1.256	1.509872
Rapgef1	NM_001039087	Mus musculus Rap guanine nucleotide exchange factor 1	0.248	1.509774
Tuba1a	NM_011653	Mus musculus tubulin, alpha 1A	1.105	1.509731
Lyl1	NM_008535	Mus musculus lymphoblastomic leukemia 1	1.256	1.509706

Kdelr2	NM_025841	Mus musculus KDEL endoplasmic reticulum protein retention receptor 2	1.255	1.509099
Mb21d1	NM_173386	Mus musculus Mab-21 domain containing 1	0.754	1.508780
Wdr90	NM_001163766	Mus musculus WD repeat domain 90	0.752	1.507687
Alox5ap	NM_009663	Mus musculus arachidonate 5-lipoxygenase activating protein	1.251	1.507291
Tle1	NM_011599	Mus musculus transducin-like enhancer of split 1, homolog of Drosophila E	1.250	1.507017
Tubgcp6	NM_001163319	Mus musculus tubulin, gamma complex associated protein 6	0.246	1.506963
Zfp423	NM_033327	Mus musculus zinc finger protein 423	0.942	1.506892
Klk4	NM_019928	Mus musculus kallikrein related-peptidase 4	0.246	1.506562
Mad1l1	NM_010752	Mus musculus MAD1 mitotic arrest deficient 1-like 1	0.941	1.506263
Usp53	NM_133857	Mus musculus ubiquitin specific peptidase 53	0.750	1.505932
Smc2	NM_008017	Mus musculus structural maintenance of chromosomes 2	0.750	1.505790
Dennd6a	NM_001285467	Mus musculus DENN/MADD domain containing 6A	0.245	1.505588
D1Ertd622e	NM_133825	Mus musculus DNA segment, Chr 1, ERATO Doi 622, expressed	0.938	1.504474
Endod1	NM_028013	Mus musculus endonuclease domain containing 1	0.748	1.504194
Rassf5	NM_018750	Mus musculus Ras association domain family member 5	0.936	1.503215
Dnajb3	NM_008299	Mus musculus DnaJ homolog, subfamily B, member 3	0.746	1.502876
Arhgdib	NM_007486	Mus musculus Rho, GDP dissociation inhibitor beta	1.241	1.502526
Gm12942	NM_001099319	Mus musculus predicted gene 12942	0.935	1.502326
Rnase2a	NM_053113	Mus musculus ribonuclease, RNase A family, 2A	0.242	1.501716
Il1r1	NM_008362	Mus musculus interleukin 1 receptor, type I	0.934	1.501661
Ssh3	NM_198113	Mus musculus slingshot homolog 3	0.933	1.501029
Clec2d	NM_053109	Mus musculus C-type lectin domain family 2, member d	1.238	1.500935
Srd5a1	NM_175283	Mus musculus steroid 5 alpha-reductase 1	0.744	1.500926
Snx20	NM_027840	Mus musculus sorting nexin 20	0.932	1.500116
Sox10	NM_011437	Mus musculus SRY -box 10	0.743	1.500069
Wipf1	NM_001289722	Mus musculus WAS/WASL interacting protein family, member 1	0.743	1.500063

Supplementary Table 4. Up or down-regulated genes in splenic monocytes of 5/6Nx mice

Gene Symbol	Genbank accession	Description	5/6Nx / Sham	
			Z-score	Fold change
Ltf	NM_008522	lactotransferrin	-6.617	0.000353
Klk1b5	NM_008456	kallikrein 1-related peptidase b5	-5.782	0.000961
Cd19	NM_001357091	CD19 antigen	-5.591	0.001504
Hbb-bt	NM_008220	hemoglobin, beta adult t chain	-5.570	0.001331
Fcmr	NM_026976	Fc fragment of IgM receptor	-5.474	0.001492
Chil3	NM_009892	chitinase-like 3	-5.458	0.001755
Bcl11a	NM_016707	B cell CLL/lymphoma 11A	-5.360	0.001596
Ngp	NM_008694	neutrophilic granule protein	-5.357	0.001603
Scd1	NM_009127	stearoyl-Coenzyme A desaturase 1	-5.256	0.001809
Camp	NM_009921	cathelicidin antimicrobial peptide	-4.862	0.003508
Fosb	NM_008036	FBJ osteosarcoma oncogene B	-4.777	0.003422
Cr2	NM_001368765	complement receptor 2	-4.627	0.004088
Cd19	NM_001357091	CD19 antigen	-4.619	0.004128
Smpd3	NM_021491	sphingomyelin phosphodiesterase 3, neutral	-4.520	0.020146
Srpk3	NM_019684	serine/arginine-rich protein specific kinase 3	-4.477	0.004603
Arsi	NM_001038499	arylsulfatase i	-4.470	0.021328
Fcer2a	NM_013517	Fc receptor, IgE, low affinity II, alpha polypeptide	-4.443	0.005089
Chst3	NM_016803	carbohydrate sulfotransferase 3	-4.401	0.005045
Grrp1	NM_001099296	glycine/arginine rich protein 1	-4.389	0.023391
Col3a1	NM_009930	collagen, type III, alpha 1	-4.380	0.023612
Pxdn	NM_181395	peroxidasin	-4.380	0.023621
Ptpn20	NM_008978	protein tyrosine phosphatase, non-receptor type 20	-4.334	0.024878
Fn1	NM_010233	fibronectin 1	-4.323	0.006557
Hpgd	NM_008278	hydroxyprostaglandin dehydrogenase 15	-4.290	0.005765
Cr2	NM_007758	complement receptor 2	-4.229	0.002121
Cpb1	NM_029706	carboxypeptidase B1	-4.229	0.006201
Klra17	NM_133203	killer cell lectin-like receptor, subfamily A, member 17	-4.184	0.029493
Ccm2l	NM_145536	cerebral cavernous malformation 2-like	-4.181	0.029598
Klhl14	NM_001081403	kelch-like 14	-4.177	0.029729
Ppfia2	NM_001359672	protein tyrosine phosphatase, receptor type, f polypeptide , interacting protein , alpha 2	-4.162	0.030249
Sfrp1	NM_013834	secreted frizzled-related protein 1	-4.160	0.030325
Acaca	NM_133360	acetyl-Coenzyme A carboxylase alpha	-4.156	0.030438
Trim10	NM_011280	tripartite motif-containing 10	-4.129	0.031407
Hspa12a	NM_175199	heat shock protein 12A	-4.124	0.031563
Rhd	NM_011270	Rh blood group, D antigen	-4.111	0.002525
Myh3	NM_001099635	myosin, heavy polypeptide 3, skeletal muscle, embryonic	-4.110	0.032073
Mrap	NM_029844	melanocortin 2 receptor accessory protein	-4.100	0.032439
Aldh1a7	NM_011921	aldehyde dehydrogenase family 1, subfamily A7	-4.100	0.032453
Adh1	NM_007409	alcohol dehydrogenase 1	-4.097	0.032567
A830018L16Rik	NM_177173	RIKEN cDNA A830018L16 gene	-4.096	0.032603
Vmn2r66	NM_001033878	vomeronasal 2, receptor 66	-4.083	0.033063
Efna1	NM_010107	ephrin A1	-4.065	0.033746
Hbb-b1	NM_001278161	hemoglobin, beta adult major chain	-4.055	0.002740
Pard3b	NM_001081050	par-3 family cell polarity regulator beta	-4.035	0.034943
Olfr684	NM_207249	olfactory receptor 684	-4.022	0.035462
Slit3	NM_011412	slit guidance ligand 3	-4.015	0.035723
Phgr1	NM_001145644	proline/histidine/glycine-rich 1	-4.007	0.036049
Hdac9	NM_001271386	histone deacetylase 9	-4.004	0.036168
Cd209a	NM_133238	CD209a antigen	-3.984	0.008323
Ahsp	NM_133245	alpha hemoglobin stabilizing protein	-3.971	0.003103
Cpa2	NM_001024698	carboxypeptidase A2, pancreatic	-3.951	0.038418
Serpinb10	NM_198028	serine peptidase inhibitor, clade B , member 10	-3.950	0.003199
Cd79a	NM_007655	CD79A antigen	-3.934	0.008833
Nynrin	NM_001040072	NYN domain and retroviral integrase containing	-3.931	0.039294
Ky	NM_024291	kyphoscoliosis peptidase	-3.930	0.039359
Ggt1	NM_008116	gamma-glutamyltransferase 1	-3.928	0.039413
Htr2c	NM_008312	5-hydroxytryptamine receptor 2C	-3.924	0.039603
Cd209e	NM_130905	CD209e antigen	-3.922	0.039687
Hbb-b2	NM_016956	hemoglobin, beta adult minor chain	-3.919	0.009002
Itih5	NM_172471	inter-alpha inhibitor H5	-3.917	0.039928
Fn1	NM_010233	fibronectin 1	-3.914	0.009048
Ppp1r14a	NM_026731	protein phosphatase 1, regulatory inhibitor subunit 14A	-3.912	0.040167
Kif9	NM_010628	kinesin family member 9	-3.909	0.040275
Ankrd42	NM_028665	ankyrin repeat domain 42	-3.907	0.040396
Baiap2l2	NM_177580	BAI1-associated protein 2-like 2	-3.907	0.040403

Ackr3	NM_001271607	atypical chemokine receptor 3	-3.903	0.003428
Adam23	NM_011780	a disintegrin and metallopeptidase domain 23	-3.889	0.041217
Olfr735	NM_001011754	olfactory receptor 735	-3.887	0.041292
Ackr2	NM_021609	atypical chemokine receptor 2	-3.881	0.003542
Slc5a11	NM_146198	solute carrier family 5 , member 11	-3.869	0.042143
Ttc39a	NM_153392	tetratricopeptide repeat domain 39A	-3.853	0.042952
Abat	NM_001170978	4-aminobutyrate aminotransferase	-3.833	0.043917
Cpa1	NM_025350	carboxypeptidase A1, pancreatic	-3.818	0.010158
F13a1	NM_028784	coagulation factor XIII, A1 subunit	-3.796	0.012085
Slc2a4	NM_009204	solute carrier family 2 , member 4	-3.775	0.046900
Spib	NM_019866	Spi-B transcription factor	-3.774	0.011281
Cd207	NM_144943	CD207 antigen	-3.772	0.047035
Hba-a1	NM_008218	hemoglobin alpha, adult chain 1	-3.772	0.012431
Klra17	NM_133203	killer cell lectin-like receptor, subfamily A, member 17	-3.770	0.010751
Cfh	NM_009888	complement component factor h	-3.742	0.011719
Nkpd1	NM_027116	NTPase, KAP family P-loop domain containing 1	-3.732	0.049257
Sult1a1	NM_133670	sulfotransferase family 1A, phenol-preferring, member 1	-3.724	0.004462
Gm1673	NM_001310547	predicted gene 1673	-3.670	0.012132
Reg2	NM_009043	regenerating islet-derived 2	-3.638	0.005060
Ms4a1	NM_007641	membrane-spanning 4-domains, subfamily A, member 1	-3.628	0.014682
Abca9	NM_147220	ATP-binding cassette, sub-family A , member 9	-3.625	0.012795
Stab2	NM_138673	stabilin 2	-3.623	0.012828
Try5	NM_001003405	trypsin 5	-3.610	0.013038
Cacna1i	NM_001044308	calcium channel, voltage-dependent, alpha 1I subunit	-3.603	0.013148
Bcl11a	NM_001242934	B cell CLL/lymphoma 11A	-3.571	0.014353
Gm9733	NM_001076679	predicted gene 9733	-3.557	0.014598
Egfbp2	NM_010115	epidermal growth factor binding protein type B	-3.543	0.005818
Cela3b	NM_026419	chymotrypsin-like elastase family, member 3B	-3.536	0.014241
Hdac9	NM_001271386	histone deacetylase 9	-3.535	0.014259
Col6a4	NM_026763	collagen, type VI, alpha 4	-3.486	0.015124
Nr4a1	NM_010444	nuclear receptor subfamily 4, group A, member 1	-3.467	0.015834
Ace	NM_009598	angiotensin I converting enzyme 1	-3.461	0.015581
Klf1	NM_010635	Kruppel-like factor 1	-3.458	0.006594
Ear2	NM_007895	eosinophil-associated, ribonuclease A family, member 2	-3.458	0.017898
Cr2	NM_001368765	complement receptor 2	-3.442	0.018219
Cldn5	NM_013805	claudin 5	-3.406	0.007121
Slc4a1	NM_011403	solute carrier family 4 , member 1	-3.404	0.007142
Bend5	NM_026279	BEN domain containing 5	-3.398	0.016804
Iqcg	NM_178378	IQ motif containing G	-3.396	0.016860
Gp2	NM_025989	glycoprotein 2	-3.385	0.007350
Pi16	NM_023734	peptidase inhibitor 16	-3.379	0.018048
S100a9	NM_001281852	S100 calcium binding protein A9	-3.373	0.019758
Cd177	NM_026862	CD177 antigen	-3.372	0.007485
Smim5	NM_183259	small integral membrane protein 5	-3.346	0.007777
Myl4	NM_001355754	myosin, light polypeptide 4	-3.336	0.018114
Ctrb1	NM_025583	chymotrypsinogen B1	-3.326	0.019215
Cela2a	NM_007919	chymotrypsin-like elastase family, member 2A	-3.314	0.019498
Amy2a5	NM_001042711	amylase 2a5	-3.302	0.019764
Emid1	NM_080595	EMI domain containing 1	-3.299	0.018942
Sh3pxd2a	NM_008018	SH3 and PX domains 2A	-3.295	0.019014
Mpo	NM_010824	myeloperoxidase	-3.279	0.020311
Cd79b	NM_008339	CD79B antigen	-3.278	0.022038
Hba-a1	NM_008218	hemoglobin alpha, adult chain 1	-3.275	0.019947
Nhsl2	NM_001163610	NHS-like 2	-3.269	0.019629
Bank1	NM_001033350	B cell scaffold protein with ankyrin repeats 1	-3.268	0.020586
Cpa2	NM_001024698	carboxypeptidase A2, pancreatic	-3.261	0.019806
Fn1	NM_001276413	fibronectin 1	-3.245	0.009030
Itgad	NM_001029872	integrin, alpha D	-3.230	0.009227
Skint3	NM_001102474	selection and upkeep of intraepithelial T cells 3	-3.209	0.021094
Cyp39a1	NM_018887	cytochrome P450, family 39, subfamily a, polypeptide 1	-3.197	0.021396
Stfa2l1	NM_173869	stefin A2 like 1	-3.179	0.009949
Ebf1	NM_001362575	early B cell factor 1	-3.157	0.022443
Nhsl2	NM_001163610	NHS-like 2	-3.156	0.022486
Snn	NM_009223	stannin	-3.153	0.023599
Hba-a2	NM_001083955	hemoglobin alpha, adult chain 2	-3.149	0.023223
Aqp1	NM_007472	aquaporin 1	-3.149	0.010399
Pax5	NM_008782	paired box 5	-3.141	0.010520
Adamdec1	NM_021475	ADAM-like, decysin 1	-3.121	0.023426
Tspan18	NM_183180	tetraspanin 18	-3.114	0.010949
Slc25a17	NM_011399	solute carrier family 25 , member 17	-3.112	0.024776
Serpinb1a	NM_025429	serine peptidase inhibitor, clade B, member 1a	-3.111	0.026780

Jhy	NM_001357360	junctional cadherin complex regulator	-3.099	0.024066
Lrg1	NM_029796	leucine-rich alpha-2-glycoprotein 1	-3.080	0.024624
Apoc1	NM_007469	apolipoprotein C-I	-3.069	0.024952
Retnlg	NM_181596	resistin like gamma	-3.065	0.011752
Cd24a	NM_009846	CD24a antigen	-3.059	0.026414
Sult1a1	NM_133670	sulfotransferase family 1A, phenol-preferring, member 1	-3.058	0.011879
Ramp2	NM_019444	receptor activity modifying protein 2	-3.056	0.011919
Slc38a5	NM_172479	solute carrier family 38, member 5	-3.056	0.011921
Ephx1	NM_010145	epoxide hydrolase 1, microsomal	-3.054	0.026572
0610040J01Rik	NM_029554	RIKEN cDNA 0610040J01 gene	-3.049	0.025569
Tiam2	NM_011878	T cell lymphoma invasion and metastasis 2	-3.043	0.025743
Cmbl	NM_181588	carboxymethylenebutenolidase-like	-3.035	0.012295
Zfp318	NM_021346	zinc finger protein 318	-3.032	0.026091
Mrgpra2a	NM_001172588	MAS-related GPR, member A2A	-3.026	0.012462
Fcnb	NM_010190	ficolin B	-3.024	0.012487
Dtx1	NM_008052	deltex 1, E3 ubiquitin ligase	-3.019	0.027681
Alas2	NM_009653	aminolevulinic acid synthase 2, erythroid	-3.008	0.012793
Mrgpra2b	NM_153101	MAS-related GPR, member A2B	-3.007	0.012801
Xkrx	NM_183319	X-linked Kx blood group related, X-linked	-2.986	0.027549
H2-DMb2	NM_010388	histocompatibility 2, class II, locus Mb2	-2.981	0.013306
Prss2	NM_009430	protease, serine 2	-2.975	0.027933
Nat8l	NM_001001985	N-acetyltransferase 8-like	-2.975	0.027937
Ifitm6	NM_001033632	interferon induced transmembrane protein 6	-2.965	0.031718
Acot1	NM_012006	acyl-CoA thioesterase 1	-2.953	0.013862
Hnmt	NM_080462	histamine N-methyltransferase	-2.926	0.029610
Cpm	NM_027468	carboxypeptidase M	-2.923	0.029718
Amz1	NM_173405	archaelysin family metallopeptidase 1	-2.922	0.031060
Cx3cr1	NM_009987	chemokine receptor 1	-2.920	0.033396
4931408C20Rik	NM_001033764	RIKEN cDNA 4931408C20 gene	-2.910	0.014764
H2-Ob	NM_010389	histocompatibility 2, O region beta locus	-2.906	0.014847
Kmo	NM_133809	kynurenine 3-monooxygenase	-2.902	0.031840
Cd209b	NM_026972	CD209b antigen	-2.899	0.015002
Lifr	NM_001113386	LIF receptor alpha	-2.897	0.015055
Cd55	NM_010016	CD55 molecule, decay accelerating factor for complement	-2.895	0.030757
Gpr141b	NM_175688	G protein-coupled receptor 141B	-2.887	0.015267
Mogat2	NM_177448	monoacylglycerol O-acyltransferase 2	-2.885	0.015313
Bcl11a	NM_001242934	B cell CLL/lymphoma 11A	-2.883	0.015367
Lifr	NM_001358593	LIF receptor alpha	-2.878	0.015479
Nrm	NM_134122	nurim	-2.871	0.031641
Vpreb3	NM_009514	pre-B lymphocyte gene 3	-2.868	0.033127
Crip2	NM_024223	cysteine rich protein 2	-2.864	0.033286
Cd55	NM_010016	CD55 molecule, decay accelerating factor for complement	-2.863	0.031942
Eps8	NM_007945	epidermal growth factor receptor pathway substrate 8	-2.860	0.032060
Sparc	NM_009242	secreted acidic cysteine rich glycoprotein	-2.854	0.016047
Prtn3	NM_011178	proteinase 3	-2.852	0.016083
Apoe	NM_009696	apolipoprotein E	-2.850	0.033254
Kel	NM_032540	Kell blood group	-2.848	0.016191
Fam107a	NM_001360337	family with sequence similarity 107, member A	-2.836	0.033825
Egfbp2	NM_010115	epidermal growth factor binding protein type B	-2.825	0.016739
Cx3cr1	NM_009987	chemokine receptor 1	-2.820	0.035063
Ell3	NM_145973	elongation factor RNA polymerase II-like 3	-2.814	0.033877
F5	NM_007976	coagulation factor V	-2.812	0.033952
Slc8a3	NM_080440	solute carrier family 8 , member 3	-2.810	0.029854
Kmo	NM_133809	kynurenine 3-monooxygenase	-2.796	0.034627
Stab2	NM_138673	stabilin 2	-2.794	0.034692
H2-Ob	NM_010389	histocompatibility 2, O region beta locus	-2.788	0.034973
Kel	NM_032540	Kell blood group	-2.786	0.017716
Ptpn14	NM_008976	protein tyrosine phosphatase, non-receptor type 14	-2.782	0.017835
Cela3a	NM_001126318	chymotrypsin-like elastase family, member 3A	-2.770	0.018136
Plvap	NM_032398	plasmalemma vesicle associated protein	-2.768	0.035808
Napsa	NM_008437	napsin A aspartic peptidase	-2.767	0.039896
Pcdh12	NM_017378	protocadherin 12	-2.764	0.018295
Itga8	NM_001001309	integrin alpha 8	-2.724	0.019412
Egfl7	NM_198724	EGF-like domain 7	-2.723	0.037796
Syt10	NM_018803	synaptotagmin X	-2.721	0.019504
Tppp3	NM_026481	tubulin polymerization-promoting protein family member 3	-2.713	0.042500
Bri3bp	NM_029752	Bri3 binding protein	-2.706	0.042813
Cd209d	NM_130904	CD209d antigen	-2.699	0.040498
Aldh2	NM_009656	aldehyde dehydrogenase 2, mitochondrial	-2.697	0.043257
Gypa	NM_010369	glycophorin A	-2.696	0.020238
Car2	NM_009801	carbonic anhydrase 2	-2.695	0.040717

Pdlim1	NM_016861	PDZ and LIM domain 1	-2.680	0.044127
Hes5	NM_010419	hes family bHLH transcription factor 5	-2.673	0.040141
Spns2	NM_153060	spinster homolog 2	-2.669	0.040325
Pnliprp1	NM_018874	pancreatic lipase related protein 1	-2.662	0.021259
Grn	NM_008175	granulin	-2.653	0.042181
Tsc22d3	NM_010286	TSC22 domain family, member 3	-2.641	0.046167
Btla	NM_001037719	B and T lymphocyte associated	-2.632	0.043858
Add3	NM_013758	adducin 3	-2.632	0.046677
Cd209a	NM_133238	CD209a antigen	-2.630	0.022293
Kcne3	NM_020574	potassium voltage-gated channel, Isk-related subfamily, gene 3	-2.630	0.042262
Wrn	NM_011721	Werner syndrome RecQ like helicase	-2.628	0.043437
Cyp2ab1	NM_183158	cytochrome P450, family 2, subfamily ab, polypeptide 1	-2.627	0.022410
Neurl1b	NM_001081656	neuralized E3 ubiquitin protein ligase 1B	-2.622	0.042659
Clps	NM_025469	colipase, pancreatic	-2.617	0.042925
Rnd3	NM_028810	Rho family GTPase 3	-2.601	0.045511
Stfa1	NM_001082543	stefin A1	-2.597	0.023393
Pglyrp1	NM_009402	peptidoglycan recognition protein 1	-2.597	0.043945
Ceacam2	NM_001113368	carcinoembryonic antigen-related cell adhesion molecule 2	-2.589	0.044355
Cobll1	NM_001363076	Cobl-like 1	-2.587	0.044465
Tfr2	NM_001359206	transferrin receptor 2	-2.578	0.024072
D17H6S56E-5	NM_033075	DNA segment, Chr 17, human D6S56E 5	-2.574	0.045164
Cxcl12	NM_001012477	chemokine ligand 12	-2.573	0.024260
Cd24a	NM_009846	CD24a antigen	-2.566	0.047434
Klf1	NM_010635	Kruppel-like factor 1	-2.565	0.024514
Rnase2a	NM_053113	ribonuclease, RNase A family, 2A	-2.561	0.047761
Try4	NM_011646	trypsin 4	-2.549	0.046550
S100a4	NM_011311	S100 calcium binding protein A4	-2.548	0.047837
Ear12	NM_001012766	eosinophil-associated, ribonuclease A family, member 12	-2.547	0.048517
Sorl1	NM_011436	sortilin-related receptor, LDLR class A repeats-containing	-2.537	0.049141
Ccna2	NM_009828	cyclin A2	-2.522	0.048073
Spink2	NM_183284	serine peptidase inhibitor, Kazal type 2	-2.517	0.048386
Slco2b1	NM_175316	solute carrier organic anion transporter family, member 2b1	-2.502	0.026910
Zg16	NM_026918	zymogen granule protein 16	-2.495	0.049698
Mns1	NM_008613	meiosis-specific nuclear structural protein 1	-2.494	0.049766
Zfyve28	NM_001015039	zinc finger, FYVE domain containing 28	-2.493	0.049785
Itgad	NM_001029872	integrin, alpha D	-2.490	0.027378
Reps2	NM_178256	RALBP1 associated Eps domain containing protein 2	-2.486	0.027550
Ndn	NM_010882	necdin	-2.479	0.027852
St6galnac2	NM_009180	ST6 -N-acetylgalactosaminide alpha-2,6-sialyltransferase 2	-2.459	0.028670
Aanat	NM_009591	arylalkylamine N-acetyltransferase	-2.459	0.028684
Il5ra	NM_008370	interleukin 5 receptor, alpha	-2.449	0.029092
9830107B12Rik	NM_001177896	RIKEN cDNA 9830107B12 gene	-2.448	0.029128
Pik3c2b	NM_001099276	phosphatidylinositol-4-phosphate 3-kinase catalytic subunit type 2 beta	-2.445	0.029275
Magi1	NM_001286785	membrane associated guanylate kinase, WW and PDZ domain containing 1	-2.442	0.029386
Slc29a4	NM_146257	solute carrier family 29 , member 4	-2.438	0.029573
Lhx6	NM_001355125	LIM homeobox protein 6	-2.435	0.047203
Ms4a3	NM_133246	membrane-spanning 4-domains, subfamily A, member 3	-2.423	0.030245
Arsi	NM_001038499	arylsulfatase i	-2.413	0.030688
Il9r	NM_001134458	interleukin 9 receptor	-2.407	0.030935
Plcb1	NM_019677	phospholipase C, beta 1	-2.383	0.032045
Ibsp	NM_008318	integrin binding sialoprotein	-2.380	0.032185
Ncam1	NM_001113204	neural cell adhesion molecule 1	-2.354	0.033443
Vwa5b2	NM_001144953	von Willebrand factor A domain containing 5B2	-2.353	0.033509
Ell3	NM_145973	elongation factor RNA polymerase II-like 3	-2.307	0.035866
Fhit	NM_001308285	fragile histidine triad gene	-2.298	0.036319
Ms4a5	NM_183190	membrane-spanning 4-domains, subfamily A, member 5	-2.279	0.037376
Dmbt1	NM_001347632	deleted in malignant brain tumors 1	-2.263	0.038227
Ccn2	NM_010217	cellular communication network factor 2	-2.261	0.038377
Abca6	NM_001166557	ATP-binding cassette, sub-family A , member 6	-2.255	0.038682
Cpm	NM_027468	carboxypeptidase M	-2.253	0.038820
Prss29	NM_053260	protease, serine 29	-2.245	0.039282
Jph3	NM_020605	junctophilin 3	-2.241	0.039499
Gjb2	NM_008125	gap junction protein, beta 2	-2.218	0.040841
Ryr1	NM_009109	ryanodine receptor 1, skeletal muscle	-2.216	0.040990
Cyp27a1	NM_024264	cytochrome P450, family 27, subfamily a, polypeptide 1	-2.208	0.041464
Lbp	NM_008489	lipopolysaccharide binding protein	-2.207	0.041546
Cd22	NM_009845	CD22 antigen	-2.206	0.041595
Depdc1b	NM_178683	DEP domain containing 1B	-2.203	0.041749
Muc13	NM_010739	mucin 13, epithelial transmembrane	-2.194	0.042322
Cldn13	NM_020504	claudin 13	-2.186	0.042837
Pcp4	NM_008791	Purkinje cell protein 4	-2.175	0.043513

Lifr	NM_001358593	LIF receptor alpha	-2.174	0.043628
Isoc2b	NM_026158	isochorismatase domain containing 2b	-2.169	0.043903
Fcrl1	NM_178165	Fc receptor-like 1	-2.164	0.044266
Gprc5c	NM_001110337	G protein-coupled receptor, family C, group 5, member C	-2.159	0.044562
Myh10	NM_175260	myosin, heavy polypeptide 10, non-muscle	-2.149	0.045218
Sema5a	NM_009154	sema domain, seven thrombospondin repeats , transmembrane domain and short cytoplasmic domain, 5A	-2.144	0.045583
Dmtn	NM_013514	dematin actin binding protein	-2.130	0.046487
Gpr156	NM_153394	G protein-coupled receptor 156	-2.128	0.046630
Rhd	NM_011270	Rh blood group, D antigen	-2.124	0.046893
Rapgef4	NM_001355478	Rap guanine nucleotide exchange factor 4	-2.118	0.047311
Fhl1	NM_001077361	four and a half LIM domains 1	-2.101	0.048510
Stbd1	NM_175096	starch binding domain 1	-2.100	0.048609
Amy2a5	NM_001042711	amylase 2a5	-2.100	0.048643
Gsg1	NM_010352	germ cell associated 1	-2.088	0.049510
Crip3	NM_053250	cysteine-rich protein 3	-2.086	0.049598
Nfasc	NM_182716	neurofascin	-2.082	0.049934
Znhit6	NM_001346521	zinc finger, HIT type 6	2.006	20.381
Luc7l	NM_025881	Luc7-like	2.007	20.402
Cd226	NM_178687	CD226 antigen	2.009	20.478
Mgarp	NM_026358	mitochondria localized glutamic acid rich protein	2.012	20.561
1700013F07Rik	NM_029314	RIKEN cDNA 1700013F07 gene	2.019	33.369
Clec5a	NM_021364	C-type lectin domain family 5, member a	2.020	20.804
Flot2	NM_001362626	flotillin 2	2.026	20.997
Plekhm1	NM_183034	pleckstrin homology domain containing, family M member 1	2.029	33.733
Osbpl1a	NM_207530	oxysterol binding protein-like 1A	2.035	33.976
Zglp1	NM_001103168	zinc finger, GATA-like protein 1	2.037	34.043
Oas1d	NM_133893	2-5 oligoadenylate synthetase 1D	2.038	21.361
Hgsnat	NM_029884	heparan-alpha-glucosaminide N-acetyltransferase	2.049	34.521
Camk1g	NM_144817	calcium/calmodulin-dependent protein kinase I gamma	2.049	34.530
Ppp1r7	NM_023200	protein phosphatase 1, regulatory subunit 7	2.053	21.831
Parp11	NM_181402	poly polymerase family, member 11	2.056	21.957
Plekhs1	NM_172641	pleckstrin homology domain containing, family S member 1	2.058	22.003
Ccdc169	NM_001290138	coiled-coil domain containing 169	2.060	34.957
Nkiras1	NM_023526	NFKB inhibitor interacting Ras-like protein 1	2.064	22.204
Ppid	NM_001356326	peptidylprolyl isomerase D	2.065	22.224
Snx6	NM_026998	sorting nexin 6	2.070	22.384
Manea	NM_172865	mannosidase, endo-alpha	2.071	35.401
Fmn1	NM_010230	formin 1	2.081	22.778
Crem	NM_013498	cAMP responsive element modulator	2.083	22.828
Cxcl5	NM_009141	chemokine ligand 5	2.085	22.910
Mrps9	NM_023514	mitochondrial ribosomal protein S9	2.088	22.983
Osbpl3	NM_001347213	oxysterol binding protein-like 3	2.091	23.110
Usp48	NM_001355588	ubiquitin specific peptidase 48	2.094	23.195
Casp3	NM_001284409	caspase 3	2.097	23.315
Tnfsf15	NM_177371	tumor necrosis factor superfamily, member 15	2.102	23.468
Thrap3	NM_001356455	thyroid hormone receptor associated protein 3	2.104	36.728
Gpr26	NM_173410	G protein-coupled receptor 26	2.104	23.532
Il23r	NM_144548	interleukin 23 receptor	2.106	23.615
F3	NM_010171	coagulation factor III	2.106	36.840
Zfp967	NM_001177406	zinc finger protein 967	2.109	23.708
Usp2	NM_198092	ubiquitin specific peptidase 2	2.117	24.010
Pla2g4c	NM_001168504	phospholipase A2, group IVC	2.121	37.443
Pgm1	NM_025700	phosphoglucomutase 1	2.121	24.131
G6pd2	NM_019468	glucose-6-phosphate dehydrogenase 2	2.121	24.136
Pogk	NM_175170	pogo transposable element with KRAB domain	2.123	24.218
Reep3	NM_178606	receptor accessory protein 3	2.124	24.266
Lamtor3	NM_019920	late endosomal/lysosomal adaptor, MAPK and MTOR activator 3	2.125	37.614
Cfap100	NM_173775	cilia and flagella associated protein 100	2.128	24.394
Sod3	NM_011435	superoxide dismutase 3, extracellular	2.130	24.462
Rusc2	NM_001369198	RUN and SH3 domain containing 2	2.130	37.851
Pdk1	NM_172665	pyruvate dehydrogenase kinase, isoenzyme 1	2.130	24.482
Tpgs2	NM_001142698	tubulin polyglutamylase complex subunit 2	2.131	37.877
Ttc39b	NM_027238	tetratricopeptide repeat domain 39B	2.131	24.513
Catsperd	NM_175350	cation channel sperm associated auxiliary subunit delta	2.133	24.579
Susd2	NM_027890	sushi domain containing 2	2.135	24.639
Senp2	NM_029457	SUMO/sentrin specific peptidase 2	2.138	24.759
Vps50	NM_001167751	VPS50 EARP/GARPII complex subunit	2.141	38.302
Folr2	NM_008035	folate receptor 2	2.142	24.906
Slc38a8	NM_001009950	solute carrier family 38, member 8	2.144	38.467
Nmd3	NM_133787	NMD3 ribosome export adaptor	2.148	25.118
Zfp970	NM_001177568	zinc finger protein 970	2.148	38.643
Smcr8	NM_001085440	Smith-Magenis syndrome chromosome region, candidate 8 homolog	2.149	38.665
Ddx58	NM_172689	DEAD box polypeptide 58	2.153	25.293
Rbm26	NM_134077	RNA binding motif protein 26	2.155	38.942
Klra5	NM_008463	killer cell lectin-like receptor, subfamily A, member 5	2.156	38.981

Krt16	NM_001313958	keratin 16	2.162	39.237
Npat	NM_001081152	nuclear protein in the AT region	2.169	39.557
Tmem62	NM_175285	transmembrane protein 62	2.170	25.944
Olfr1414	NM_147039	olfactory receptor 1414	2.171	25.995
Abcf1	NM_013854	ATP-binding cassette, sub-family F , member 1	2.177	26.206
Tmem189	NM_145538	transmembrane protein 189	2.177	26.216
Abca17	NM_001031621	ATP-binding cassette, sub-family A , member 17	2.181	26.363
Cdyl	NM_009881	chromodomain protein, Y chromosome-like	2.188	26.642
Tnfrsf9	NM_011612	tumor necrosis factor receptor superfamily, member 9	2.193	40.647
Pax6	NM_013627	paired box 6	2.194	26.880
Flrt2	NM_201518	fibronectin leucine rich transmembrane protein 2	2.196	26.965
Tcerg1	NM_001039474	transcription elongation regulator 1	2.198	27.046
Cbx5	NM_001110216	chromobox 5	2.201	41.029
Il22	NM_016971	interleukin 22	2.204	27.262
Rhox3f	NM_001040089	reproductive homeobox 3F	2.206	41.248
Dpys	NM_022722	dihydropyrimidinase	2.211	27.551
Tmem38b	NM_028053	transmembrane protein 38B	2.224	42.092
Sncb	NM_001362407	synuclein, beta	2.241	28.799
Zgrf1	NM_197997	zinc finger, GRF-type containing 1	2.243	28.901
Slc41a1	NM_173865	solute carrier family 41, member 1	2.245	28.989
Bcl2l1	NM_009743	BCL2-like 1	2.246	43.186
C2cd3	NM_001017985	C2 calcium-dependent domain containing 3	2.251	43.392
Csl	NM_027945	citrate synthase like	2.253	29.313
Spsb4	NM_145134	splA/ryanodine receptor domain and SOCS box containing 4	2.255	43.604
Marf1	NM_001081154	meiosis regulator and mRNA stability 1	2.259	43.782
Nrg4	NM_032002	neuregulin 4	2.261	29.657
Scn1b	NM_011322	sodium channel, voltage-gated, type I, beta	2.263	44.017
Srrd	NM_027323	SRR1 domain containing	2.270	44.330
Dnmt3l	NM_019448	DNA -methyltransferase 3-like	2.274	30.249
Prpf38a	NM_172697	PRP38 pre-mRNA processing factor 38 domain containing A	2.277	30.390
Gm5136	NM_203660	predicted gene 5136	2.280	44.858
Klra12	NM_010646	killer cell lectin-like receptor subfamily A, member 12	2.282	30.608
Scrt1	NM_130893	scratch family zinc finger 1	2.296	45.696
Vmn1r70	NM_134228	vomeronasal 1 receptor 70	2.298	31.330
Me1	NM_001198933	malic enzyme 1, NADP-dependent, cytosolic	2.335	33.087
Cbll1	NM_001253848	Casitas B-lineage lymphoma-like 1	2.335	47.766
Slfn5	NM_183201	schlafen 5	2.336	33.139
Cyp11a1	NM_019779	cytochrome P450, family 11, subfamily a, polypeptide 1	2.339	33.267
Slc25a43	NM_001085497	solute carrier family 25, member 43	2.346	48.363
Adhfe1	NM_001357377	alcohol dehydrogenase, iron containing, 1	2.360	34.291
Vmn1r46	NM_053229	vomeronasal 1 receptor 46	2.367	34.685
Rbm7	NM_001326356	RNA binding motif protein 7	2.369	34.773
Entpd4	NM_026174	ectonucleoside triphosphate diphosphohydrolase 4	2.369	34.780
Pgm5	NM_175013	phosphoglucomutase 5	2.369	34.782
Cdkn2b	NM_007670	cyclin dependent kinase inhibitor 2B	2.372	34.927
Fam178b	NM_001126046	family with sequence similarity 178, member B	2.387	35.707
Fopnl	NM_025345	Fgfr1op N-terminal like	2.398	36.295
Ifi208	NM_001162938	interferon activated gene 208	2.399	36.344
Tuft1	NM_011656	tuftelin 1	2.416	37.250
Slc6a19	NM_028878	solute carrier family 6 , member 19	2.423	37.616
Ddx4	NM_001145885	DEAD box polypeptide 4	2.453	39.340
Dst	NM_133833	dystonin	2.464	39.988
Tmem199	NM_199199	transmembrane protein 199	2.465	40.045
Skil	NM_001039090	SKI-like	2.471	40.425
Gnb4	NM_013531	guanine nucleotide binding protein , beta 4	2.472	40.478
Gvin1	NM_029000	GTPase, very large interferon inducible 1	2.484	20.353
H2-L	NM_001267808	histocompatibility 2, D region locus L	2.488	20.462
Igsf9b	NM_001326702	immunoglobulin superfamily, member 9B	2.499	42.104
Diablo	NM_023232	diablo, IAP-binding mitochondrial protein	2.504	20.016
Zfp943	NM_001025373	zinc finger prtoein 943	2.508	42.687
Thoc2	NM_001033422	THO complex 2	2.511	42.814
Rheb	NM_053075	Ras homolog enriched in brain	2.512	42.905
Serpine1	NM_008871	serine peptidase inhibitor, clade E, member 1	2.516	43.185
H2-M11	NM_177635	histocompatibility 2, M region locus 11	2.519	43.342
Akap9	NM_194462	A kinase anchor protein 9	2.525	20.530
Lyz1	NM_013590	lysozyme 1	2.530	21.516
Olfr1410	NM_146491	olfactory receptor 1410	2.530	44.078
Sdc3	NM_011520	syndecan 3	2.535	20.495
Mfsd2b	NM_001033488	major facilitator superfamily domain containing 2B	2.539	20.473
1700065D16Rik	NM_001271569	RIKEN cDNA 1700065D16 gene	2.544	44.948
Igsf9	NM_033608	immunoglobulin superfamily, member 9	2.546	20.761
Wfdc13	NM_001012704	WAP four-disulfide core domain 13	2.546	45.091
Abcc5	NM_176839	ATP-binding cassette, sub-family C , member 5	2.551	45.474
Slc35f5	NM_028787	solute carrier family 35, member F5	2.552	45.503
Adam8	NM_007403	a disintegrin and metallopeptidase domain 8	2.555	21.278
Slc23a2	NM_018824	solute carrier family 23 , member 2	2.559	21.380
Usf3	NM_001029889	upstream transcription factor family member 3	2.560	46.024
Dot1l	NM_199322	DOT1-like, histone H3 methyltransferase	2.562	21.461
Zbtb12	NM_198886	zinc finger and BTB domain containing 12	2.567	21.580
Glmn	NM_133248	glomulin, FKBP associated protein	2.575	47.089
Fkbp15	NM_001045528	FK506 binding protein 15	2.578	21.881
Oas3	NM_145226	2-5 oligoadenylate synthetase 3	2.581	21.650
Vxn	NM_178399	vexin	2.583	21.995

Clec5a	NM_001038604	C-type lectin domain family 5, member a	2.587	21.817
Gzmc	NM_010371	granzyme C	2.588	22.136
Oas1b	NM_001083925	2-5 oligoadenylate synthetase 1B	2.588	47.982
Gdpd3	NM_024228	glycerophosphodiester phosphodiesterase domain containing 3	2.593	21.967
Syncrip	NM_019666	synaptotagmin binding, cytoplasmic RNA interacting protein	2.593	22.281
Ptges	NM_022415	prostaglandin E synthase	2.596	22.359
Wrn	NM_011721	Werner syndrome RecQ like helicase	2.598	48.680
Ddx60	NM_001293783	DEAD box polypeptide 60	2.598	22.403
Ptger2	NM_008964	prostaglandin E receptor 2	2.598	48.724
Usp2	NM_198092	ubiquitin specific peptidase 2	2.599	48.755
Gk	NM_212444	glycerol kinase	2.603	22.547
Erich5	NM_173421	glutamate rich 5	2.606	49.302
Rnf24	NM_178607	ring finger protein 24	2.607	22.636
Adora2a	NM_009630	adenosine A2a receptor	2.607	22.641
Tcf7l2	NM_001142923	transcription factor 7 like 2, T cell specific, HMG box	2.609	49.520
Oprl1	NM_001318925	opioid receptor-like 1	2.610	49.557
Myo18b	NM_028901	myosin XVIIIb	2.622	50.429
Krtap4-1	NM_001048196	keratin associated protein 4-1	2.625	50.659
Ralgps1	NM_175211	Ral GEF with PH domain and SH3 binding motif 1	2.628	22.888
Itpripl2	NM_001033380	inositol 1,4,5-triphosphate receptor interacting protein-like 2	2.628	22.903
Rai14	NM_001356534	retinoic acid induced 14	2.629	50.983
Dab2	NM_001037905	disabled 2, mitogen-responsive phosphoprotein	2.633	51.250
Siglec1	NM_011426	sialic acid binding Ig-like lectin 1, sialoadhesin	2.633	23.048
Tnks1bp1	NM_001081260	tankyrase 1 binding protein 1	2.639	23.544
Zmynd15	NM_001029929	zinc finger, MYND-type containing 15	2.641	23.252
Gbp9	NM_172777	guanylate-binding protein 9	2.648	23.781
Dclre1c	NM_175683	DNA cross-link repair 1C	2.649	52.459
Optn	NM_001356487	optineurin	2.649	52.484
Exosc9	NM_019393	exosome component 9	2.650	23.505
Dennd1c	NM_153551	DENN/MADD domain containing 1C	2.654	23.617
Zswim9	NM_177312	zinc finger SWIM-type containing 9	2.654	52.879
Dcun1d5	NM_029775	DCN1, defective in cullin neddylation 1, domain containing 5	2.655	52.989
Armcx3	NM_001358520	armadillo repeat containing, X-linked 3	2.656	53.048
Il15ra	NM_133836	interleukin 15 receptor, alpha chain	2.658	23.738
Il6	NM_031168	interleukin 6	2.662	23.851
Atg2b	NM_029654	autophagy related 2B	2.664	53.634
Ccdc71l	NM_001162903	coiled-coil domain containing 71 like	2.665	24.272
Socs1	NM_009896	suppressor of cytokine signaling 1	2.665	21.837
Lrp12	NM_172814	low density lipoprotein-related protein 12	2.673	24.513
Psg29	NM_054064	pregnancy-specific glycoprotein 29	2.674	54.416
Cd86	NM_019388	CD86 antigen	2.678	24.658
Bmp8a	NM_001256019	bone morphogenetic protein 8a	2.678	24.659
Psme3	NM_011192	proteaseome activator subunit 3	2.678	24.669
D430042O09Rik	NM_001081022	RIKEN cDNA D430042O09 gene	2.690	55.769
Plpp5	NM_001293703	phospholipid phosphatase 5	2.691	25.039
LOC547349	NM_001025208	MHC class I family member	2.697	22.653
Exoc3l4	NM_028807	exocyst complex component 3-like 4	2.702	25.015
Hcar2	NM_030701	hydroxycarboxylic acid receptor 2	2.705	25.107
Mcam	NM_023061	melanoma cell adhesion molecule	2.706	22.902
Tnfrsf9	NM_011612	tumor necrosis factor receptor superfamily, member 9	2.707	25.523
AA467197	NM_001004174	expressed sequence AA467197	2.709	25.204
Cptp	NM_024472	ceramide-1-phosphate transfer protein	2.728	58.966
Pdcl3	NM_026850	phosducin-like 3	2.728	26.193
Usp18	NM_011909	ubiquitin specific peptidase 18	2.743	26.254
Lrrk1	NM_146191	leucine-rich repeat kinase 1	2.746	26.753
Ephb3	NM_010143	Eph receptor B3	2.755	24.250
Fmr1	NM_001290424	fragile X mental retardation 1	2.756	61.448
Cttnbp2nl	NM_030249	CTTNBP2 N-terminal like	2.763	27.307
Gbp7	NM_145545	guanylate binding protein 7	2.765	26.957
Cxcl13	NM_018866	chemokine ligand 13	2.768	62.556
Ep400	NM_029337	E1A binding protein p400	2.773	27.628
Luzp1	NM_024452	leucine zipper protein 1	2.777	27.776
Tuba3a	NM_009446	tubulin, alpha 3A	2.779	27.417
Ifi44	NM_133871	interferon-induced protein 44	2.780	27.423
Lhx9	NM_001025565	LIM homeobox protein 9	2.786	64.212
Parp14	NM_001039530	poly polymerase family, member 14	2.787	27.664
Zfp791	NM_001037745	zinc finger protein 791	2.789	27.745
Gnai2	NM_008138	guanine nucleotide binding protein , alpha inhibiting 2	2.792	25.294
Cd180	NM_008533	CD180 antigen	2.796	65.115
Syn2	NM_013681	synapsin II	2.797	65.281
Oas1a	NM_145211	2-5 oligoadenylate synthetase 1A	2.798	29.689
H2-K1	NM_001347346	histocompatibility 2, K1, K region	2.800	29.766
Gfra4	NM_001271002	glial cell line derived neurotrophic factor family receptor alpha 4	2.808	28.374
Gm10142	NM_001205035	predicted gene 10142	2.821	67.565
Gm11127	NM_001199967	predicted gene 11127	2.823	30.592
Il18bp	NM_010531	interleukin 18 binding protein	2.824	28.903
Plpp1	NM_008903	phospholipid phosphatase 1	2.824	67.902
Fam219a	NM_001159583	family with sequence similarity 219, member A	2.825	26.283
Ifit3	NM_010501	interferon-induced protein with tetratricopeptide repeats 3	2.825	26.289
Inhba	NM_008380	inhibin beta-A	2.831	68.589
Plaat3	NM_139269	phospholipase A and acyltransferase 3	2.832	29.204
Nkap	NM_025937	NFKB activating protein	2.835	68.973
Sntb2	NM_009229	syntrophin, basic 2	2.835	29.792

Vegfa	NM_001025257	vascular endothelial growth factor A	2.839	69.440
Marcksl1	NM_010807	MARCKS-like 1	2.839	69.455
S1pr2	NM_010333	sphingosine-1-phosphate receptor 2	2.840	69.464
Pex14	NM_019781	peroxisomal biogenesis factor 14	2.846	70.082
P2ry13	NM_028808	purinergic receptor P2Y, G-protein coupled 13	2.849	30.285
Snph	NM_198214	syntaphilin	2.851	70.621
Tnfsf10	NM_009425	tumor necrosis factor superfamily, member 10	2.857	71.226
Slc7a11	NM_011990	solute carrier family 7 , member 11	2.860	71.579
Gbp11	NM_001039647	guanylate binding protein 11	2.865	30.349
Slc11a2	NM_008732	solute carrier family 11 , member 2	2.873	31.165
Ralgds	NM_009058	ral guanine nucleotide dissociation stimulator	2.881	73.879
Hspe1	NM_008303	heat shock protein 1	2.887	31.690
Ogfod1	NM_177767	2-oxoglutarate and iron-dependent oxygenase domain containing 1	2.889	28.325
Mpzl2	NM_007962	myelin protein zero-like 2	2.902	76.177
Tmem239	NM_025753	transmembrane 239	2.903	28.794
Gm4951	NM_001033767	predicted gene 4951	2.913	32.140
Pmp22	NM_001302260	peripheral myelin protein 22	2.916	32.798
Map2	NM_001039934	microtubule-associated protein 2	2.921	78.332
Car13	NM_024495	carbonic anhydrase 13	2.929	33.324
Oas1g	NM_011852	2-5 oligoadenylate synthetase 1G	2.931	33.421
Btn1a1	NM_013483	butyrophilin, subfamily 1, member A1	2.932	29.763
Scimp	NM_001045526	SLP adaptor and CSK interacting membrane protein	2.941	80.610
Zfp950	NM_001001737	zinc finger protein 950	2.949	35.612
Zmynd15	NM_001029929	zinc finger, MYND-type containing 15	2.960	82.876
Slc6a8	NM_133987	solute carrier family 6 , member 8	2.965	83.492
Lair1	NM_178611	leukocyte-associated Ig-like receptor 1	2.987	35.746
Gm45935	NM_001320725	predicted gene, 45935	2.991	35.252
Serpina3i	NM_001199940	serine peptidase inhibitor, clade A, member 3I	2.998	36.206
Lhfpl1	NM_178358	lipoma HMGIC fusion partner-like 1	3.006	88.751
Psma3	NM_001310595	proteasome subunit, alpha type 3	3.023	36.635
Aoah	NM_001281854	acyloxyacyl hydrolase	3.027	91.446
Nagk	NM_019542	N-acetylglucosamine kinase	3.029	37.588
Fut1	NM_008051	fucosyltransferase 1	3.035	92.577
Cndp2	NM_023149	CNDP dipeptidase 2	3.040	37.384
Oasl1	NM_145209	2-5 oligoadenylate synthetase-like 1	3.041	33.797
Inhba	NM_008380	inhibin beta-A	3.042	37.453
Isg15	NM_015783	ISG15 ubiquitin-like modifier	3.053	40.335
Cxcr6	NM_030712	chemokine receptor 6	3.054	38.016
Gtdc1	NM_001362986	glycosyltransferase-like domain containing 1	3.058	95.718
Adgre1	NM_010130	adhesion G protein-coupled receptor E1	3.059	95.881
Smpdl3b	NM_133888	sphingomyelin phosphodiesterase, acid-like 3B	3.059	38.950
Vps54	NM_139061	VPS54 GARP complex subunit	3.089	100.244
Vax2	NM_011912	ventral anterior homeobox 2	3.094	40.642
Edn1	NM_010104	endothelin 1	3.096	101.267
Blvrb	NM_144923	biliverdin reductase B)	3.107	36.487
Capza2	NM_007604	capping protein muscle Z-line, alpha 2	3.108	103.096
Ppp6r1	NM_172894	protein phosphatase 6, regulatory subunit 1	3.126	42.204
Ahrr	NM_009644	aryl-hydrocarbon receptor repressor	3.128	106.167
Ak4	NM_001177602	adenylate kinase 4	3.133	42.551
Htr7	NM_001347442	5-hydroxytryptamine receptor 7	3.140	42.952
Mmp14	NM_008608	matrix metallopeptidase 14	3.158	43.859
Srgap2	NM_001081011	SLIT-ROBO Rho GTPase activating protein 2	3.160	43.105
Slc15a3	NM_023044	solute carrier family 15, member 3	3.160	38.784
Ccl2	NM_011333	chemokine ligand 2	3.163	46.085
Tma16	NM_001357208	translation machinery associated 16	3.170	39.229
Mertk	NM_008587	MER proto-oncogene tyrosine kinase	3.171	43.667
Zc3h12c	NM_001162921	zinc finger CCCH type containing 12C	3.184	45.289
Cxcl10	NM_021274	chemokine ligand 10	3.188	47.478
Zfp281	NM_001160251	zinc finger protein 281	3.199	46.077
Ccl8	NM_021443	chemokine ligand 8	3.216	120.767
Batf2	NM_028967	basic leucine zipper transcription factor, ATF-like 2	3.219	41.549
Best1	NM_011913	bestrophin 1	3.248	126.652
Ttc39b	NM_027238	tetratricopeptide repeat domain 39B	3.249	126.765
Il6	NM_031168	interleukin 6	3.258	49.444
Gm12250	NM_001135115	predicted gene 12250	3.273	131.380
Tlr4	NM_021297	toll-like receptor 4	3.282	133.059
Clec4e	NM_019948	C-type lectin domain family 4, member e	3.282	44.680
Ch25h	NM_009890	cholesterol 25-hydroxylase	3.302	51.040
Radx	NM_175326	RPA1 related single stranded DNA binding protein, X-linked	3.312	139.158
Rpl13	NM_016738	ribosomal protein L13	3.319	52.106
Gm8909	NM_001081032	predicted gene 8909	3.321	52.209
Serpinb2	NM_011111	serine peptidase inhibitor, clade B, member 2	3.343	145.714
Cd1d1	NM_007639	CD1d1 antigen	3.347	48.179
Ifi208	NM_001162938	interferon activated gene 208	3.355	148.278
Cybb	NM_007807	cytochrome b-245, beta polypeptide	3.359	54.627
Ctla2b	NM_007797	cytotoxic T lymphocyte-associated protein 2 beta	3.359	54.641
Iigp1	NM_021792	interferon inducible GTPase 1	3.362	49.035
Rsad2	NM_021384	radical S-adenosyl methionine domain containing 2	3.372	49.582
Spaca4	NM_027055	sperm acrosome associated 4	3.373	152.234
Tnf	NM_013693	tumor necrosis factor	3.373	55.575
Il10	NM_010548	interleukin 10	3.384	154.597
Mitd1	NM_001356262	MIT, microtubule interacting and transport, domain containing 1	3.391	58.015
Adgb	NM_001127353	androglobin	3.430	60.794

Cers6	NM_172856	ceramide synthase 6	3.432	165.994
Slc7a11	NM_011990	solute carrier family 7 , member 11	3.433	166.294
Oxsm	NM_027695	3-oxoacyl-ACP synthase, mitochondrial	3.436	64.001
Ccl3	NM_011337	chemokine ligand 3	3.438	53.576
Me1	NM_001198933	malic enzyme 1, NADP-dependent, cytosolic	3.451	170.651
Rnf208	NM_176834	ring finger protein 208	3.461	173.330
Il18bp	NM_010531	interleukin 18 binding protein	3.471	62.456
Nrp2	NM_001077403	neuropilin 2	3.487	56.662
Bcl2l2	NM_007537	BCL2-like 2	3.506	185.009
C1ra	NM_023143	complement component 1, r subcomponent A	3.511	67.017
Cxcl1	NM_008176	chemokine ligand 1	3.512	67.118
P2ry14	NM_133200	purinergic receptor P2Y, G-protein coupled, 14	3.517	67.475
Calhm6	NM_175449	calcium homeostasis modulator family member 6	3.521	66.272
Acsl1	NM_007981	acyl-CoA synthetase long-chain family member 1	3.528	68.403
Gp6	NM_001163014	glycoprotein 6	3.549	197.068
Il1a	NM_010554	interleukin 1 alpha	3.549	197.276
Ggh	NM_010281	gamma-glutamyl hydrolase	3.554	70.558
Mx1	NM_010846	MX dynamin-like GTPase 1	3.558	70.881
Gm4841	NM_001034859	predicted gene 4841	3.565	201.773
Prm1	NM_013637	protamine 1	3.567	71.655
Cd274	NM_021893	CD274 antigen	3.575	62.755
Tnip3	NM_001001495	TNFAIP3 interacting protein 3	3.578	205.716
Calca	NM_007587	calcitonin/calcitonin-related polypeptide, alpha	3.587	208.464
Cfb	NM_001142706	complement factor B	3.598	64.457
1700001K19Rik	NM_025488	RIKEN cDNA 1700001K19 gene	3.641	225.816
Clec4n	NM_020001	C-type lectin domain family 4, member n	3.642	67.840
Agtrap	NM_009642	angiotensin II, type I receptor-associated protein	3.647	78.909
Ccr5	NM_009917	chemokine receptor 5	3.654	79.558
Kazn	NM_001109685	kazrin, periplakin interacting protein	3.659	80.047
Apol9a	NM_173786	apolipoprotein L 9a	3.679	79.978
Slc6a12	NM_133661	solute carrier family 6 , member 12	3.716	252.161
Slfn4	NM_011410	schlafen 4	3.719	83.887
Rsad2	NM_021384	radical S-adenosyl methionine domain containing 2	3.725	86.666
Phb	NM_008831	prohibitin	3.751	87.077
Fam167a	NM_177628	family with sequence similarity 167, member A	3.766	88.712
Stab1	NM_138672	stabilin 1	3.795	91.769
Ccl1	NM_011329	chemokine ligand 1	3.795	283.321
Procr	NM_011171	protein C receptor, endothelial	3.806	92.933
Gbp8	NM_029509	guanylate-binding protein 8	3.818	96.825
Pdpn	NM_010329	podoplanin	3.863	313.130
Ly6i	NM_020498	lymphocyte antigen 6 complex, locus I	3.889	90.359
Upp1	NM_009477	uridine phosphorylase 1	3.892	103.040
F7	NM_010172	coagulation factor VII	3.917	338.846
Scn8a	NM_001077499	sodium channel, voltage-gated, type VIII, alpha	3.934	111.378
Rbpj	NM_001359152	recombination signal binding protein for immunoglobulin kappa J region	3.949	355.015
Ppp1r3b	NM_001364451	protein phosphatase 1, regulatory subunit 3B	3.966	112.451
Gbp2b	NM_010259	guanylate binding protein 2b	3.975	368.805
Tgm2	NM_009373	transglutaminase 2, C polypeptide	3.989	101.504
Apol9b	NM_001168660	apolipoprotein L 9b	3.991	119.274
Gm4951	NM_001033767	predicted gene 4951	4.018	393.044
Oasl1	NM_001359945	2-5 oligoadenylate synthetase-like 1	4.049	108.820
Cxcl3	NM_203320	chemokine ligand 3	4.051	412.433
Slc13a3	NM_054055	solute carrier family 13 , member 3	4.091	130.573
Cfb	NM_008198	complement factor B	4.122	145.935
Ier3	NM_133662	immediate early response 3	4.136	467.679
Slc7a2	NM_001044740	solute carrier family 7 , member 2	4.251	553.669
Oas1f	NM_145153	2-5 oligoadenylate synthetase 1F	4.301	145.785
Serpina3f	NM_001168294	serine peptidase inhibitor, clade A, member 3F	4.373	662.209
Saa3	NM_011315	serum amyloid A 3	4.379	198.845
Basp1	NM_027395	brain abundant, membrane attached signal protein 1	4.560	228.089
Saa1	NM_009117	serum amyloid A 1	4.586	235.185
Acod1	NM_008392	aconitate decarboxylase 1	4.824	267.466
Serpina3f	NM_001168294	serine peptidase inhibitor, clade A, member 3F	4.956	379.581
Ptges	NM_022415	prostaglandin E synthase	4.989	394.740
Nos2	NM_010927	nitric oxide synthase 2, inducible	5.104	435.498
Gpnmb	NM_053110	glycoprotein nmb	5.222	522.466
Ifit1	NM_008331	interferon-induced protein with tetratricopeptide repeats 1	5.412	655.579
Gbp6	NM_194336	guanylate binding protein 6	5.535	760.518
Ly6f	NM_008530	lymphocyte antigen 6 complex, locus F	5.782	976.275
Ccl7	NM_013654	chemokine ligand 7	5.795	990.575
Ccl12	NM_011331	chemokine ligand 12	6.245	1781.630
Gm15056	NM_001177471	predicted gene 15056	6.967	3996.626

Supplementary Table 5. Statistically signifigcant KEGG pathway of the differentally expressed genes between sham and 5/6Nx splenic monocytes

**1. Metabolism**	**%**	**P-Value**
Fatty acid biosynthesis	0.5	0.075
Primary bile acid biosynthesis	0.5	0.095
beta-Alanine metabolism	0.7	0.092

**2. Genetic Information Processing**	**%**	**P-Value**

**3. Environmental Information Processing**	**%**	**P-Value**
Jak-STAT signaling pathway	1.7	0.048
TNF signaling pathway	1.7	0.0093
Cytokine-cytokine receptor interaction	4.9	4.4E-09
Cell adhesion molecules (CAMs)	2.5	0.00083

**4. Cellular Processes**	**%**	**P-Value**
Phagosome	2	0.025

**5. Organismal Systems**	**%**	**P-Value**
Hematopoietic cell lineage	2	0.000094
Complement and coagulation cascades	1.7	0.00079
Intestinal immune network for IgA production	1.2	0.0023
Chemokine signaling pathway	3	0.00023
Pancreatic secretion	1.8	0.0015
Protein digestion and absorption	2.2	0.000026

**6. Human Diseases**	**%**	**P-Value**
Asthma	0.7	0.042
Rheumatoid arthritis	2.2	0.000013
Autoimmune thyroid disease	1	0.083
Inflammatory bowel disease (IBD)	1.5	0.00062
Allograft rejection	1.2	0.0096
Graft-versus-host disease	1.3	0.0014
Viral myocarditis	1.2	0.044
Type I diabetes mellitus	1.2	0.015
Salmonella infection	1.3	0.014
Pertussis	1.7	0.00065
Legionellosis	1	0.038
Staphylococcus aureus infection	1	0.023
Tuberculosis	2.7	0.00063
Measles	2.2	0.0016
Influenza A	3.2	0.000011
Hepatitis C	1.7	0.034
Amoebiasis	2	0.0015
Malaria	2	2.9E-07
Toxoplasmosis	1.8	0.0022
Leishmaniasis	1.3	0.0047
Chagas disease (American trypanosomiasis)	2	0.00052
African trypanosomiasis	1.5	0.000013

**7. Drug Development**	**%**	**P-Value**

Supplementary Table 6. Primer sets for PCR analysis of gene expression

Gene	Primers
**Mouse *Gpr68***	
Forward	5'-CGTGGTCATCTTCCTGGCTT-3'
Reverse	5'-TGGTGAGGAGGAGGGAGAAG-3'
**Mouse *aSMA***	
Forward	5'-GGCACCACTGAACCCTAAGG-3'
Reverse	5'-ACAATACCAGTTGTACGTCCAGA-3'
**Mouse *Col1a2***	
Forward	5'-AAGGGTGCTACTGGACTCCC-3'
Reverse	5'-TTGTTACCGGATTCTCCTTTGG-3'
**Mouse *Tnf-a***	
Forward	5'-CTGAACTTGGGGGTGATCGG-3'
Reverse	5'-GGCTTGTCACTCGAATTTTGAG-3'
**Mouse *IL-6***	
Forward	5'-CTGCAAGAGACTTCCATCCAG-3'
Reverse	5'-AGTGGTATAGACAGGTCTGTTGG-3'
**Mouse *Clock***	
Forward	5'-TTGCTCCACGGGAATCCTT-3'
Reverse	5'-GGAGGGAAAGTGCTCTGTTGTAG-3'
**Mouse *Arntl***	
Forward	5'-GGACTTCGCCTCTACCTGTTCA-3'
Reverse	5'-AACCATGTGCGAGTGCAGGCGC-3'
**Mouse *Per1***	
Forward	5'-CCAGATTGGTGGAGGTTACTGAGT-3'
Reverse	5'-GCGAGAGTCTTCTTGGAGCAGTAG-3'
**Mouse *Nr1d1***	
Forward	5'-CCCTGGACTCCAATAACAACACA-3'
Reverse	5'-GCCATTGGAGCTGTCACTGTAG-3'
**Mouse *Cys1***	
Forward	5'-GAAGAGGAGCTCATGGCGAG-3'
Reverse	5'-ACCGAGACCTTGGTGGAAAC-3'
**Mouse *Ppl***	
Forward	5'-CAAAGGCAAATACAGCCCAAC-3'
Reverse	5'-TTCCACCTGGTCTGCATTCTT-3'
**Mouse *Sele***	
Forward	5'-ATGCCTCGCGCTTTCTCTC-3'
Reverse	5'-GTAGTCCCGCTGACAGTATGC-3'
**Mouse *Vcam1***	
Forward	5'-AGTTGGGGATTCGGTTGTTCT-3'

	Reverse	5'-CCCCTCATTCCTTACCACCC-3'
**Mouse** ***Aldh1a1***		
	Forward	5'-AACACAGGTTGGCAAGTTAATCA-3'
	Reverse	5'-TGCGACACAACATTGGCCTT-3'
**Mouse** ***Aldh1a2***		
	Forward	5'-AGGTGGATATAGACAAGGCAGT-3'
	Reverse	5'-CCGCCATTTAGGGATTCCATAG-3'
**Mouse** ***Aldh1a3***		
	Forward	5'-GAAGAGGCCAGTTGGAGACC-3'
	Reverse	5'-CACATTCTAGCTTGGCCCCT-3'
**Mouse** ***Rbp1***		
	Forward	5'-AACGTGGCCTTACGCAAAATC-3'
	Reverse	5'-GCGGTCGTCTATGCCTGTC-3'
**Mouse** ***18s Ribosome***		
	Forward	5'-AGAAGCCCCTGGCACTCTAT-3'
	Reverse	5'-GCAAAGTGGGCACAGTGATG-3'
**Human** ***GPR68***		
	Forward	5'-TGTACCATCGACCATACCATCC-3'
	Reverse	5'-GGTAGCCGAAGTAGAGGGACA-3'
**Human** ***IL6***		
	Forward	5'-TGCAATAACCACCCCTGACC-3'
	Reverse	5'-GTGCCCATGCTACATTTGCC-3'
**Human** ***CLOCK***		
	Forward	5'-CACAGTCAGCAAACATCTCTACCC-3'
	Reverse	5'-AATCTGGACCATGCTTCCGG-3'
**Human** ***ARNTL***		
	Forward	5'-AAGGGAAGCTCACAGTCAGAT-3'
	Reverse	5'-GGACATTGCGTTGCATGTTGG-3'
**Human** ***TNF***		
	Forward	5'-GAGGCCAAGCCCTGGTATG-3'
	Reverse	5'-CGGGCCGATTGATCTCAGC-3'
**Human** ***18s Ribosome***		
	Forward	5'-AGAAGCCCCTGGCACTCTAT-3'
	Reverse	5'-GCAAAGTGGGCACAGTGATG-3'

Supplementary Table 7. Primer sets for PCR analysis of ChIP analysis

Gene		Primers
**Mouse *Gpr68* gene (Ebox 1)**		
	Forward	5'-AGTAATAAAACTTCATTGCAGTCCC-3'
	Reverse	5'-GTCACTCACCCAATCAATGAAACC-3'
**Mouse *Gpr68* gene (Ebox 2)**		
	Forward	5'-AGGGCCATCTTTAACAACTG-3'
	Reverse	5'-GTCCCTGTTCTTTTGGTTTG-3'
**Mouse *Clock* gene STAT5 binding site**		
	Forward	5'-GGCCTGCGAAGGGAAGGGG-3'
	Reverse	5'-CGGGCGCGCATGCGACTTTC-3'
**Mouse *Arntl* gene STAT5 binding site**		
	Forward	5'-GTCTTCTAAATGCGCTAGCT-3'
	Reverse	5'-GTGACTGCCTCTCAGCTC-3'
**Human *GPR68* gene Ebox I**		
	Forward	5'-AGGGACCCAGGAGGCTG-3'
	Reverse	5'-GGGTGAGAAGCCCAGAGAACA-3'
**Human *ARNTL* gene STAT5 binding site**		
	Forward	5'-TTGCAGTCCTCGGGGTGGAAAT-3'
	Reverse	5'-ACTCCCTCTCTCCCTCCGGCT-3'
**Human *CLOCK* gene STAT5 binding site**		
	Forward	5'-TGCGCATGCGACTTGCCCCG-3'
	Reverse	5'-CCTTTCCCCAGCGCGGCT-3'

Supplementaly Table 8. Concentrations of various components in the serum of human specimens.

	Scr	Total protein	Total chol	HDL chol	LDL chol	BNP	Blood Gluocose	Retinol	RBP4	TNF	IL6
**Subject#**	**mg/dL**	**g/mL**	**mg/dL**	**mg/dL**	**mg/dL**	**pg/mL**	**mg/dL**	**μM**	**μM**	**pg/mL**	**pg/mL**
**Healthy-1**	0.68	6.9	169.0	51.0	85	12.52	104	1.15	1.32	7.04	11.18
**Healthy-2**	0.64	7.3	210.0	44.0	107	17.09	88	1.63	1.31	11.62	12.70
**Healthy-3**	1.11	6.9	242.0	43.0	127	14.27	116	1.69	2.45	1.92	17.69
**Healthy-4**	0.74	7.0	158.0	65.0	58	15.77	125	2.01	1.29	10.46	7.98
**Healthy-5**	0.62	5.7	157.0	51.0	88	27.44	137	1.53	1.36	0.55	1.14
**Healthy-6**	0.01	9.8	89.0	-	-	10.92	116	1.26	1.43	6.60	6.70
**Healthy-7**	0.02	8.0	99.3	-	-	13.54	132	1.22	1.56	9.79	4.36
**Healthy-8**	0.01	7.3	86.9	-	-	13.19	92	1.33	1.65	12.90	7.33
**CKD-1**	1.66	5.7	186.0	110	74.00	140.30	113	4.37	3.21	14.91	26.39
**CKD-2**	1.61	7.3	173.0	241	47.00	16.60	132	2.51	2.03	5.99	10.21
**CKD-3**	1.39	7.2	153.0	89	48.00	31.30	119	2.77	3.10	14.97	5.63
**CKD-4**	2.93	6.8	123.0	104	51.00	407.60	151	4.66	2.15	17.43	3.88
**CKD-5**	1.45	6.7	124.0	103	56.00	84.50	147	1.96	3.42	10.07	45.51
**CKD-6**	5.82	8.0	162.0	117	57.00	60.30	113	2.87	4.09	11.01	7.04
**CKD-7**	4.6	8.3	154.0	117	32.00	680.10	79	2.07	4.54	11.62	67.97
**CKD-8**	1.25	7.2	147.0	88	45.00	67.50	110	2.73	2.91	15.75	12.70
**CKD-9**	1.25	4.0	252.0	231	30.00	639.00	76	4.11	1.28	11.12	11.38
**CKD-10**	1.36	7.5	136.0	52	52.00	58.30	98	2.67	2.34	20.07	27.35
**CKD-11**	1.22	7.7	187.0	130	70.00	28.20	97	3.46	3.92	16.82	5.52
**CKD-12**	1.04	7.3	164.0	-	-	76.56	156	3.91	2.13	7.26	9.86
**CKD-13**	1.06	10.2	126.2	-	-	84.21	130	3.37	1.60	14.97	68.32
**CKD-14**	1.07	9.0	114.9	-	-	81.45	103	3.41	2.48	4.36	13.51
**CKD-15**	9.92	9.3	102.3	-	-	216.64	140	6.65	6.36	9.24	8.44
**CKD-16**	6.79	9.1	106.7	-	-	210.39	184	8.03	3.56	2.68	5.68
**CKD-17**	9.14	10.4	138.6	-	-	226.43	165	6.94	4.21	29.81	9.66
