# Supplementary material for: Alteration of circadian machinery in monocytes underlies chronic kidney disease-associated cardiac inflammation and fibrosis
Source: Nat Commun. 2021 May 13;12:2783. doi: 10.1038/s41467-021-23050-x (PMC8119956; doi:10.1038/s41467-021-23050-x)
Supplement: Supplementary file 3 — Reporting Summary [file 41467_2021_23050_MOESM3_ESM.pdf]

nature research

Corresponding author(s): Shigehiro Ohdo
Last updated by author(s): Mar 8, 2021

Reporting Summary

Nature Research wishes to improve the reproducibility of the work that we publish. This form provides structure for consistency and transparency in reporting. For further information on Nature Research policies, see our Editorial Policies and the Editorial Policy Checklist.

Statistics

For all statistical analyses, confirm that the following items are present in the figure legend, table legend, main text, or Methods section.

n/a	Confirmed	
☐	☒	The exact sample size (*n*) for each experimental group/condition, given as a discrete number and unit of measurement
☐	☒	A statement on whether measurements were taken from distinct samples or whether the same sample was measured repeatedly
☐	☒	The statistical test(s) used AND whether they are one- or two-sided<br>*Only common tests should be described solely by name; describe more complex techniques in the Methods section.*
☐	☒	A description of all covariates tested
☐	☒	A description of any assumptions or corrections, such as tests of normality and adjustment for multiple comparisons
☐	☒	A full description of the statistical parameters including central tendency (e.g. means) or other basic estimates (e.g. regression coefficient) AND variation (e.g. standard deviation) or associated estimates of uncertainty (e.g. confidence intervals)
☐	☒	For null hypothesis testing, the test statistic (e.g. *F*, *t*, *r*) with confidence intervals, effect sizes, degrees of freedom and *P* value noted<br>*Give P values as exact values whenever suitable.*
☒	☐	For Bayesian analysis, information on the choice of priors and Markov chain Monte Carlo settings
☒	☐	For hierarchical and complex designs, identification of the appropriate level for tests and full reporting of outcomes
☒	☐	Estimates of effect sizes (e.g. Cohen's *d*, Pearson's *r*), indicating how they were calculated

Our web collection on statistics for biologists contains articles on many of the points above.

Software and code

Policy information about availability of computer code

Data collection	BD FACSDiva version 8.0.1 for flowcytometry, BZ-810 (KEYENCE) for Immunofluorescence histochemical and MT staining, Nemio GX image analyzing system (SSA-580A, Toshiba Medical Systems), Agilent 2100 Bioanalyzer (Agilent Technologies), AQUITY UPLC H-Class XHCLQT0100 system (Waters).
Data analysis	FlowJo v10 (Flow Jo LLC) , ImageJ ver.1.53e (NIH) , LightCycler® 96 System (Roche), BZ-H3C Analysis Application Hybrid cell count (KEYENCE), AQUITY TQ mass spectrometer controlled by MasslynxTM software (Waters).

For manuscripts utilizing custom algorithms or software that are central to the research but not yet described in published literature, software must be made available to editors and reviewers. We strongly encourage code deposition in a community repository (e.g. GitHub). See the Nature Research guidelines for submitting code & software for further information.

Data

Policy information about availability of data

All manuscripts must include a data availability statement. This statement should provide the following information, where applicable:

- Accession codes, unique identifiers, or web links for publicly available datasets
- A list of figures that have associated raw data
- A description of any restrictions on data availability

All data supporting the results of the present study are included in the article, either in the main figures, supplementary Information Files or Source data. Microarray data were submitted to the Gene Expression Omnibus at the National Center for Biotechnology Information (Cardiac ventricle: GSE150094, Kidney: GSE57799, Liver: GSE35135, Monocytes: GSE140706). Functional analysis of the microarray datasets were performed using Kyoto Encyclopedia of Genes and Genomes (KEGG) (https://www.genome.jp/kegg/kegg_ja.html) database and Gene Ontology Recource (http://geneontology.org/) on the DAVID system (https://david.ncifcrf.gov/home.jsp).

Field-specific reporting

Please select the one below that is the best fit for your research. If you are not sure, read the appropriate sections before making your selection.

☒ Life sciences ☐ Behavioural & social sciences ☐ Ecological, evolutionary & environmental sciences

For a reference copy of the document with all sections, see nature.com/documents/nr-reporting-summary-flat.pdf

Life sciences study design

All studies must disclose on these points even when the disclosure is negative.

Sample size	No statistical method was used to predetermine sample sizes, but our sample sizes are similar to those reported in previous publications (Ohdo et al.,Nat Med. 2001 Mar;7(3):356-60. Koyanagi et al.,Nat Commun. 2016 Oct 14;7:13102. etc).This statement is described in method.
Data exclusions	No data were excluded from the analysis
Replication	Experiments were replicated several times with reproducible results, as indicated in each figure legend.
Randomization	Mice were paired based on gender (male), weight and age, and randomly selected for experiments. The other samples (cultured monocytes) were randomly allocated to control and experimental groups.
Blinding	[For experimental mouse interventions] Investigators were not blinded to group allocation during data collection and analysis as knowledge of the treatment groups was required.<br>[For experiments using human serum] The doctors who collected the serum samples and the investigators are another persons. The experiments are blinded because the investigators performed the experiments and analysis without knowing the clinical data of the patients who donated the serum.

Reporting for specific materials, systems and methods

We require information from authors about some types of materials, experimental systems and methods used in many studies. Here, indicate whether each material, system or method listed is relevant to your study. If you are not sure if a list item applies to your research, read the appropriate section before selecting a response.

Materials & experimental systems

n/a	Involved in the study	
☐	☒	Antibodies
☐	☒	Eukaryotic cell lines
☒	☐	Palaeontology and archaeology
☐	☒	Animals and other organisms
☐	☒	Human research participants
☒	☐	Clinical data
☒	☐	Dual use research of concern

Methods

n/a	Involved in the study	
☒	☐	ChIP-seq
☐	☒	Flow cytometry
☒	☐	MRI-based neuroimaging

Antibodies

Antibodies used	anti-GPR68 (CSB-PA060199; CUSABIO, Lot No: G1421R2), anti-ARNTL (ab-93806; Abcam, Lot No: GR3191558-31), anti-CLOCK (ab-3517; Abcam, Lot No: GR322368-3), anti-phosphorylated STAT5A/B (Y694/699) (9359; Cell Signaling, Clone No: C11C5, Lot No: 0008), anti-STAT5A/B (sc-835; Santa Cruz Biotechnology, Lot No: D0714), anti-STRA6 (NBP1-00242; Novus Biologicals, Lot No: C2), anti-RBP4 (ab-109193; Abcam, Clone No: EP3657, Lot No: GR252174-10), anti-ACTB (SC-1616; Santa Cruz Biotechnology, Lot No: B1815), Cy3 anti-Rabbit IgG (AP132C; Sigma Aldrich), Alexa Fluor® 488 anti-Rat IgG (A11006; Invitrogen, Lot No: 1259366), anti-VCAM1 (ab134047; Abcam, Clone no: EPR5047), anti-TBP (ab-51841; Abcam, Clone No: mAbcam51841), anti-F4/80 (MCA497RT; BioRad, Batch No: 1603), anti-F4/80-APCfireTM (BL-123151; BioLegend, Clone No: BM8, Lot No: B235527), anti-F4/80-FITC (BL-123108; BioLegend, Clone No: BM8, Lot No: B222019), anti-CD11b-PE (BL-101208; BioLegend, Clone No: M1/70, Lot No: B253922), anti-Ly6C-APC (BL-128016; BioLegend, Clone No: HK1.4, Lot No: B234316) anti-Ly6C-FITC (BL-128006; BioLegend, Clone No: HK1.4, Lot No: B247728), anti-TNF-α-APC (BL-506307; BioLegend, Clone No: MP6-KT22, Lot No: B255366), anti-IL-6 (BL-504507; BioLegend, Clone No: MP5-20F3, Lot No: B263271), anti-Ly6G-FITC (BL-127606; BioLegend, Clone No: 1A8, Lot No: B277117), anti-Alexa Fluor® 647 Rabbit IgG (BL-406414; BioLegend, Clone No: Poly4064, Lot No: B268583) anti-FITC Rat IgG2b isotype control (BL-400606; BioLegend, Clone No: RTK4530, Lot No: B265826), anti-PE Mouse IgG2a isotype control (BL-400606; BioLegend, Clone No: MOPC-173, Lot No: B302650), anti-PE Rat IgG2a isotype control (BL-400508; BioLegend, Clone No: RTK2758, Lot No: B290861), anti-APC Rat IgG2a isotype control (BL-400512; BioLegend, Clone No: RTK2758, Lot No: B284967), anti-APCfireTM Rat IgG2a isotype control (BL-400567; BioLegend, Clone No: RTK2758, Lot No: B304219), anti-HRP mouse IgG (ab6820; Abcam, Lot No: GR3186683-12), anti-HRP rabbit IgG (ab97051; Abcam, Lot No: GR3231028-5), anti-HRP guinea pig IgG (ab97055; Abcam, Lot No: GR3175094-10)

For WB, Can Get Signal Immunoreaction Enhancer Solution (Toyobo) was used for dilution of antibodies. For Immunofluorescence histochemical staining, Can Get Signal Immunostain Immunoreaction Enhancer Solution (Toyobo) was used for dilution of antibodies. For ChIP, antibodies were diluted in purified water with 16.7 mM Tris-HCl, 167 mM NaCl, 1.2 mM EDTA, 1.1 % Triton X-100, 10 % SDS. For FCM, antibodies were diluted in PBS with 10 % FBS and 10 mM EDTA.

Validation

All the antibodies used in this study were commercial antibodies. Validation of all antibodies was performed by the respective companies.
anti-GPR68 (CSB-PA060199; CUSABIO, Lot No: G1421R2), https://www.cusabio.com/Polyclonal-Antibody/GPR68-Antibody-11096773.html
anti-ARNTL (ab-93806; Abcam, Lot No: GR3191558-31), https://www.abcam.co.jp/bmal1-antibody-ab93806.html
anti-CLOCK (ab-3517; Abcam, Lot No: GR322368-3), https://www.abcam.co.jp/kat13d--clock-antibody-ab3517.html
anti-phosphorylated STAT5A/B (Y694/699) (9359; Cell Signaling, Clone No: C11C5, Lot No: 0008),
anti-STAT5A/B (sc-835; Santa Cruz Biotechnology, Lot No: D0714), https://www.cellsignal.jp/products/primary-antibodies/phospho-stat5-tyr694-c11c5-rabbit-mab/9359
anti-STRA6 (NBP1-00242; Novus Biologicals, Lot No: C2), https://www.antibodies.com/stra6-antibody-a84350
anti-RBP4 (ab-109193; Abcam, Clone No: EP3657, Lot No: GR252174-10), https://www.abcam.co.jp/rbp4-antibody-ep3657-ab109193.html
anti-ACTB (SC-1616; Santa Cruz Biotechnology, Lot No: B1815), https://www.scbt.com/ja/p/actin-antibody-i-19
Cy3 anti-Rabbit IgG (AP132C; Sigma Aldrich), https://www.merckmillipore.com/JP/ja/product/Goat-Anti-Rabbit-IgG-Antibody-Cy3-conjugate,MM_NF-AP132C
Alexa Fluor® 488 anti-Rat IgG (A11006; Invitrogen, Lot No: 1259366), https://www.thermofisher.com/antibody/product/Goat-anti-Rat-IgG-H-L-Cross-Adsorbed-Secondary-Antibody-Polyclonal/A-11006
anti-VCAM1 (ab134047; Abcam, Clone no: EPR5047), https://www.abcam.co.jp/vcam1-antibody-epr5047-ab134047.html
anti-TBP (ab-51841; Abcam, Clone No: mAbcam51841), https://www.abcam.co.jp/tata-binding-protein-tbp-antibody-mabcam-51841-chip-grade-ab51841.html
anti-F4/80 (MCA497RT; BioRad, Batch No: 1603), https://bio-rad-antibody.jp/antibody/detail/gid:bio-rad/cnum:MCA497RT/
anti-F4/80-APCfireTM (BL-123151; BioLegend, Clone No: BM8, Lot No: B235527), https://www.biolegend.com/ja-jp/products/apc-fire-750-anti-mouse-f4-80-antibody-13197
anti-F4/80-FITC (BL-123108; BioLegend, Clone No: BM8, Lot No: B222019), https://www.biolegend.com/ja-jp/products/fitc-anti-mouse-f4-80-antibody-4067
anti-CD11b-PE (BL-101208; BioLegend, Clone No: M1/70, Lot No: B253922), https://www.biolegend.com/ja-jp/products/pe-anti-mouse-human-cd11b-antibody-349
anti-Ly6C-APC (BL-128016; BioLegend, Clone No: HK1.4, Lot No: B234316), https://www.biolegend.com/ja-jp/products/apc-anti-mouse-ly-6c-antibody-6047
anti-Ly6C-FITC (BL-128006; BioLegend, Clone No: HK1.4, Lot No: B247728), https://www.biolegend.com/ja-jp/products/fitc-anti-mouse-ly-6c-antibody-4896
anti-TNF-α-APC (BL-506307; BioLegend, Clone No: MP6-KT22, Lot No: B255366), https://www.biolegend.com/ja-jp/products/apc-anti-mouse-tnf-alpha-antibody-975
anti-IL-6 (BL-504507; BioLegend, Clone No: MP5-20F3, Lot No: B263271), https://www.biolegend.com/ja-jp/products/apc-anti-mouse-il-6-antibody-7035
anti-Ly6G-FITC (BL-127606; BioLegend, Clone No: 1A8, Lot No: B277117), https://www.biolegend.com/ja-jp/products/fitc-anti-mouse-ly-6g-antibody-4775
anti-Alexa Fluor® 647 Rabbit IgG (BL-406414; BioLegend, Clone No: Poly4064, Lot No: B268583), https://www.biolegend.com/ja-jp/products/alexa-fluor-647-donkey-anti-rabbit-igg-minimal-x-reactivity-9379
anti-FITC Rat IgG2b isotype control (BL-400606; BioLegend, Clone No: RTK4530, Lot No: B265826), https://www.biolegend.com/ja-jp/products/fitc-rat-igg2b-kappa-isotype-ctrl-1854
anti-PE Mouse IgG2a isotype control (BL-400606; BioLegend, Clone No: MOPC-173, Lot No: B302650), https://www.biolegend.com/ja-jp/products/fitc-rat-igg2b-kappa-isotype-ctrl-1854
anti-PE Rat IgG2a isotype control (BL-400508; BioLegend, Clone No: RTK2758, Lot No: B290861), https://www.biolegend.com/ja-jp/products/pe-rat-igg2a-kappa-isotype-ctrl-1843
anti-APC Rat IgG2a isotype control (BL-400512; BioLegend, Clone No: RTK2758, Lot No: B284967), https://www.biolegend.com/ja-jp/products/apc-rat-igg2a-kappa-isotype-ctrl-1838
anti-APCfireTM Rat IgG2a isotype control (BL-400567; BioLegend, Clone No: RTK2758, Lot No: B304219), https://www.biolegend.com/ja-jp/products/apc-fire-750-rat-igg2a-kappa-isotype-ctrl-13013
anti-HRP mouse IgG (ab6820; Abcam, Lot No: GR3186683-12), https://www.abcam.co.jp/donkey-mouse-igg-hl-hrp-ab6820.html
anti-HRP rabbit IgG (ab97051; Abcam, Lot No: GR3231028-5), https://www.abcam.co.jp/goat-rabbit-igg-hl-hrp-ab97051.html
anti-HRP guinea pig IgG (ab97055; Abcam, Lot No: GR3175094-10), https://www.abcam.co.jp/goat-rat-igg-hl-biotin-ab97055.html

Anti-GPR68 (CSB-PA060199; CUSABIO, Lot No: G1421R2), anti-ARNTL (ab-93806; Abcam, Lot No: GR3191558-31), anti-CLOCK (ab-3517; Abcam, Lot No: GR322368-3), anti-phosphorylated STAT5A/B (Y694/699) (9359; Cell Signaling, Clone No: C11C5, Lot No: 0008), anti-STAT5A/B (sc-835; Santa Cruz Biotechnology, Lot No: D0714), anti-STRA6 (NBP1-00242; Novus Biologicals, Lot No: C2), anti-RBP4 (ab-109193; Abcam, Clone No: EP3657, Lot No: GR252174-10), anti-ACTB (SC-1616; Santa Cruz Biotechnology, Lot No: B1815), anti-TBP (ab-51841; Abcam, Clone No: mAbcam51841), anti-HRP mouse IgG (ab6820; Abcam, Lot No: GR3186683-12), anti-HRP rabbit IgG (ab97051; Abcam, Lot No: GR3231028-5), anti-HRP guinea pig IgG (ab97055; Abcam, Lot No: GR3175094-10) have been confirmed to be WB grade on the manufacturer's specification sheets. Anti-ARNTL (ab-93806; Abcam, Lot No: GR3191558-31), anti-CLOCK (ab-3517; Abcam, Lot No: GR322368-3), anti-STAT5A/B (sc-835; Santa Cruz Biotechnology, Lot No: D0714) have been confirmed to be ChIP grade on the manufacturer's specification sheets. Anti-GPR68 (CSB-PA060199; CUSABIO, Lot No: G1421R2), Cy3 anti-Rabbit IgG (AP132C; Sigma Aldrich), Alexa Fluor® 488 anti-Rat IgG (A11006; Invitrogen, Lot No: 1259366), anti-VCAM1 (ab134047; Abcam, Clone no: EPR5047), anti-F4/80 (MCA497RT; BioRad, Batch No: 1603) have been confirmed to be Immunofluorescence histochemical staining grade on the manufacturer's specification sheets. Anti-F4/80-APCfireTM (BL-123151; BioLegend, Clone No: BM8, Lot No:B235527), anti-F4/80-FITC (BL-123108; BioLegend, Clone No: BM8, Lot No: B222019), anti-CD11b-PE (BL-101208; BioLegend, Clone No: M1/70, Lot No: B253922), anti-Ly6C-APC (BL-128016; BioLegend, Clone No: HK1.4, Lot No: B234316), anti-Ly6C-FITC (BL-128006; BioLegend, Clone No: HK1.4, Lot No: B247728), anti-TNF-α-APC (BL-506307; BioLegend, Clone No: MP6-KT22, Lot No: B255366), anti-IL-6 (BL-504507; BioLegend, Clone No: MP5-20F3, Lot No: B263271), anti-Ly6G-FITC

(BL-127606; BioLegend, Clone No: 1A8, Lot No: B277117), anti-Alexa Fluor® 647 Rabbit IgG (BL-406414; BioLegend, Clone No: Poly4064, Lot No: B268583), anti-FITC Rat IgG2b isotype control (BL-400606; BioLegend, Clone No: RTK4530, Lot No: B265826), anti-PE Mouse IgG2a isotype control (BL-400606; BioLegend, Clone No: MOPC-173, Lot No: B302650), anti-PE Rat IgG2a isotype control (BL-400508; BioLegend, Clone No: RTK2758, Lot No: B290861), anti-APC Rat IgG2a isotype control (BL-400512; BioLegend, Clone No: RTK2758, Lot No: B284967), anti-APCfireTM Rat IgG2a isotype control (BL-400567; BioLegend, Clone No: RTK2758, Lot No: B304219) have been confirmed to be FCM grade on the manufacturer's specification sheets. Isotype controls were used to accurately determine the thresholds.

Eukaryotic cell lines

Policy information about cell lines

Cell line source(s)	Lenti-X™ 293T (CLN632180), which provided Takara Bio Co., Ltd. (Osaka, Japan)
Authentication	Cell line authenticated by STR method.
Mycoplasma contamination	Cell line were tested for mycoplasma contamination (Test result: Negative).
Commonly misidentified lines (See ICLAC register)	None of the used cell lines is listed in ICLAC database.

Animals and other organisms

Policy information about studies involving animals; ARRIVE guidelines recommended for reporting animal research

Laboratory animals	Clock mutant mice (C57BL/6J-ClockmlJt/J) were purchased from the Jackson Laboratory (Bar Harbor, ME, USA) and backcrossed to wild-type Jcl: ICR mice (Charles River Laboratory Japan, Inc.; Yokohama, Japan) for more than eight generations to improve breeding and offspring care. Male ICR mice were housed in a light-controlled room (lights on from ZT0 to ZT12) at 24 ± 1 °C and 60% ± 10% humidity, and the animals had free access to water and AIN-93G pelleted diet (positive control diet groups contained 4000 IU of vitamin A/kg) or a vitamin-A-free AIN-93G pelleted diet (Oriental Yeast Co., ltd.).
Wild animals	This study did not involve wild animals.
Field-collected samples	This study did not involve samples collected from the field
Ethics oversight	All animal experiments were conducted in accordance with the Guidelines for Animal Experiments of Kyushu University and were approved by the Institutional Animal Care and Use Committee of Kyushu University (approved protocol ID #A30-061).

Note that full information on the approval of the study protocol must also be provided in the manuscript.

Human research participants

Policy information about studies involving human research participants

Population characteristics	Human samples from healthy subjects male individuals with no diagnosed disease at the time of collection, which were used for culture of human primary monocytes.<br><br>Human serum from healthy subjects and CKD patients without diagnosed diabetes were obtained from Nagasaki Kamigoto Hospital and Kyushu University Hospital. The patients were women aged 21-42 years or men aged 26-87 years. There were no abnormal levels of total protein, total cholesterol, and sugar concentrations in collected serum samples.<br>All values of measured parameters in blood were described in Figure and Supplementary information.
Recruitment	About human samples from healthy subjects which were used for culture of human primary monocytes, investigators of this manuscript did not participate in the recruitment of human participants or received any patient identifiers. The vendors was responsible for recruitment of donors and sample collection.<br><br>About human serum from healthy subjects and CKD patients, participants were recruited following standard procedures in Nagasaki Kamigoto Hospital and Kyushu University Hospital.<br>All experiments using samples from healthy subjects and CKD patients were approved by the institutional review board of Nagasaki Kamigoto Hospital or Kyushu University Hospital, and written informed consent was obtained from all patients.<br>Approval number: 26-11 (Nagasaki Kamigoto Hospital), 757-00 (Kyushu University Hospital)<br><br>To eliminate bias due to diseases other than CKD, samples were selected by the doctors who performed the serum collection based on what is described in "Population characteristics". The doctors performed this collection and selection without knowing the serum parameters that were expected to significantly affect the claims of this study, such as retinol and RBP4 concentration. There is no reason to believe that these selection criteria would introduce a selection bias or otherwise impact the results.
Ethics oversight	The handling of serum samples from healthy subjects and CKD patients, and experimental protocol were approved by the research ethics committees of Nagasaki Kamigoto Hospital (approved protocol ID #26-11) and Kyushu University Hospital (approved protocol ID # 757-00), and are complied with all relevant ethical regulations. BioIVT and BIOPRREDIC collected from consented donors under their IRB approved protocols at FDA registered donor centers and expansive clinical collection network. Written informed consent was obtained from all subjects.

Note that full information on the approval of the study protocol must also be provided in the manuscript.

Flow Cytometry

Plots

Confirm that:

☒ The axis labels state the marker and fluorochrome used (e.g. CD4-FITC).

☒ The axis scales are clearly visible. Include numbers along axes only for bottom left plot of group (a 'group' is an analysis of identical markers).

☒ All plots are contour plots with outliers or pseudocolor plots.

☒ A numerical value for number of cells or percentage (with statistics) is provided.

Methodology

Sample preparation	The description is stated in Methods section.<br>Cardiac/renal-derived cells: Eight weeks after the 5/6Nx operation, the heart was exposed and perfused with 10 mL of PBS from the left ventricle. After removing the atria, the ventricles were digested in PBS containing 500 µg/mL of collagenase type II (FUJIFILM Wako Pure Chemical Corporation), 200 µg/mL of CaCl2 (Nacalai Tesque), 0.05% trypsin (Sigma-Aldrich), and 10% FBS at 37°C for 10 min with agitation. Digested samples were further dissociated by passing them through a 23-gauge needle three times. After treating with Red Blood Lysis buffer (BioLegend, CA, USA), isolated cells were filtered through a 40-µm strainer.<br>Bone marrow, spleen and blood cells: Eight weeks after the 5/6Nx operation, bone marrow, spleen and blood were collected. After treating with Red Blood Lysis buffer (BioLegend, CA, USA), isolated cells were filtered through a 40-µm strainer.
Instrument	Isolated cells were stained with fluorochrome-conjugated antibody.<br>For cell sorting: FACSAria III (Becton Dickinson).<br>For analysis: FACSCanto III (Becton Dickinson).
Software	FACSDiva for collection and FlowJo (v10) for analysis
Cell population abundance	Populations were validated for purity by a post-sort analysis by FACS.
Gating strategy	The description is stated in Figure, Supplementary information and Methods section.<br>Cardiac/renal CD11b+/Ly6C+ monocytes: F4/80+/Ly6G-/Ly6C+/CD11b+<br>CD11b+/Ly6C+ monocytes in bone marrow, spleen and blood: Ly6C+/CD11b+ live cells

☒ Tick this box to confirm that a figure exemplifying the gating strategy is provided in the Supplementary Information.
